# 3,3-Bis(hydroxyaryl)oxindoles and Spirooxindoles Bearing a Xanthene Moiety: Synthesis, Mechanism, and Biological Activity

**DOI:** 10.1021/acs.joc.5c00270

**Published:** 2025-05-07

**Authors:** Dániel Steinsits, Bettina Rávai, Zsolt Kelemen, László Hackler, Viktor Vernyik, László G. Puskás, Erika Bálint

**Affiliations:** † Department of Organic Chemistry and Technology, Faculty of Chemical Technology and Biotechnology, Budapest University of Technology and Economics, Műegyetem rkp. 3, H-1111 Budapest, Hungary; ‡ CycloLab Cyclodextrin Research and Development Ltd., Illatos út 7, H-1097 Budapest, Hungary; § Department of Inorganic and Analytical Chemistry, Faculty of Chemical Technology and Biotechnology, Budapest University of Technology and Economics, Műegyetem rkp. 3, H-1111 Budapest, Hungary; ∥ Anthelos Kft., Alsó kikötő sor 11., H-6726 Szeged, Hungary

## Abstract

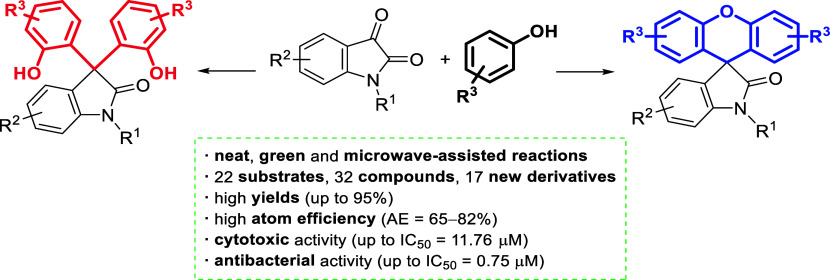

A facile and efficient methanesulfonic acid-catalyzed, solvent-free, microwave-assisted method was developed for the synthesis of biologically active 3,3-bis(hydroxyaryl)oxindoles and spirooxindoles bearing a xanthene moiety. The scope of the procedure was investigated with a wide range of isatin and phenol derivatives; moreover, the reaction mechanism was studied by density functional theory calculations. Both 3,3-bis(hydroxyaryl)oxindoles and spirooxindoles bearing a xanthene moiety synthesized were evaluated for their anticancer and antimicrobial activity, and most of them showed promising or significant activity on six cancer cell lines and against Gram-positive bacteria.

## Introduction

Isatin derivatives are pharmaceutically interesting molecules due to their various biological properties, including central nervous system, antimicrobial, antiviral, antioxidant, and anticancer activity.^1−4^ Xanthenes exhibit similar biological activities,^5^ which along with their use as traditional and fluorescent dyes,^6^ make them valuable molecules both industrially and pharmaceutically. In addition, xanthenes can also be used in photodynamic therapy,^7^ chemoanalytics,^8,9^ biological imaging, and bioanalytics.^10^

**Scheme 1:**
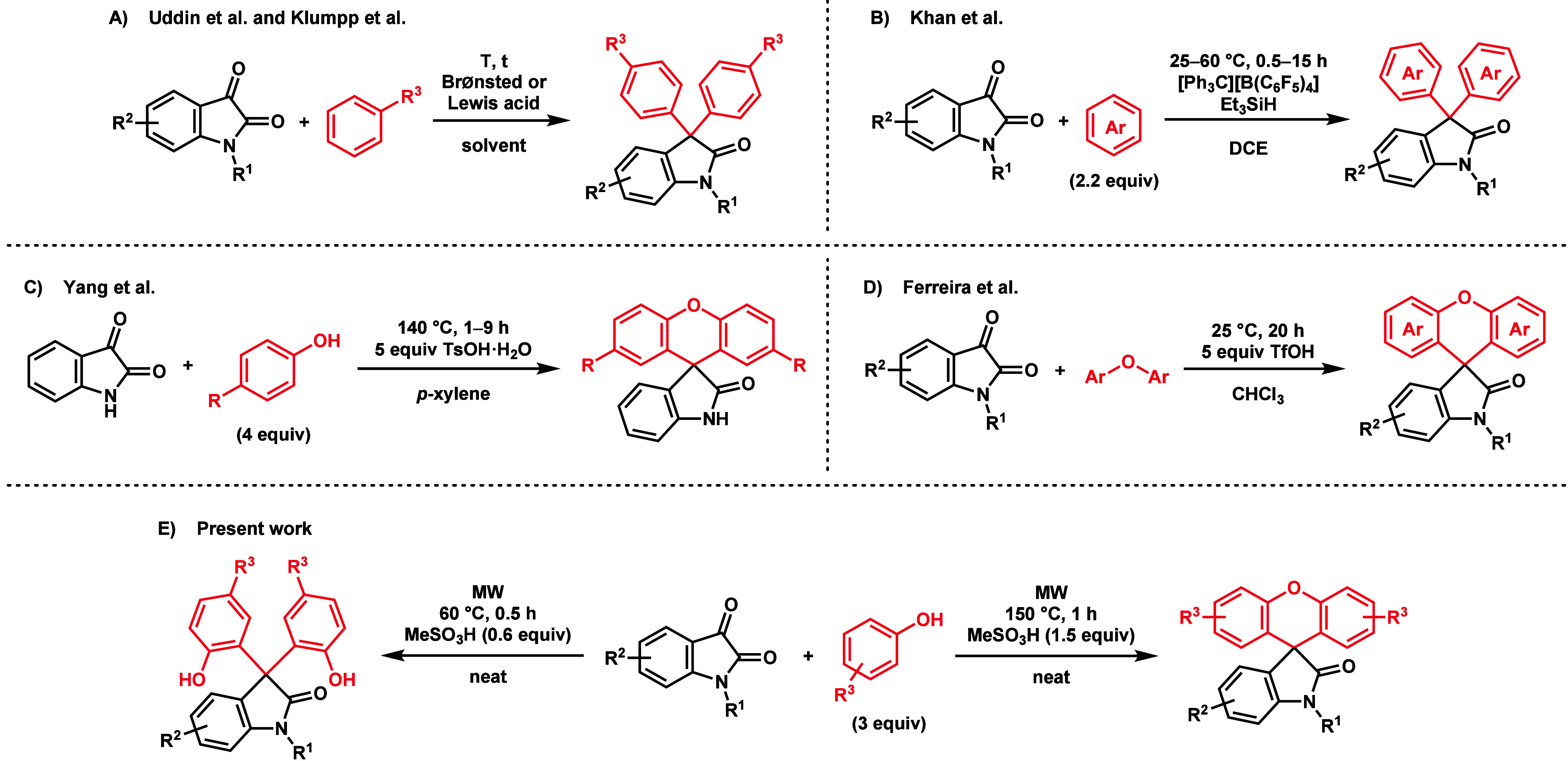
(A–D) Previous Work Related to C3-Diarylation and Xanthene Formation on Isatin Platforms and (E) This Work Utilizing MW-Assisted Solvent-Free Reaction

One group of the biologically active isatin derivatives consists of 3,3-bis(hydroxyaryl)oxindoles, showing promising antiproliferative activity.^11^ The simplest synthetic approach for the structurally similar, symmetrical 3,3-diarlyoxindoles is the reaction of isatins and arenes under Brønsted or Lewis acid catalysis in a Friedel–Crafts-type reaction (Scheme 1A). Recently, highly efficient and sustainable versions of such reactions were described by Singh et al. transforming various aldehydes and ketones.^12^ For the synthesis of symmetrical 3,3-diaryloxindoles, Klumpp et al. developed a method in which isatins were reacted with benzene derivatives in triflic acid (TfOH).^13^ The procedure was only applied to a limited number of isatins and alkyl- and halobenzenes. Uddin et al. investigated the reaction of various isatins with phenol in the presence of Lewis acids (AcOH, H_2_SO_4_, and TfOH), affording low to moderate yields.^11^ Recent advancements in the field include the procedure developed by Khan et al., using *in situ*-generated Lambert salt as a catalyst, a wide selection of arenes, and a handful of isatins (Scheme 1B).^14^

Since both isatins and xanthenes possess biologically active properties, the idea of combining them by synthesizing spirooxindoles containing a xanthene moiety would likely result in valuable molecular structures. So far, the research of these compounds is limited, and in most cases, the synthesis strategies starting from isatins use large amounts of acid catalysts, long reaction times, and non-green organic solvents. One such method was developed by Yang et al. using isatin and 4 equiv of *para*-substituted phenols in the presence of 5 equiv of *p*-toluenesulfonic acid (TsOH·H_2_O) (Scheme 1C).^15^ The scope of this method was only investigated with unsubstituted isatin and *p*-alkyl- and *p*-alkoxyphenols. In another method, Ferreira et al. utilized various substituted isatins and *para*-substituted biaryl ethers along with 5 equiv of TfOH to form the desired products. However, only a limited number of biaryl ethers were investigated (Me, F, Br, and naphthyl) (Scheme 1D).^16^

Without a doubt, these examples suffer from low efficiency, a non-green nature, toxic or exotic chemicals, and the lack of a comprehensive substituent scope, as well as biological evaluation. Thus, in this paper, our aim was to develop a more efficient synthetic procedure for both types of oxindoles utilizing microwave (MW) irradiation, with reduced equivalents and green or no solvent at all. In addition, mechanistic quantum chemical calculations and biological evaluation, such as *in vitro* cytotoxicity and antibacterial assays, were also performed.

## Results and Discussion

First, the optimization of the acid-catalyzed reaction of isatin and *p*-cresol toward the formation of the 3,3-bis(hydroxyaryl)oxindole (**3a**) was investigated (Table 1). Initially, 4 equiv of *p*-cresol (**2a**) and 0.5 equiv of TsOH·H_2_O as a catalyst were used at room temperature, which resulted in a total conversion to **3a** in 20 h (Table 1, entry 1). Using conventional heating (oil bath) at 60 °C, only 1 h was enough to obtain the same result (Table 1, entry 2). Switching to MW conditions, a conversion of 78% was observed after 10 min (Table 1, entry 3). During the screening of other possible catalysts, methanesulfonic acid (MeSO_3_H) resulted in a higher conversion of 92% (Table 1, entry 4), while other Brønsted and Lewis acidic catalysts or dehydrating agent (T_3_P, propanephosphonic acid anhydride) showed no reaction at all (Table 1, entries 5–7). Decreasing the amount of *p*-cresol could be balanced by increasing the reaction time in the case of 3 equiv of *p*-cresol (**2a**), while a further decrease demanded larger amounts of the catalyst (Table 1, entries 8 and 9). Eventually, three alternative optimal combinations were developed. Upon application of 2 equiv of *p*-cresol (**2a**), the use of 0.7 equiv of MeSO_3_H and a somewhat longer reaction time were sufficient for complete conversion (Table 1, entry 10). Carrying out the reaction with 3 equiv of *p*-cresol (**2a**), only 0.6 equiv of the catalyst and a reaction time of 30 min were necessary (Table 1, entry 11). It should be noted that the reaction conditions applied in entry 10 proved to be inadequate when they were applied to substituted isatins, as the smaller amount of *p*-cresol (**2a**) did not provide sufficient media for the reaction, necessitating the development of entry 11. Similarly, using TsOH·H_2_O under the same conditions, as in entry 11, also resulted in full conversion, although with a lower atom efficiency and a higher price (Table 1, entry 12). Furthermore, the optimized procedure is much greener than other acid-catalyzed methods and is on par with the method of Khan et al., with an atom efficiency of 82% and an *E*-factor of 0.61 (Table S3).

**Table 1:**
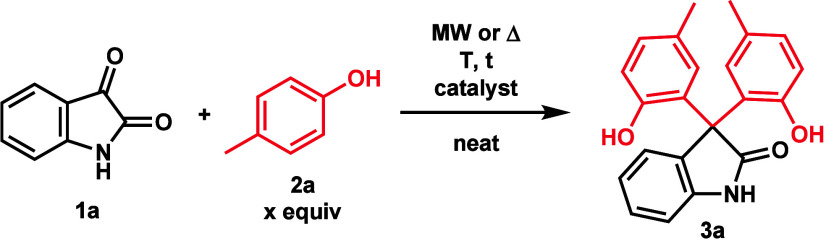
Optimization for the Synthesis of **3a**^a^

entry	heating method	*x* equiv	catalyst (equiv)	*T* (°C)	*t* (min)	yield of **3a**^b^ (%)
1	–	4	TsOH·H_2_O (0.5)	RT	1200	100
2	Δ	4	TsOH·H_2_O (0.5)	60	60	99
3	MW	4	TsOH·H_2_O (0.5)	60	10	78
4	MW	4	MeSO_3_H (0.5)	60	10	92
5	MW	4	TFA (0.5)	60	10	0
6	MW	4	Zn(OTf)_2_ (0.1)	60	10	0
7	MW	4	T_3_P (0.2)	60	10	0
8	MW	3	MeSO_3_H (0.5)	60	20	97
9	MW	2	MeSO_3_H (0.6)	60	20	97
10	MW	2	MeSO_3_H (0.7)	60	40	100
11	MW	3	MeSO_3_H (0.6)	60	30	100
12	MW	3	TsOH·H_2_O (0.6)	60	30	100

a For the reactions, 0.25 mmol of isatin (**1a**), 2–4 equiv of *p*-cresol, and 0.2–0.7 equiv of the catalyst were stirred under MW irradiation under neat conditions.

b Conversion determined by RP-HPLC-MS DAD (256 nm).

**Figure 1:**
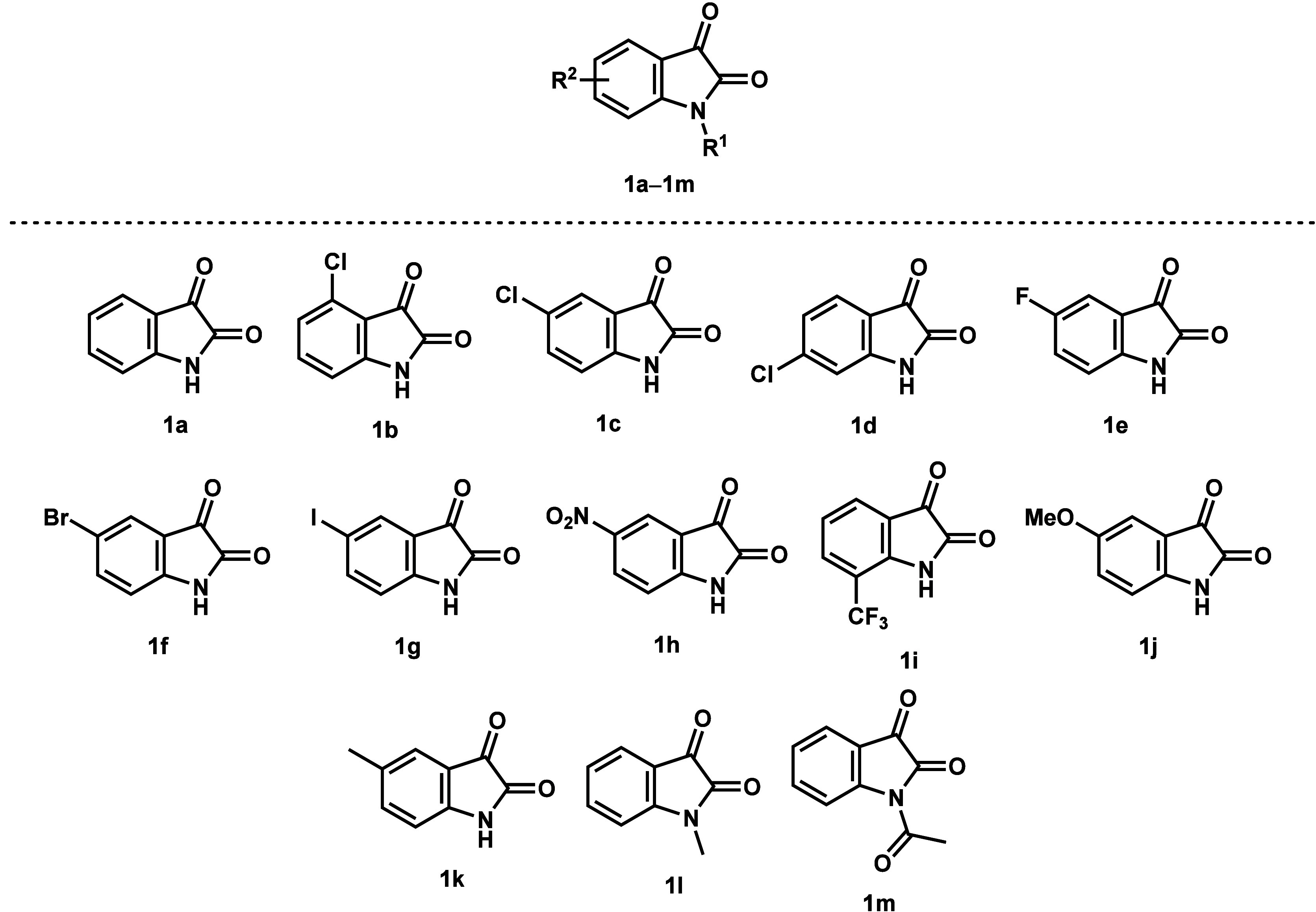
All investigated isatin derivatives (**1a–m**).

**Figure 2:**
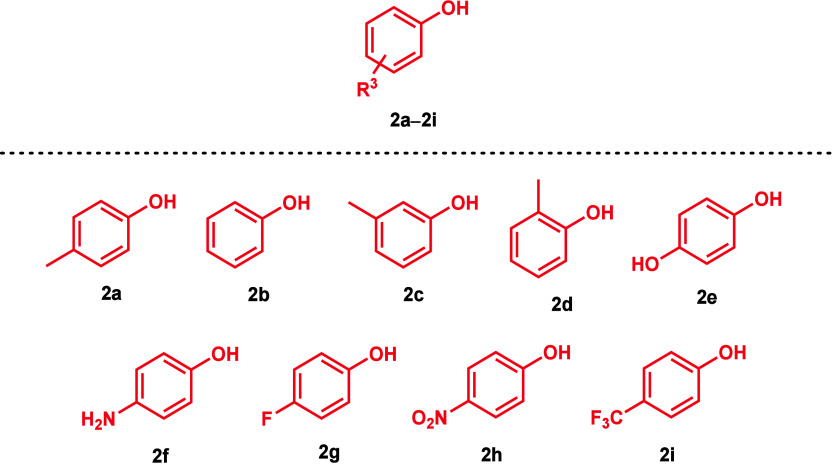
All investigated phenol derivatives (**2a–i**).

**Scheme 2:**
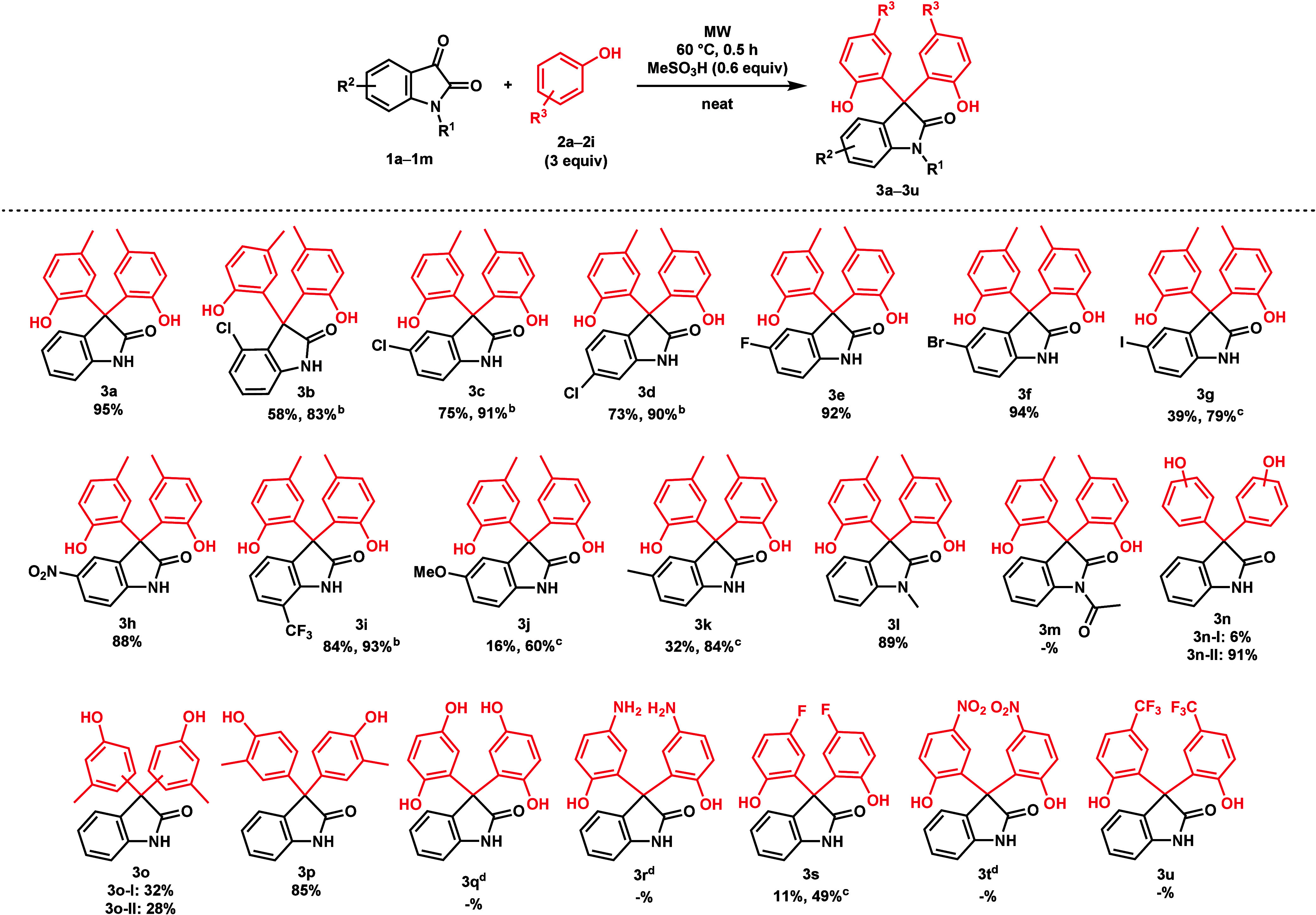
Substrate Scope for the Synthesis of Compounds **3** For the reactions, 1.00 mmol of the isatin derivative (**1**) was reacted with 3 equiv of a phenol (**2**) derivative in the presence of 0.6 equiv of MeSO_3_H at 60 °C for 30 min under MW irradiation under neat conditions. At 80 °C. At 90 °C for 90 min with 1 equiv of MeSO_3_H. With 0.6 mL of EtOAc.

**Scheme 3:**
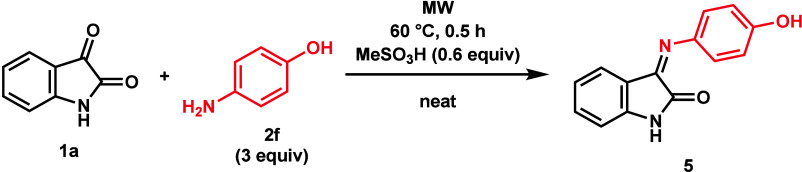
Likely Side Reaction of **1a** and **2f**, Leading to the Formation of **5**

With the optimized conditions in hand, the reaction was extended using various isatins (**1**) and phenols (**2**) (Figures 1 and 2 and Scheme 2). First, substituted isatins **1b**–**m** were investigated with *p*-cresol (**2a**). Using chloroisatins (**1b**–**d**), slightly lower yields of product **3b**–**d** were obtained compared to the unsubstituted isatin (**1a**), although with 4-Cl-isatin (**1d**) only a yield of 58% was achieved due to the formation of **4b** in 10% yield. Increasing the temperature to 80 °C increased the yield for all three chloroisatin products (**3b**–**d**). Other halogenated isatins **1e** and **1f** provided high yields, except for 5-I-isatin (**1g**). It should be emphasized that at 90 °C deiodination occurred, resulting in a yield of **3a** of 9%. Isatins containing other electron-withdrawing groups, such as nitro compound **1h** and trifluoromethyl compound **1i**, also provided 3,3-bis(hydroxyaryl)oxindoles **3h** and **3i**, respectively, in good yields. Increasing the temperature to 80 °C in the case of **1i** increased the yield to 93%. Electron-donating groups significantly decreased the yields for methoxy derivative **3j** and methyl derivative **3k**. Thus, changing the conditions to 90 °C and 90 min and using 1 equiv of catalyst, the yields of **3j** and **3k** were increased. The *N*-substituted isatins (*N*-Me **1l** and *N*-Ac **1m**) were also investigated. The *N*-Me-product (**3l**) was prepared with a yield of 89%, while the *N*-Ac-isatin (**1m**) was hydrolyzed due to the acidic conditions. Various phenol derivatives **2b**–**i** were also reacted with **1a**. Unsubstituted phenol (**2b**) provided two isomers (**3n-I** and **3n-II**) with the isomer containing two *para*-positioned phenol rings forming in a higher ratio (*o*–*o*, 6% yield; *p*–*p*, 91% yield). *m*-Cresol (**2c**) provided three isomers in about a 1:1:1 ratio, but only two of them could be sufficiently purified: **3o-I** (32%) and **3o-II** (28%). Similarly to the unsubstituted phenol (**2b**), *o*-cresol (**2d**) provided two isomers, but only one isomer (**3p**) could be isolated, as the other was only formed in trace amounts. Furthermore, hydroquinone (**2e**) formed xanthene **4q** instead of **3q**, probably due to the higher reactivity of **3q** toward ring closure, making xanthene formation preferable. Likewise, aminophenol derivative **3r** could not be prepared, as a Schiff base (**5**) was formed with *p*-aminophenol (**2f**), as expected based on the literature (Scheme 3).^17^ With the exception of fluorophenol **2g**, highly electron-deficient phenols **2h** and **2i** degraded and did not form the desired products **3t** and **3u**, respectively. Even *p*-F-phenol (**2g**) provided **3s** in yields of only 11% and 49%, depending on the conditions.

Afterward, the synthesis of spiroxanthenes (**4**) was investigated (Table 2). Since the formation of **4a** was not observed at lower temperatures, we started to investigate the reaction at a higher temperature. As expected, this favored the formation of **4a** (Table 2, entries 1–3). Applying a larger amount of MeSO_3_H increased while decreasing the amount of *p*-cresol (**2a**) decreased the conversion of **1a** into product **4a** (Table 2, entries 4 and 5, respectively). Even larger amounts of a catalyst slightly increased the conversion, as did an increase in the amount of *p*-cresol (**2a**) (Table 2, entries 6 and 7). The use of 2.5 equiv of catalyst resulted in practically the same conversion as 1.5 equiv (Table 2, entry 8). This indicates that the ring-closing step does not depend strongly on the acidity of the media. Finally, the total conversion of **3a** was achieved by increasing the reaction time to 1 h (Table 2, entry 9). For this procedure, the green chemical metrics are much better than those previously reported, with an atom efficiency of 64.5% and an *E*-factor of 1.14 (Table S3).

**Table 2:**
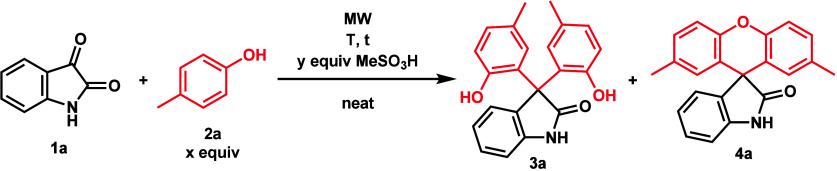
Optimization for the Synthesis of **4a**^a^

Entry	x equiv	y equiv	T (°C)	t (h)	**3a**^b^ (%)	**4a**^b^ (%)	Other^b^,^c^ (%)
1	3	0.6	80	0.5	100	0	0
2	3	0.6	120	0.5	93	7	0
3	3	0.6	150	0.5	24	68	8
4	3	1.0	150	0.5	11	81	8
5	2	1.0	150	0.5	33	55	12^d^
6	3	1.5	150	0.5	4	88	8
7	4	1.5	150	0.5	12	84	4
8	3	2.5	150	0.5	4	87	9
9	3	1.5	150	1.0	0	92	8

a For the reactions, 0.25 mmol of isatin (**1a**), 2–3 equiv of *p*-cresol, and 0.6–2.5 equiv of a catalyst were stirred under MW irradiation under neat conditions.

b Ratio determined by RP-HPLC-MS DAD (256 nm).

c Various degradation products.

d With 7% isatin (**1a**).

**Figure 3:**
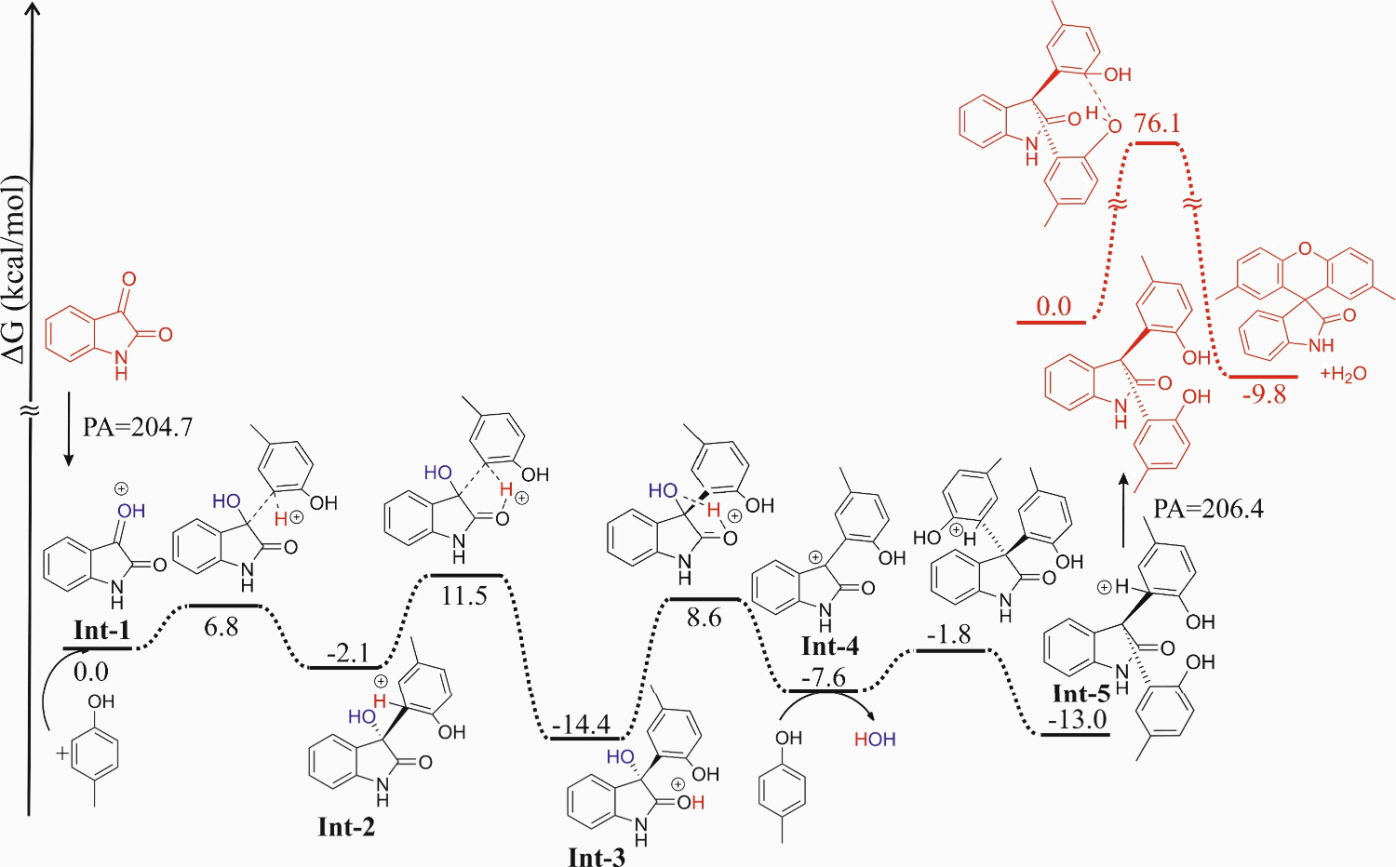
Computed reaction mechanism. Relative Gibbs free energy values are given in kilocalories per mole.

To better understand the reaction mechanism (Figure 3), density functional theory calculations were performed at the ωB97X-D/def2-TZVP level of theory. The initial step involves the protonation of isatin (**1a**) (the computed proton affinity is 204.7 kcal/mol), which allows the nucleophilic attack of *p*-cresol (**2a**) (formation of **Int-2**). This process has a low energy barrier of 6.8 kcal/mol. In the following step, the proton migrates to the carbonyl functional group of the isatin ring, which has a somewhat higher reaction barrier (11.5 kcal/mol) than the previous nucleophilic attack. The formed **Int-3** intermediate can be considered as a resting state (Δ*G* = −14.4 kcal/mol) of the process. The movement of the proton to the OH group in position 3 results in the formation of intermediate **Int-4** (Δ*G* = −7.6 kcal/mol), which is ready for nucleophilic attack of the second *p*-cresol (**2a**). After deprotonation of formed intermediate **Int-5**, product **3a** is obtained. The mechanism until this step involves low barriers, which is in full agreement with the observation that the reaction proceeds even at room temperature. Investigating the ring-closing step, the monomolecular proton migration is assisted by the carbonyl group. This step has a high barrier (76.0 kcal/mol), which is consistent with the applied harsher reaction conditions. At this point, it should be highlighted that proton migration can be assisted by solvent molecules, which can significantly decrease the computed monomolecular reaction barrier.

**Scheme 4:**
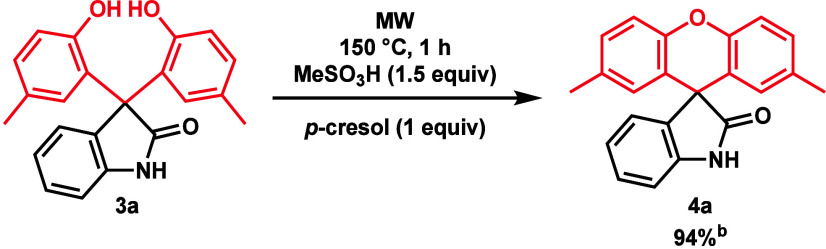
Transformation of **3a** into **4a** For the reaction, 0.15 mmol of prepared 3,3-bis(2-hydroxy-5-methylphenyl)indolin-2-one (**3a**) was stirred for 1 h at 150 °C in the presence of 1.5 equiv of MeSO_3_H in 1 equiv of *p*-cresol (**2a**) under MW irradiation. Conversion determined by RP-HPLC-MS DAD (256 nm).

To verify that the spirooxindole formation proceeds via ring closure of **3a**, we investigated the transformation of **3a** into **4a**. Using the previously optimized reaction conditions, a conversion of 94% was obtained, which verified our original hypotheses (Scheme 4).

**Scheme 5:**
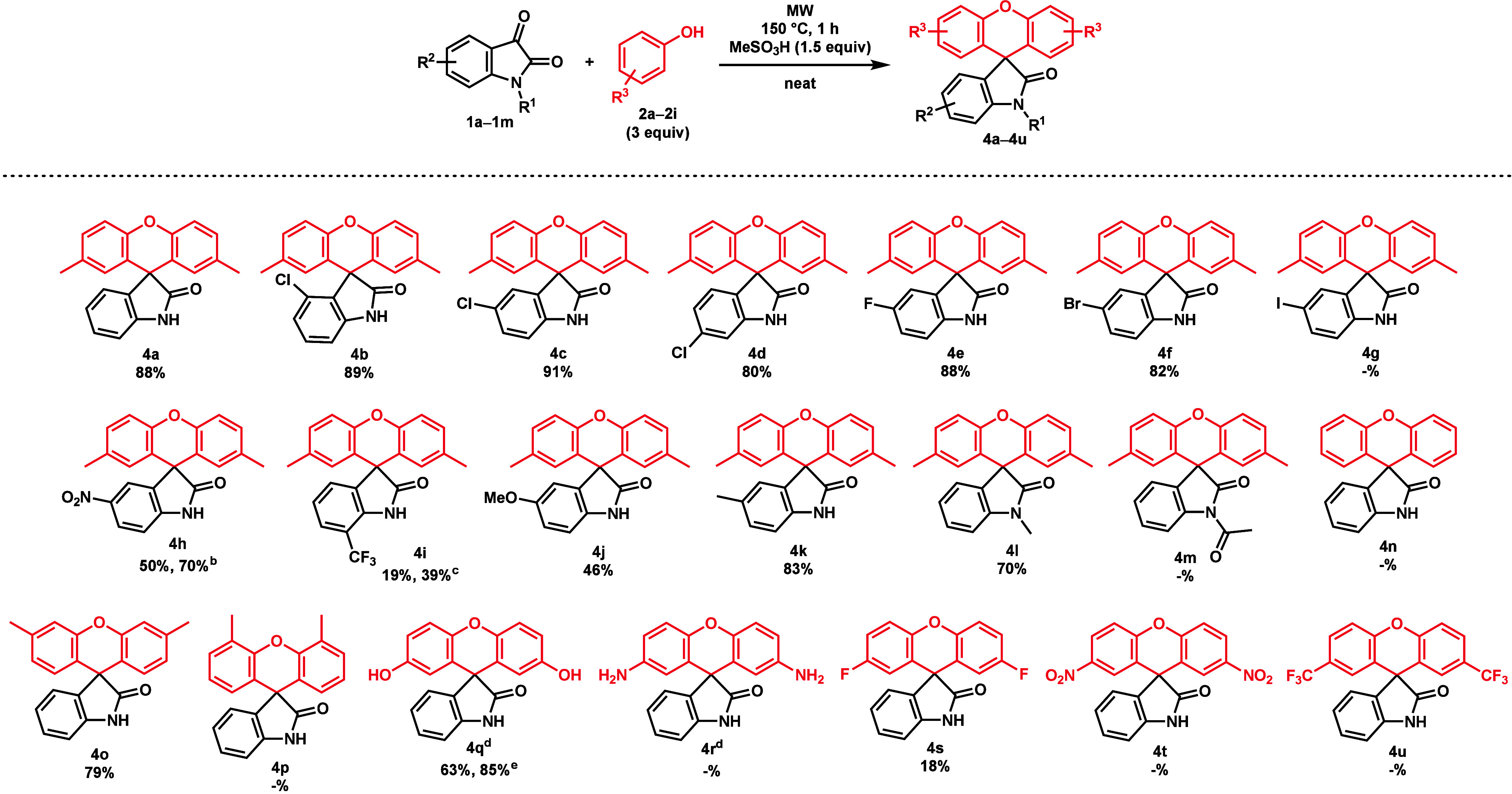
Substrate Scope for the Synthesis of **4** For the reactions, 1.00 mmol of an isatin derivative (**1**) was reacted with 3 equiv of a phenol (**2**) derivative in the presence of 1.5 equiv of MeSO_3_H at 150 °C for 1 h under MW irradiation under neat conditions. At 135 °C for 80 min. At 135 °C for 80 min with 4 equiv of *p*-cresol. With 0.6 mL of EtOAc. At 90 °C for 45 min.

**Scheme 6:**
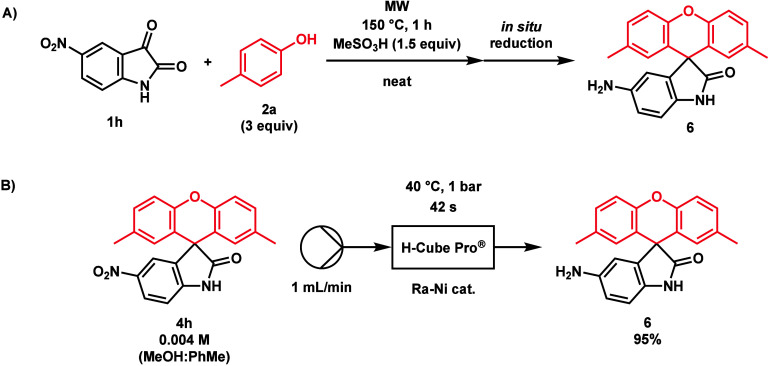
(A) Likely Side Reaction Occurring When Synthesizing **4h** and (B) Our Reduction of **4h** to **6** In a ThalesNano H-Cube Pro^Ⓡ^ flow reactor, a solution of **4h**, made by 10 mg of **4h** dissolved in 7 mL of a 1:1 MeOH/PhMe mixture, was pumped with a flow rate of 1 mL/min, hydrogenated at 40 °C with a Raney nickel catalyst.

**Scheme 7:**
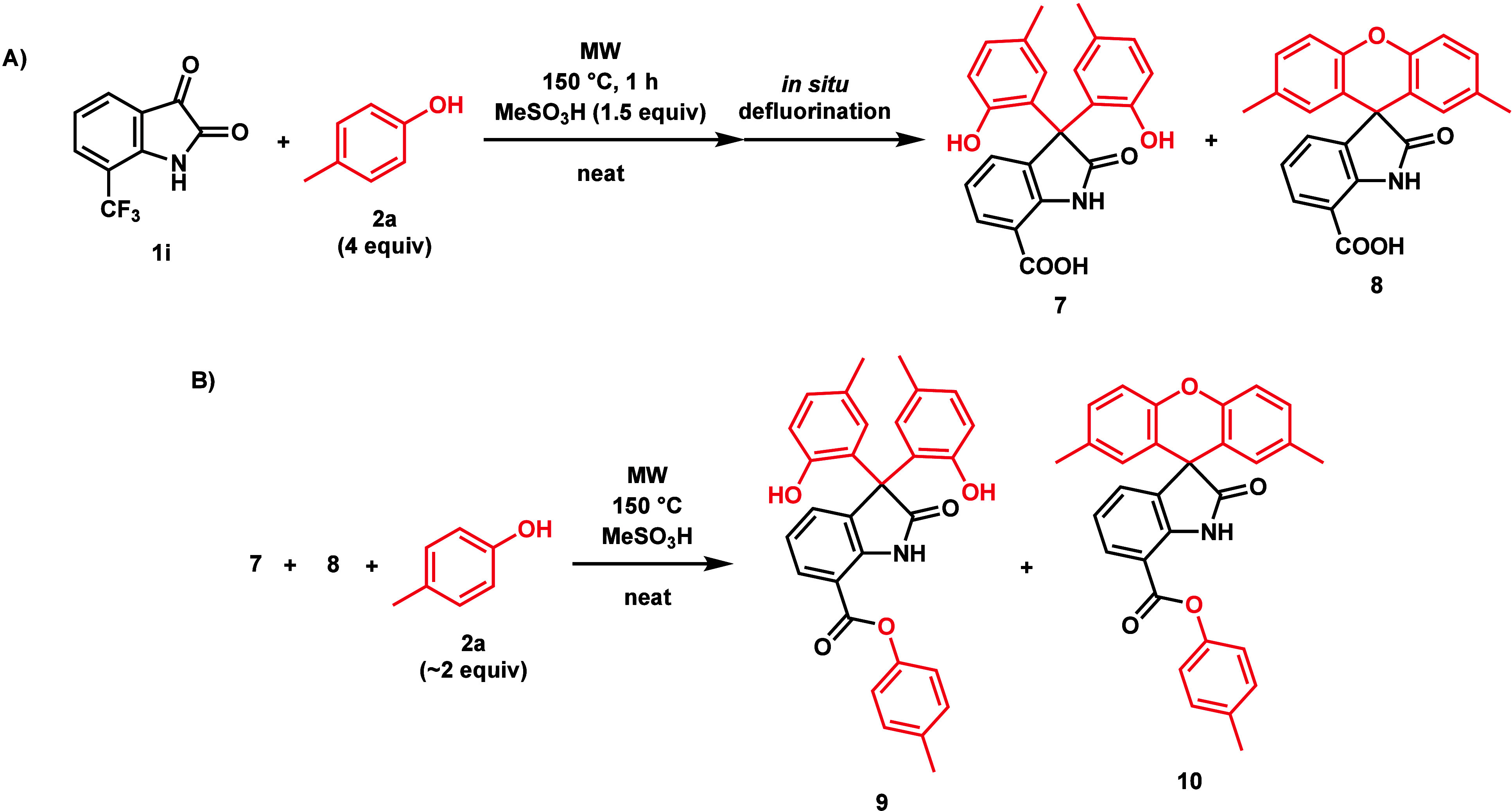
(A) Likely Side Reaction Occurring When Synthesizing **4i**, Producing Defluorinated Side Product **7** and **8** and (B) Their Esterification by Excess *p*-Cresol

To broaden the scope of the reaction, the formation of the corresponding spirooxindole (**4**) was investigated starting from the same isatins (**1b**–**m**) and phenols (**2a**–**l**) previously investigated (Scheme 5). Once again, substituted isatins (**1b**–**m**) were reacted with *p*-cresol (**2a**). This time, all chloroisatins (**1b**–**d**) provided similar yields. Other halogenated isatins such as fluoroisatin (**1e**) and bromoisatin (**1f**) provided products **4e** and **4f** in yields of 88% and 82%, respectively, except 5-I-isatin (**1g**), which was deiodinated even at 120 °C. The other two electron-deficient isatins (5-NO_2_**1h** and 7-CF_3_**1i**) displayed unique side reactions under the optimized conditions. Compound **4h** was isolated in a yield of 50%, due to the 5-NO_2_ group being reduced to a 5-NH_2_ side product (**6**) (Scheme 6). 7-CF_3_-isatin (**1i**) only afforded product **4i** in 16% yield due to a side reaction producing defluorinated, 7-carboxyl analogues (**7** and **8**) of **3i** and **4i** (Scheme 7), and etching the glass of the MW reaction vials, which was accompanied by an unpleasant irritating odor, suggesting hydrogen fluoride formation. A similar observation was made by Klummp et al. using (trifluoromethyl)benzene and TfOH.^18^ In the case of product **4h**, applying a temperature of 135 °C for 80 min increased the yield to 70%. By the reduction of compound **4h** using a hydrogenation H-Cube Pro^Ⓡ^ flow reactor, the 5-NH_2_ side product (**6**) was prepared and characterized (Scheme 6). Similarly, the yield of product **4i** could be increased by applying 4 equiv of *p*-cresol (**2a**) at 135 °C for 80 min. Interestingly, this created two ester derivatives (**9** and **10**) of the defluorinated side products (**7** and **8**), one of which (**10**) was isolated and characterized (Scheme 7). Electron-donating groups did not directly affect the reaction as 5-Me compound **4k** was prepared in 83% yield, but **4j** could only be isolated in a yield of 46% due to hydrolysis during the reaction and workup. A yield of 70% was observed in the case of *N*-Me product **4l**, while *N*-acetyl spiroxanthene **4m** did not form due to hydrolysis. Unsubstituted phenol (**2b**) did not produce compound **4n**, as the reaction mixture was heavily degraded even at 120 °C. *m*-Cresol (**2c**) reacted well (79%), while using *o*-cresol (**2d**), a degradation similar to that of phenol (**2b**) occurred. The hydroquinone derivative (**4q**) could be obtained in good yield (85%) in the presence of 0.6 equiv of MeSO_3_H, at 90 °C for 30 min, while under the optimized conditions, a significant oxidation was observed, leading to a lower yield. *p*-Aminophenol (**2f**) formed the aforementioned Schiff base (**5**), which degraded at higher temperatures; thus, spiroxanthene **4r** could not be prepared. The electron-deficient phenol products (**4t** and **4u**) could not be synthesized. Upon application of *p*-CF_3_-phenol (**2i**), a strong degradation producing voluminous black degradation products was observed. The product of *p*-F-phenol (**4s**) was again an exception but was isolated in only a yield of 18%.

The *in vitro* cytotoxicity evaluations were carried out on six different cancer cell lines, such as 4T1 breast cancer, A549 human lung adenocarcinoma, B16 mouse melanoma, HT29 human colorectal adenocarcinoma, HT168 human melanoma, and HL-60 human promyelocytic leukemia cell lines. During the measurements, the fluorescent resazurin assay was applied, and doxorubicin was the positive control. The IC_50_ values (50% inhibitory concentration) obtained for the active molecules are shown in Table 3.

**Table 3:**
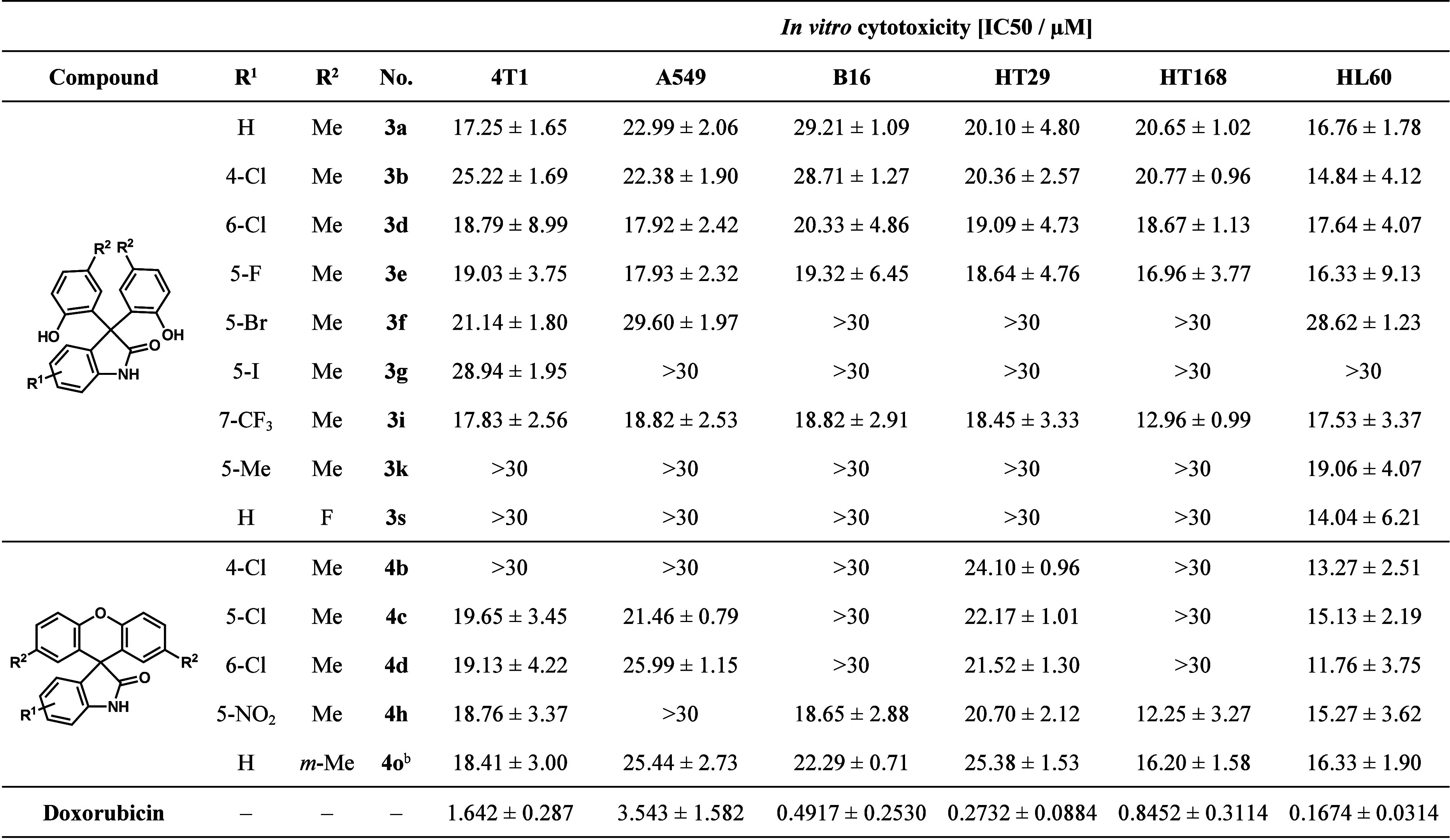
*In Vitro* Cytotoxicity Study Data of Active Compounds **3** and **4** on Six Different Cell Lines^a^

a Data are expressed as mean ± standard deviation.

b *m*-Cresol (**2c**) derivative.

Altogether, nine 3,3-bis(hydroxyaryl)oxindoles (**3a**, **3b**, **3d–g**, **3i**, **3k**, and **3s**) and five spiroxanthenes (**4b–d**, **4h**, and **4o**) proved to be active against the cancer cells investigated. The unsubstituted 3,3-bis(hydroxyaryl)oxindole (**3a**) and those that contain a 4-Cl (**3b**), 6-Cl (**3d**), 5-F (**3e**), or 7-CF_3_ (**3i**) substituent on the isatin ring showed moderate or promising activity (IC_50_ = 12.96 ± 0.99 to 29.21 ± 1.09 μM) against all six cell lines. The 5-Br-substituted derivative (**3f**) also showed modest activity against 4T1, A549, and HL60 cells. The 5-I-substituted compound (**3g**) was only active on the 4T1 cell line. Products **3k** and **3s** showed 19.06 ± 4.07 and 14.04 ± 6.21 μM activity against the human leukemia cell line (HL-60), respectively. Among spirooxindoles, chloro- and nitro-substituted isatin derivatives (**4b–d** and **4h**) were active. Among the phenol-substituted compounds, only one of the *m*-cresol derivatives showed activity, however, against all six cell lines studied (16.20 ± 1.58 to 25.44 ± 2.73 μM). The most active compounds proved to be the 7-CF_3_-substituted 3,3-bis(hydroxyaryl)oxindole (**3i**), for which an IC_50_ value of 12.96 ± 0.99 μM was obtained against HT168 cells, as well as the 6-Cl-substituted spirooxindole (**4d**), which showed 11.76 ± 3.75 μM activity on the HL60 cell line.

The antibacterial activity of the compounds was tested on green fluorescent protein (GFP) producing *Bacillus subtilis* (Gram-positive) and *Escherichia coli* (Gram-negative) bacterial cells. Positive controls were doxycycline and gentamicin. The IC_50_ values (50% inhibiting concentration) obtained for the active molecules are shown in Table 4.

**Table 4:**
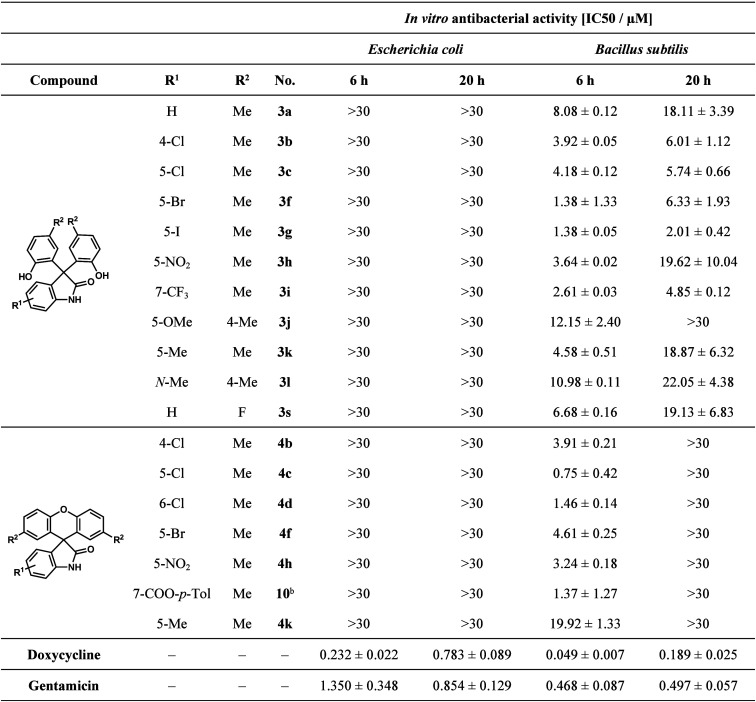
*In Vitro* Antibacterial Study Data of Active Compounds **3** and **4** on Gram-Negative and Gram-Positive Bacteria^a^

a Data are expressed as mean ± standard deviation.

b *p*-Tolyl ester of defluorinated **4i**.

According to the results of the antibacterial activity studies, none of the derivatives were active against selected *E. coli* bacteria; however, the growth of *B. subtilis* bacteria was reduced by 11 3,3-bis(hydroxyaryl)oxindoles (**3a–c**, **3f–l**, and **3s**) and seven spiroxanthenes (**4b–d**, **4f**, **4h**, **4k**, and **10**). Most of these derivatives showed activity in the range of 1–5 μM after 6 h. Among the 3,3-bis(hydroxyaryl)oxindoles, those compounds that contain a 4-Cl (**3b**), 5-Cl (**3c**), 5-Br (**3f**), 5-I (**3g**), or 7-CF_3_ (**3i**) substituent on the isatin ring showed significant activity after both 6 and 20 h. The 5-I-substituted derivative (**3g**) proved to be the most effective, as a 2.01 ± 0.42 μM IC_50_ value was obtained after 20 h. The spirooxindoles (**4b–d**, **4f**, **4h**, **4k**, and **10**) also showed great activity after 6 h (0.75 ± 0.42 to 4.61 ± 0.25 μM); however, they were inactive after 20 h. In this case, the 5-Cl-isatin derivative (**4c**) proved to be the most active compound with an IC_50_ value quite close to that of the reference gentamicin. Based on the biological results, both families of compounds were found to be biologically active. The most promising effect was shown by compounds **3g** and **4c** against *B. subtilis* bacteria after 6 h. The former (**3g**) showed good activity, even after 20 h.

## Conclusions

A simple and efficient approach was developed for the synthesis of biologically active 3,3-bis(hydroxyaryl)oxindoles and spirooxindoles containing a xanthene moiety by the direct reaction of isatins and phenols in the presence of methanesulfonic acid under mild, solvent-free, MW-assisted conditions and short reactions times (30–90 min). Our presented method is faster, more atom efficient, and more environmentally friendly than most approaches described in the literature. Altogether, 32 derivatives were prepared, in high yields (18–95%, with a median yield of 84%); among them, 17 are new compounds. The mechanism of the ring-closing step (transforming **3** into **4**) was also investigated by quantum chemical calculation, a step that is crucial for the formation of the xanthene products (**4**). Moreover, the *in vitro* cytotoxicity and antibacterial activity of the compounds synthesized were also studied. Both the 3,3-bis(hydroxyaryl)oxindoles and spiroxanthenes were found to be active in the tested biological indications. Among them, 14 compounds showed cytotoxic activity on six cancer cell lines with a best overall IC_50_ value of 11.76 ± 3.75 μM (**4d**) and 18 derivatives showed significant antibacterial activity against Gram-positive *B. subtilis* bacteria with a best overall IC_50_ value of 0.75 ± 0.42 μM (**4c**) after 6 h. Furthermore, the 5-I-substituted 3,3-bis(hydroxyaryl)oxindole (**3g**) showed good activity even after 20 h (IC_50_ = 2.01 ± 0.42 μM).

## Experimental Section

### General Information

Starting materials were obtained from commercial suppliers and used without further purification. Thin-layer chromatography (TLC) was performed on Merck DC precoated TLC plates with 0.25 mm Kieselgel 60 F254 with a 254 nm UV lamp for visualization. Purification was performed with a Teledyne ISCO Combiflash Nextgen 300+ flash chromatography system using Teledyne ISCO RediSep Rf silica flash chromatography columns with *n*-hexane and ethyl acetate gradients. Reactions were carried out using an 850 W Anton Paar Monowave 400 microwave reactor equipped with an external IR sensor, in closed microwave reaction vials using a 30 s ramp up time to set the temperature with dynamic power usage (5–15 W). The hydrogenation reaction was conducted in an H-Cube Pro^Ⓡ^ continuous flow hydrogenating system (with a total volume of 3.5 mL), supported by a Knauer Azura P 2.1S HPLC pump.

High-performance liquid chromatography-mass spectrometry (HPLC-MS) measurements were performed with an Agilent 1200 liquid chromatography system coupled with a 6130 quadrupole mass spectrometer equipped with an ESI ion source (Agilent Technologies, Palo Alto, CA). Analysis was performed at 40 °C on a Gemini C18 column (150 mm × 4.6 mm, 3 μm; Phenomenex, Torrance, CA) with a mobile phase flow rate of 0.6 mL/min. Eluent A consisted of 0.1% (NH_4_)(HCOO) in water; eluent B consisted of 0.1% (NH_4_)(HCOO) and 8% water in acetonitrile (from 0 to 3 min, 5% B; gradient from 3 to 13 min; from 13 to 20 min, 100% B). The injection volume was 2 μL. The chromatographic profile was registered at 256 nm. The MSD operating parameters were as follows: positive ionization mode, scan spectra from *m*/*z* 120 to 1000, drying gas temperature of 300 °C, nitrogen flow rate of 12 L/min, nebulizer pressure of 60 psi, and capillary voltage of 4000 V.

The ^1^H, ^13^C{^1^H}, and ^19^F{^1^H} NMR spectra were recorded in a DMSO-*d*_6_ solution on a Bruker AV-300 or DRX-500 spectrometer operating at 300, 75.5, and 282 or 500, 125.7, and 470 MHz, respectively. Chemical shifts are expressed in parts per million (δ) using the residual solvent peaks of DMSO-*d*_6_ as an internal standard (^1^H δ 2.50 and ^13^C δ 39.52); coupling constants (*J*) are expressed in hertz, and multiplicities are denoted as s (singlet), d (doublet), t (triplet), q (quartet), m (multiplet), dd (doublet of doublet), td (triplet of doublet), and ddd (doublet of doublets of doublets). Structural assignments were made with additional information from gCOSY, gHSQC, and gHMBC experiments.

Melting points were measured on a SETARAM DSC92-type differential scanning calorimeter under a 1.6 bar nitrogen atmosphere in uncovered aluminum pans with a heating rate of 10 °C/min and a temperature range of 25–360 °C.

High-resolution measurements were performed on a Sciex TripleTOF 5600+ high-resolution tandem mass spectrometer equipped with a DuoSpray ion source. Electrospray ionization was applied in the positive ion detection mode. Samples were dissolved in acetonitrile and flow injected into a 50:50 acetonitrile/water flow. The flow rate was 0.2 mL/min. The resolution of the mass spectrometer was 35000.

### Synthesis of Compounds **3a–s**

*Method A*. To a 10 mL reaction vial suitable for microwave reaction, equipped with a magnetic stirrer, were added 1.00 mmol of the isatin derivative (**1a**–**m**), 3.00 mmol (3.00 equiv) of the phenol derivative (**2a**–**i**), and 39 μL (0.60 mmol, 0.60 equiv) of MeSO_3_H. The vial was sealed, and the mixture was heated to 60 °C in 30 s and held at that temperature for 30 min. Afterward, the reaction mixture was analyzed via TLC and HPLC-MS measurements. The mixture was dissolved in EtOAc and washed with water, and then the aqueous phase was extracted twice with EtOAc. The combined organic phase was dried over anhydrous Na_2_SO_4_, filtered and evaporated onto Celite, and purified by flash chromatography using *n*-hexane and EtOAc gradients. The purified products were dried with DCM and heated at 110–120 °C for 1–3 h to dry from any solvents.

*Method B*. Identical to method A except that the reaction was carried out at 80 °C.

*Method C*. Identical to method A except that an increased amount of MeSO_3_H (1.00 mmol) was used and the reaction was carried out at 90 °C, with a reaction time of 90 min.

#### 3,3-Bis(2-hydroxy-5-methylphenyl)indolin-2-one (**3a**)

Synthesized according to method A: yield 95% (328.1 mg); white solid; an eluent gradient of *n*-hexane and EtOAc (0% → 35%) was used during flash chromatography; mp 323.9 °C; ^1^H and ^13^C{^1^H} NMR data are in accordance with data previously disclosed in the literature (see ref (15)); HRMS (ESI) *m*/*z* [M + H]^+^ calcd for C_22_H_20_NO_3_ 346.1443, found 346.1449.

#### 4-Chloro-3,3-bis(2-hydroxy-5-methylphenyl)indolin-2-one (**3b**)

Synthesized according to methods A and B: yield 58% (220.3 mg) with method A and 83% (315.3 mg) with method B; white solid; an eluent gradient of *n*-hexane and EtOAc (0% → 30%) was used during flash chromatography; mp 221.9 °C; ^1^H NMR (500 MHz, DMSO-*d*_6_) δ 11.19 (s, 1H), 9.48 (s, 1H), 9.35 (s, 1H), 7.31 (t, *J* = 8.0 Hz, 1H), 7.08 (dd, *J* = 8.4, 2.2 Hz, 1H), 7.01 (dd, *J* = 8.2, 1.0 Hz, 1H), 6.94 (dd, *J* = 7.7, 1.0 Hz, 1H), 6.90 (dd, *J* = 8.2, 2.2 Hz, 1H), 6.85 (d, *J* = 8.1 Hz, 1H), 6.69 (d, *J* = 2.2 Hz, 1H), 6.57 (d, *J* = 8.1 Hz, 1H), 6.51 (d, *J* = 2.2 Hz, 1H), 2.11 (s, 3H), 2.07 (s, 3H); ^13^C{^1^H} NMR (125.7 MHz, DMSO-*d*_6_) δ 182.8, 155.0, 152.4, 144.1, 132.1, 131.1, 130.4, 130.2, 129.3, 129.2, 128.3, 128.1, 125.9, 123.3, 120.9, 120.3, 119.2, 115.1, 109.0, 61.1, 20.5, 20.4; HRMS (ESI) *m*/*z* [M + H]^+^ calcd for C_22_H_19_ClNO_3_ 380.1047, found 380.1059.

#### 5-Chloro-3,3-bis(2-hydroxy-5-methylphenyl)indolin-2-one (**3c**)

Synthesized according to methods A and B: yield 75% (284.9 mg) with method A and 91% (345.7 mg) with method B; off-white solid; an eluent gradient of *n*-hexane and EtOAc (0% → 40%) was used during flash chromatography; mp 287.9 °C; ^1^H NMR (500 MHz, DMSO-*d*_6_) δ 11.02 (s, 1H), 9.46 (s, 2H), 7.28 (dd, *J* = 8.3, 2.2 Hz, 1H), 7.13–6.96 (m, 4H), 6.75 (d, *J* = 8.1 Hz, 1H), 6.64 (d, *J* = 8.1 Hz, 1H), 6.61 (s, 1H), 6.57 (s, 1H), 2.10 (s, 6H); ^13^C{^1^H} NMR (125.7 MHz, DMSO-*d*_6_) δ 182.4, 154.3, 152.6, 140.9, 134.8, 129.9, 129.5, 129.2, 128.9, 127.8, 127.2, 125.7, 124.3, 123.4, 118.3, 115.6, 111.2, 60.3, 20.4; HRMS (ESI) *m*/*z* [M + H]^+^ calcd for C_22_H_19_ClNO_3_ 380.1047, found 380.1062.

#### 6-Chloro-3,3-bis(2-hydroxy-5-methylphenyl)indolin-2-one (**3d**)

Synthesized according to methods A and B: yield 73% (277.3 mg) with method A and 90% (342.9 mg) with method B; white solid; an eluent gradient of *n*-hexane and EtOAc (0% → 35%) was used during flash chromatography; mp 219.2 °C; ^1^H NMR (500 MHz, DMSO-*d*_6_) δ 11.07 (s, 1H), 9.50 (s, 1H), 9.41 (s, 1H), 7.04 (d, *J* = 2.0 Hz, 1H), 7.03 (d, *J* = 2.0 Hz, 1H), 6.95 (d, *J* = 2.0 Hz, 1H), 6.91 (d, *J* = 8.0 Hz, 2H), 6.78 (d, *J* = 8.0 Hz, 1H), 6.60 (d, *J* = 7.9 Hz, 1H), 6.55 (s, 2H), 2.11 (s, 3H), 2.08 (s, 3H); ^13^C{^1^H} NMR (125.7 MHz, DMSO-*d*_6_) δ 183.2, 143.4, 132.2, 131.2, 130.1, 129.3, 128.8, 128.1, 127.5, 121.5, 118.7, 109.9, 59.7, 20.4; HRMS (ESI) *m*/*z* [M + H]^+^ calcd for C_22_H_19_ClNO_3_ 380.1047, found 380.1058.

#### 5-Fluoro-3,3-bis(2-hydroxy-5-methylphenyl)indolin-2-one (**3e**)

Synthesized according to method A: yield 92% (334.3 mg); white solid; an eluent gradient of *n*-hexane and EtOAc (0% → 35%) was used during flash chromatography; mp 263.7 °C; ^1^H NMR (500 MHz, DMSO-*d*_6_) δ 10.93 (s, 1H), 9.58 (s, 1H), 9.40 (s, 1H), 7.11–6.99 (m, 2H), 6.93 (d, *J* = 4.4 Hz, 1H), 6.91 (d, *J* = 4.5 Hz, 1H), 6.80 (dd, *J* = 8.5, 2.7 Hz, 1H), 6.76 (d, *J* = 7.8 Hz, 1H), 6.62 (d, *J* = 8.0 Hz, 1H), 6.58 (d, *J* = 2.1 Hz, 2H), 2.11 (s, 3H), 2.10 (s, 3H); ^13^C{^1^H} NMR (125.7 MHz, DMSO-*d*_6_) δ 183.0, 159.0, 157.1, 153.6 (d, *J*_C–F_ = 243.4 Hz), 138.2, 134.3 (d, *J*_C–F_ = 8.0 Hz), 130.0, 129.4, 129.2, 129.0, 128.0, 127.2, 124.5, 123.5, 118.5, 115.6, 114.4 (d, *J*_C–F_ = 23.2 Hz), 113.6 (d, *J*_C–F_ = 24.7 Hz), 110.6 (d, *J*_C–F_ = 8.3 Hz), 60.5, 20.5; ^19^F{^1^H} NMR (282 MHz, DMSO-*d*_6_) δ −121.4; HRMS (ESI) *m*/*z* [M + H]^+^ calcd for C_22_H_19_FNO_3_ 364.1348, found 364.1357.

#### 5-Bromo-3,3-bis(2-hydroxy-5-methylphenyl)indolin-2-one (**3f**)

Synthesized according to method A: yield 94% (398.8 mg); white solid; an eluent gradient of *n*-hexane and EtOAc (0% → 30%) was used during flash chromatography; mp 307.0 °C; ^1^H NMR (500 MHz, DMSO-*d*_6_) δ 11.02 (s, 1H), 9.45 (s, 1H), 9.44 (s, 1H), 7.40 (dd, *J* = 8.3, 2.1 Hz, 1H), 7.13 (d, *J* = 2.1 Hz, 1H), 7.02 (d, *J* = 8.1 Hz, 1H), 6.93 (d, *J* = 8.0 Hz, 1H), 6.89 (d, *J* = 8.2 Hz, 1H), 6.74 (d, *J* = 8.1 Hz, 1H), 6.64 (d, *J* = 8.2 Hz, 1H), 6.61 (s, 1H), 6.56 (s, 1H), 2.10 (s, 6H); ^13^C{^1^H} NMR (125.7 MHz, DMSO-*d*_6_) δ 182.2, 154.2, 152.6, 141.3, 135.2, 130.6, 129.9, 129.5, 129.2, 128.9, 128.3, 127.8, 127.2, 124.3, 123.4, 118.2, 115.7, 113.3, 111.7, 60.2, 20.5; HRMS (ESI) *m*/*z* [M + H]^+^ calcd for C_22_H_19_BrNO_3_ 424.0548, found 424.0558.

#### 3,3-Bis(2-hydroxy-5-methylphenyl)-5-iodoindolin-2-one (**3g**)

Synthesized according to methods A and C: yield 39% (183.8 mg) with method A and 79% (372.3 mg) with method C; white solid; an eluent gradient of *n*-hexane and EtOAc (0% → 25%) was used during flash chromatography; ^1^H NMR (500 MHz, DMSO-*d*_6_) δ 11.00 (s, 1H), 9.44 (s, 2H), 7.56 (dd, *J* = 8.2, 1.8 Hz, 1H), 7.25 (d, *J* = 1.9 Hz, 1H), 7.02 (d, *J* = 8.4 Hz, 1H), 6.92 (d, *J* = 8.6 Hz, 1H), 6.78 (d, *J* = 8.1 Hz, 1H), 6.75 (d, *J* = 8.1 Hz, 1H), 6.64 (d, *J* = 8.1 Hz, 1H), 6.59 (s, 1H), 6.55 (s, 1H), 2.10 (s, 6H); ^13^C{^1^H} NMR (125.7 MHz, DMSO-*d*_6_) δ 182.1, 154.3, 152.6, 141.8, 136.5, 135.3, 133.8, 129.9, 129.5, 129.2, 128.9, 127.8, 127.2, 124.3, 123.4, 118.3, 115.7, 112.3, 84.5, 60.0, 20.5; HRMS (ESI) *m*/*z* [M + H]^+^ calcd for C_22_H_19_INO_3_ 472.0404, found 472.0442.

#### 3,3-Bis(2-hydroxy-5-methylphenyl)-5-nitroindolin-2-one (**3h**)

Synthesized according to method A: yield 88% (343.5 mg); yellow solid; an eluent gradient of *n*-hexane and EtOAc (0% → 35%) was used during flash chromatography; mp 279.8 °C; ^1^H NMR (500 MHz, DMSO-*d*_6_) δ 11.45 (s, 1H), 9.69 (s, 1H), 9.14 (s, 1H), 8.19–8.10 (m, 2H), 7.06 (d, *J* = 8.5 Hz, 1H), 6.97 (dd, *J* = 8.1, 2.1 Hz, 2H), 6.82 (s, 1H), 6.73 (d, *J* = 8.0 Hz, 1H), 6.67 (d, *J* = 7.9 Hz, 1H), 6.62 (s, 1H), 2.14 (s, 3H), 2.11 (s, 3H); ^13^C{^1^H} NMR (125.7 MHz, DMSO-*d*_6_) δ 181.7, 153.3, 148.6, 142.0, 135.0, 130.4, 129.6, 128.9, 127.5, 127.4, 124.9, 123.2, 120.9, 117.0, 116.1, 109.4, 60.0, 20.4; HRMS (ESI) *m*/*z* [M + H]^+^ calcd for C_22_H_19_N_2_O_5_ 391.1288, found 391.1307.

#### 3,3-Bis(2-hydroxy-5-methylphenyl)-7-(trifluoromethyl)indolin-2-one (**3i**)

Synthesized according to methods A and B: yield 84% (347.3 mg) with method A and 93% (384.5 mg) with method B; white solid; an eluent gradient of *n*-hexane and EtOAc (0% → 20%) was used during flash chromatography; mp 277.1 °C; ^1^H NMR (500 MHz, DMSO-*d*_6_) δ 11.37 (s, 1H), 9.54 (s, 1H), 9.43 (s, 1H), 7.54 (dd, *J* = 7.9, 1.2 Hz, 1H), 7.22 (d, *J* = 7.4 Hz, 1H), 7.16 (t, *J* = 7.7 Hz, 1H), 7.05 (d, *J* = 7.9 Hz, 1H), 6.92 (dd, *J* = 8.2, 2.2 Hz, 1H), 6.79 (d, *J* = 8.1 Hz, 1H), 6.63–6.59 (m, 2H), 6.50 (d, *J* = 2.1 Hz, 1H), 2.10 (s, 6H); ^13^C{^1^H} NMR (125.7 MHz, DMSO-*d*_6_) δ 183.6, 170.3, 154.5, 152.5, 139.3, 134.3, 130.2, 130.0, 129.4 (d, *J*_C–F_ = 5.0 Hz), 128.9, 128.2, 127.2, 124.5 (q, *J*_C–F_ = 4.2 Hz), 124.1, 123.7 (d, *J*_C–F_ = 272.0 Hz), 123.0, 121.9, 118.6, 115.5, 111.0 (q, *J*_C–F_ = 32.7 Hz), 59.1, 20.5, 20.4; ^19^F{^1^H} NMR (282 MHz, DMSO-*d*_6_) δ −59.7; HRMS (ESI) *m*/*z* [M + H]^+^ calcd for C_23_H_19_F_3_NO_3_ 414.1317, found 414.1328.

#### 3,3-Bis(2-hydroxy-5-methylphenyl)-5-methoxyindolin-2-one (**3j**)

Synthesized according to methods A and C: yield 16% (60.1 mg) with method A and 60% (225.3 mg) with method C; off-white solid; an eluent gradient of *n*-hexane and EtOAc (0% → 30%) was used during flash chromatography; mp 238.6 °C; ^1^H NMR (500 MHz, DMSO-*d*_6_) δ 10.80 (s, 1H), 9.82 (s, 1H), 9.30 (s, 1H), 7.03 (d, *J* = 7.7 Hz, 1H), 6.93–6.80 (m, 3H), 6.77 (d, *J* = 8.1 Hz, 1H), 6.65–6.55 (m, 2H), 6.52 (s, 1H), 6.41 (d, *J* = 2.5 Hz, 1H), 3.66 (s, 3H), 2.11 (s, 3H), 2.08 (s, 3H); ^13^C{^1^H} NMR (125.7 MHz, DMSO-*d*_6_) δ 183.1, 154.9, 152.4, 135.3, 133.5, 130.0, 129.3, 128.9, 126.9, 125.1, 123.8, 118.8, 115.4, 113.4, 112.3, 110.3, 60.5, 55.5, 20.5; HRMS (ESI) *m*/*z* [M + H]^+^ calcd for C_23_H_22_NO_4_ 376.1543, found 376.1554.

#### 3,3-Bis(2-hydroxy-5-methylphenyl)-5-methylindolin-2-one (**3k**)

Synthesized according to methods A and C: yield 32% (115.0 mg) with method A and 84% (301.9 mg) with method C; off-white solid; an eluent gradient of *n*-hexane and EtOAc (0% → 25%) was used during flash chromatography; mp 271.4 °C; ^1^H NMR (500 MHz, DMSO-*d*_6_) δ 10.85 (s, 1H), 9.82 (s, 1H), 9.28 (s, 1H), 7.08–7.01 (m, 2H), 6.88 (d, *J* = 8.1 Hz, 1H), 6.84 (d, *J* = 7.9 Hz, 1H), 6.78 (d, *J* = 8.1 Hz, 1H), 6.65 (s, 1H), 6.62–6.55 (m, 2H), 6.51 (s, 1H), 2.23 (s, 3H), 2.10 (s, 3H), 2.07 (s, 3H); ^13^C{^1^H} NMR (125.7 MHz, DMSO-*d*_6_) δ 183.4, 154.9, 152.3, 139.4, 132.0, 130.7, 130.0, 129.3, 128.8, 128.4, 128.0, 126.9, 126.6, 125.2, 123.9, 118.9, 115.4, 109.8, 60.1, 20.7, 20.5; HRMS (ESI) *m*/*z* [M + H]^+^ calcd for C_23_H_22_NO_3_ 360.1594, found 360.1608.

#### 3,3-Bis(2-hydroxy-5-methylphenyl)-1-methylindolin-2-one (**3l**)

Synthesized according to method A: yield 89% (319.9 mg); white solid; an eluent gradient of *n*-hexane and EtOAc (0% → 25%) was used during flash chromatography; mp 229.2 °C; ^1^H NMR (500 MHz, DMSO-*d*_6_) δ 9.72 (s, 1H), 9.33 (s, 1H), 7.35 (td, *J* = 7.7, 1.2 Hz, 1H), 7.15 (d, *J* = 7.8 Hz, 1H), 7.09 (td, *J* = 7.6, 1.1 Hz, 1H), 7.05 (d, *J* = 7.9 Hz, 1H), 6.92 (dd, *J* = 7.5, 1.2 Hz, 1H), 6.88 (d, *J* = 8.0 Hz, 1H), 6.80 (d, *J* = 8.1 Hz, 1H), 6.68–6.48 (m, 3H), 3.21 (s, 3H), 2.08 (s, 6H); ^13^C{^1^H} NMR (125,7 MHz, DMSO-*d*_6_) δ 181.5, 154.8, 152.1, 143.4, 131.0, 130.2, 129.3, 129.0, 128.7, 128.1, 127.0, 126.0, 125.1, 123.5, 122.6, 118.9, 115.5, 109.1, 59.4, 26.7, 20.4; HRMS (ESI) *m*/*z* [M + H]^+^ calcd for C_23_H_22_NO_3_ 360.1594, found 360.1604.

#### 3,3-Bis(2-hydroxyphenyl)indolin-2-one (**3n-I**)

Synthesized according to method A: yield 6% (19.0 mg); off-white solid; an eluent gradient of *n*-hexane and EtOAc (0% → 40%) was used during flash chromatography; mp 244.5 °C; ^1^H NMR (500 MHz, DMSO-*d*_6_) δ 10.28 (s, 1H), 9.40 (s, 2H), 7.17 (ddd, *J* = 7.7, 6.5, 2.4 Hz, 1H), 7.11 (d, *J* = 8.4 Hz, 2H), 7.06 (ddd, *J* = 7.9, 6.6, 2.4 Hz, 1H), 6.98–6.88 (m, 2H), 6.85 (d, *J* = 7.7 Hz, 1H), 6.75–6.66 (m, 4H), 6.65 (dd, *J* = 8.0, 1.1 Hz, 1H); ^13^C{^1^H} NMR (125.7 MHz, DMSO-*d*_6_) δ 179.9, 156.5, 155.1, 142.5, 133.1, 129.8, 129.6, 129.0, 128.0, 127.5, 125.3, 120.8, 118.4, 115.4, 114.8, 109.1, 58.8; HRMS (ESI) *m*/*z* [M + H]^+^ calcd for C_20_H_16_NO_3_ 318.1124, found 318.1131.

#### 3,3-Bis(4-hydroxyphenyl)indolin-2-one (**3n-II**)

Synthesized according to method A: yield 91% (288.8 mg); white solid; an eluent gradient of *n*-hexane and EtOAc (0% → 45%) was used during flash chromatography; mp 196.9 °C; ^1^H and ^13^C{^1^H} NMR data are in accordance with data previously disclosed in the literature (see ref (14)); HRMS (ESI) *m*/*z* [M + H]^+^ calcd for C_20_H_16_NO_3_ 318.1124, found 318.1135.

#### Isomer I Derived from Isatin and *m*-Cresol (**3o-I**)

Synthesized according to method A: yield 32% (110.5 mg); white solid; an eluent gradient of *n*-hexane and EtOAc (0% → 25%) was used during flash chromatography; mp 245.1 °C; ^1^H NMR (500 MHz, DMSO-*d*_6_) δ 10.96 (s, 1H), 9.91 (s, 1H), 9.45 (s, 1H), 7.24 (td, *J* = 7.6, 1.3 Hz, 1H), 7.00 (td, *J* = 7.6, 1.1 Hz, 1H), 6.95 (dd, *J* = 7.8, 3.5 Hz, 1H), 6.80 (d, *J* = 7.4 Hz, 1H), 6.74–6.38 (m, 6H), 2.23 (s, 3H), 2.17 (s, 3H); ^13^C{^1^H} NMR (125,7 MHz, DMSO-*d*_6_) δ 184.2, 157.5, 154.9, 142.3, 139.7, 138.4, 132.6, 129.3, 128.9, 128.5, 126.5, 123.0, 122.3, 121.5, 120.9, 120.0, 116.6, 110.5, 60.1, 21.0; HRMS (ESI) *m*/*z* [M + H]^+^ calcd for C_22_H_20_NO_3_ 346.1443, found 346.1444.

#### Isomer II Derived from Isatin and *m*-Cresol (**3o-II**)

Synthesized according to method A: yield 28% (96.7 mg); white solid; an eluent gradient of *n*-hexane and EtOAc (0% → 35%) was used during flash chromatography; mp 274.1 °C; ^1^H NMR (500 MHz, DMSO-*d*_6_) δ 10.41 (s, 1H), 9.28 (s, 1H), 9.23 (s, 1H), 7.21 (td, *J* = 7.7, 1.2 Hz, 1H), 6.95 (td, *J* = 7.6, 1.1 Hz, 1H), 6.90 (d, *J* = 7.6 Hz, 1H), 6.82 (d, *J* = 7.5 Hz, 1H), 6.68 (d, *J* = 8.5 Hz, 1H), 6.59 (d, *J* = 2.6 Hz, 1H), 6.53–6.46 (m, 3H), 6.42 (dd, *J* = 8.6, 2.7 Hz, 1H), 2.03 (s, 3H), 1.93 (s, 3H); ^13^C{^1^H} NMR (125,7 MHz, DMSO-*d*_6_) δ 179.8, 156.2, 155.9, 140.9, 134.3, 130.4, 130.2, 128.9, 128.6, 127.9, 125.8, 121.9, 119.4, 118.7, 112.6, 112.3, 109.4, 61.9, 22.6, 20.6; HRMS (ESI) *m*/*z* [M + H]^+^ calcd for C_22_H_20_NO_3_ 346.1443, found 346.1447.

#### 3,3-Bis(4-hydroxy-3-methylphenyl)indolin-2-one (**3p**)

Synthesized according to method A: yield 85% (284.6 mg); white solid; an eluent gradient of *n*-hexane and EtOAc (0% → 35%) was used during flash chromatography; mp 255.5 °C; ^1^H and ^13^C{^1^H} NMR data are in accordance with data previously disclosed in the literature (see ref (14)); HRMS (ESI) *m*/*z* [M + H]^+^ calcd for C_22_H_20_NO_3_ 346.1443, found 346.1450.

#### 3,3-Bis(5-fluoro-2-hydroxyphenyl)indolin-2-one (**3s**)

Synthesized according to methods A and C: yield 11% (38.9 mg) with method A and 49% (173.1 mg) with method C; off-white solid; an eluent gradient of *n*-hexane and EtOAc (0% → 30%) was used during flash chromatography; mp 252.8 °C; ^1^H NMR (500 MHz, DMSO-*d*_6_) δ 11.13 (s, 1H), 9.91 (s, 1H), 9.73 (s, 1H), 7.29 (ddd, *J* = 7.7, 6.8, 2.1 Hz, 1H), 7.11 (s, 1H), 7.08–6.95 (m, 4H), 6.92 (s, 1H), 6.71 (s, 1H), 6.57–6.45 (m, 2H); ^13^C{^1^H} NMR (125.7 MHz, DMSO-*d*_6_) δ 182.3, 152.4 (d, *J*_C–F_ = 254.2 Hz), 142.0, 131.1, 129.0, 126.2, 125.4 (d, *J*_C–F_ = 2.4 Hz), 122.7, 120.3 (d, *J*_C–F_ = 11.4 Hz), 116.7 (d, *J*_C–F_ = 7.3 Hz), 115.8 (d, *J*_C–F_ = 24.7 Hz), 115.3 (d, *J*_C–F_ = 23.2 Hz), 110.7, 60.0; ^19^F{^1^H} NMR (282 MHz, DMSO-*d*_6_) δ −125.6, −124.0; HRMS (ESI) *m*/*z* [M + H]^+^ calcd for C_20_H_14_F_2_NO_3_ 354.0936, found 354.0934.

### Synthesis of Compounds **4a–s**

*Method A*. To a 10 mL reaction vial suitable for microwave reaction, equipped with a magnetic stirrer, were added 1.00 mmol of the isatin derivative (**1a**–**m**), 3.00 mmol (3.00 equiv) of the phenol derivative (**2a**–**i**), and 97 μL (1.50 mmol, 1.50 equiv) of MeSO_3_H (in the case of **4q**, 0.6 mL of EtOAc and 0.6 mL/mmol isatin were also added). The vial was sealed, and the mixture was heated to 150 °C in 30 s and held at that temperature for 60 min. Afterward, the reaction mixture was analyzed by TLC and HPLC-MS measurements. The mixture was transferred onto Celite and purified by flash chromatography using *n*-hexane and EtOAc gradients. The purified products were dried with DCM and heated at 110–120 °C for 1–3 h to dry from any solvent.

*Method B*. Identical to method A except that the reaction was carried out at 135 °C, with a reaction time of 80 min.

*Method C*. Identical to method A except that an increased amount of *p*-cresol (4.00 mmol) was used and the reaction was carried out at 135 °C, with a reaction time of 80 min.

*Method D*. Identical to method A except that a reduced amount of MeSO_3_H (0.60 mmol) was used, 0.6 mL of EtOAc was added, and the reaction was carried out at 90 °C, with a reaction time of 45 min.

#### 2′,7′-Dimethylspiro[indoline-3,9′-xanthen]-2-one (**4a**)

Synthesized according to method A: yield 88% (288.1 mg); white solid; an eluent gradient of *n*-hexane and EtOAc (0% → 20%) was used during flash chromatography; mp 333.3 °C; ^1^H and ^13^C{^1^H} NMR data are in accordance with data previously disclosed in the literature (see ref (15)); HRMS (ESI) *m*/*z* [M + H]^+^ calcd for C_22_H_18_NO_2_ 328.1337, found 328.1339.

#### 4-Chloro-2′,7′-dimethylspiro[indoline-3,9′-xanthen]-2-one (**4b**)

Synthesized according to method A: yield 89% (322.0 mg); white solid; an eluent gradient of *n*-hexane and EtOAc (0% → 20%) was used during flash chromatography; mp 299.3 °C; ^1^H and ^13^C{^1^H} NMR data are in accordance with data previously disclosed in the literature (see ref (16)); HRMS (ESI) *m*/*z* [M + H]^+^ calcd for C_22_H_17_ClNO_2_ 362.0942, found 362.0945.

#### 5-Chloro-2′,7′-dimethylspiro[indoline-3,9′-xanthen]-2-one (**4c**)

Synthesized according to method A: yield 91% (329.2 mg); white solid; an eluent gradient of *n*-hexane and EtOAc (0% → 25%) was used during flash chromatography; mp 306.8 °C; ^1^H and ^13^C{^1^H} NMR data are in accordance with data previously disclosed in the literature (see ref (16)); HRMS (ESI) *m*/*z* [M + H]^+^ calcd for C_22_H_17_ClNO_2_ 362.0942, found 362.0942.

#### 6-Chloro-2′,7′-dimethylspiro[indoline-3,9′-xanthen]-2-one (**4d**)

Synthesized according to method A: yield 80% (289.5 mg); white solid; an eluent gradient of *n*-hexane and EtOAc (0% → 15%) was used during flash chromatography; mp 279.7 °C; ^1^H NMR (500 MHz, DMSO-*d*_6_) δ 10.79 (s, 1H), 7.12 (d, *J* = 1.2 Hz, 4H), 7.07 (d, *J* = 2.0 Hz, 1H), 7.04 (dd, *J* = 7.9, 2.0 Hz, 1H), 6.95 (d, *J* = 8.0 Hz, 1H), 6.40 (s, 2H), 2.14 (s, 6H); ^13^C{^1^H} NMR (125.7 MHz, DMSO-*d*_6_) δ 178.6, 148.5, 143.9, 134.9, 133.2, 132.6, 130.0, 126.9, 126.5, 122.7, 120.0, 116.6, 110.2, 52.2, 20.1; HRMS (ESI) *m*/*z* [M + H]^+^ calcd for C_22_H_17_ClNO_2_ 362.0942, found 362.0941.

#### 5-Fluoro-2′,7′-dimethylspiro[indoline-3,9′-xanthen]-2-one (**4e**)

Synthesized according to method A: yield 88% (303.9 mg); white solid; an eluent gradient of *n*-hexane and EtOAc (0% → 15%) was used during flash chromatography; mp 314.5 °C; ^1^H NMR data are in accordance with data previously disclosed in the literature (see ref (16)); ^13^C{^1^H} NMR (126 MHz, DMSO-*d*_6_) δ 178.7, 158.6 (d, *J*_C–F_ = 238.4 Hz), 148.5, 138.6 (d, *J*_C–F_ = 2.0 Hz), 137.4 (d, *J*_C–F_ = 7.7 Hz), 132.6, 130.0, 126.8, 120.0, 116.6, 115.6 (d, *J*_C–F_ = 23.3 Hz), 112.7 (d, *J*_C–F_ = 24.5 Hz), 111.1 (d, *J*_C–F_ = 8.1 Hz), 53.0, 20.2; ^19^F{^1^H} NMR (282 MHz, DMSO-*d*_6_) δ −120.1; HRMS (ESI) *m*/*z* [M + H]^+^ calcd for C_22_H_17_FNO_2_ 346.1243, found 346.1250.

#### 5-Bromo-2′,7′-dimethylspiro[indoline-3,9′-xanthen]-2-one (**4f**)

Synthesized according to method A: yield 82% (333.1 mg); white solid; an eluent gradient of *n*-hexane and EtOAc (0% → 20%) was used during flash chromatography; mp 335.5 °C; ^1^H and ^13^C{^1^H} NMR data are in accordance with data previously disclosed in the literature (see ref (16)); HRMS (ESI) *m*/*z* [M + H]^+^ calcd for C_22_H_17_BrNO_2_ 406.0442, found 406.0449.

#### 2′,7′-Dimethyl-5-nitrospiro[indoline-3,9′-xanthen]-2-one (**4h**)

Synthesized according to methods A and B: yield 50% (186.2 mg) with method A and 70% (260.7 mg) with method B; white solid; an eluent gradient of *n*-hexane and EtOAc (0% → 30%) was used during flash chromatography; mp 327.1 °C; ^1^H and ^13^C{^1^H} NMR data are in accordance with data previously disclosed in the literature (see ref (16)); HRMS (ESI) *m*/*z* [M + H]^+^ calcd for C_22_H_17_N_2_O_4_ 373.1182, found 373.1189.

#### 2′,7′-Dimethyl-7-(trifluoromethyl)spiro[indoline-3,9′-xanthen]-2-one (**4i**)

Synthesized according to methods A and C: yield 19% (75.1 mg) with method A and 39% (154.2 mg) with method C; white solid; an eluent gradient of *n*-hexane and EtOAc (0% → 10%) was used during flash chromatography; mp 260.2 °C; ^1^H NMR (500 MHz, DMSO-*d*_6_) δ 11.12 (s, 1H), 7.63 (dd, *J* = 7.9, 1.3 Hz, 1H), 7.24 (d, *J* = 7.3 Hz, 1H), 7.18 (t, *J* = 7.4 Hz, 1H), 7.15 (d, *J* = 0.9 Hz, 4H), 6.37 (s, 2H), 2.13 (s, 6H); ^13^C{^1^H} NMR (125.7 MHz, DMSO-*d*_6_) δ 179.0, 148.5, 139.6 (q, *J*_C–F_ = 2.2 Hz), 137.7, 132.8, 130.2, 129.3, 126.7, 125.7 (q, *J*_C–F_ = 4.4 Hz), 123.5 (d, *J*_C–F_ = 271.9 Hz), 123.2, 122.5, 119.8, 116.8, 111.2 (q, *J*_C–F_ = 33.1 Hz), 51.7, 20.1; ^19^F{^1^H} NMR (282 MHz, DMSO-*d*_6_) δ −59.7; HRMS (ESI) *m*/*z* [M + H]^+^ calcd for C_23_H_17_F_3_NO_2_ 396.1211, found 396.1218.

#### 5-Methoxy-2′,7′-dimethylspiro[indoline-3,9′-xanthen]-2-one (**4j**)

Synthesized according to method A: yield 46% (164.4 mg); white solid; an eluent gradient of *n*-hexane and EtOAc (0% → 25%) was used during flash chromatography; mp 298.1 °C; ^1^H NMR (500 MHz, DMSO-*d*_6_) δ 10.45 (s, 1H), 7.11 (d, *J* = 1.2 Hz, 4H), 6.98 (d, *J* = 8.5 Hz, 1H), 6.90 (dd, *J* = 8.5, 2.6 Hz, 1H), 6.50 (d, *J* = 2.6 Hz, 1H), 6.38 (s, 2H), 3.62 (s, 3H), 2.14 (s, 6H); ^13^C{^1^H} NMR (125.7 MHz, DMSO-*d*_6_) δ 178.6, 155.6, 148.5, 137.2, 135.5, 132.4, 129.8, 126.9, 120.6, 116.5, 113.9, 111.4, 110.6, 55.4, 53.1, 20.2; HRMS (ESI) *m*/*z* [M + H]^+^ calcd for C_23_H_20_NO_3_ 358.1437, found 358.1440.

#### 2′,5,7′-Trimethylspiro[indoline-3,9′-xanthen]-2-one (**4k**)

Synthesized according to method A: yield 83% (296.6 mg); off-white solid; an eluent gradient of *n*-hexane and EtOAc (0% → 20%) was used during flash chromatography; mp 307.3 °C; ^1^H and ^13^C{^1^H} NMR data are in accordance with data previously disclosed in the literature (see ref (16)); HRMS (ESI) *m*/*z* [M + H]^+^ calcd for C_23_H_20_NO_2_ 342.1488, found 342.1494.

#### 1,2′,7′-Trimethylspiro[indoline-3,9′-xanthen]-2-one (**4l**)

Synthesized according to method A: yield 70% (239.0 mg); off-white solid; an eluent gradient of *n*-hexane and EtOAc (0% → 15%) was used during flash chromatography; mp 209.5 °C; ^1^H and ^13^C{^1^H} NMR data are in accordance with data previously disclosed in the literature (see ref (16)); HRMS (ESI) *m*/*z* [M + H]^+^ calcd for C_23_H_20_NO_2_ 342.1488, found 342.1488.

#### 3′,6′-Dimethylspiro[indoline-3,9′-xanthen]-2-one (**4o**)

Synthesized according to method A: yield 79% (258.6 mg); white solid; an eluent gradient of *n*-hexane and EtOAc (0% → 20%) was used during flash chromatography; mp 290.3 °C; ^1^H NMR (500 MHz, DMSO-*d*_6_) δ 10.60 (s, 1H), 7.30 (td, *J* = 7.7, 1.2 Hz, 1H), 7.07–7.02 (m, 3H), 6.98 (t, *J* = 7.5 Hz, 1H), 6.89 (d, *J* = 7.5 Hz, 1H), 6.81 (dd, *J* = 8.0, 1.8 Hz, 2H), 6.47 (d, *J* = 7.8 Hz, 2H), 2.28 (s, 6H); ^13^C{^1^H} NMR (125.7 MHz, DMSO-*d*_6_) δ 178.8, 150.4, 142.4, 138.9, 136.1, 128.9, 126.9, 124.9, 124.5, 122.8, 118.2, 116.9, 109.9, 52.1, 20.6; HRMS (ESI) *m*/*z* [M + H]^+^ calcd for C_22_H_18_NO_2_ 328.1332, found 328.1326.

#### 2′,7′-Dihydroxyspiro[indoline-3,9′-xanthen]-2-one (**4q**)

Synthesized according to method A with 0.6 mL of EtOAc added and method D: yield 63% (208.7 mg) with method A and 85% (281.6 mg) with method D; white solid; an eluent gradient of *n*-hexane and EtOAc (0% → 50%) was used during flash chromatography; mp 329.7 °C; ^1^H and ^13^C{^1^H} NMR data are in accordance with data previously disclosed in the literature (see ref (14)); HRMS (ESI) *m*/*z* [M + H]^+^ calcd for C_20_H_14_NO_4_ 332.0917, found 332.0920.

#### 2′,7′-Difluorospiro[indoline-3,9′-xanthen]-2-one (**4s**)

Synthesized according to method A: yield 18% (60.4 mg); white solid; an eluent gradient of *n*-hexane and EtOAc (0% → 15%) was used during flash chromatography; mp 291.9 °C; ^1^H and ^19^F{^1^H} NMR data are in accordance with data previously disclosed in the literature (see ref (15)); ^13^C{^1^H} NMR (126 MHz, DMSO-*d*_6_) δ 177.5, 157.9 (d, *J*_C–F_ = 240.2 Hz), 147.0 (d, *J*_C–F_ = 2.0 Hz), 142.5, 134.1, 129.7, 125.1, 121.7 (d, *J*_C–F_ = 7.6 Hz), 118.7 (d, *J*_C–F_ = 8.5 Hz), 116.6 (d, *J*_C–F_ = 23.4 Hz), 112.7 (d, *J*_C–F_ = 24.4 Hz), 110.5, 53.2; HRMS (ESI) *m*/*z* [M + H]^+^ calcd for C_20_H_12_F_2_NO_2_ 336.0830, found 336.0832.

#### *p*-Tolyl-2′,7′-dimethyl-2-oxospiro[indoline-3,9′-xanthene]-7-carboxylate (**10**)

A side product of the synthesis of **4i** was obtained when using method C: yield 5% (22.0 mg); yellow solid; an eluent gradient of *n*-hexane and EtOAc (0% → 10%) was used during flash chromatography; mp 237.0 °C; ^1^H NMR (500 MHz, DMSO-*d*_6_) δ 10.57 (s, 1H), 8.04 (dd, *J* = 8.0, 1.3 Hz, 1H), 7.33–7.25 (m, 3H), 7.25–7.21 (m, 2H), 7.21–7.17 (m, 1H), 7.16 (d, *J* = 1.2 Hz, 4H), 6.45 (s, 2H), 2.36 (s, 3H), 2.15 (s, 6H); ^13^C{^1^H} NMR (125.7 MHz, DMSO-*d*_6_) δ 178.8, 163.8, 148.5, 148.2, 144.1, 137.8, 135.2, 132.7, 130.4, 130.2, 130.1, 129.8, 126.9, 123.0, 121.9, 119.9, 116.7, 111.4, 51.6, 20.5, 20.1; HRMS (ESI) *m*/*z* [M + H]^+^ calcd for C_30_H_24_NO_4_ 462.1699, found 462.1694.

### Synthesis of **6**

In order to verify its formation during the synthesis of **4h**, **6** was synthesized via the reduction of **4h** in a ThalesNano H-Cube Pro flow reactor according to the following procedure. First, 10 mg (0.027 mmol) of **4h** was dissolved in 7 mL of a 1:1 MeOH/PhMe mixture (0.004 M **4h**), and the mixture was pumped with a flow rate of 1 mL/min and hydrogenated at 40 °C (heating via the flow reactor’s built-in heater) with a Raney nickel catalyst. Then, the solvents were evaporated in vacuo. The retention time of the product (**6**) matched that of the side product seen during the synthesis of **4h**.

#### 5-Amino-2′,7′-dimethylspiro[indoline-3,9′-xanthen]-2-one (**6**)

Yield 95% (9.2 mg); light brown solid, which darkened during storage; ^1^H NMR (500 MHz, DMSO-*d*_6_) δ 10.20 (s, 1H), 7.10 (s, 4H), 6.73 (d, *J* = 8.2 Hz, 1H), 6.49 (d, *J* = 7.9 Hz, 1H), 6.43 (s, 2H), 6.16 (s, 1H), 4.78 (s, 2H), 2.15 (s, 6H); ^13^C{^1^H} NMR (125.7 MHz, DMSO-*d*_6_) δ 178.4, 148.4, 144.7, 137.5, 132.3, 131.3, 129.5, 127.2, 121.2, 116.4, 113.8, 111.2, 110.3, 52.8, 20.2; HRMS (ESI) *m*/*z* [M + H]^+^ calcd for C_22_H_19_N_2_O_2_ 343.1440, found 343.1440.

### Conversion of **3a** to **4a**

To a 10 mL reaction vial suitable for microwave reaction, equipped with a magnetic stirrer, were added 0.15 mmol (51.8 mg) of **3a**, 0.15 mmol (1 equiv) of *p*-cresol (**2a**), and 15 μL (0.23 mmol, 1.5 equiv) of MeSO_3_H. The vial was sealed, and the mixture was heated to 150 °C in 30 s and held at that temperature for 60 min. Then, the reaction mixture was analyzed by HPLC-MS measurements, and a conversion of 94% was determined.

### Cell Culture

All cell lines were purchased from the American Type Culture Collection (ATCC, Manassas, VA). A549 human lung adenocarcinoma cells were maintained in Dulbecco’s modified Eagle’s medium (DMEM). HL-60 human promyelocytic leukemia cells, 4T1 mouse breast cancer cells, human melanoma HT168 cells, and HT29 human colorectal adenocarcinoma cells were maintained in Roswell Park Memorial Institute 1640 medium (RPMI-1640). B16 mouse melanoma cells were maintained in DMEM/F12 (all media from Capricorn Scientific) medium containing 10% FCS. All media were supplemented with 2 mM GlutaMAX, 100 units/mL penicillin, and 100 μg/mL streptomycin (Life Technologies, Carlsbad, CA). Cell cultures were maintained at 37 °C in a humidified incubator in an atmosphere of 5% CO_2_ (Sanyo).

### Cytotoxicity Assay

The cytotoxicity of the synthesized molecules was determined on A549, B16, 4T1, HT168, CT29, and HL-60 cells using the fluorescent resazurin assay.^19^ Briefly, cells were seeded into 384-well plates (1000 cells/well) (Corning Life Sciences) in medium and incubated overnight. Test compounds were dissolved in dimethyl sulfoxide (DMSO) and diluted to the appropriate concentrations in the culture media. Cells were treated with increasing concentrations of test compounds (1–30 μM). The positive control was doxorubicin (4T1, IC_50_ = 1.642 ± 0.287 μM; A549, IC_50_ = 3.543 ± 1.582 μM; B16, IC_50_ = 0.4917 ± 0.253 μM; HT29, IC_50_ = 0.2732 ± 0.088 μM; HT168, IC_50_ = 0.8452 ± 0.311 μM; HL60, IC_50_ = 0.1674 ± 0.031 μM). Cell viability was determined after incubation for 72 h. Resazurin reagent (Sigma-Aldrich) was added at a final concentration of 25 μg/mL. After a 2 h incubation at 37 °C and 5% CO_2_, the fluorescence (530 nm excitation/580 nm emission) was recorded on a multimode microplate reader (Cytofluor4000, PerSeptive Biosytems). Viability was calculated with respect to untreated control cells and blank wells containing media without cells. IC_50_ values (50% inhibitory concentration) were calculated by GraphPad Prism 5 (GraphPad, La Jolla, CA).

### Antibacterial Activity

All reagents were purchased from Sigma-Aldrich (Budapest, Hungary). *E. coli*-GFP and *B. subtilis*-GFP bacteria were grown in lysogeny broth (Luria-Bertani broth, LB, 10 g of tryptone, 5 g of yeast extract, 10 g/L NaCl, pH 7.0) medium overnight (ON) at 37 °C in an incubator with continuous shaking.^20^ The ON bacterial culture was diluted (*E. coli*-GFP, 1:10 000; *B. subtilis*-GFP, 1:1000) in LB medium containing 10 μg/mL ampicillin. Then, 200 μL of diluted *E. coli* suspension per well was transferred to 96-well plates, and different agents were added at the appropriate concentration and then incubated in a shaker (600 rpm) at 37 °C. Test compounds were dissolved in 3-fold amounts of dimethyl sulfoxide (DMSO). Cells were treated with increasing concentrations of test compounds (1–30 μM). Doxycyclin [IC_50_ values of 0.232 ± 0.022 μM (6 h) and 0.783 ± 0.089 μM (20 h) for *E. coli* and IC_50_ values of 0.049 ± 0.007 μM (6 h) and 0.189 ± 0.025 μM (20 h) for *B. subtilis*] and gentamicin [IC_50_ values of 1.350 ± 0.348 μM (6 h) and 0.854 ± 0.129 μM (20 h) for *E. coli* and IC_50_ values of 0.468 ± 0.087 μM (6 h) and 0.497 ± 0.057 μM (20 h) for *B. subtilis*] were used as positive controls. For induction of GFP expression, IPTG (0.1 mM final concentration) was added to each well and incubated at 37 °C. Following incubation, samples were centrifuged at 2750 rpm and room temperature (RT) for 5 min and washed twice with 200 μL of PBS, with an intermitent centrifugation step between the two washes. After a third centrifugation step, the bacterial cells were suspended in 100 μL of PBS. The quantity of bacteria was estimated according to the fluorimetric measurements. The fluorescence of the bacterial cells was measured with a Victor 1420 multilabel counter (PerkinElmer, Waltham, MA) at 485/535 nm for 1 s per well controlled by Wallac 1420 Manager software. Each treatment was repeated in at least two wells per plate during the experiments. The percent fluorescence intensity based on the quantity of *E. coli*-GFP cells was calculated using untreated control values as 100%. The error was represented by the standard deviation (SD). IC_50_ values (50% inhibitory concentration) were calculated by GraphPad Prism 5.

## Data Availability

The data underlying this study are available in the published article and its Supporting Information.

## Supporting Information Available

Green chemical calculations, computational details, and copies of ^1^H, ^13^C{^1^H}, ^19^F{^1^H}, and two-dimensional NMR spectra (PDF)
FAIR data, including the primary NMR FID files, for compounds **3a–s**, **4a–s**, **6**, and **10** (ZIP)
