# Supplementary material for: 3,3-Bis(hydroxyaryl)oxindoles and Spirooxindoles Bearing a Xanthene Moiety: Synthesis, Mechanism, and Biological Activity
Source: J Org Chem. 2025 May 7;90(19):6454–67. doi: 10.1021/acs.joc.5c00270 (PMC12090213; doi:10.1021/acs.joc.5c00270)
Supplement: Supplementary file 1 — jo5c00270_si_001.pdf [file jo5c00270_si_001.pdf]

Supporting Information

3,3-Bis(hydroxyaryl)oxindoles and Spirooxindoles Bearing a Xanthene Moiety: Synthesis, Mechanism and Biological Activity

Dániel Steinsits,[a] Bettina Rávai,[a, b] Zsolt Kelemen,[c] László Hackler Jr.,[d] Viktor Vernyik,[d] László G. Puskás,[d] Erika Bálint[a]*

[a] *Department of Organic Chemistry and Technology, Faculty of Chemical Technology and Biotechnology, Budapest University of Technology and Economics, Műegyetem rkp. 3., H-1111 Budapest, Hungary*

[b] *CycloLab Cyclodextrin Research and Development Ltd., Illatos út 7., H-1097 Budapest, Hungary*

[c] *Department of Inorganic and Analytical Chemistry, Faculty of Chemical Technology and Biotechnology, Budapest University of Technology and Economics, Műegyetem rkp. 3., H-1111 Budapest, Hungary*

[d] *Anthelos Kft., Alsó kikötő sor 11., H-6726 Szeged, Hungary*

Email:
Erika Bálint* - balint.erika@vbk.bme.hu
*Corresponding author

Table of Contents

1. **Green metrics calculations** **S3**
2. **Computational details** **S5**
3. **Cartesian coordinates, total energy and Gibbs free energy values** **S5**
4. **^{1}H NMR, ^{13}C NMR and ^{19}F NMR spectra of the synthesized compounds** **S16**
5. **2D NMR spectra of compound 3a** **S90**
6. **2D NMR spectra of compound 4a** **S93**

1. Green metrics calculations

Herein, two calculations are presented for the green metrics (atom-economy and E-factor) mentioned (comparing the work of Ferreira et al.[S1] to our solvent-free synthesis of **4a**). For the calculations of E-factors, it was hypothesized, that the solvents are reused, and thus only the mass of the reagents and the products were included in our estimations.

Table S1. Synthesis of **4k** by Ferreira et al.[S1]

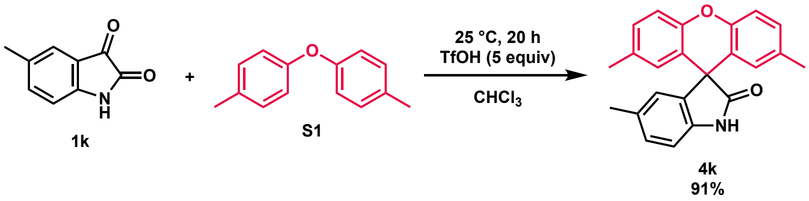

	5-Me-isatin (**1k**)	*p*-Tolyl ether (**S1**)	Triflic acid	Product (**4k**)
Molar mass (g/mol)	161.16	198.27	150.07	341.41
Mass (g)	0.161	0.198	0.750	0.311
Equivalent	1	1	5	–
Moles (mmol)	1	1	5	0.91

Atom-economy

$$AE_{Ferreira} = \frac{M(product)}{M(5\text{-Me-isatin}) + M(p\text{-tolyl ether}) + 5\cdot M(TfOH)} \cdot 100\% = \frac{341.41}{161.16 + 198.27 + 5\cdot 150.07} \cdot 100\% = 30.8\%$$

E-factor (1.00 mmol scale)

$$E_{Ferreira} = \frac{m(waste)}{m(product)} = \frac{m(starting\ materials) - m(product)}{m(product)} = \frac{(0.161 + 0.198 + 0.750) - 0.311}{0.311} = 2.57$$

Table S2. Synthesis of **4a** in this paper

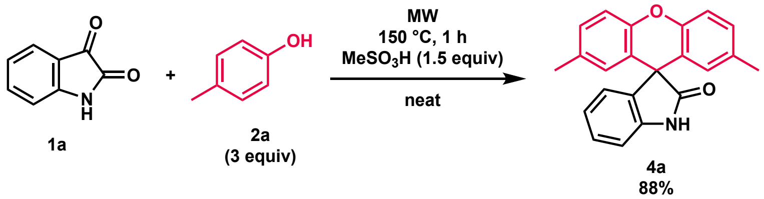

	Isatin (**1a**)	*p*-Cresol (**2a**)	Methanesulfonic acid	Product (**4a**)
Molar mass (g/mol)	147.13	108.14	96.10	327.38
Mass (g)	0.147	0.324	0.144	0.288
Equivalent	1	3	1.5	–
Moles (mmol)	1	3	1.5	0.88

[S1] Ferreira, J. C.; Hood, J. C.; Klumpp, D. A. *Tetrahedron* **2022**, *128*, 133123.

Atom-economy

$$AE_{Steinsits} = \frac{M(product)}{M(Isatin) + 2\cdot M(p\text{-cresol}) + 1.5\cdot M(MeSO_3H)} \cdot 100\% = \frac{327.38}{147.13 + 2\cdot 108.14 + 1.5\cdot 96.10} \cdot 100\% = 64.5\%$$

E-factor (1.00 mmol scale)

$$E_{Steinsits} = \frac{m(waste)}{m(product)} = \frac{m(starting\ materials) - m(product)}{m(product)} = \frac{(0.147 + 0.324 + 0.144) - 0.288}{0.288} = 1.14$$

Below listed are the simplest examples from previously reported procedures cited in our paper and a detailed comparison including our work in Table S3.

Table S3. Comparison of previous methods and our work.

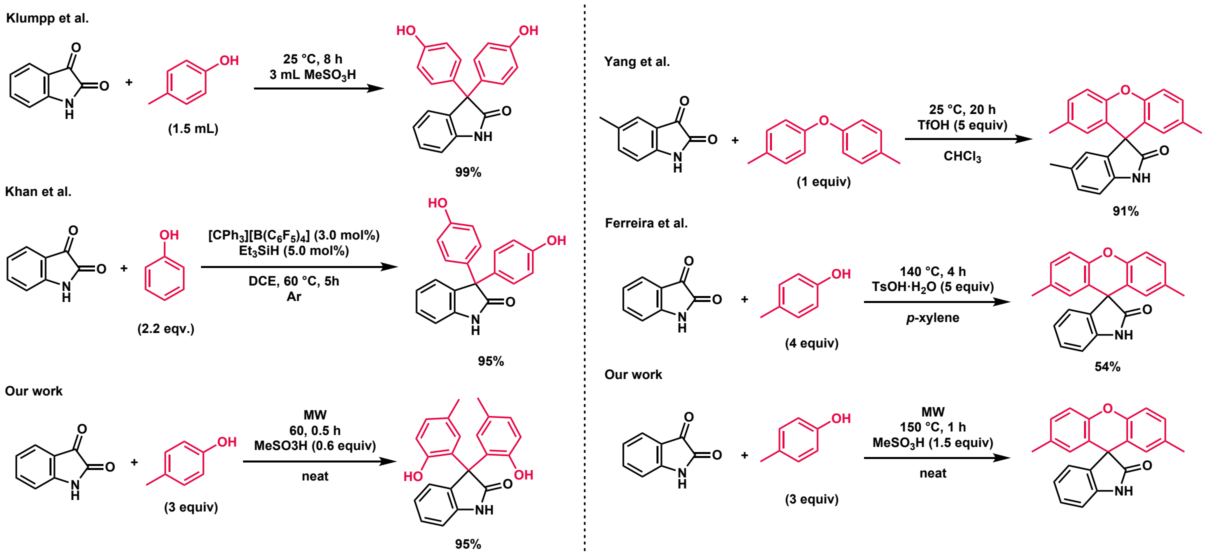

Compounds	Yield (%)	T (°C)	t (h)	AE (%)	E-factor	Ref.
3,3-Bis(hydroxyaryl)oxindoles	99	25	8	5.1	11.53	Klumpp[S2]
	95	60	5	86.0	0.29	Khan[S3]
	95	60	0.5	82.0	0.61	This work
Xanthene-based spirooxindoles	54	140	4	21.4	7.66	Yang[S4]
	91	25	20	30.8	2.57	Ferreira[S1]
	88	150	1	64.5	1.14	This work

[S2] Klumpp, D. A.; Yeung, K. Y.; Prakash, G. S.; Olah, G. A. *J. Org. Chem.* **1998**, *63*, 4481.
[S3] Khan, J.; Tyagi, A.; Yadav, N.; Mahato, R.; Hazra, C. K. *J. Org. Chem.* **2021**, *86*, 17833.
[S4] Yang, H.; Takrouri, K.; Chorev, M. *Curr. Org. Chem.* **2012**, *16*, 1581.

2. Computational details

Calculations based on density functional theory were carried out with Gaussian 16 program package.[S5] Geometry optimizations in succession with frequency calculations were performed at the ωB97X-D/def2-TZVPP level of theory.

3. Cartesian coordinates, total energy and Gibbs free energy values

16
isatin scf done: -513.088135 Gibbs free energy. -513.004443

C	0.162721	0.000000	-0.436616
C	-0.121813	0.000000	0.913966
C	0.954364	0.000000	1.795648
C	2.271861	-0.000000	1.350125
C	2.542885	-0.000000	-0.010510
C	1.481530	-0.000000	-0.895833
H	-1.139957	0.000000	1.278332
H	0.756397	0.000000	2.859485
H	3.080959	-0.000000	2.066571
H	3.557480	-0.000000	-0.386043
C	1.443491	-0.000000	-2.365172
C	-0.084631	0.000000	-2.723739
O	2.329295	-0.000000	-3.168169
O	-0.581507	0.000000	-3.811694
N	-0.730822	0.000000	-1.509270
H	-1.730823	0.000000	-1.423470

17
Int-1 scf done: -513.427363 Gibbs free energy:

C	0.132910	0.000000	-0.423550
C	-0.142612	0.000000	0.924961
C	0.943275	0.000000	1.790894
C	2.282613	-0.000000	1.356898
C	2.567854	-0.000000	0.020516
C	1.484696	-0.000000	-0.886979
H	-1.155188	0.000000	1.301382
H	0.748202	0.000000	2.855785
H	3.077078	-0.000000	2.088229
H	3.585012	-0.000000	-0.345910
C	1.409775	-0.000000	-2.263444

[S5] Frisch, M. J.; Trucks, G. W.; Schlegel, H. B.; Scuseria, G. E.; Robb, M. A.; Cheeseman, J. R.; Scalmani, G.; Barone, V.; Petersson, G. A.; Nakatsuji, H.; Li, X.; Caricato, M.; Marenich, A. V.; Bloino, J.; Janesko, B. G.; Gomperts, R.; Mennucci, B.; Hratchian, H. P.; Ortiz, J. V.; Izmaylov, A. F.; Sonnenberg, J. L.; Williams-Young, D.; Ding, F.; Lipparini, F.; Egidi, F.; Goings, J.; Peng, B.; Petrone, A.; Henderson, T.; Ranasinghe, D.; Zakrzewski, V. G.; Gao, J.; Rega, N.; Zheng, G.; Liang, W.; Hada, M.; Ehara, M.; Toyota, K.; Fukuda, R.; Hasegawa, J.; Ishida, M.; Nakajima, T.; Honda, Y.; Kitao, O.; Nakai, H.; Vreven, T.; Throssell, K.; Montgomery Jr., J. A.; Peralta, J. E.; Ogliaro, F.; Bearpark, M. J.; Heyd, J. J.; Brothers, E. N.; Kudin, K. N.; Staroverov, V. N.; Keith, T. A.; Kobayashi, R.; Normand, J.; Raghavachari, K.; Rendell, A. P.; Burant, J. C.; Iyengar, S. S.; Tomasi, J.; Cossi, M.; Millam, J. M.; Klene, M.; Adamo, C.; Cammi, R.; Ochterski, J. W.; Martin, R. L.; Morokuma, K.; Farkas, O.; Foresman, J. B.; Fox, D. J. *GAUSSIAN 16, Revision C.01*, Gaussian, Inc., Wallingford CT, **2016**.

C -0.071856 0.000000 -2.671075
O 2.327376 -0.000000 -3.147257
O -0.444689 0.000000 -3.802304
N -0.755457 0.000000 -1.485368
H -1.759511 0.000000 -1.414491
H 1.943898 -0.000000 -4.043182

16
p-cresol scf done: -346.803894 Gibbs free energy: -346.702973
C -0.038257 0.010005 0.047489
C -0.004636 0.083299 1.434802
C 1.210690 0.074732 2.092521
C 2.418152 -0.005564 1.400930
C 2.361706 -0.079316 0.016623
C 1.150455 -0.072034 -0.661544
H -0.936019 0.144854 1.980831
H 1.221685 0.131124 3.174649
C 3.729335 -0.001026 2.137957
H 3.280861 -0.145025 -0.552833
H 1.135360 -0.131871 -1.743997
O -1.256893 0.020621 -0.556207
H -1.145704 -0.035055 -1.504794
H 4.563220 -0.190064 1.462871
H 3.905980 0.961062 2.622823
H 3.748552 -0.765742 2.915878

3
water scf done: -76.441481 Gibbs free energy: -76.437361
O 0.006881 0.000000 0.004866
H 0.044174 -0.000000 0.960230
H 0.920038 0.000000 -0.278429

33
Int-2 scf done: -860.259969 Gibbs free energy: -860.036956
C -0.055289 0.077177 0.095121
C 0.086348 0.148324 1.572086
C 1.503322 0.299544 2.050422
C 2.600605 0.325194 1.156927
C 2.322528 0.250815 -0.176717
C 1.003377 0.115093 -0.718443
C -0.884329 1.193942 2.266643
C -2.262057 1.294831 1.658079
C -3.201094 0.886450 2.595313
N -2.540461 0.510396 3.786486
C -1.223058 0.670286 3.690834
C -2.681434 1.807627 0.447829
C -4.045000 1.848587 0.177800
C -4.964253 1.397134 1.113509
C -4.554630 0.914830 2.352510
O -0.292175 2.461963 2.307530
O -0.386856 0.433474 4.557910

C	3.988312	0.462990	1.702988
H	-1.053842	-0.058159	-0.292891
H	-5.273414	0.593453	3.093231
H	-6.020473	1.435803	0.886313
H	-4.391837	2.244318	-0.765985
H	-1.969569	2.188832	-0.271762
H	-2.991479	0.190588	4.628402
H	0.255258	2.571767	3.088726
O	1.762870	0.408807	3.297135
H	3.152207	0.290378	-0.873104
H	0.883726	0.033723	-1.789085
H	-0.219761	-0.849142	1.929778
H	4.718976	0.463049	0.897921
H	4.093899	1.389568	2.267418
H	4.220882	-0.355308	2.384287
H	0.933580	0.397660	3.909142

33

Int-3 scf done: -860.279075 Gibbs free energy: -860.056606

C	0.141584	0.075975	-0.048738
C	0.052266	0.022164	1.323683
C	1.204885	-0.032257	2.091388
C	2.467998	-0.041038	1.555527
C	2.550115	0.002692	0.165029
C	1.410316	0.059930	-0.623519
N	0.831685	-0.058649	3.471134
C	-0.456725	0.027502	3.595232
C	-1.161977	-0.004994	2.228123
C	-2.145189	1.134002	2.060491
C	-3.408953	0.907535	1.524880
C	-4.305374	1.946246	1.317264
C	-3.903026	3.232914	1.666597
C	-2.653469	3.479912	2.204283
C	-1.771217	2.430430	2.399553
O	-0.525031	2.595692	2.926789
C	-5.668422	1.699477	0.735095
O	-1.859704	-1.229331	2.198218
O	-1.040644	0.025691	4.737474
H	-1.300513	-1.912324	1.822303
H	3.357044	-0.076662	2.169490
H	3.523852	-0.003692	-0.304169
H	1.508955	0.098824	-1.699027
H	-0.748096	0.136330	-0.661181
H	1.475033	-0.096540	4.247977
H	-3.691425	-0.103678	1.268873
H	-4.582361	4.062563	1.517337
H	-2.362545	4.488975	2.470026
H	-0.363561	3.517011	3.132799
H	-6.450538	2.038001	1.415310
H	-5.795159	2.242076	-0.202344
H	-5.831253	0.642008	0.535691

H	-2.001027	0.086425	4.636055

33

TS-1 scf done: -860.245537 Gibbs free energy: -860

C	0.002164	0.041501	-0.003092
C	0.003829	0.014815	1.371001
C	1.198766	-0.004873	2.101580
C	2.423130	0.020516	1.454916
C	2.434313	0.052144	0.068217
C	1.240217	0.054983	-0.642183
N	-1.095112	0.039537	2.244399
C	-0.699569	0.065723	3.537767
C	0.832112	-0.022169	3.502328
O	1.510705	0.635449	4.419015
O	-1.351007	0.118225	4.549831
C	0.960194	-1.968219	4.211942
C	0.018508	-2.700719	3.434775
C	0.449579	-3.342451	2.275059
C	1.793754	-3.390066	2.003013
C	2.784124	-2.845032	2.862546
C	2.352508	-2.173886	3.965345
O	-1.265000	-2.767159	3.742532
C	4.235829	-3.021845	2.532174
H	0.651315	-1.677215	5.209569
H	-0.917525	0.068301	-0.570191
H	1.268118	0.081637	-1.723019
H	3.374483	0.081851	-0.463152
H	3.344341	0.024728	2.020317
H	-2.061717	0.055656	1.966142
H	0.948748	0.805376	5.188462
H	3.059657	-1.713591	4.642545
H	2.119332	-3.896786	1.102155
H	-0.274486	-3.827128	1.636165
H	-1.478672	-2.280237	4.546153
H	4.460416	-2.615865	1.544663
H	4.873106	-2.524126	3.260159
H	4.503391	-4.078867	2.516165

33

TS-2 scf done: -860.235481 Gibbs free energy: -860.015245

C	0.028015	-0.191047	-0.062042
C	-0.068954	0.055027	1.347410
C	1.150180	0.306085	2.068677
C	2.328928	0.449629	1.417816
C	2.365580	0.301081	0.016315
C	1.258984	-0.006270	-0.733716
C	-1.294087	-0.291985	2.201405
C	-1.250728	-1.071490	3.486236
C	-1.868309	-0.300921	4.473027
N	-2.170751	0.999737	3.961312
C	-1.693880	1.103363	2.733615

C	-0.843468	-2.358714	3.764960
C	-1.051326	-2.854942	5.048786
C	-1.666953	-2.074477	6.015625
C	-2.090786	-0.774929	5.742666
O	-1.425222	2.073533	2.022121
O	-2.350720	-0.744781	1.365787
C	1.311097	-0.185017	-2.220101
O	-0.998462	-0.519255	-0.811834
H	1.098647	0.416208	3.144764
H	-2.567015	-0.172393	6.503485
H	-1.822049	-2.478335	7.006418
H	-0.729600	-3.856705	5.294811
H	-0.365421	-2.965921	3.007646
H	-2.468323	1.780974	4.523667
H	-2.918498	-1.346951	1.850222
H	3.312157	0.428693	-0.494063
H	3.235639	0.672753	1.960454
H	2.318131	-0.012452	-2.591907
H	1.003653	-1.192019	-2.502531
H	0.631769	0.506187	-2.719146
H	-1.775234	-0.697662	-0.256170
H	-0.553140	1.346868	1.291206

33

TS-3 scf done: -860.239868 Gibbs free energy: -860.019976

C	-0.030495	-0.021491	0.008053
C	-0.011443	-0.001075	1.388928
C	1.205615	0.012926	2.075149
C	2.414131	-0.015144	1.420740
C	2.380741	-0.076702	0.030818
C	1.181083	-0.084710	-0.667899
N	0.980139	0.042904	3.478759
C	-0.322104	0.013076	3.711922
C	-1.092732	0.235230	2.396896
C	-1.860544	1.518601	2.238918
C	-1.555528	2.647084	2.987328
C	-2.280166	3.815938	2.788686
C	-3.287535	3.853793	1.848560
C	-3.608213	2.736798	1.074809
C	-2.878197	1.582424	1.286566
O	-0.537213	2.562373	3.880972
C	-4.714785	2.797413	0.061182
O	-2.124137	-0.834354	2.634135
O	-0.980026	-0.435907	4.653586
H	-1.854828	-1.678325	2.245632
H	3.352612	0.005486	1.956566
H	3.313664	-0.110620	-0.514536
H	1.190732	-0.125739	-1.747490
H	-0.963680	0.012360	-0.537536
H	1.673675	-0.180199	4.173758
H	-3.120830	0.696587	0.712369

H	-3.841024	4.773620	1.709006
H	-2.046255	4.697442	3.372924
H	-0.503066	3.343425	4.434474
H	-5.670310	3.010722	0.541426
H	-4.532386	3.588081	-0.667081
H	-4.814350	1.857963	-0.479705
H	-1.942696	-0.839854	3.758820

46

TS-4 scf done: -1130.625736 Gibbs free energy: -1130.302156

C	0.021910	0.007442	-0.017158
C	0.008022	0.012521	1.405047
C	1.255977	-0.005142	2.090907
C	2.434149	0.156586	1.431466
C	2.388402	0.259181	0.013307
C	1.226053	0.166047	-0.703975
C	3.751422	0.247824	2.136850
O	-1.086326	-0.113691	-0.729689
C	-0.766924	1.966772	1.848183
C	-2.022152	2.032046	1.127364
C	-1.791564	2.691237	-0.095013
N	-0.462530	3.040871	-0.194068
C	0.234654	2.714838	0.943807
C	-2.808262	2.944495	-0.999435
C	-4.080635	2.523351	-0.662506
C	-4.340230	1.875132	0.549082
C	-3.323725	1.631037	1.447694
C	-0.621866	2.038394	3.307199
C	0.181152	3.046576	3.862187
C	0.326400	3.220071	5.226365
C	-0.334185	2.328706	6.067719
C	-1.114436	1.310006	5.558433
C	-1.266802	1.159910	4.189842
O	-1.997465	0.132452	3.678435
C	1.155948	4.340197	5.786505
O	1.392191	2.950970	1.152018
H	-2.614835	3.454330	-1.932524
H	-4.895241	2.705757	-1.350238
H	-5.349904	1.571453	0.785211
H	-3.524727	1.134521	2.382922
H	-0.037918	3.534242	-0.959384
H	0.699374	3.722194	3.200040
H	-0.234882	2.429478	7.141080
H	-1.611342	0.619945	6.230037
H	-2.329050	-0.418772	4.389043
H	1.803953	3.985212	6.587554
H	1.781680	4.793144	5.019763
H	0.519046	5.121862	6.203390
H	1.241172	-0.084265	3.169506
H	3.319617	0.400493	-0.521690
H	1.217996	0.205554	-1.783622

H	-1.865284	-0.200369	-0.171702
H	4.176908	1.243901	2.005357
H	3.644102	0.063400	3.203543
H	4.464523	-0.471579	1.733368
H	-0.855059	-0.407544	1.904957

33

Int-3 scf done: -860.260398 Gibbs free energy: -860.045666

C	0.029111	-0.028443	-0.007673
C	0.022638	-0.007696	1.376835
C	1.230506	0.002732	2.129842
C	2.464907	0.080761	1.443135
C	2.468647	0.073923	0.075804
C	1.257178	0.001902	-0.637245
N	-1.054752	0.037907	2.213231
C	-0.654328	0.174829	3.527300
C	0.891375	0.101031	3.492696
C	1.657089	0.100406	4.665270
C	2.911595	-0.576704	4.783108
C	3.657232	-0.441157	5.947488
C	3.175233	0.315584	6.986965
C	1.919093	0.957682	6.941577
C	1.178208	0.820249	5.804091
O	3.394169	-1.419417	3.873353
C	1.443522	1.763059	8.114443
O	-1.371195	0.249315	4.476853
O	-0.034701	2.900052	3.182271
H	-0.890443	-0.050317	-0.574457
H	1.289419	-0.010221	-1.718838
H	3.401204	0.134263	-0.465607
H	3.394865	0.164577	1.987230
H	-2.017377	0.107509	1.932849
H	0.576134	3.577856	2.894222
H	0.225374	1.316924	5.720362
H	3.775941	0.407178	7.884082
H	4.598955	-0.965703	6.020562
H	2.768206	-1.600246	3.168030
H	1.417457	1.150813	9.016580
H	2.113549	2.601566	8.308332
H	0.444080	2.157785	7.945398
H	-0.774118	3.372271	3.565464

46

Int-5 scf done: -1130.643151 Gibbs free energy: -1130.319886

C	0.001392	-0.003370	0.002485
C	0.000255	-0.000872	1.398953
C	1.232245	-0.001118	2.047657
C	2.441668	-0.011286	1.365972
C	2.402060	-0.003595	-0.022418
C	1.196229	0.003529	-0.697373
C	-1.304488	-0.040888	2.193051

C	-1.899822	-1.439779	2.183895
C	-1.849727	-1.952834	3.481687
N	-1.329623	-0.991594	4.347257
C	-1.046467	0.179338	3.710878
C	-2.281929	-3.227170	3.783603
C	-2.782942	-4.001454	2.743658
C	-2.841133	-3.512451	1.446866
C	-2.390435	-2.228585	1.155329
C	-2.272631	1.122342	1.761858
C	-3.629639	1.105064	2.363672
C	-4.221209	2.280260	2.812013
C	-3.520304	3.456111	2.748080
C	-2.195228	3.571188	2.239180
C	-1.601323	2.441082	1.805932
O	-4.335882	0.016099	2.432844
C	-1.509866	4.904284	2.253173
O	-0.701928	1.212034	4.222935
C	3.747500	-0.048869	2.107869
O	-1.199462	0.002110	-0.658437
H	-2.236218	-3.612545	4.792714
H	-3.132209	-5.002998	2.953225
H	-3.231990	-4.134206	0.654272
H	-2.417086	-1.852803	0.142909
H	-1.263657	-1.085027	5.346348
H	1.262478	0.019299	3.128461
H	3.324603	-0.000296	-0.588389
H	1.181636	0.011022	-1.781113
H	-1.049922	0.032149	-1.604140
H	4.514301	0.521576	1.585482
H	3.646495	0.359132	3.112410
H	4.108571	-1.074689	2.200877
H	-0.584477	2.464099	1.437707
H	-4.002116	4.355009	3.117320
H	-5.222640	2.238213	3.215020
H	-3.828595	-0.772820	2.165746
H	-1.414524	5.273383	3.274993
H	-0.512912	4.838418	1.823906
H	-2.079256	5.641803	1.686540
H	-2.458707	0.941796	0.686533

45

3a scf done: -1130.302783 Gibbs free energy:

C	-0.003955	0.010500	-0.006505
C	0.000125	0.006140	1.386152
C	1.242706	0.013979	2.043443
C	2.409619	0.088670	1.288927
C	2.368146	0.116571	-0.089816
C	1.155355	0.061073	-0.766088
C	-1.319632	0.041754	2.180565
C	-2.545427	-0.108874	1.302178
C	-3.077917	-1.382341	1.461512

N	-2.324948	-2.076862	2.407909
C	-1.379327	-1.285838	2.983168
C	-4.198804	-1.798659	0.774026
C	-4.800011	-0.885896	-0.087226
C	-4.296292	0.397451	-0.236808
C	-3.162210	0.794290	0.467134
C	-1.440427	1.281042	3.063093
C	-0.667428	2.410226	2.846240
C	-0.801512	3.568897	3.603966
C	-1.755355	3.579941	4.609373
C	-2.554393	2.472187	4.841617
C	-2.406324	1.332314	4.070314
O	-3.196319	0.238282	4.245125
C	0.092217	4.751052	3.349314
O	-0.675087	-1.603895	3.917750
O	1.391075	0.007827	3.382876
C	1.100165	0.038913	-2.269023
H	-4.605062	-2.792532	0.905072
H	-5.681467	-1.182820	-0.639628
H	-4.788829	1.094435	-0.900620
H	-2.766730	1.796463	0.362294
H	-2.552577	-2.980042	2.782163
H	-0.952549	-0.013772	-0.523975
H	3.294923	0.171269	-0.648622
H	3.350655	0.114020	1.821348
H	1.319586	-0.958604	-2.656143
H	0.113338	0.323094	-2.633624
H	1.829710	0.724004	-2.702252
H	0.079310	2.383666	2.063307
H	-1.883919	4.463292	5.222499
H	-3.305127	2.496812	5.623319
H	-0.271195	5.640523	3.862903
H	1.107033	4.554051	3.700148
H	0.154645	4.977770	2.284449
H	-3.764010	0.363473	5.004164
H	0.656254	-0.474097	3.803024

45

TS-4 scf done: -1130.182408 Gibbs free energy:

C	3.837141	-1.391475	0.824534
C	2.561223	-0.902314	0.989322
C	1.728545	-0.561324	-0.065334
C	2.196298	-0.692512	-1.356822
C	3.484182	-1.178506	-1.556606
C	4.290778	-1.528055	-0.482371
N	1.875168	-0.639555	2.196533
C	0.681446	-0.141482	1.953043
C	0.397354	-0.093606	0.464530
C	-0.781729	-1.018678	0.128700
C	-2.053718	-0.660922	0.768452
O	-2.042338	0.798961	1.205564

C	-1.332032	1.650767	0.422842
C	-0.031145	1.309832	0.056805
C	0.733333	2.209622	-0.667249
C	0.220004	3.447125	-1.043653
C	-1.082604	3.761284	-0.671960
C	-1.862012	2.872055	0.050919
C	-3.204375	-0.976647	-0.071394
C	-3.105933	-1.849616	-1.114106
C	-1.884267	-2.411181	-1.538960
C	-0.746273	-1.923221	-0.927856
C	1.045804	4.405372	-1.857608
O	-2.215727	-1.173194	2.128319
C	-1.830238	-3.437518	-2.636322
O	-0.054066	0.267198	2.913224
H	4.462089	-1.653691	1.667172
H	5.287402	-1.907620	-0.660569
H	3.861684	-1.287511	-2.563867
H	1.568066	-0.423072	-2.194145
H	2.230588	-0.800267	3.124417
H	1.747981	1.951699	-0.943963
H	-1.499252	4.720321	-0.954753
H	-2.876003	3.113339	0.338081
H	2.111133	4.267147	-1.673523
H	0.874715	4.257323	-2.926097
H	0.794391	5.440218	-1.626158
H	0.215980	-2.247861	-1.305659
H	-4.013906	-2.109838	-1.649404
H	-4.152531	-0.545822	0.222242
H	-2.450614	-3.150629	-3.489423
H	-0.809911	-3.563896	-3.003974
H	-2.176772	-4.422270	-2.307147
H	-2.408317	-2.103351	2.015796
H	-0.966553	0.464941	2.564952

42

4a scf done: -1053.855224 Gibbs free energy: -1053.569195

C	-0.003841	-0.034822	0.011300
C	-0.000829	-0.010215	1.404350
C	1.218895	0.031368	2.058196
C	2.407053	0.041883	1.338200
C	2.375073	0.007269	-0.038967
C	1.164011	-0.029159	-0.730478
O	1.335186	0.073179	3.414133
C	0.225173	0.341503	4.155556
C	-1.056961	0.319416	3.633451
C	-1.288889	-0.099461	2.196390
C	-2.120791	0.625834	4.479385
C	-1.936957	0.938561	5.814499
C	-0.632299	0.945689	6.308489
C	0.439099	0.655955	5.491820
C	-2.449654	0.611253	1.541328

C	-3.434831	-0.313329	1.211284
N	-3.021416	-1.587401	1.595560
C	-1.779500	-1.582122	2.183188
C	-2.631090	1.945854	1.258006
C	-3.810682	2.350194	0.639336
C	-4.785337	1.417471	0.315483
C	-4.612520	0.066116	0.597195
C	-3.100243	1.253033	6.713567
O	-1.192465	-2.540624	2.603040
C	1.138622	-0.070462	-2.233176
H	-5.374970	-0.657846	0.343009
H	-5.698244	1.743033	-0.165186
H	-3.968590	3.394884	0.410036
H	-1.864658	2.665775	1.514674
H	-3.546825	-2.433727	1.471766
H	-0.957139	-0.059579	-0.503585
H	3.306836	0.015004	-0.591313
H	3.342145	0.079310	1.879671
H	1.704609	0.759058	-2.659783
H	1.582992	-0.994555	-2.607295
H	0.119768	-0.012157	-2.613739
H	-3.124939	0.616901	4.071832
H	-0.455266	1.188808	7.349105
H	1.453226	0.668592	5.866487
H	-2.972131	2.221550	7.199419
H	-4.035749	1.278541	6.156374
H	-3.200365	0.504141	7.501269

4. ^{1}H NMR, ^{13}C NMR and ^{19}F NMR spectra of the synthesized compounds

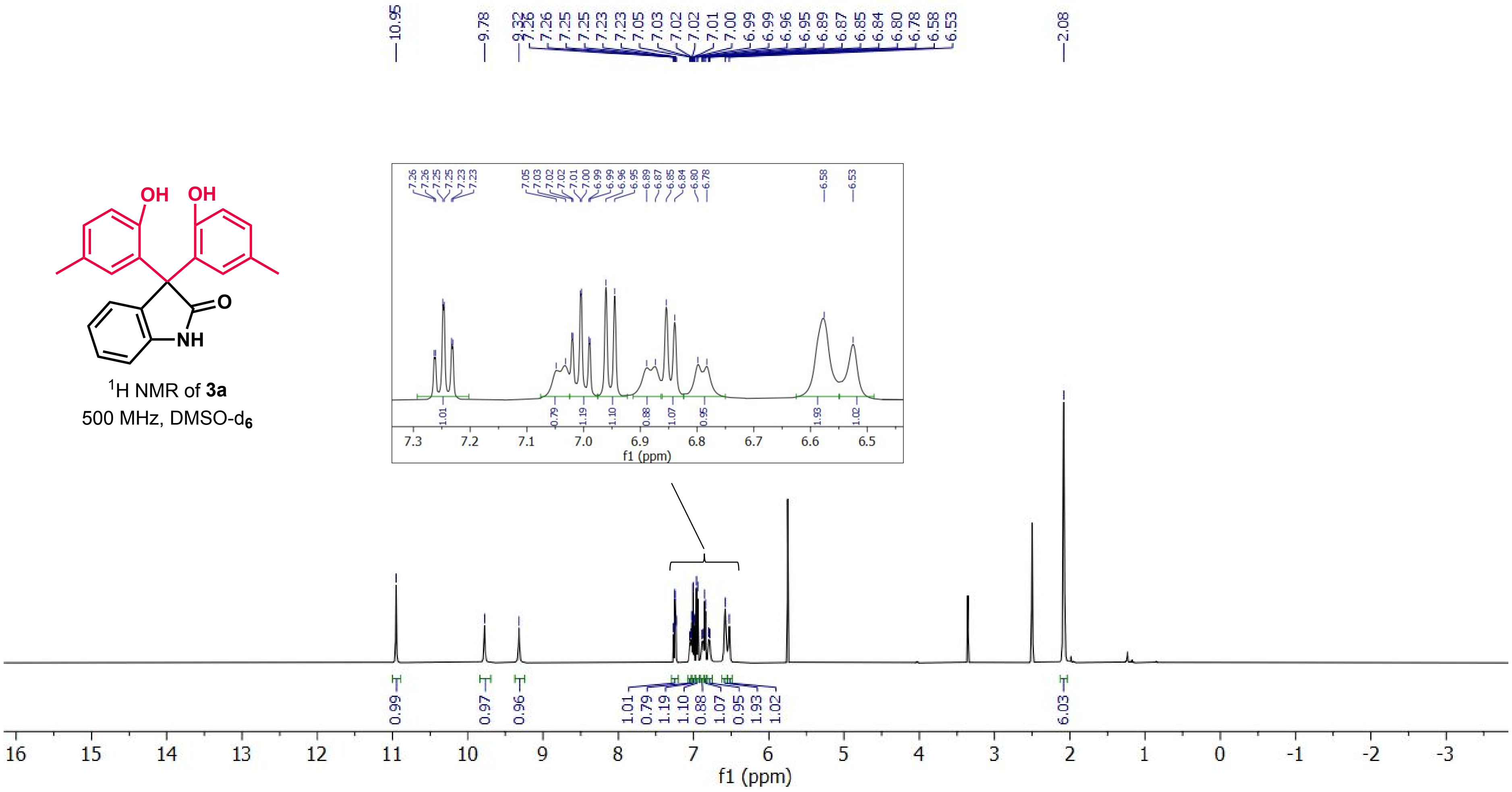

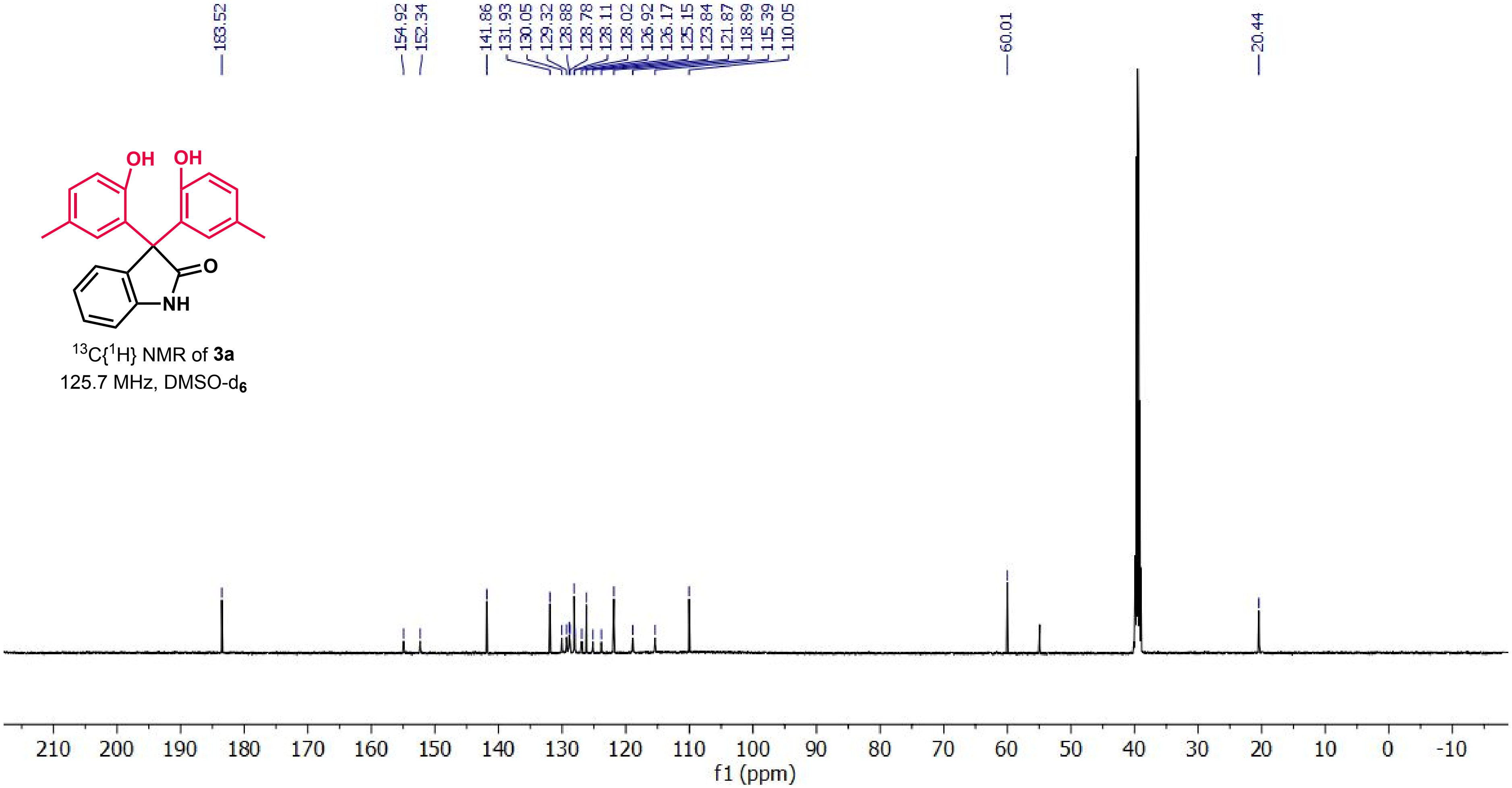

$^{13}C\{^1H\}$ NMR of **3a**
125.7 MHz, DMSO-d_6

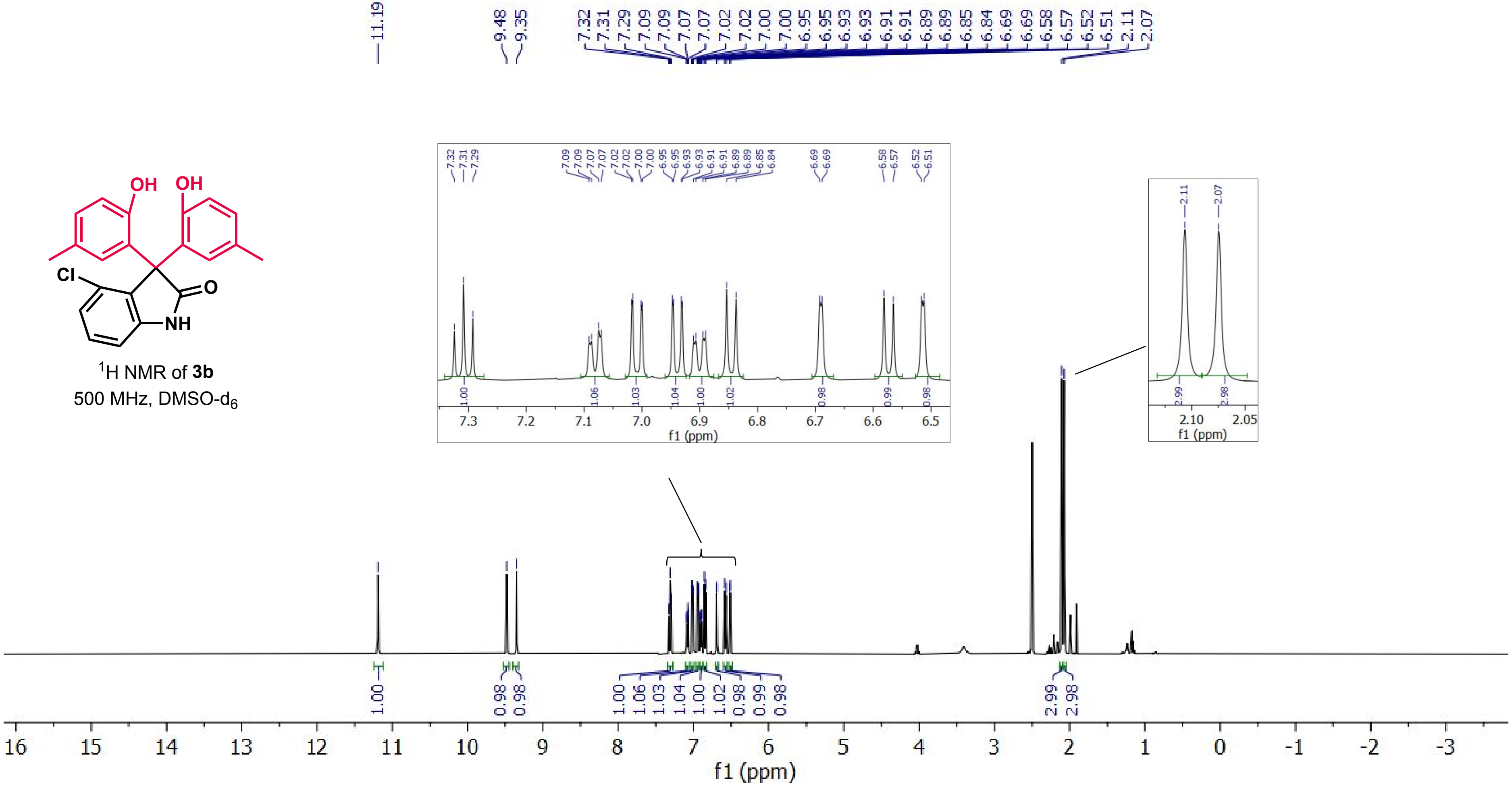

^{1}H NMR of **3b**
500 MHz, DMSO-d$_6$

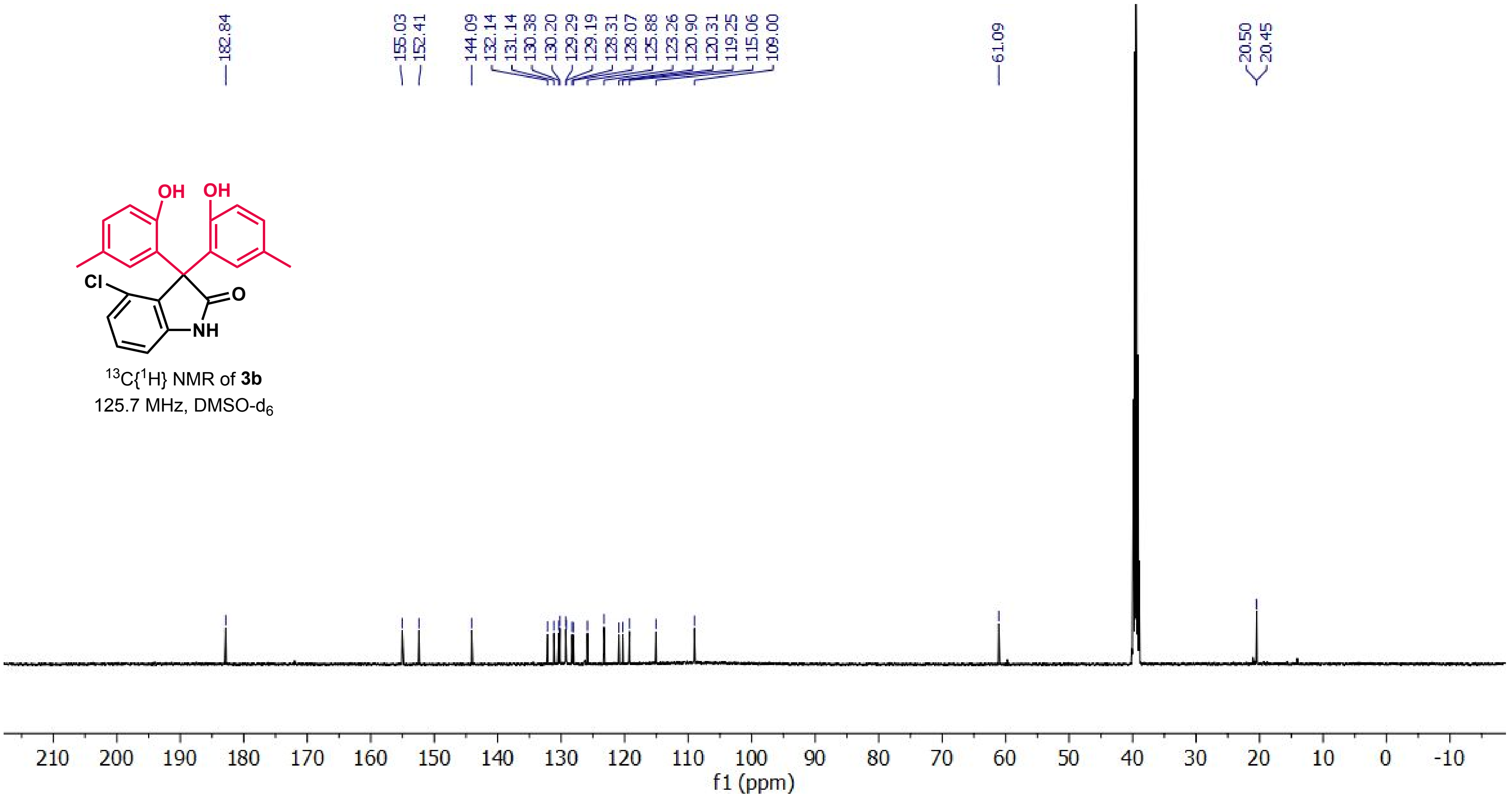

$^{13}C\{^{1}H\}$ NMR of **3b**
125.7 MHz, DMSO-d_6

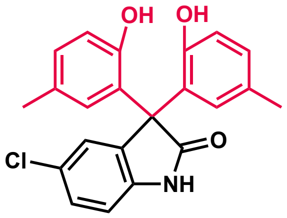

^{1}H NMR of **3c**

500 MHz, DMSO-d$_6$

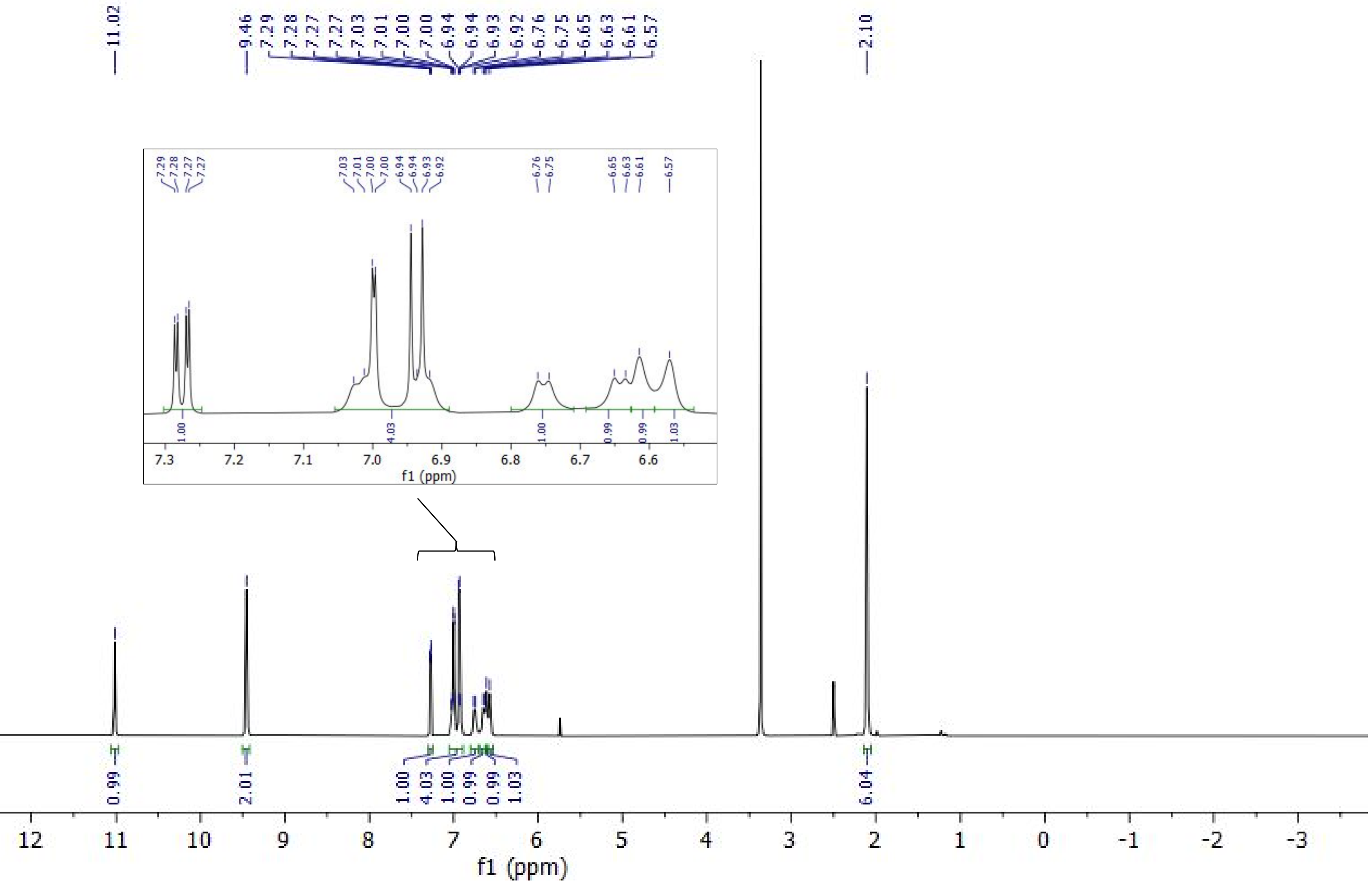

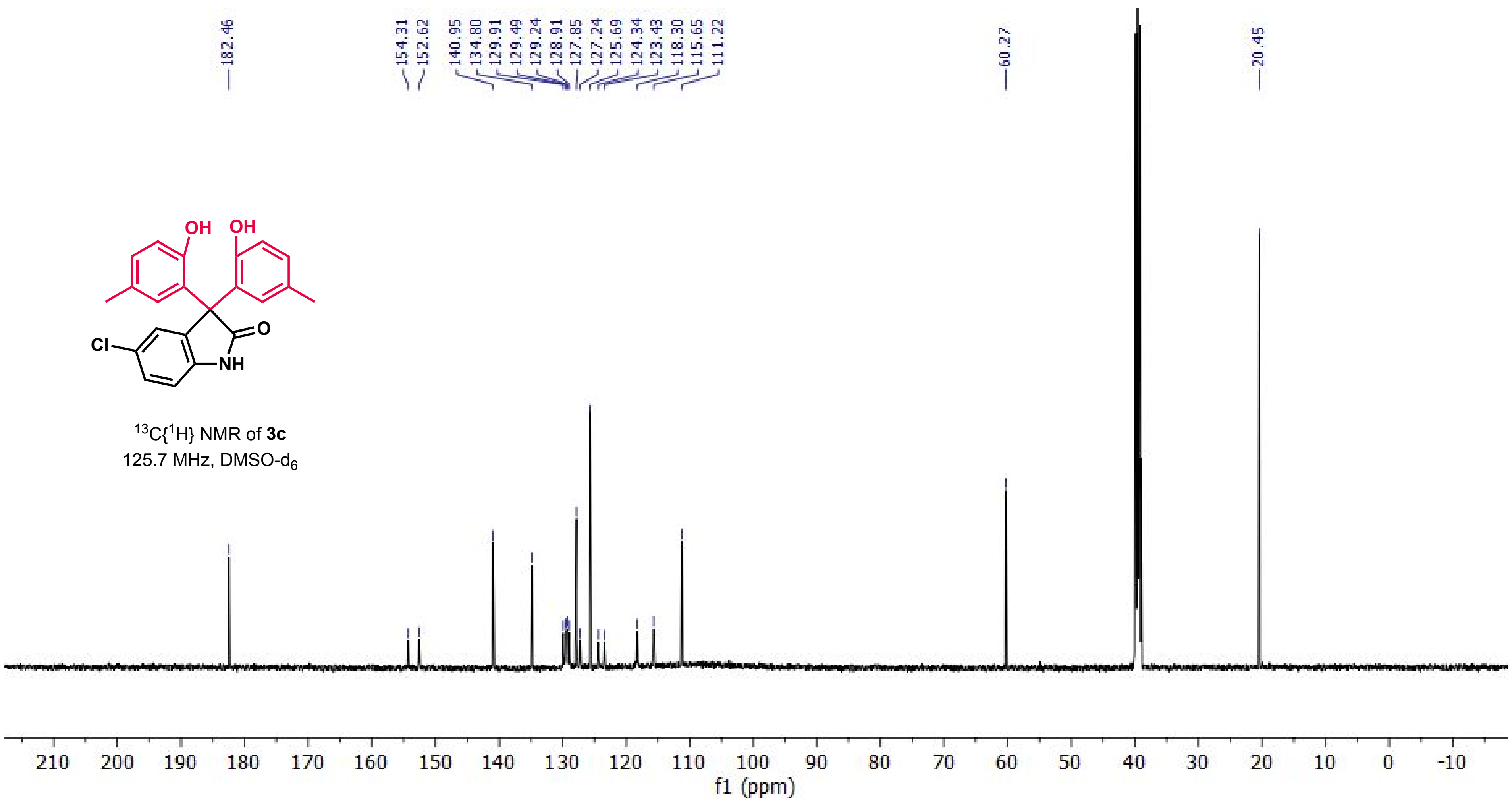

$^{13}C\{^1H\}$ NMR of **3c**
125.7 MHz, DMSO-d_6

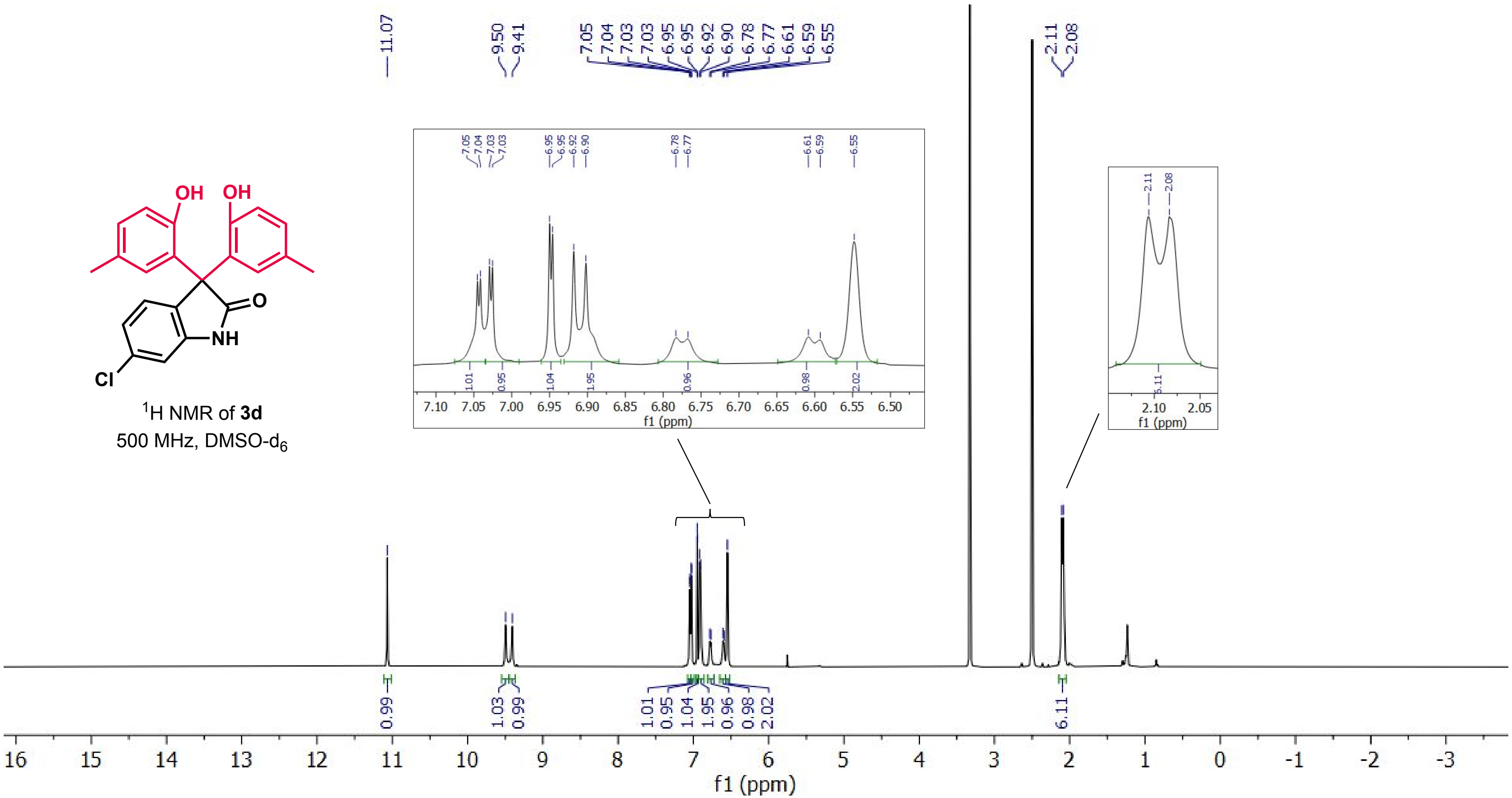

^{1}H NMR of **3d**
500 MHz, DMSO-d_6

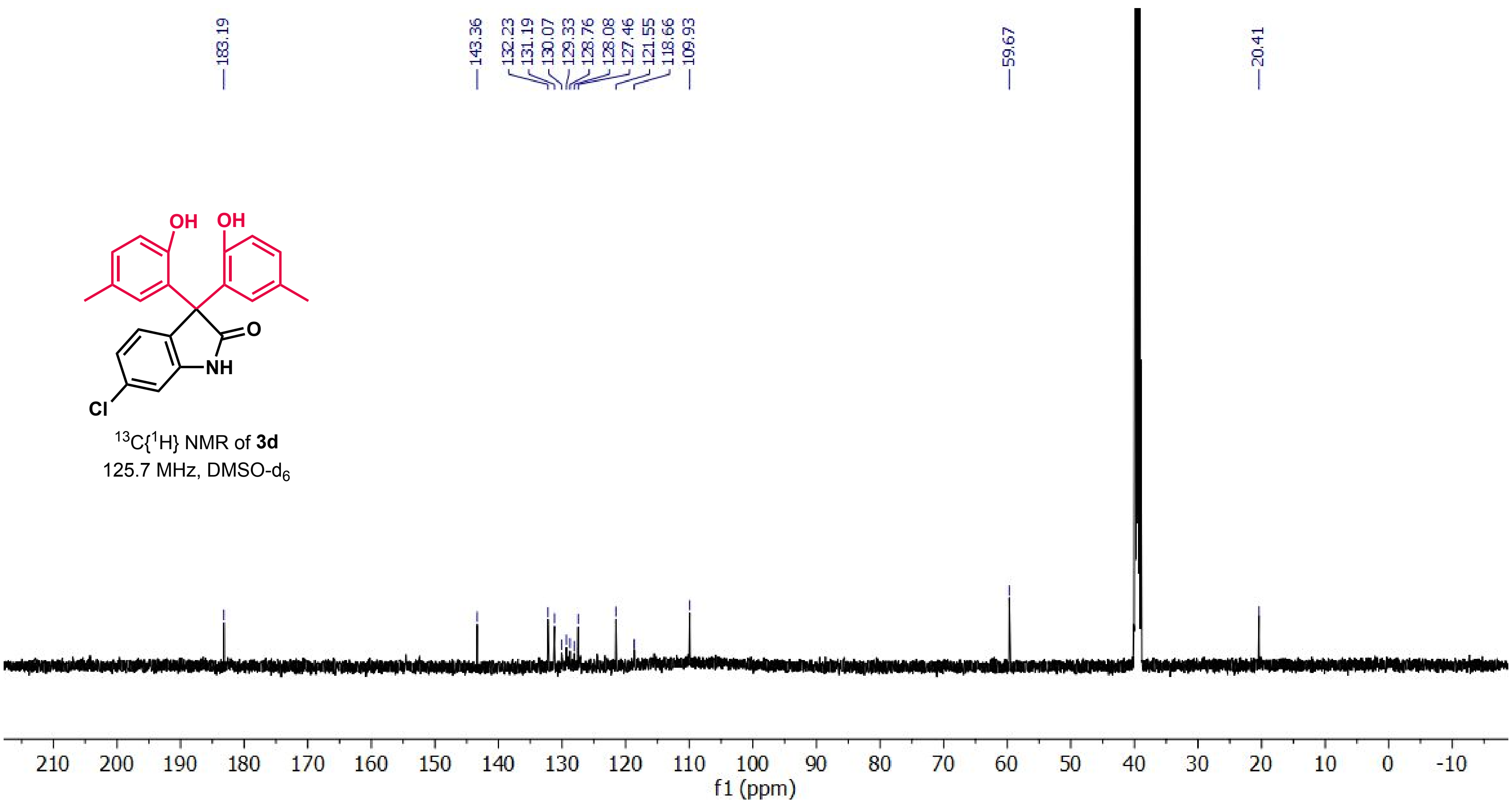
183.19
143.36
132.23
131.19
130.07
129.33
128.76
128.08
127.46
121.55
118.66
109.93
59.67
20.41
OH
OH
O
NH
Cl
13C{1H} NMR of 3d
125.7 MHz, DMSO-d6
210 200 190 180 170 160 150 140 130 120 110 100 90 80 70 60 50 40 30 20 10 0 -10
f1 (ppm)

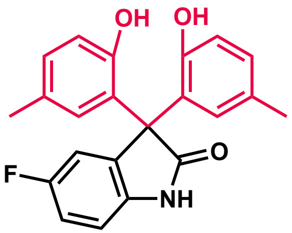

^{1}H NMR of **3e**

500 MHz, DMSO-d_6

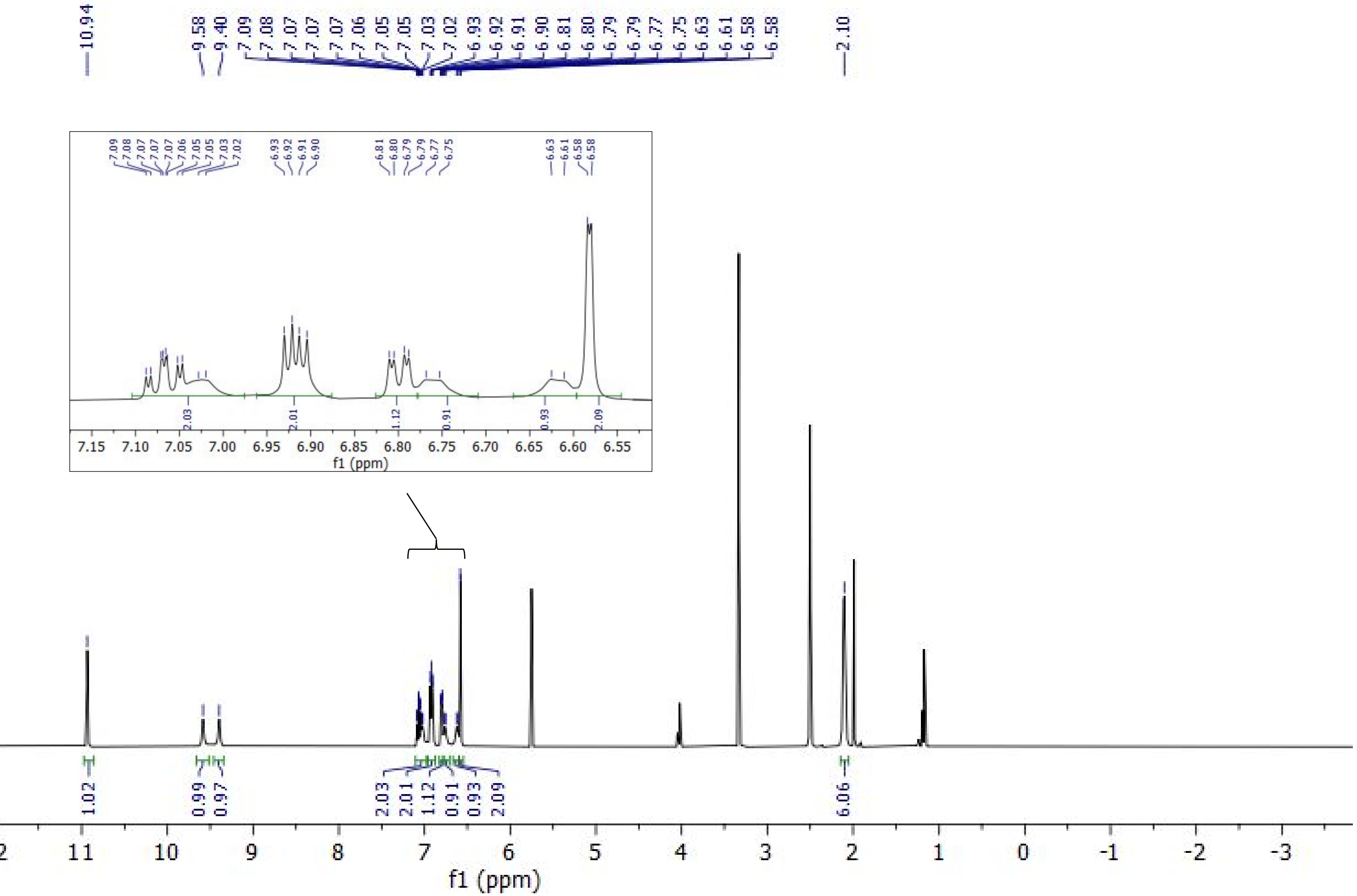

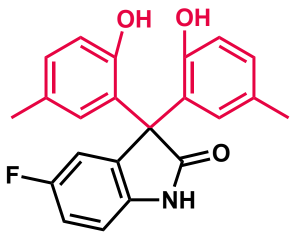

$^{13}C\{^1H\}$ NMR of **3e**
125.7 MHz, DMSO-d_6

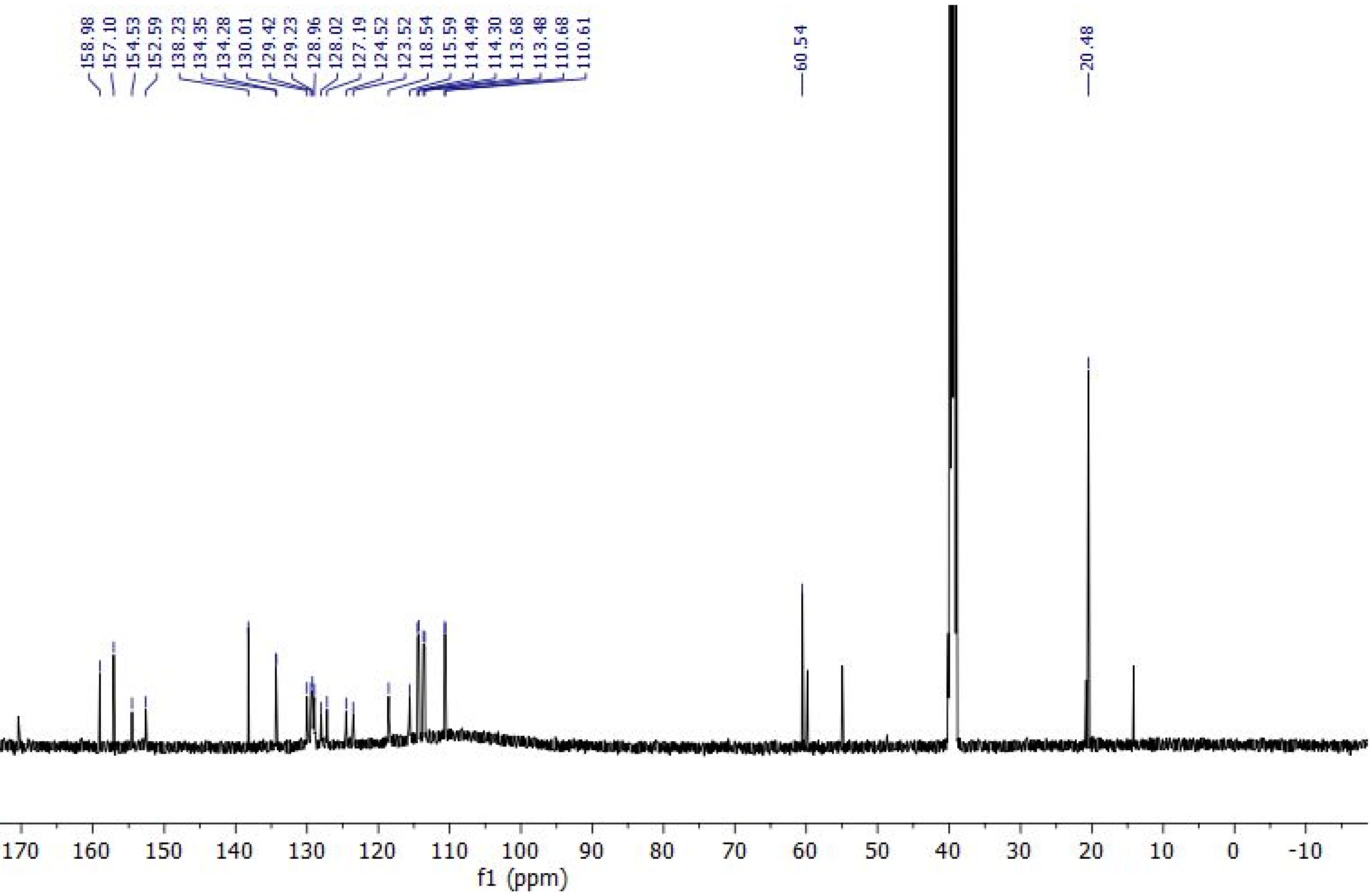

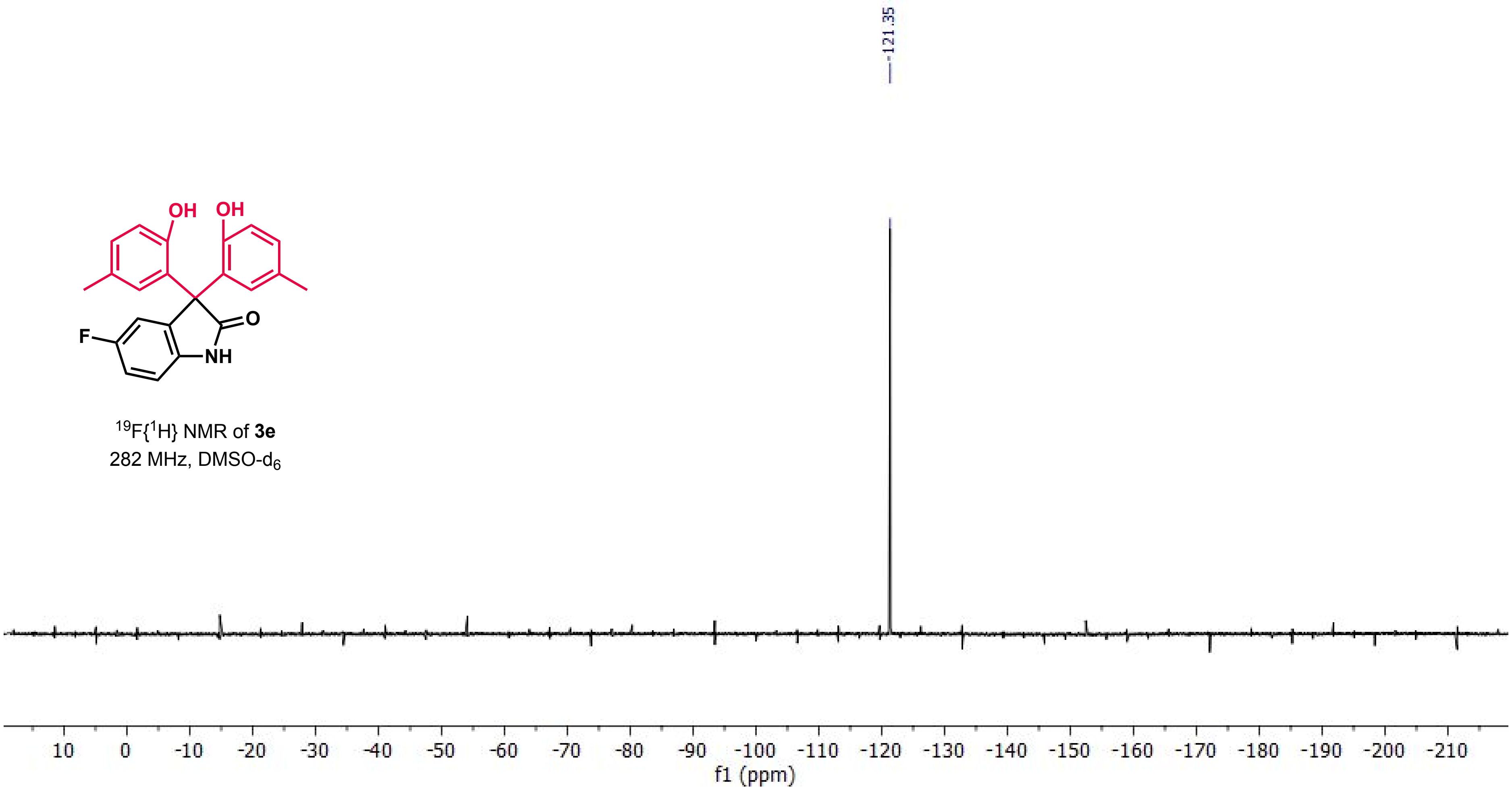

$^{19}F\{^{1}H\}$ NMR of **3e**
282 MHz, DMSO-d_6

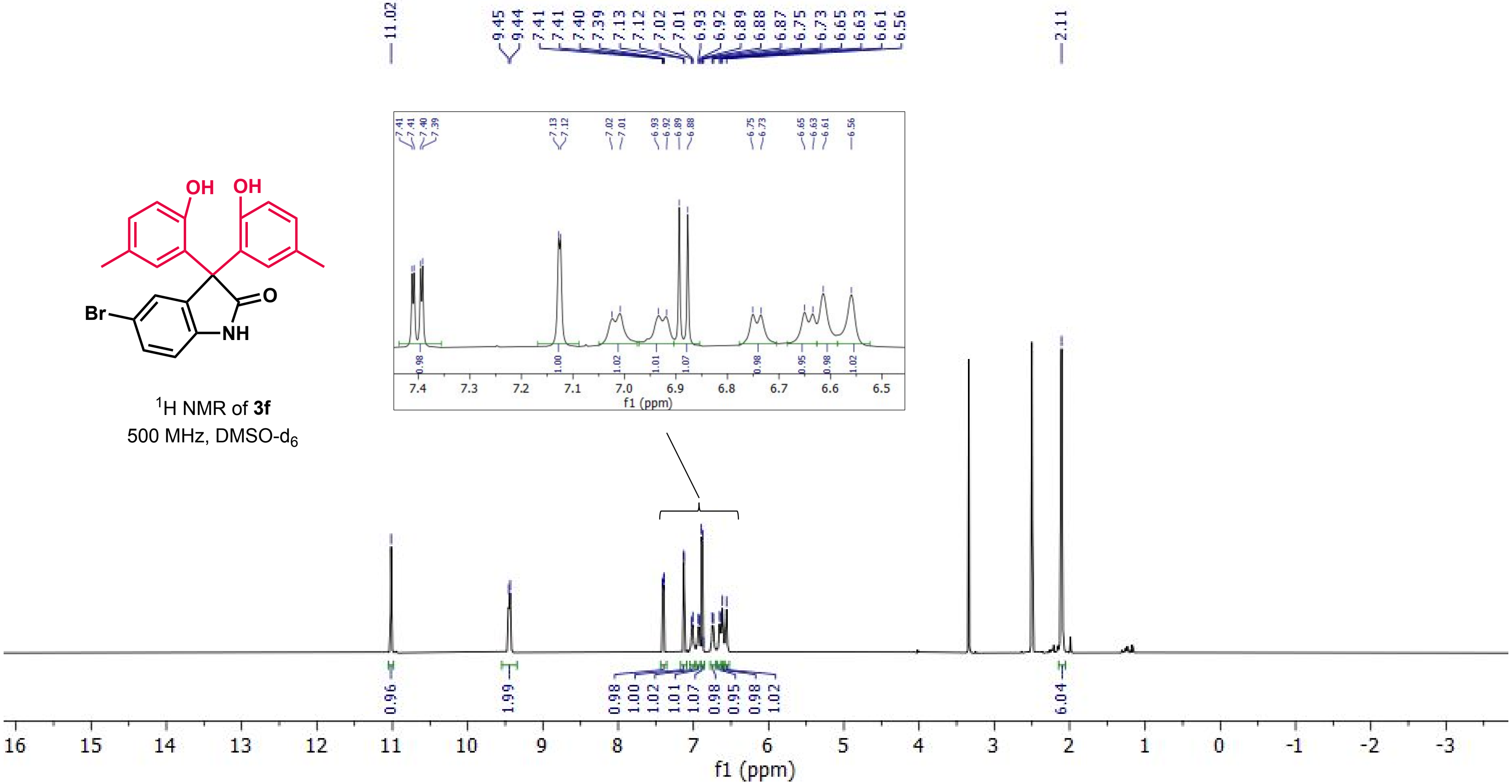

^{1}H NMR of **3f**
500 MHz, DMSO-d_6

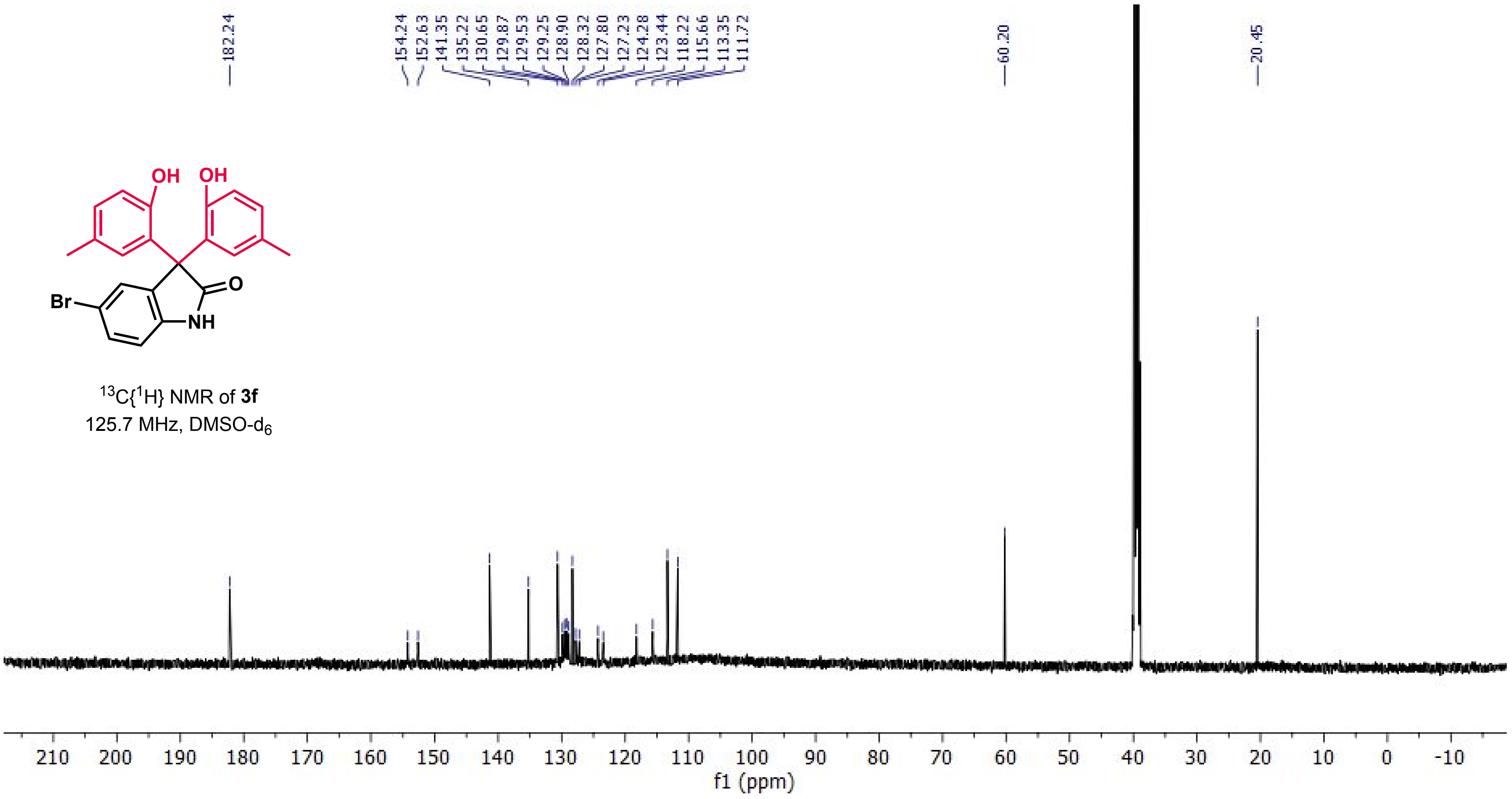
182.24
154.24
152.63
141.35
135.22
130.65
129.87
129.53
129.25
128.90
128.32
127.80
127.23
124.28
123.44
118.22
115.66
113.35
111.72
60.20
20.45
OH
OH
Br
O
NH
$^{13}C\{^{1}H\}$ NMR of **3f**
125.7 MHz, DMSO-d_6
210 200 190 180 170 160 150 140 130 120 110 100 90 80 70 60 50 40 30 20 10 0 -10
f1 (ppm)

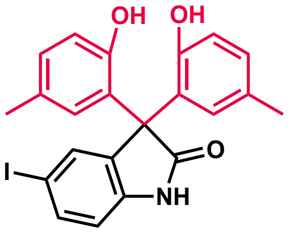

^{1}H NMR of **3g**
500 MHz, DMSO-d_6

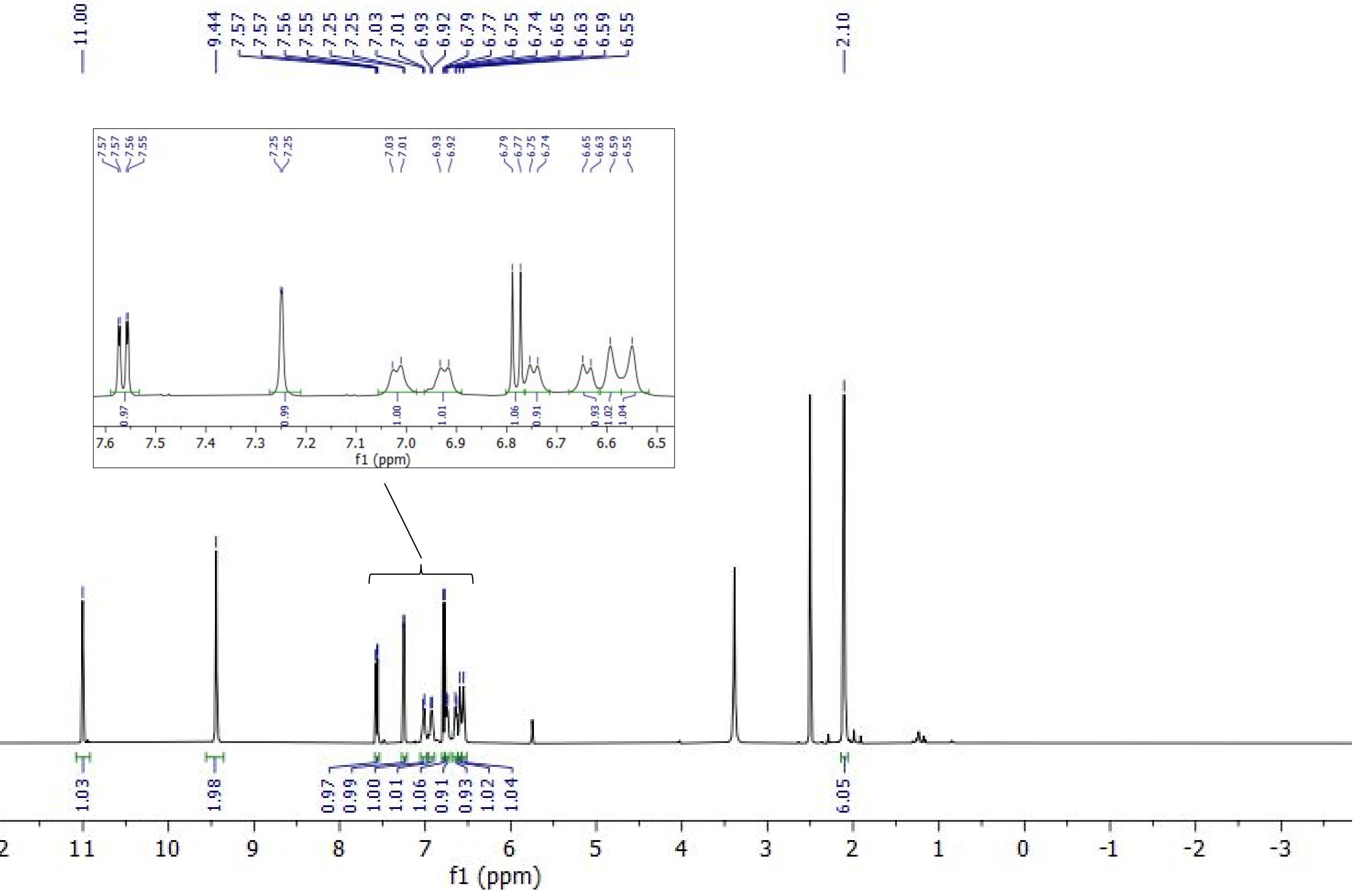

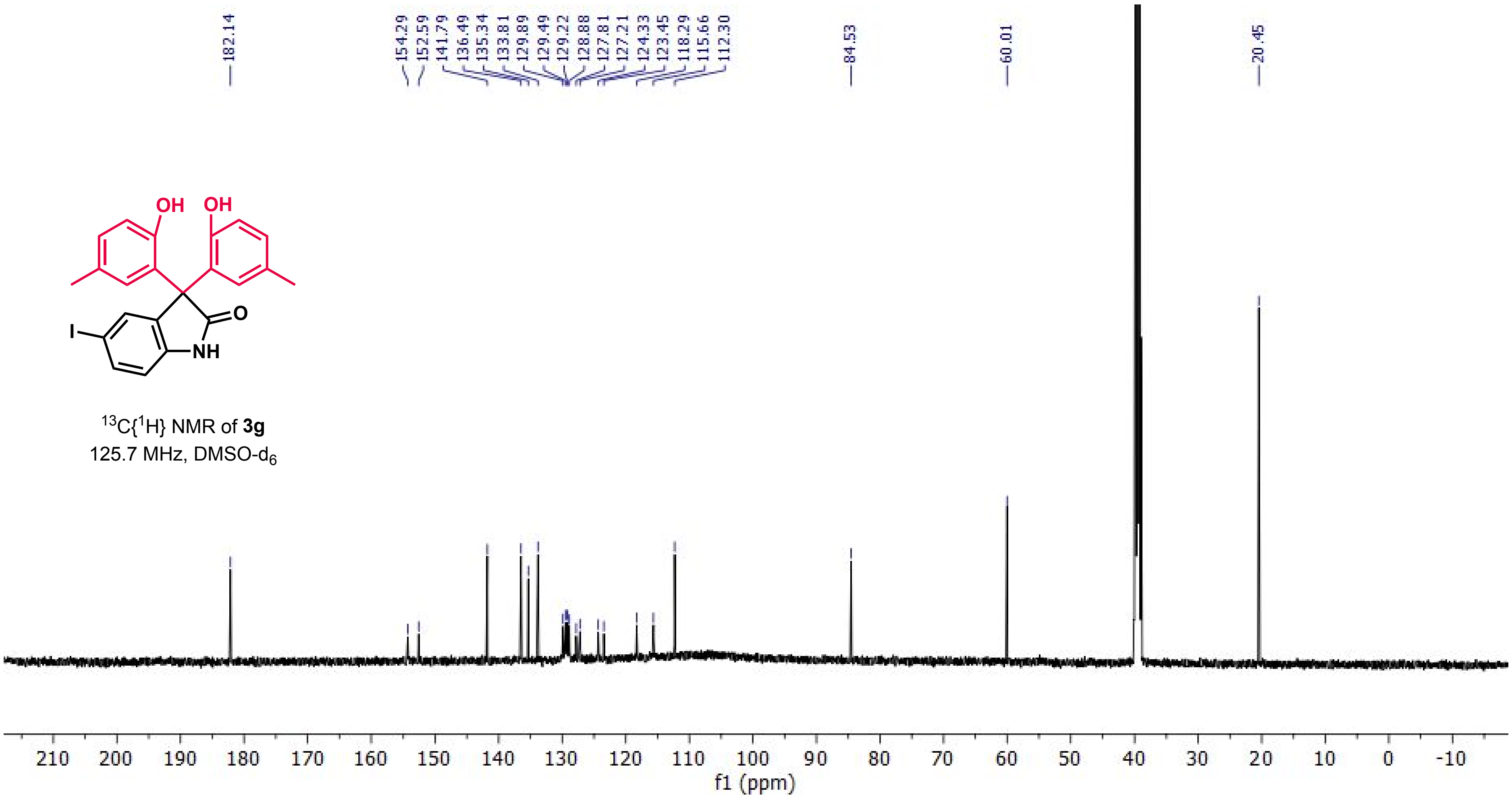

$^{13}C\{^1H\}$ NMR of **3g**
125.7 MHz, DMSO-d_6

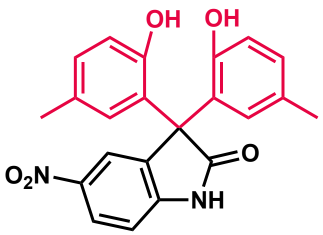

^{1}H NMR of **3h**
500 MHz, DMSO-d_6

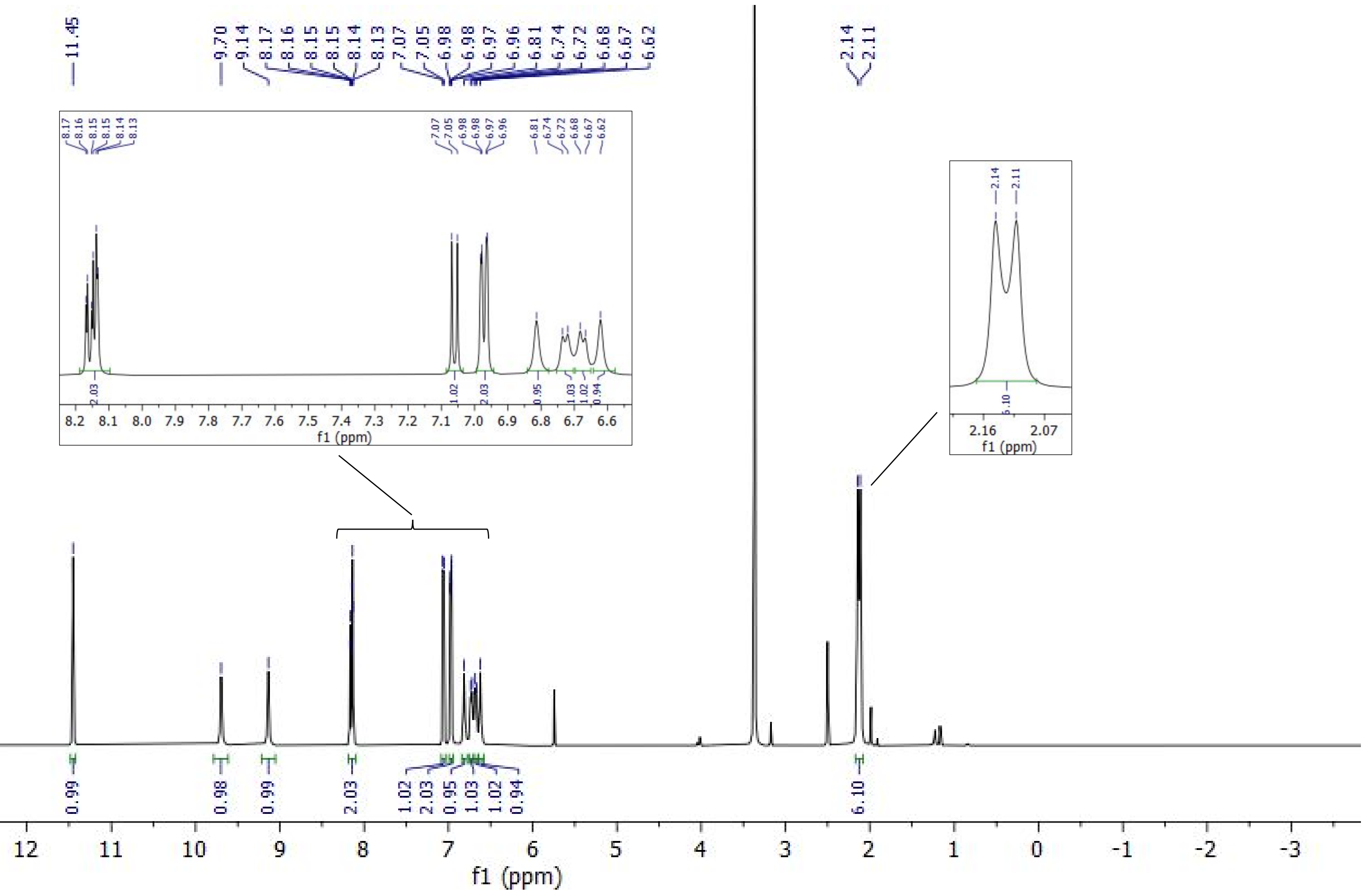

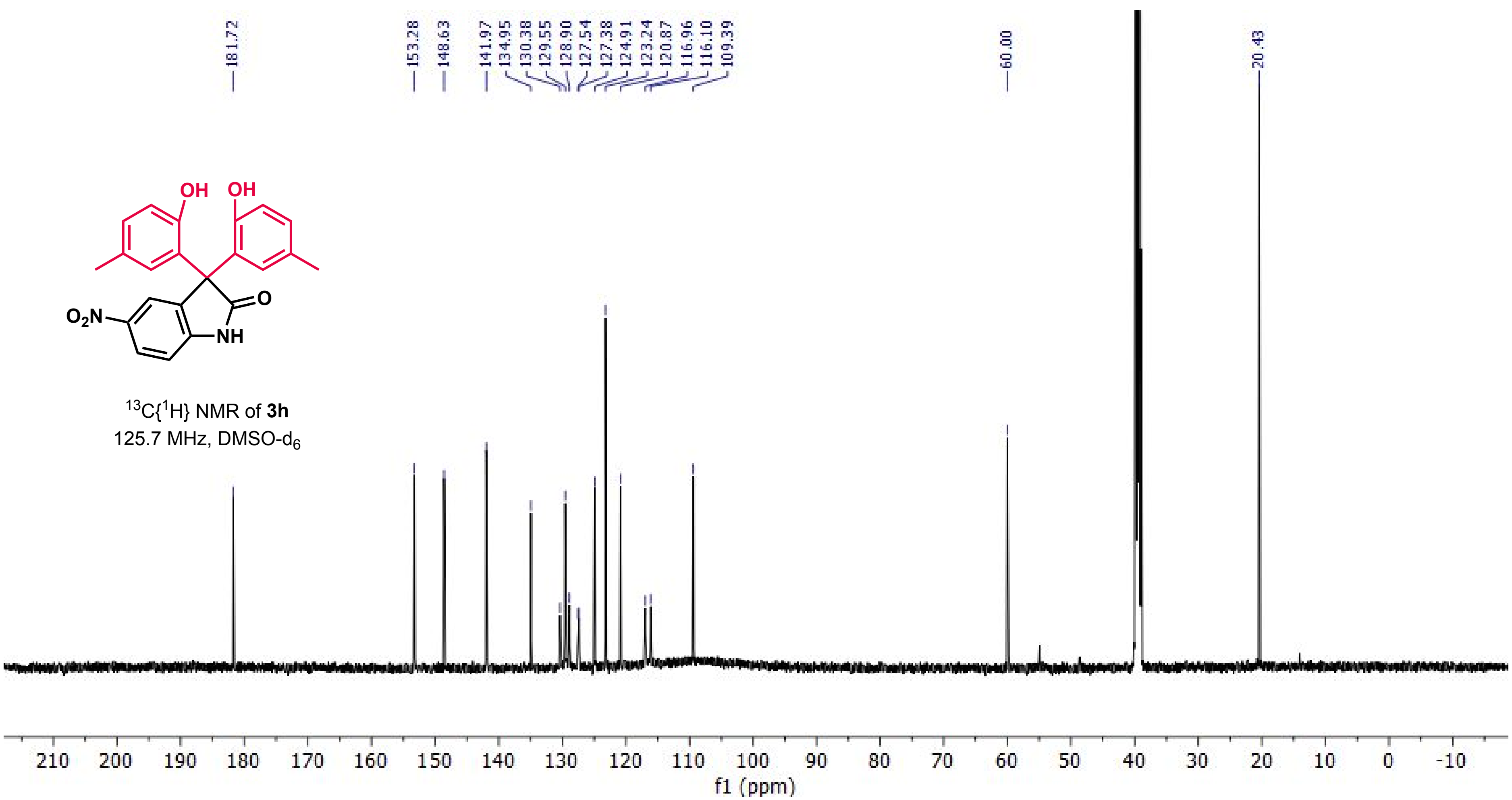

$^{13}C\{^{1}H\}$ NMR of **3h**
125.7 MHz, DMSO-d_6

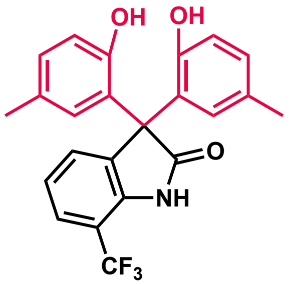

^{1}H NMR of **3i**

500 MHz, DMSO-d_6

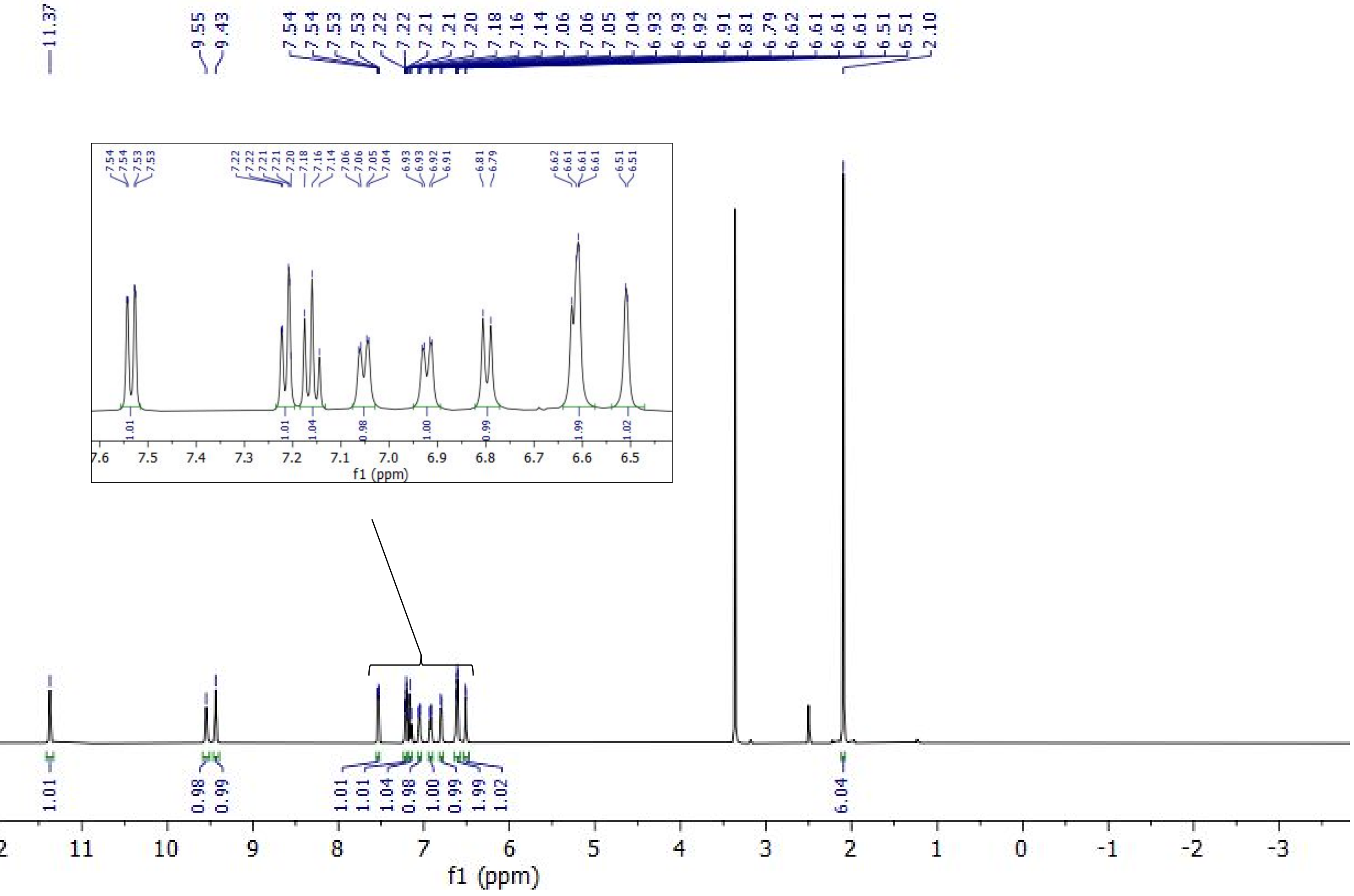

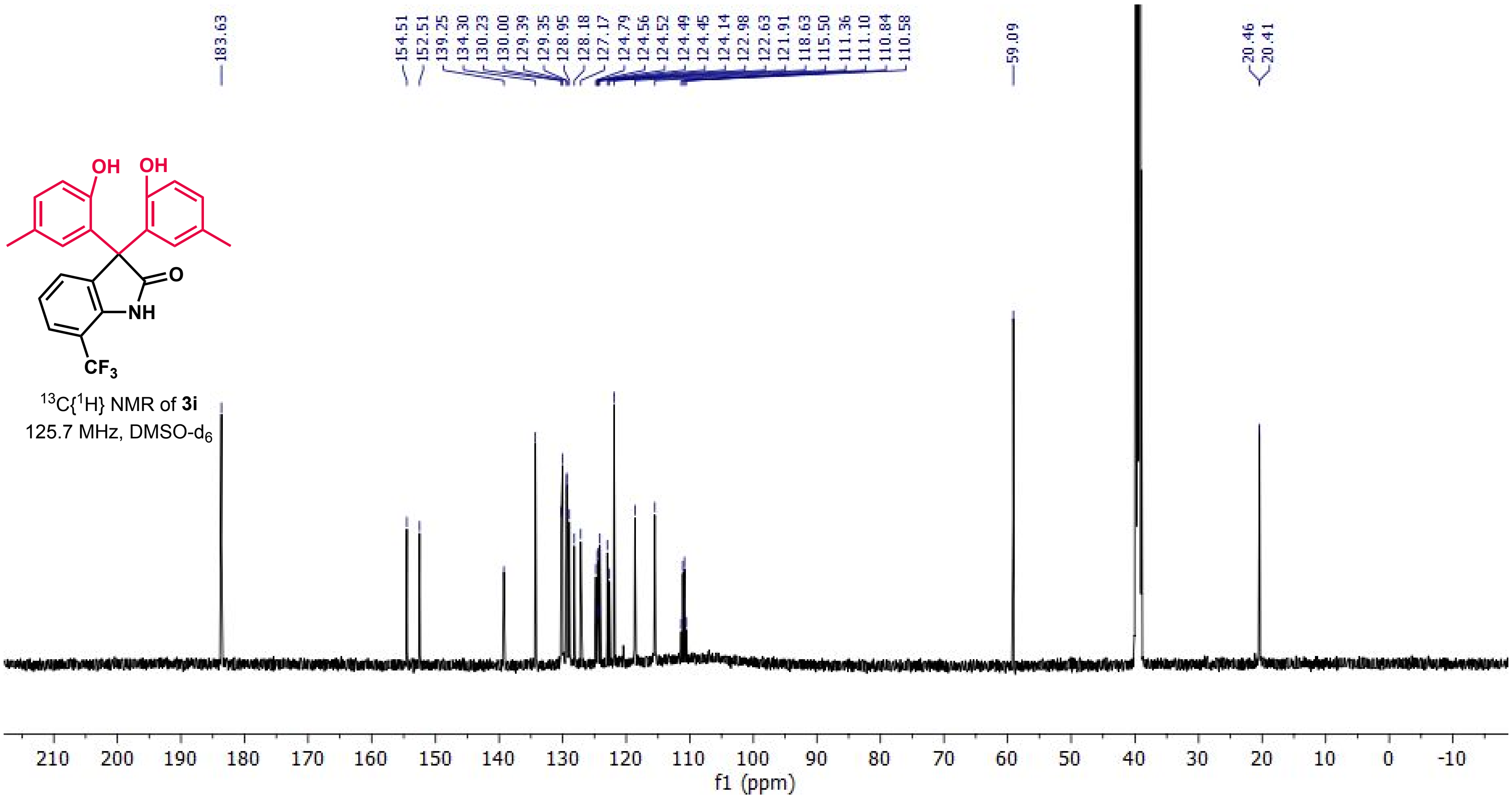
183.63
154.51
152.51
139.25
134.30
130.23
130.00
129.39
129.35
128.95
128.18
127.17
124.79
124.56
124.52
124.49
124.45
124.14
122.98
122.63
121.91
118.63
115.50
111.36
111.10
110.84
110.58
59.09
20.46
20.41
OH
OH
O
NH
CF_3
$^{13}C\{^1H\}$ NMR of **3i**
125.7 MHz, DMSO-d_6
210 200 190 180 170 160 150 140 130 120 110 100 90 80 70 60 50 40 30 20 10 0 -10
f1 (ppm)

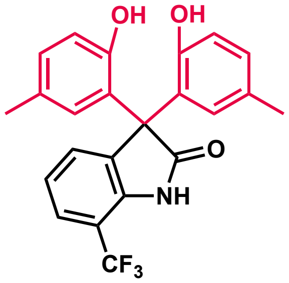

$^{19}F\{^1H\}$ NMR of **3i**
282 MHz, DMSO-d_6

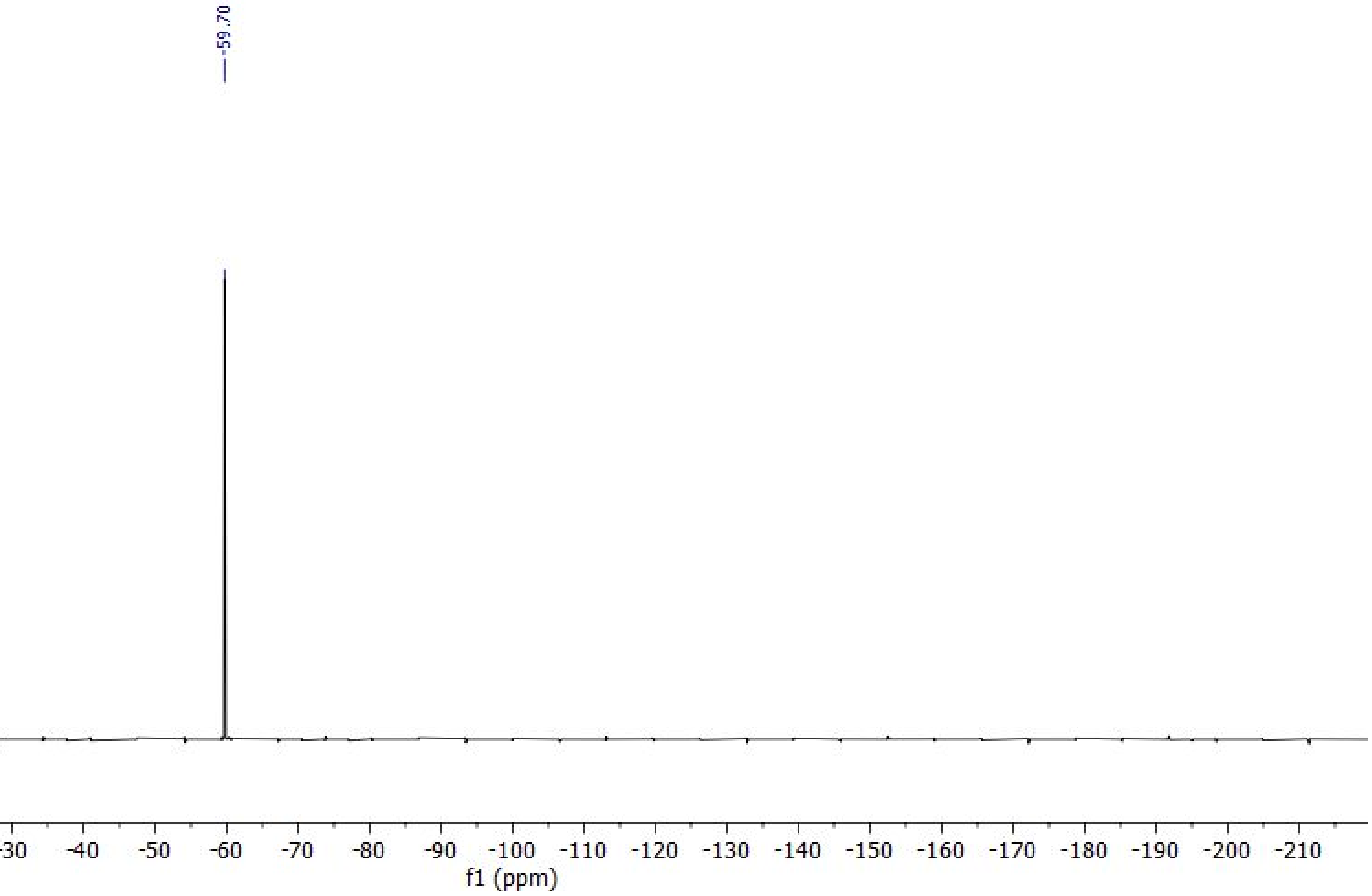

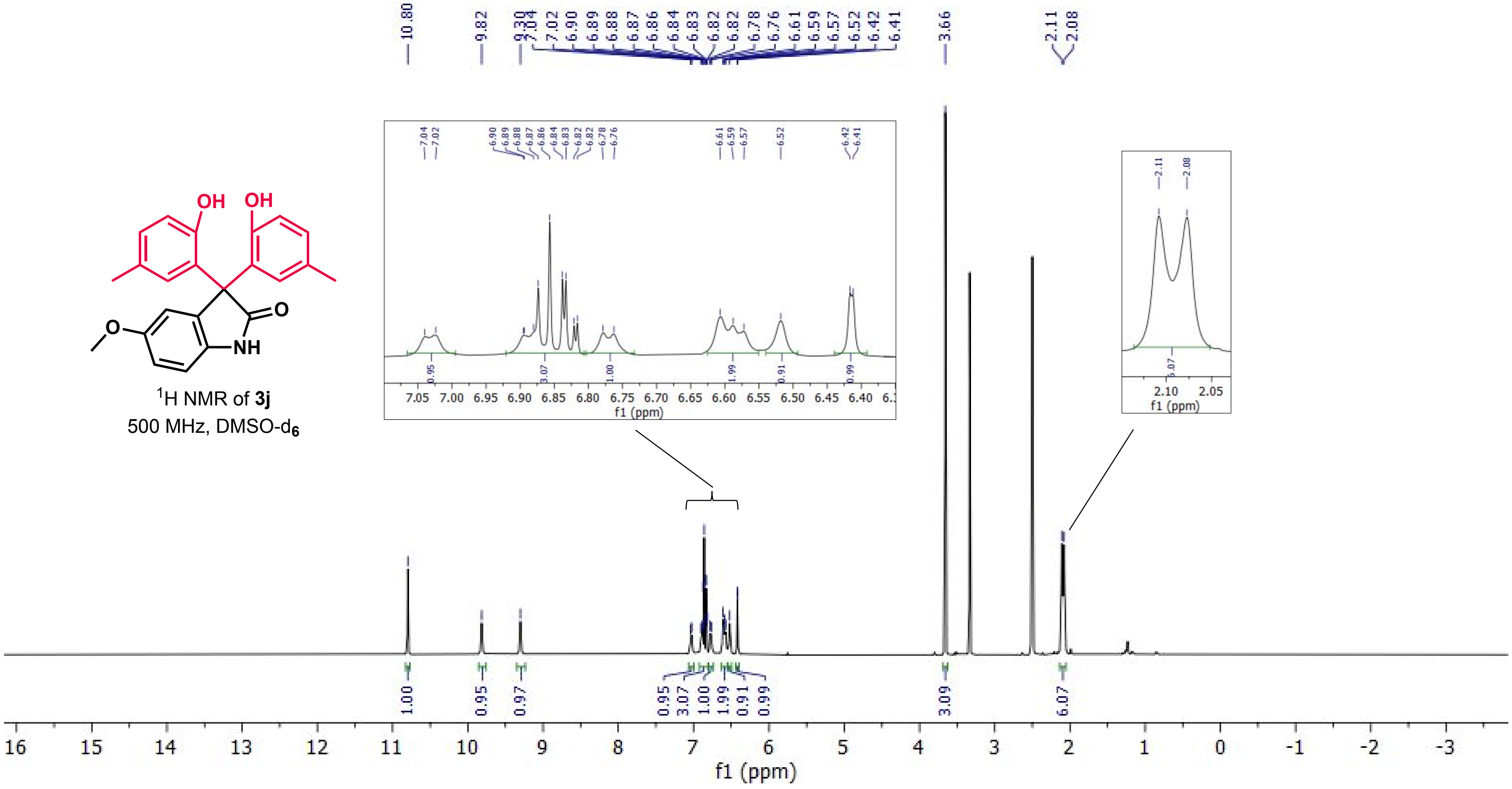

^{1}H NMR of **3j**
500 MHz, DMSO-d$_6$

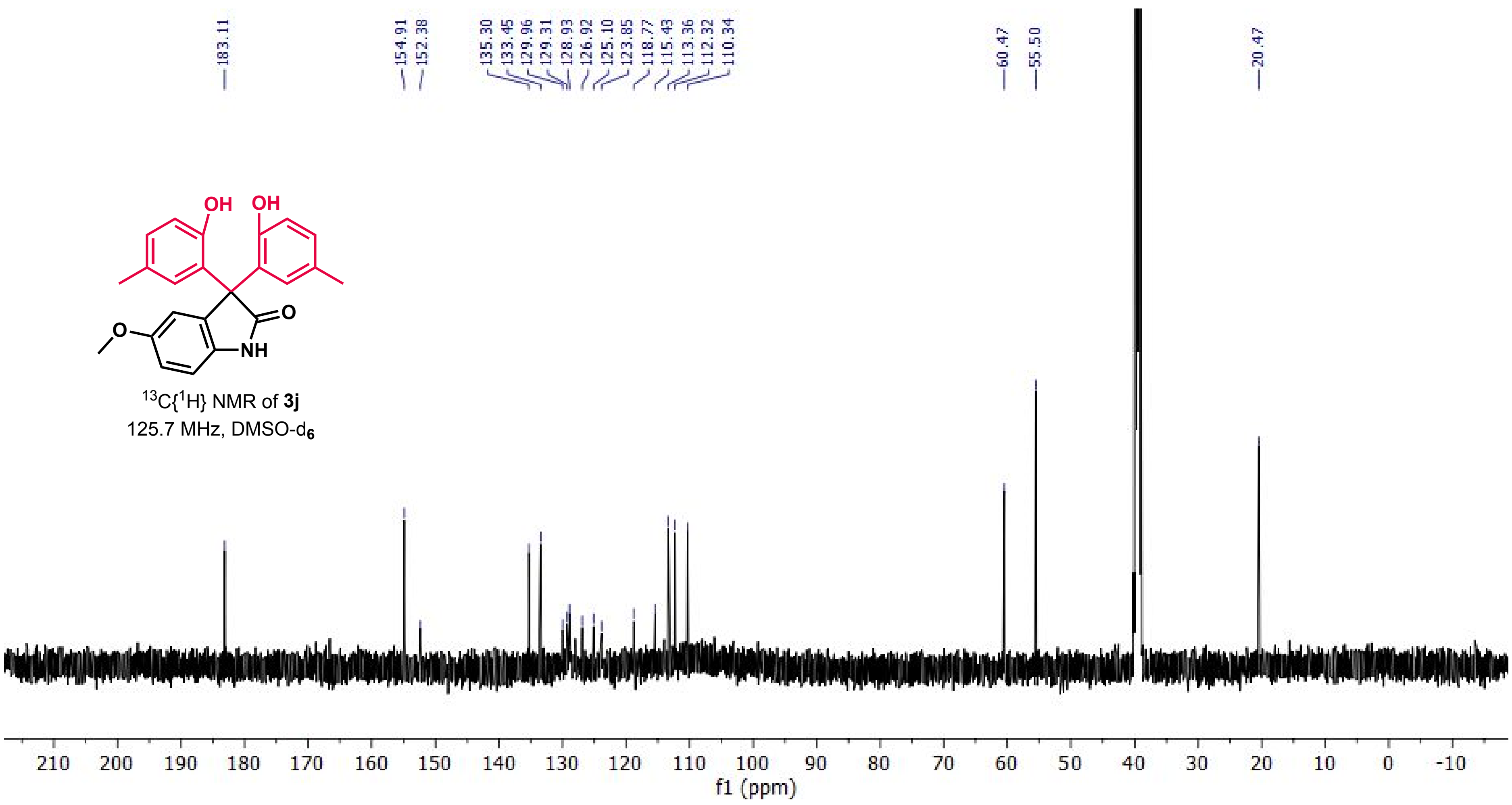

$^{13}C\{^{1}H\}$ NMR of **3j**
125.7 MHz, DMSO-d_6

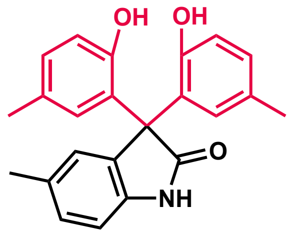

^{1}H NMR of **3k**

500 MHz, DMSO-d_6

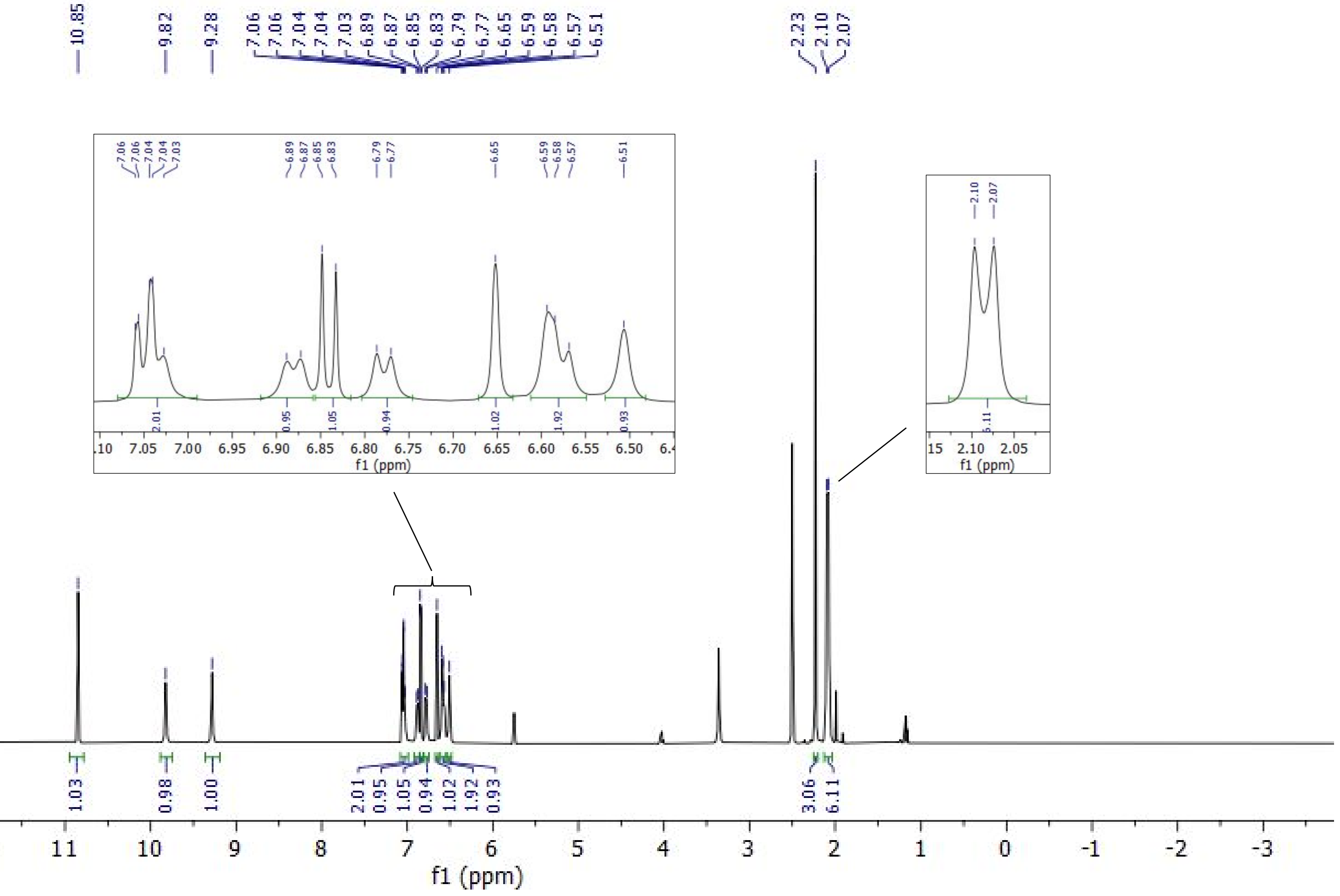

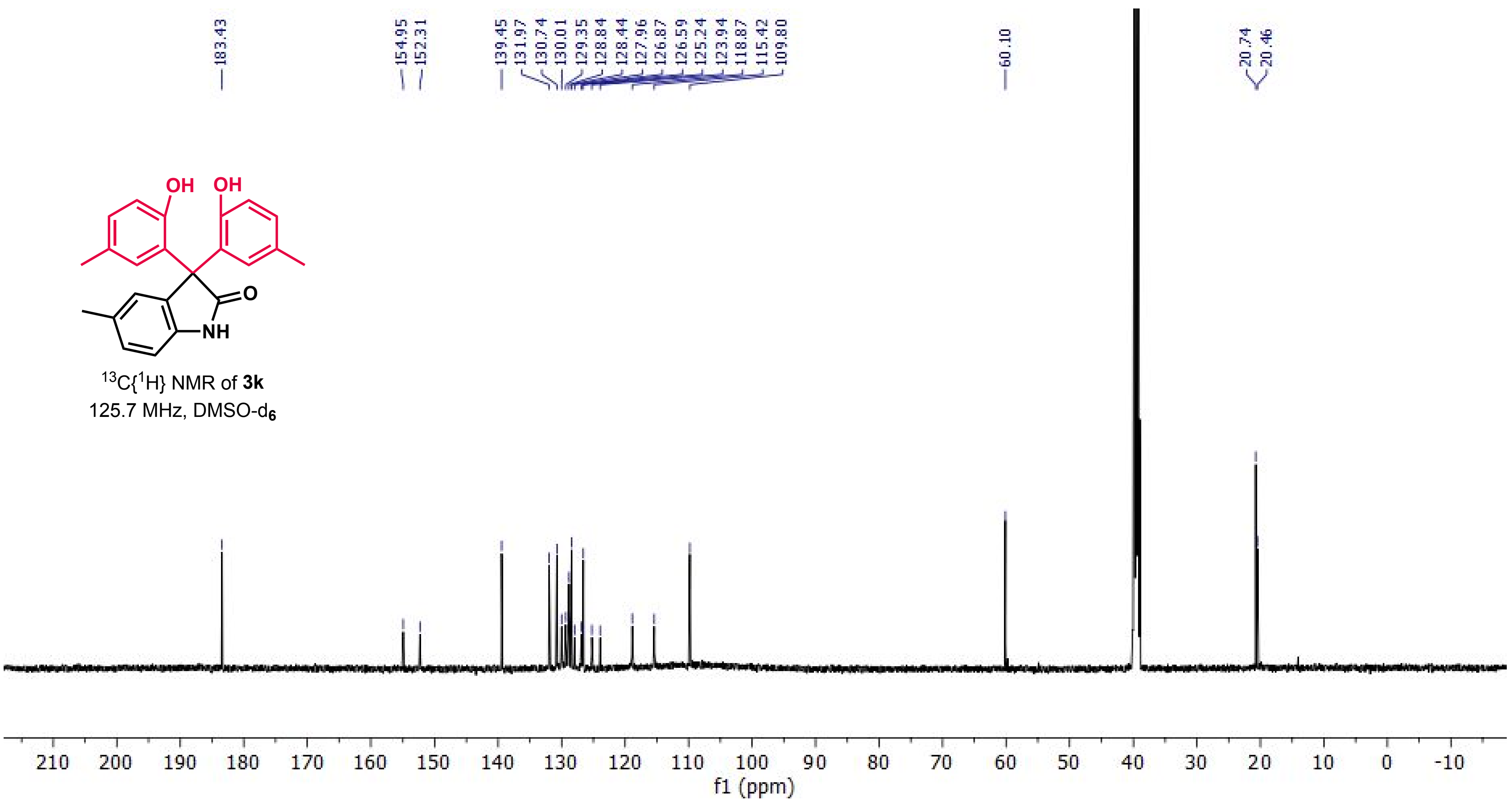

$^{13}C\{^1H\}$ NMR of **3k**
125.7 MHz, DMSO-d_6

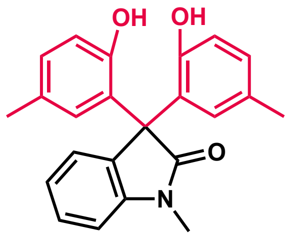

^{1}H NMR of **3l**
500 MHz, DMSO-d_6

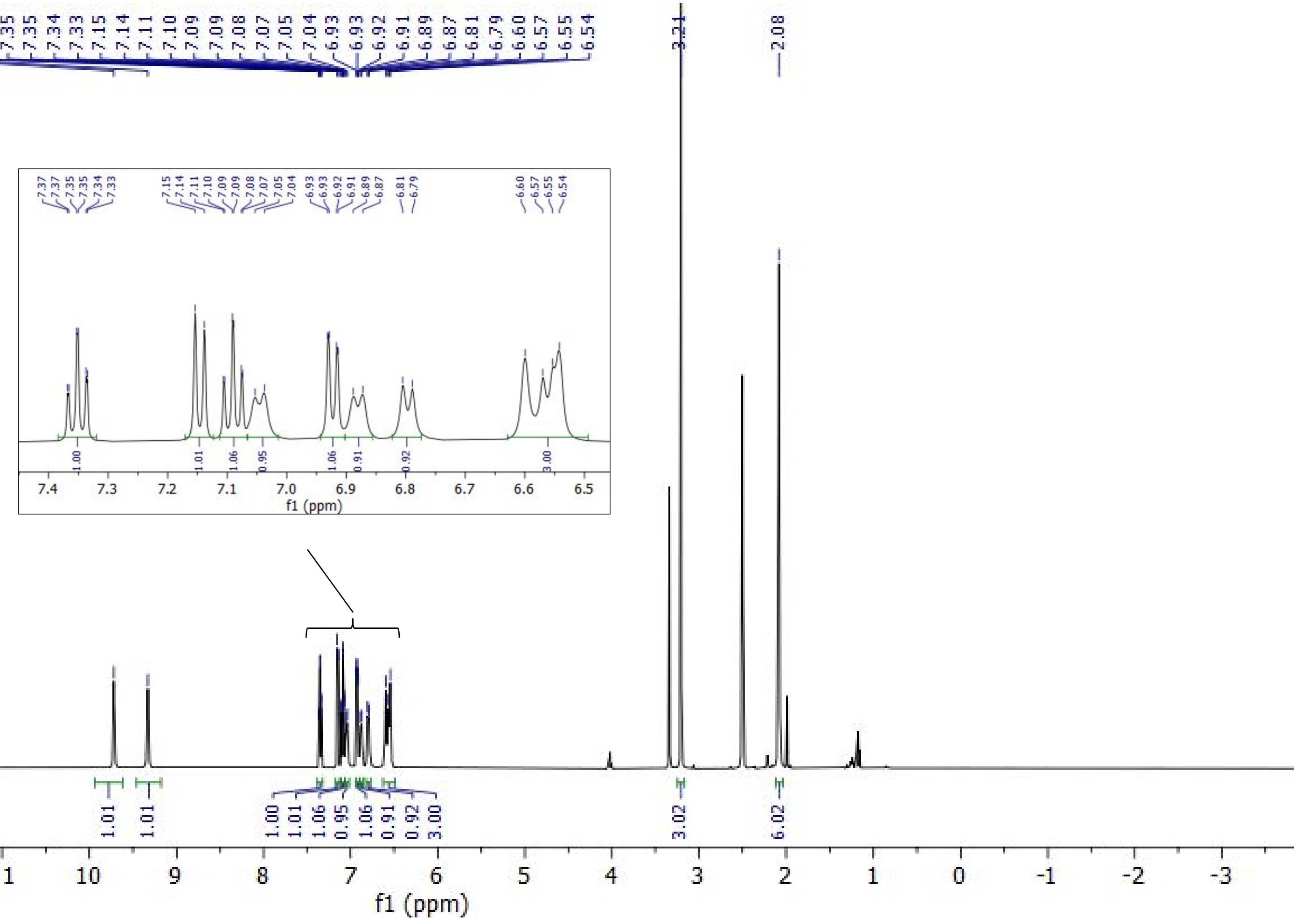

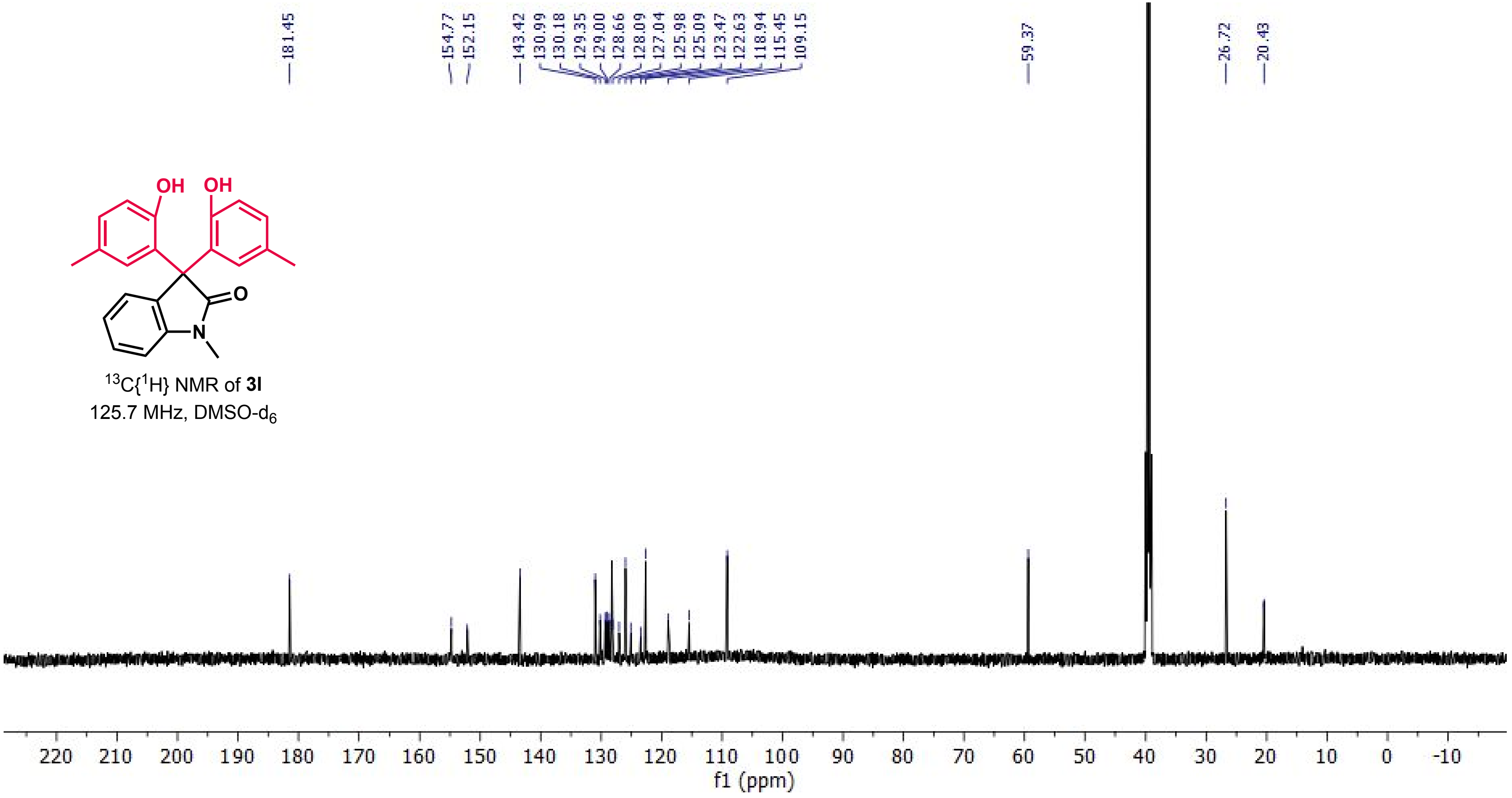

181.45
154.77
152.15
143.42
130.99
130.18
129.35
129.00
128.66
128.09
127.04
125.98
125.09
123.47
122.63
118.94
115.45
109.15
59.37
26.72
20.43
OH
OH
O
N
$^{13}C\{^{1}H\}$ NMR of **3l**
125.7 MHz, DMSO-d_6
220 210 200 190 180 170 160 150 140 130 120 110 100 90 80 70 60 50 40 30 20 10 0 -10
f1 (ppm)

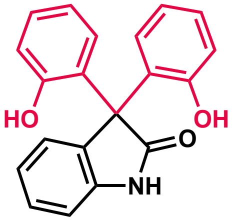

^{1}H NMR of **3n-l**
500 MHz, DMSO-d_6

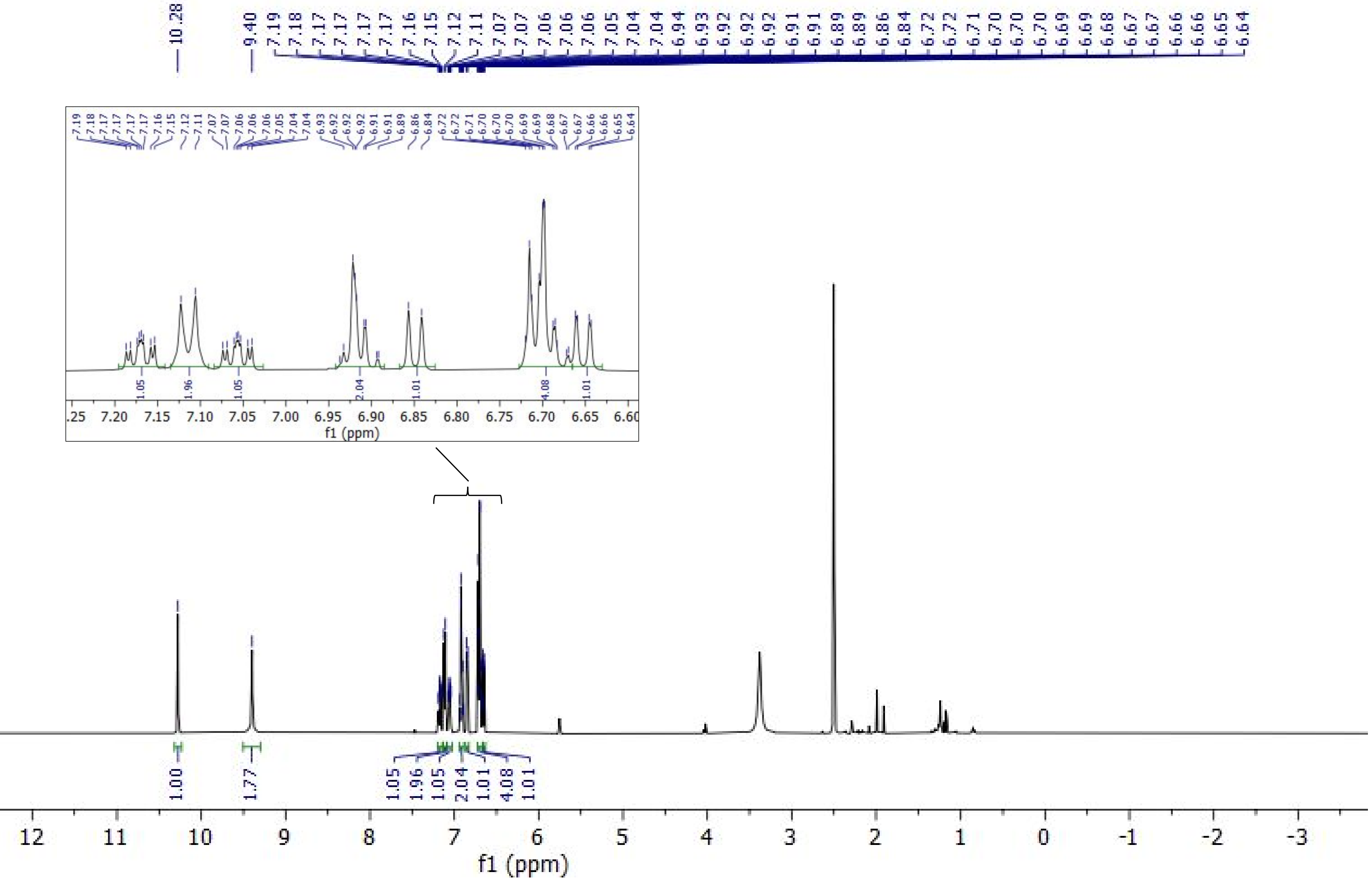

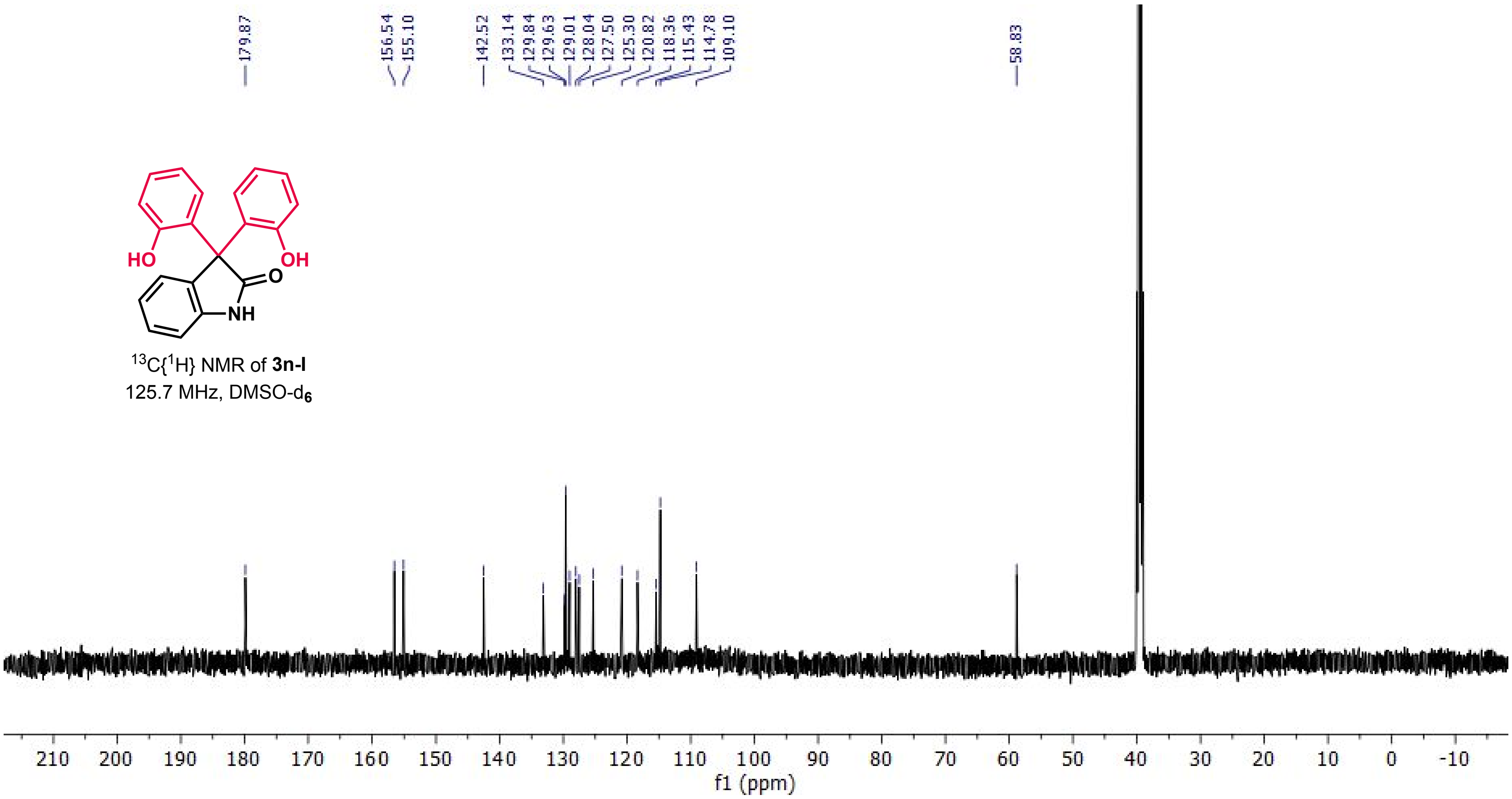

$^{13}C\{^{1}H\}$ NMR of **3n-I**
125.7 MHz, DMSO-d$_6$

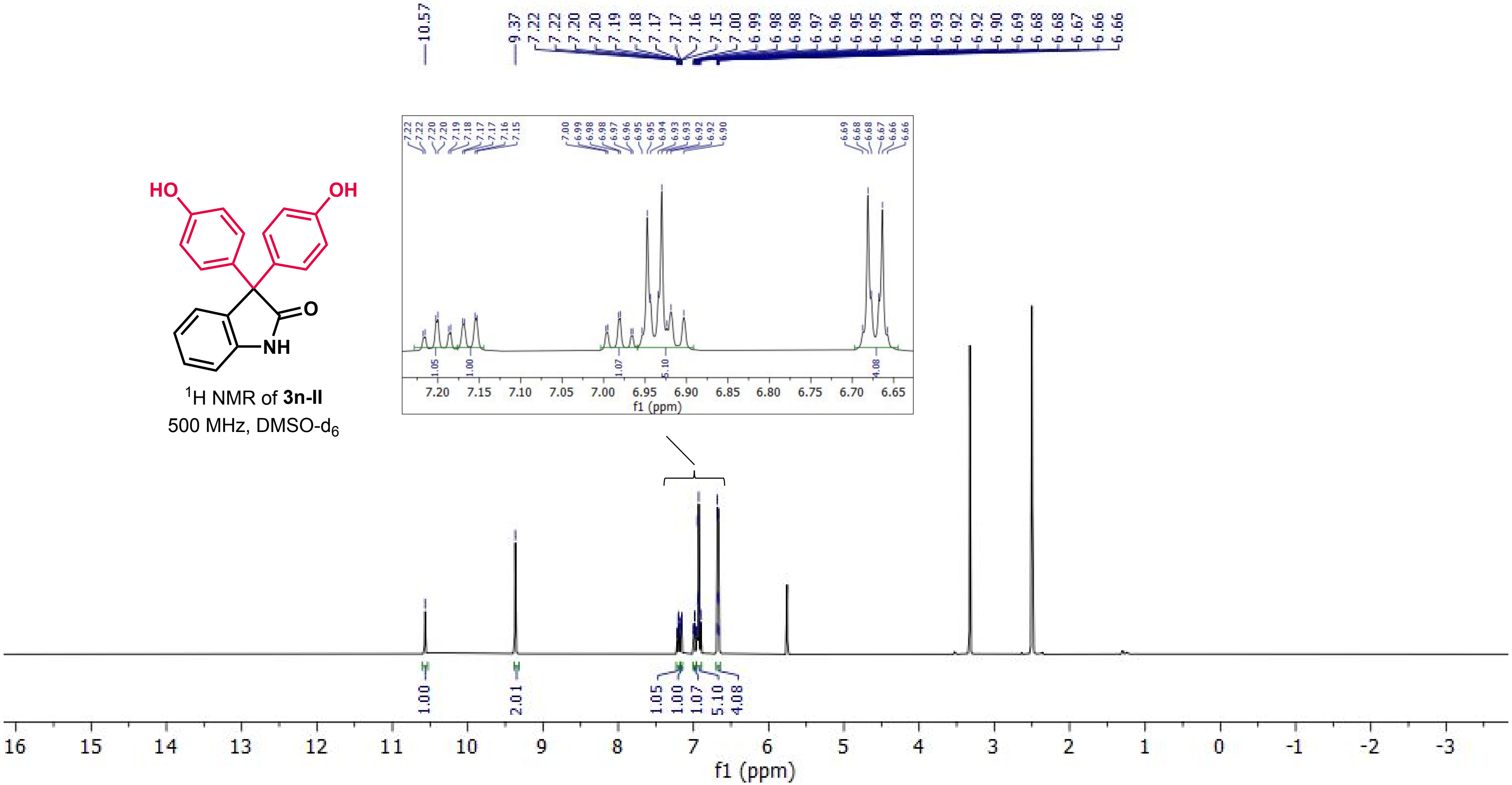

^{1}H NMR of **3n-II**
500 MHz, DMSO-d_6

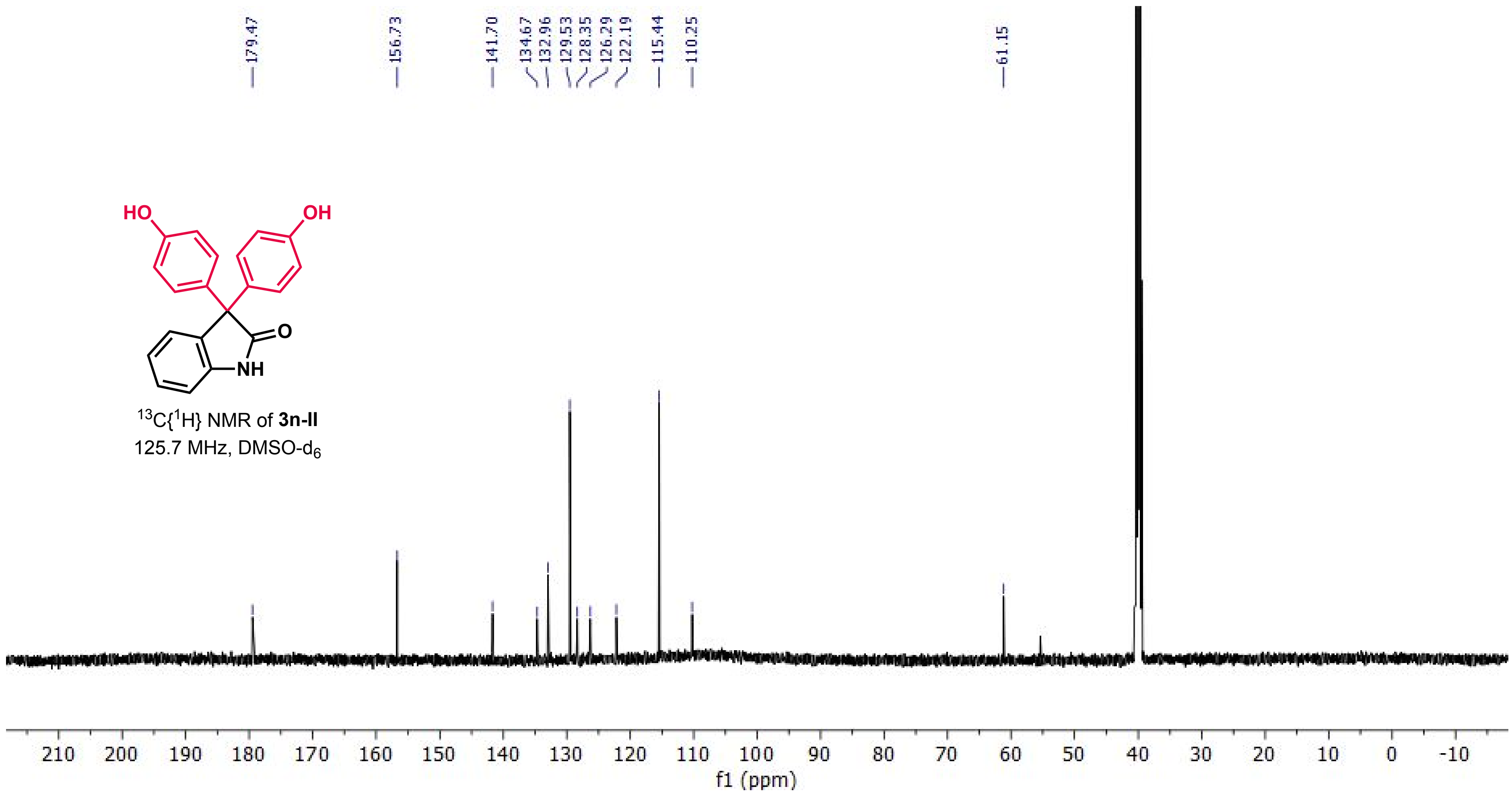
179.47
156.73
141.70
134.67
132.96
129.53
128.35
126.29
122.19
115.44
110.25
61.15
HO
OH
O
NH
$^{13}C\{^1H\}$ NMR of **3n-II**
125.7 MHz, DMSO-d_6
210 200 190 180 170 160 150 140 130 120 110 100 90 80 70 60 50 40 30 20 10 0 -10
f1 (ppm)

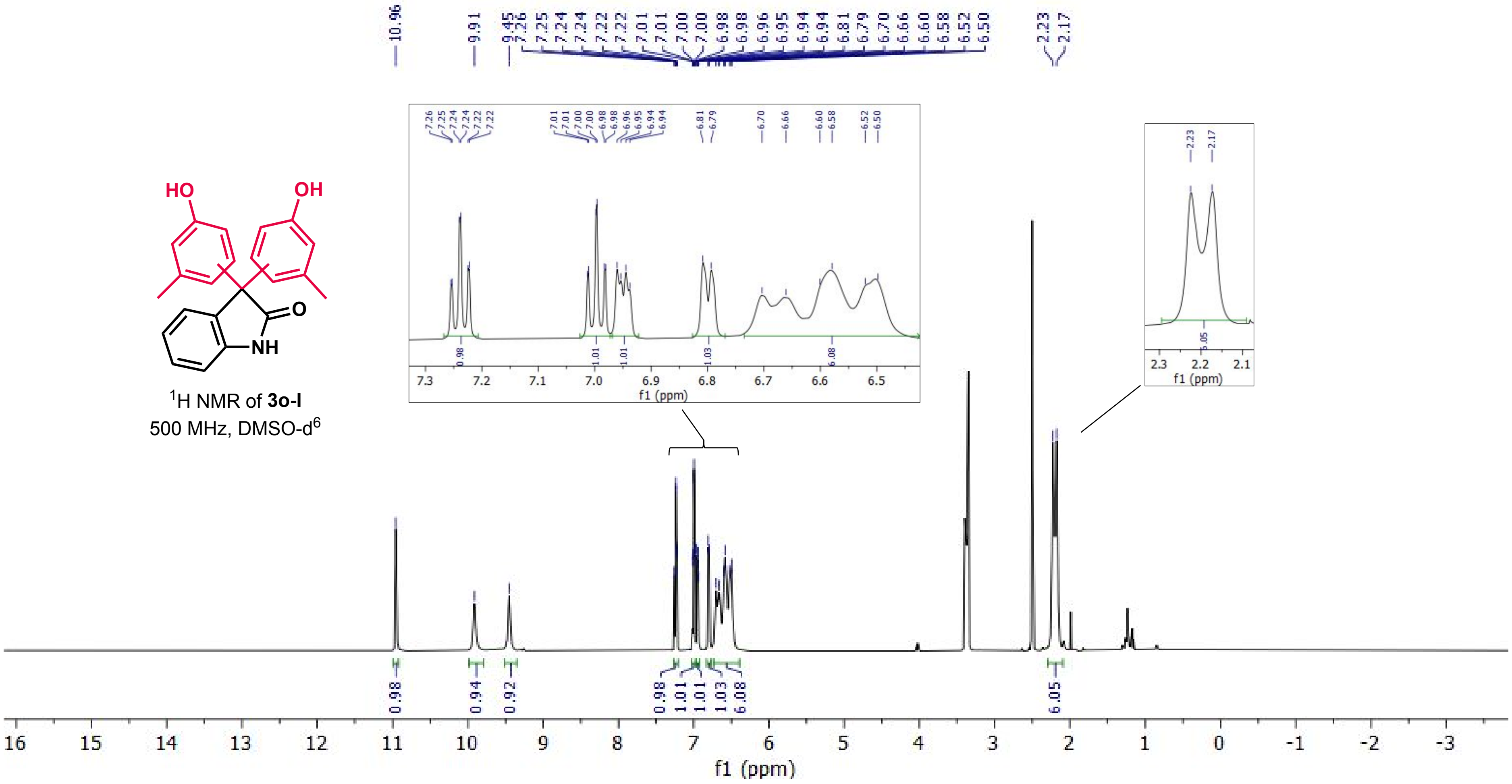
HO
OH
O
NH
^{1}H NMR of **3o-I**
500 MHz, DMSO-d6
10.96
9.91
9.45
2.23
2.17
0.98
0.94
0.92
1.01
1.03
6.08
6.05
f1 (ppm)

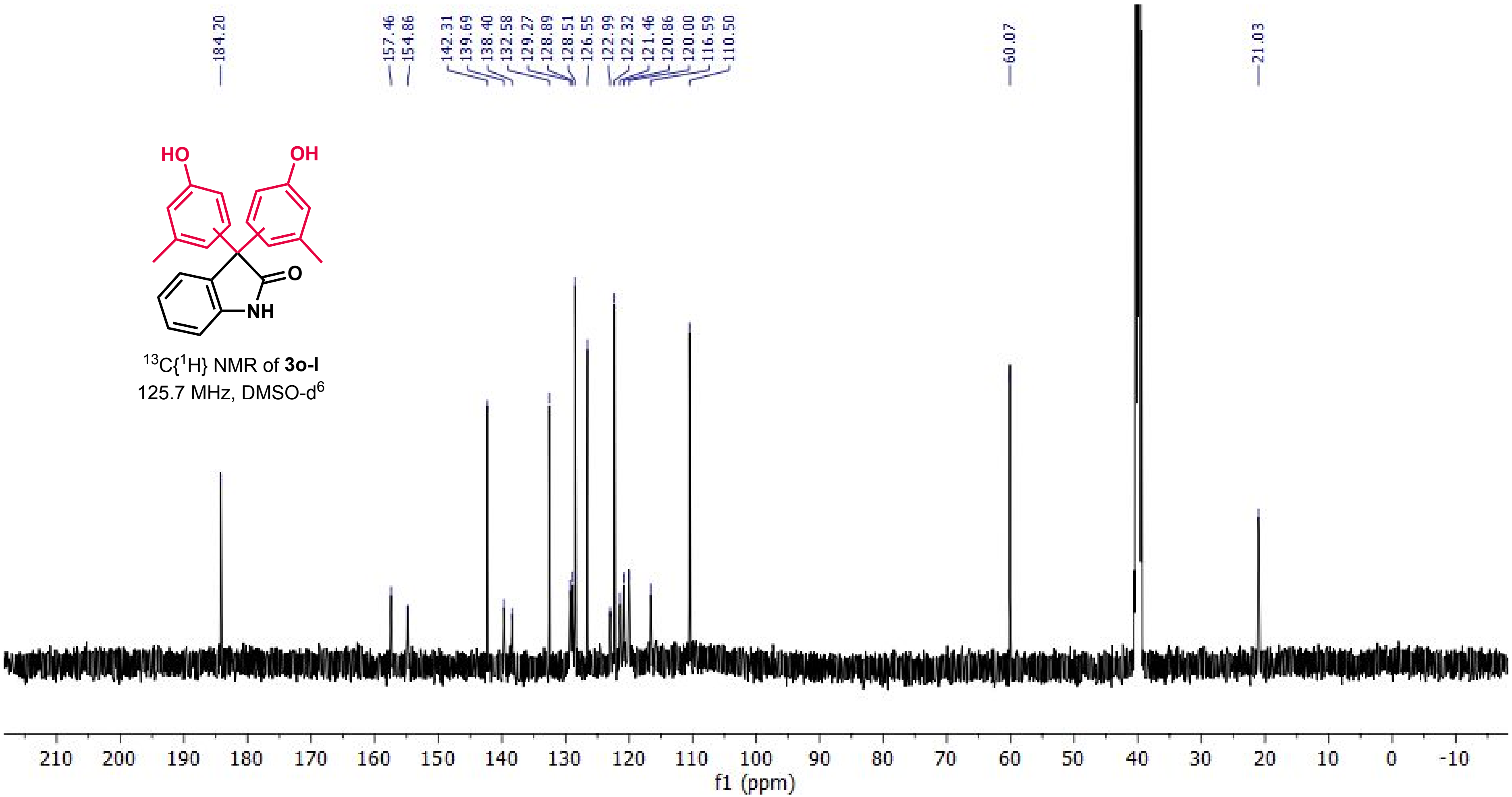
184.20
157.46
154.86
142.31
139.69
138.40
132.58
129.27
128.89
128.51
126.55
122.99
122.32
121.46
120.86
120.00
116.59
110.50
60.07
21.03
HO
OH
O
NH
^{13}C{^{1}H} NMR of **3o-l**
125.7 MHz, DMSO-d^{6}
210
200
190
180
170
160
150
140
130
120
110
100
90
80
70
60
50
40
30
20
10
0
-10
f1 (ppm)

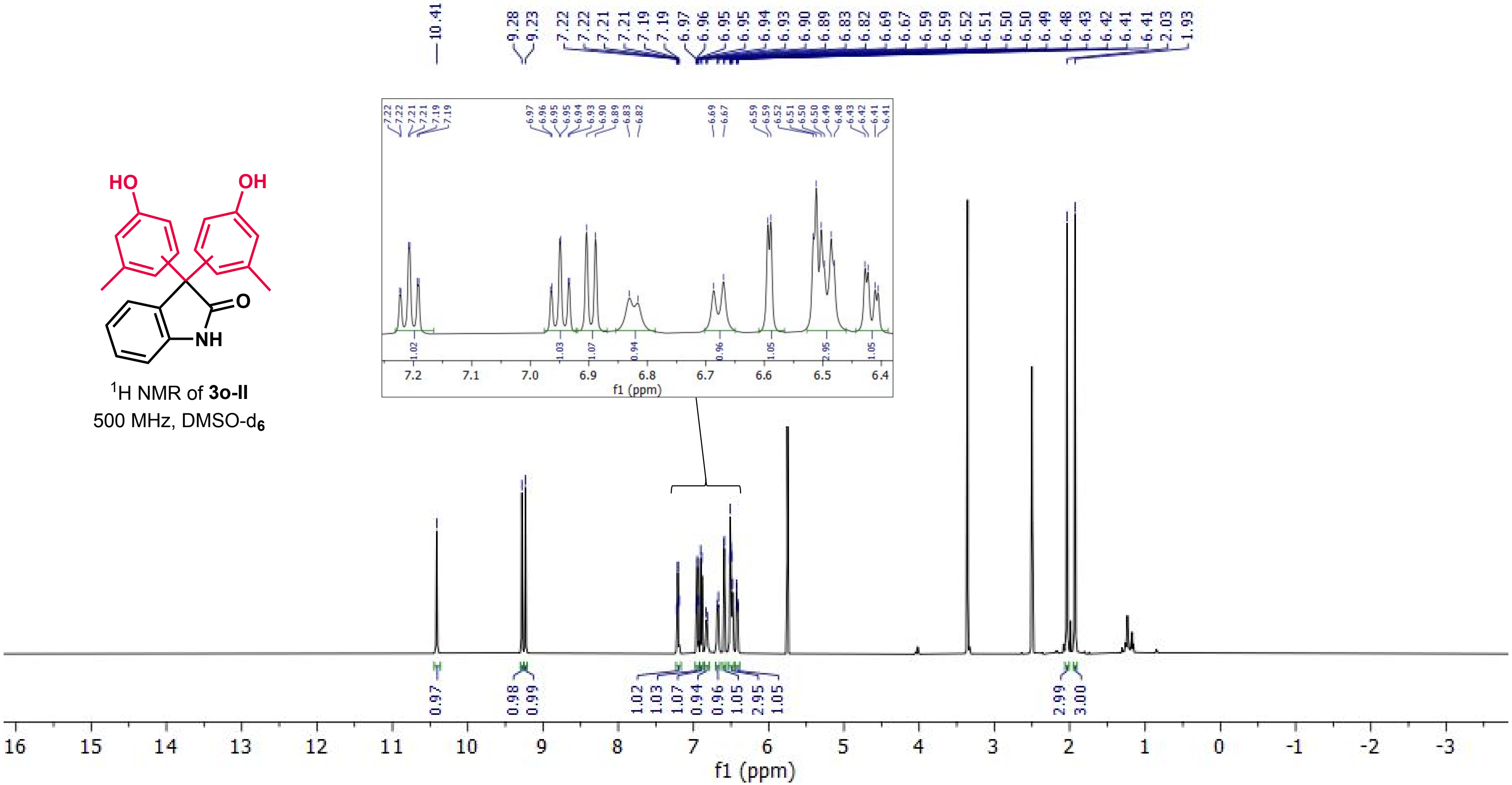

^{1}H NMR of **3o-II**
500 MHz, DMSO-d$_6$

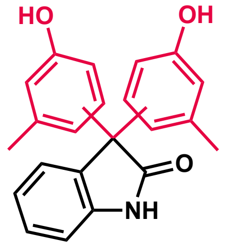

$^{13}C\{^1H\}$ NMR of **3o-II**
125.7 MHz, DMSO-d$_6$

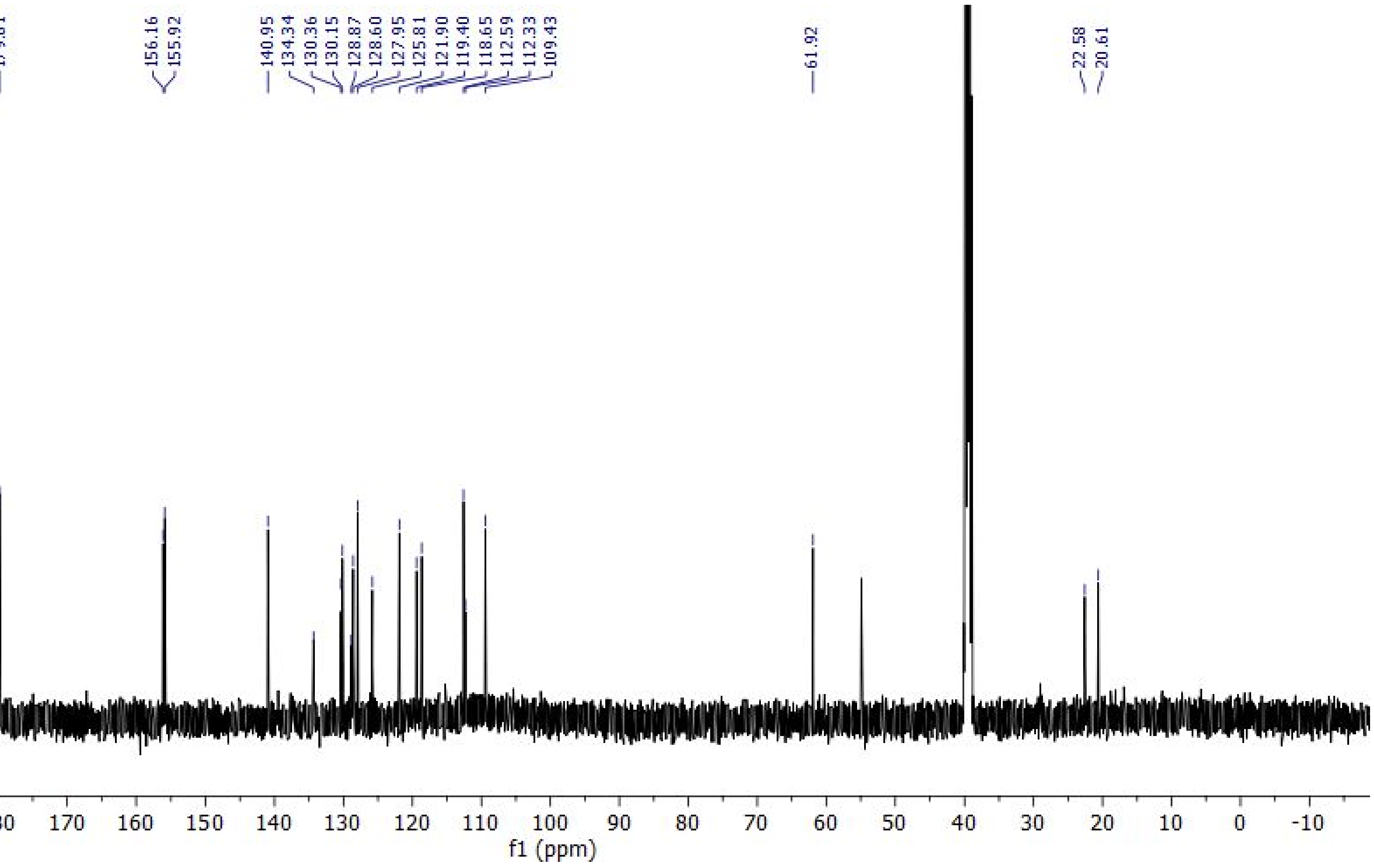

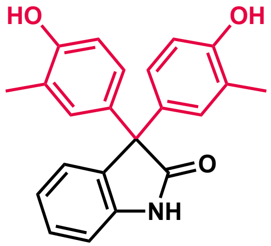

^{1}H NMR of **3p**
500 MHz, DMSO-d_6

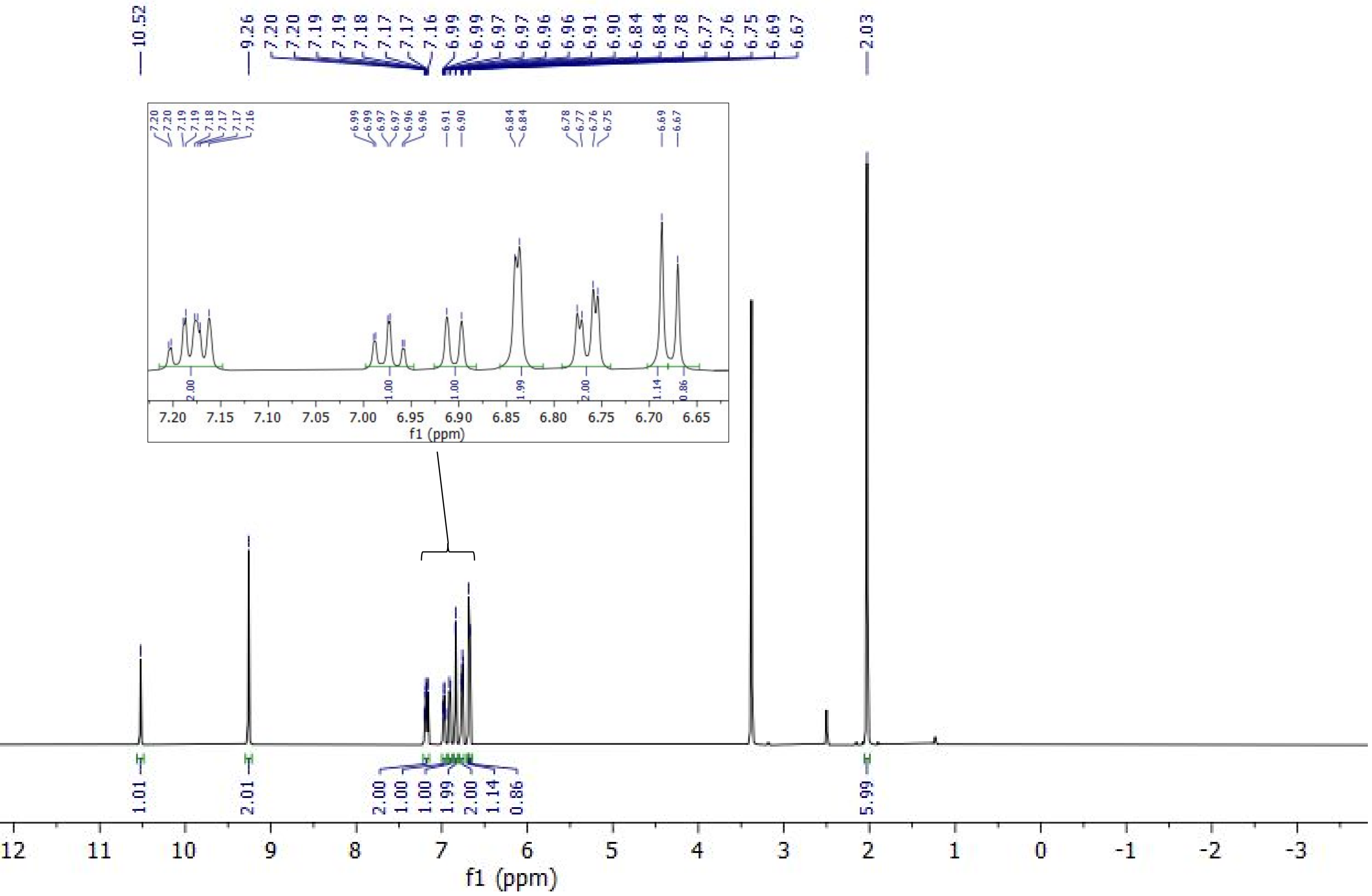

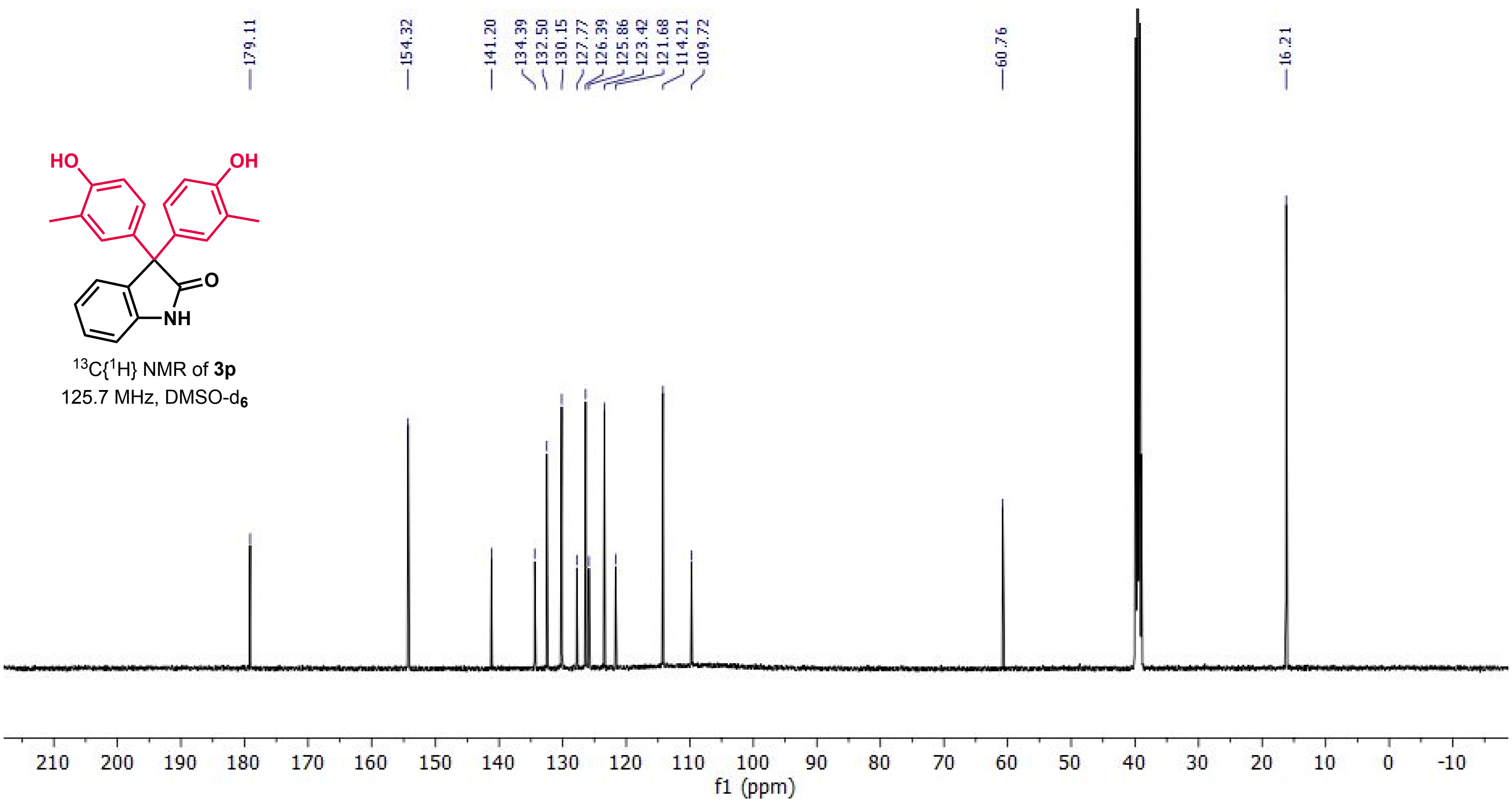

$^{13}C\{^1H\}$ NMR of **3p**
125.7 MHz, DMSO-d_6

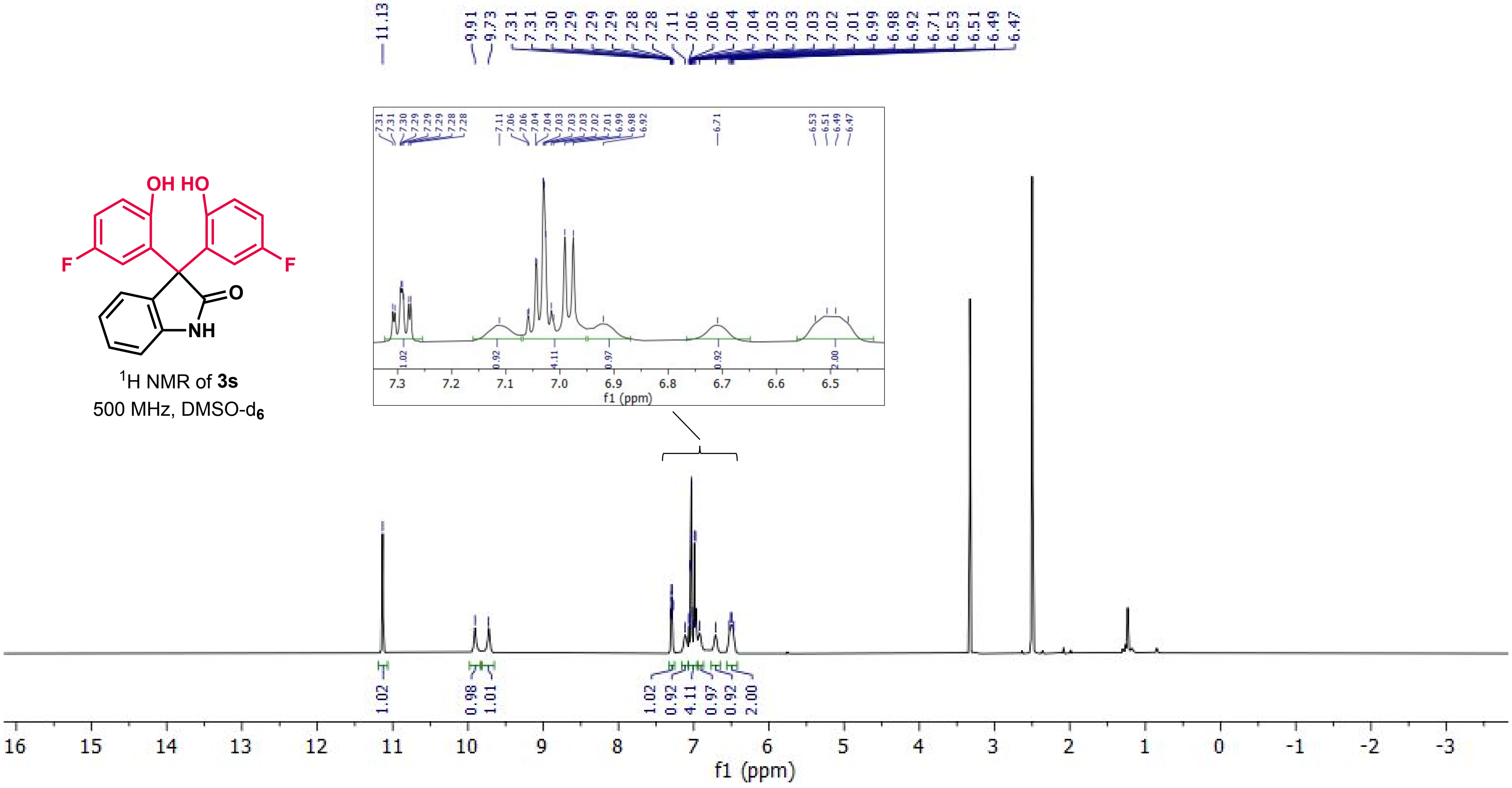

^{1}H NMR of **3s**
500 MHz, DMSO-d_6

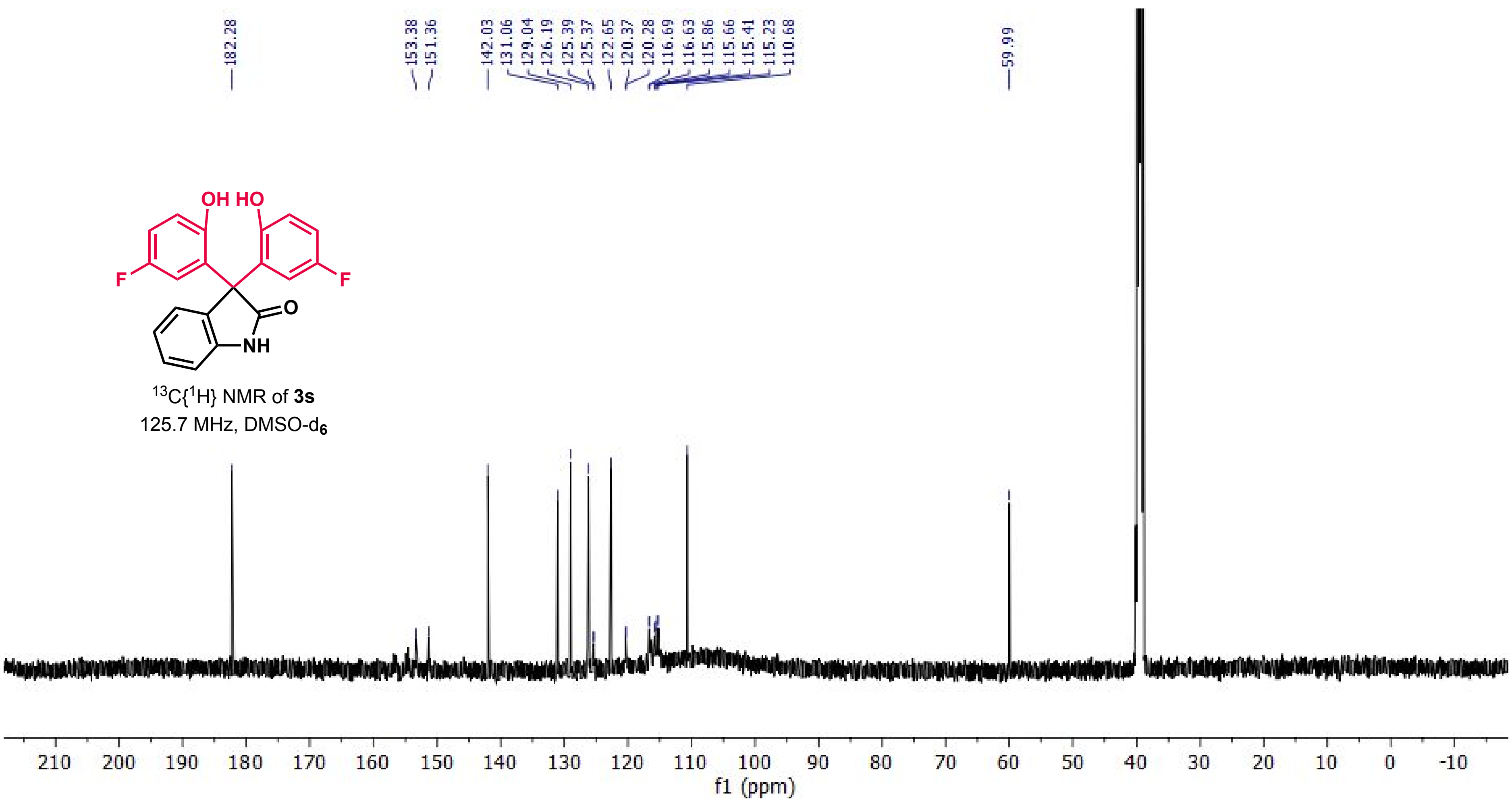

$^{13}C\{^{1}H\}$ NMR of **3s**
125.7 MHz, DMSO-d_6

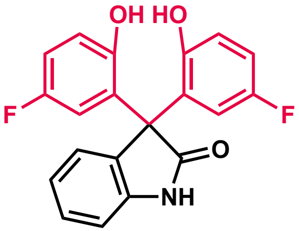

$^{19}F\{^{1}H\}$ NMR of **3s**
282 MHz, DMSO-d_6

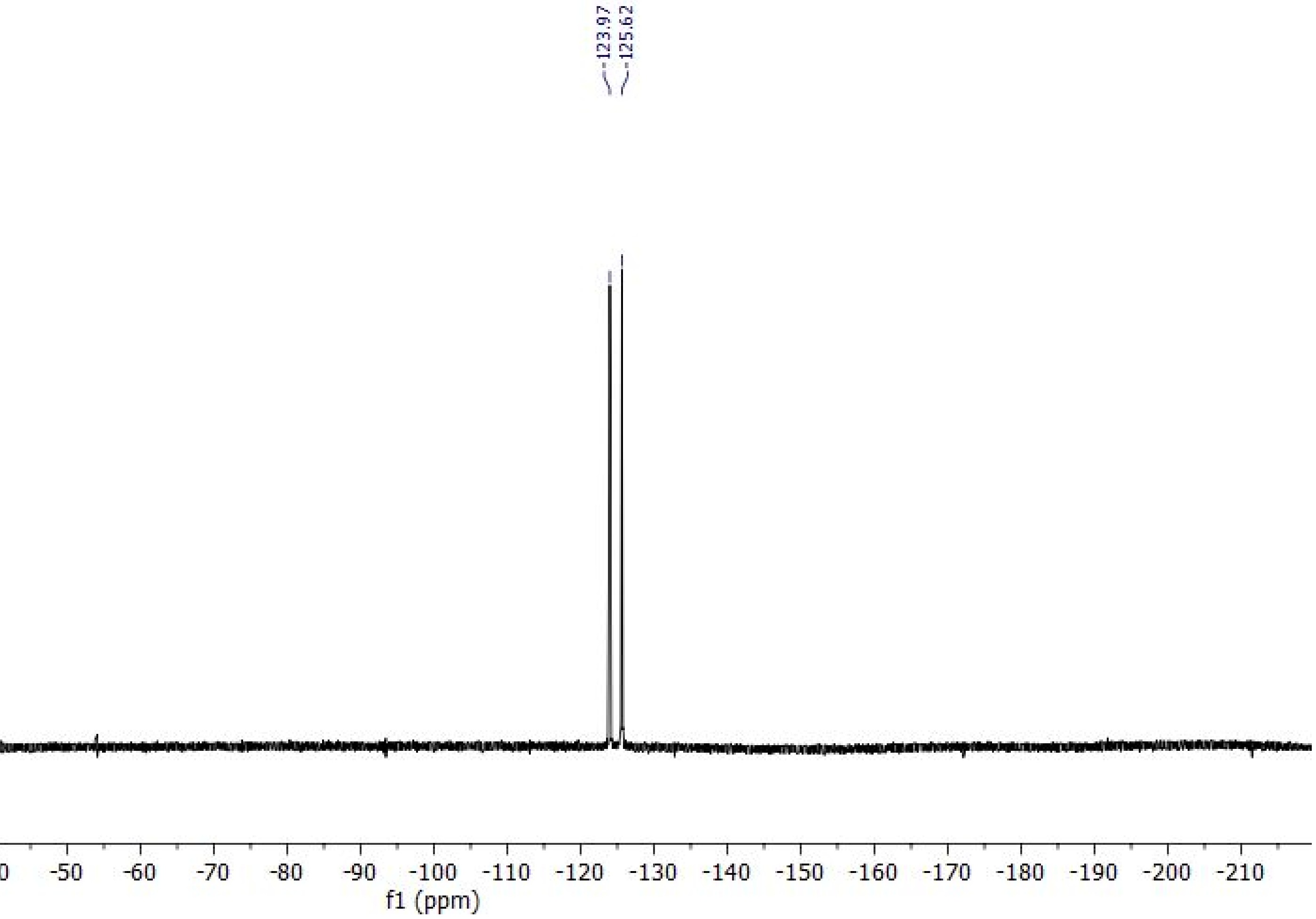

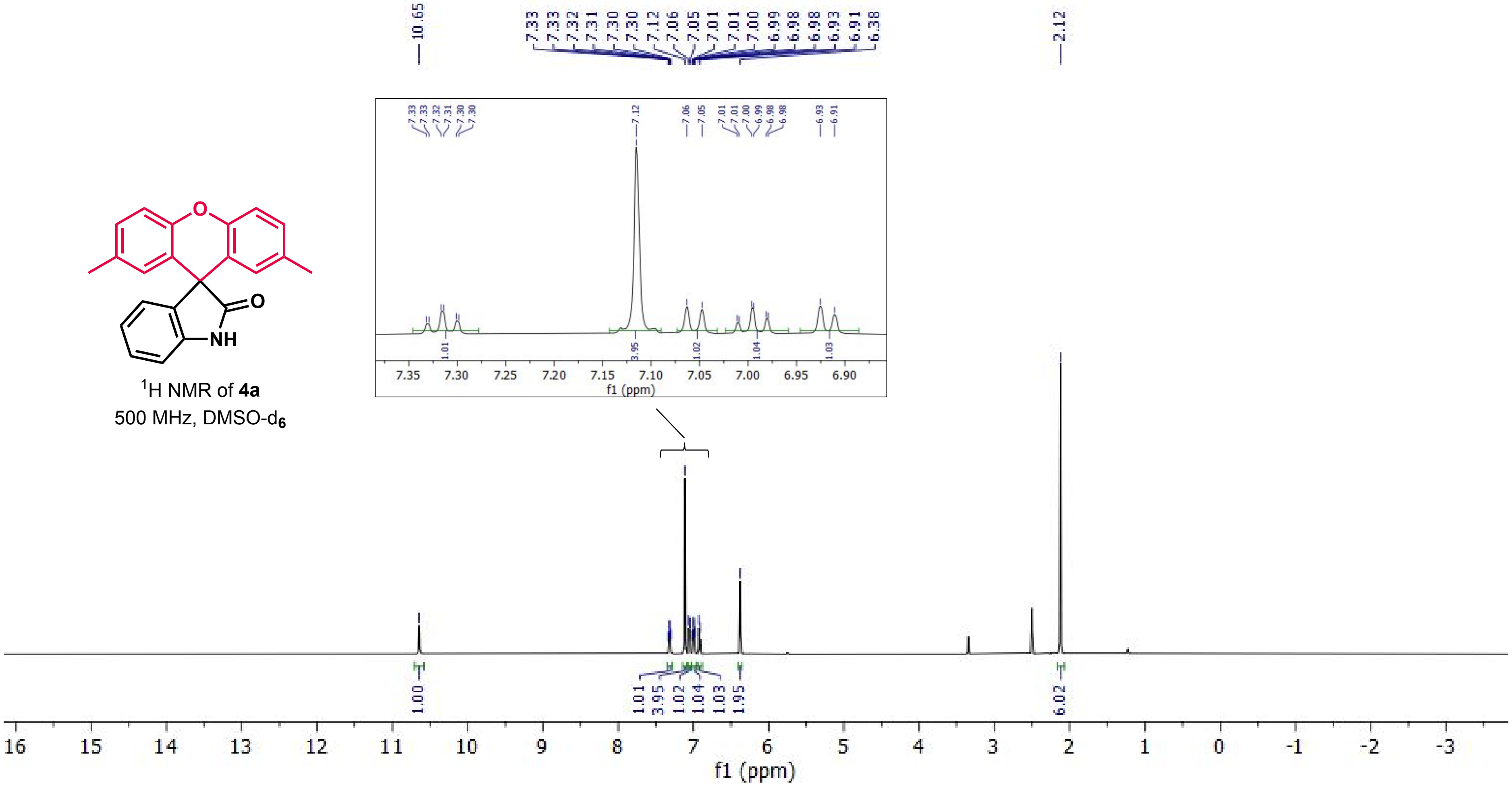

^{1}H NMR of **4a**

500 MHz, DMSO-d_6

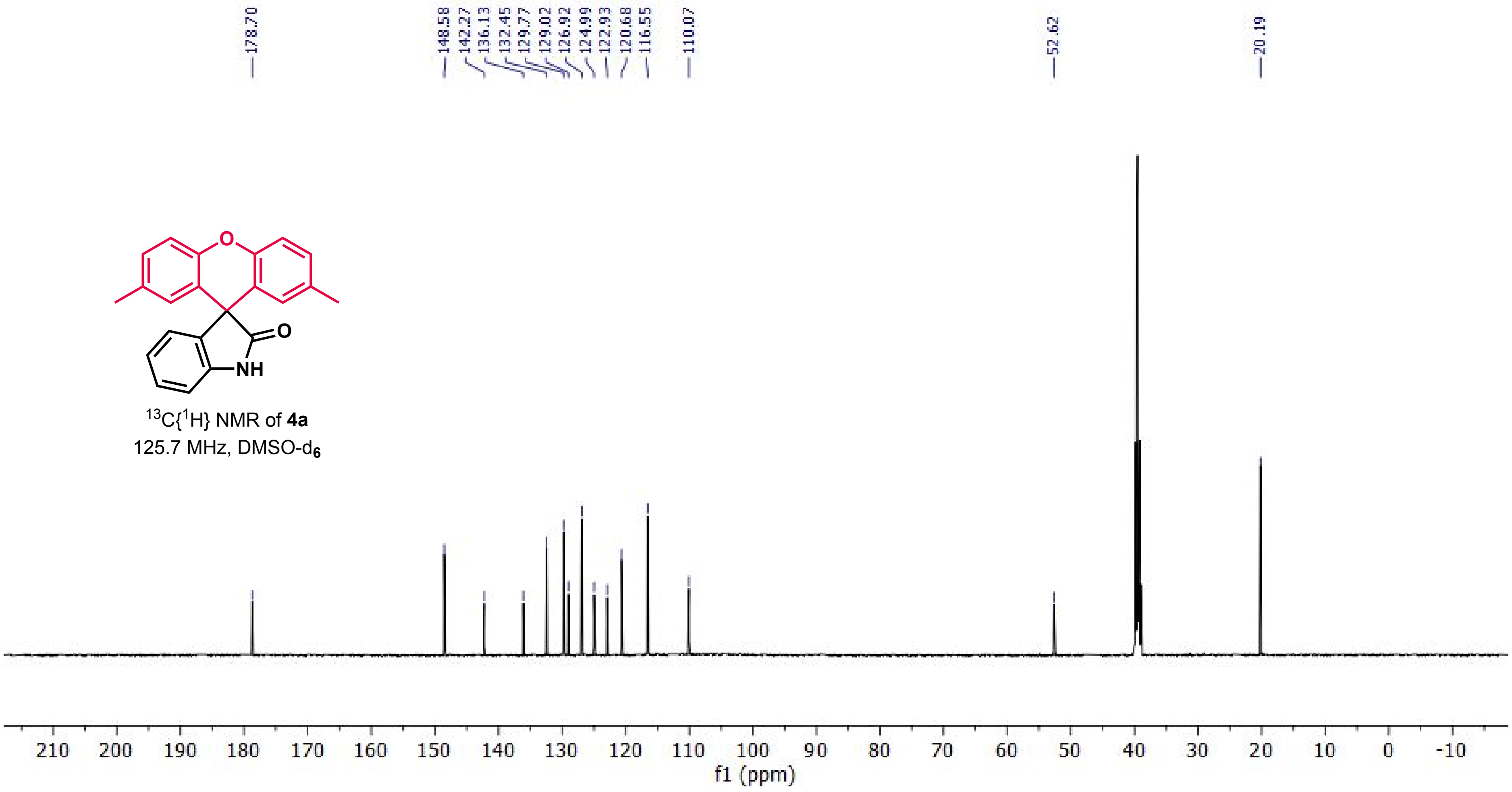
178.70
148.58
142.27
136.13
132.45
129.77
129.02
126.92
124.99
122.93
120.68
116.55
110.07
52.62
20.19
O
NH
$^{13}C\{^1H\}$ NMR of **4a**
125.7 MHz, DMSO-d_6
210 200 190 180 170 160 150 140 130 120 110 100 90 80 70 60 50 40 30 20 10 0 -10
f1 (ppm)

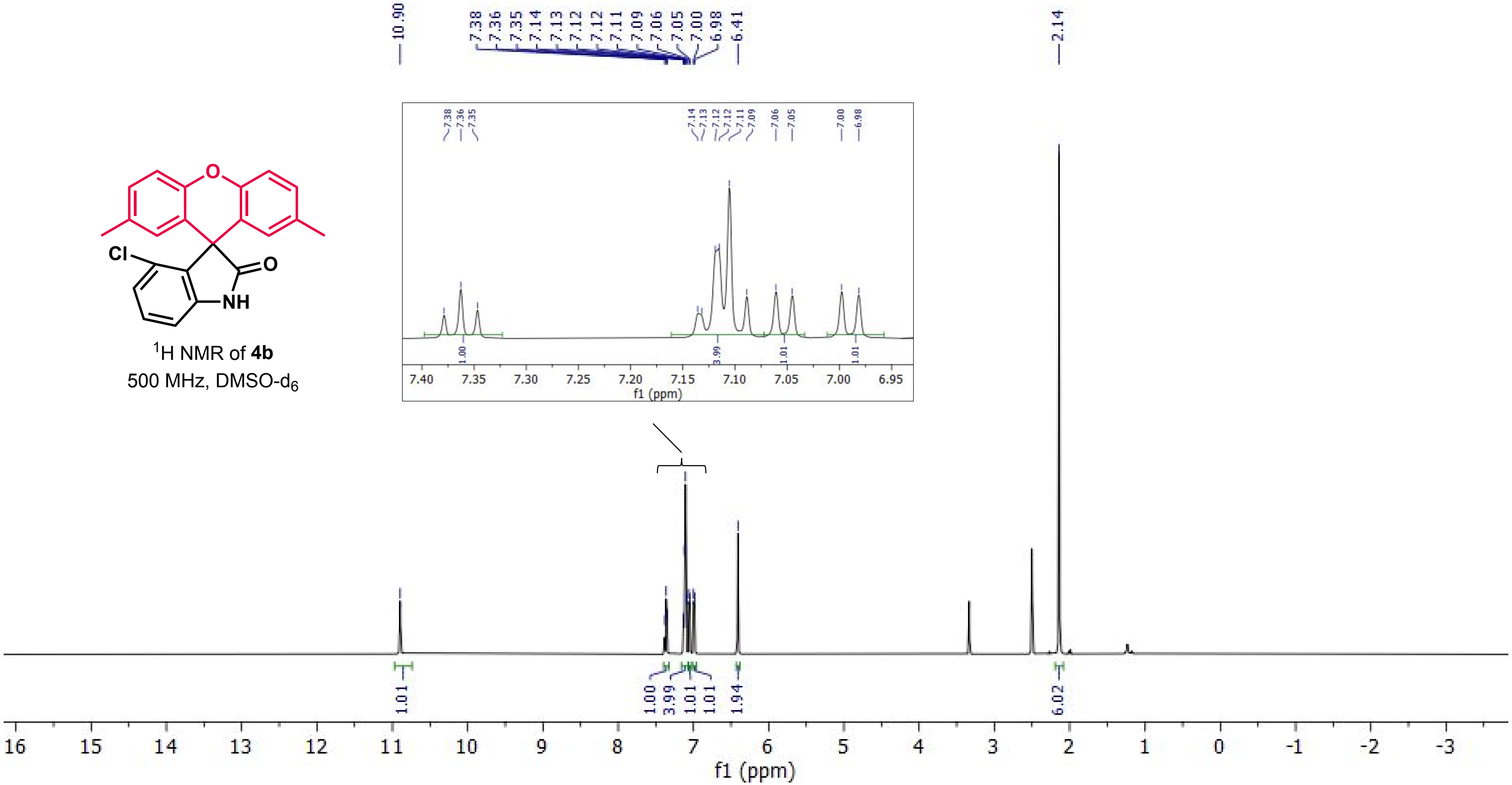

Cl
O
NH
^{1}H NMR of **4b**
500 MHz, DMSO-d_6
10.90
7.38
7.36
7.35
7.14
7.13
7.12
7.12
7.11
7.09
7.06
7.05
7.00
6.98
6.41
2.14
1.01
1.00
3.99
1.01
1.01
1.94
6.02
f1 (ppm)

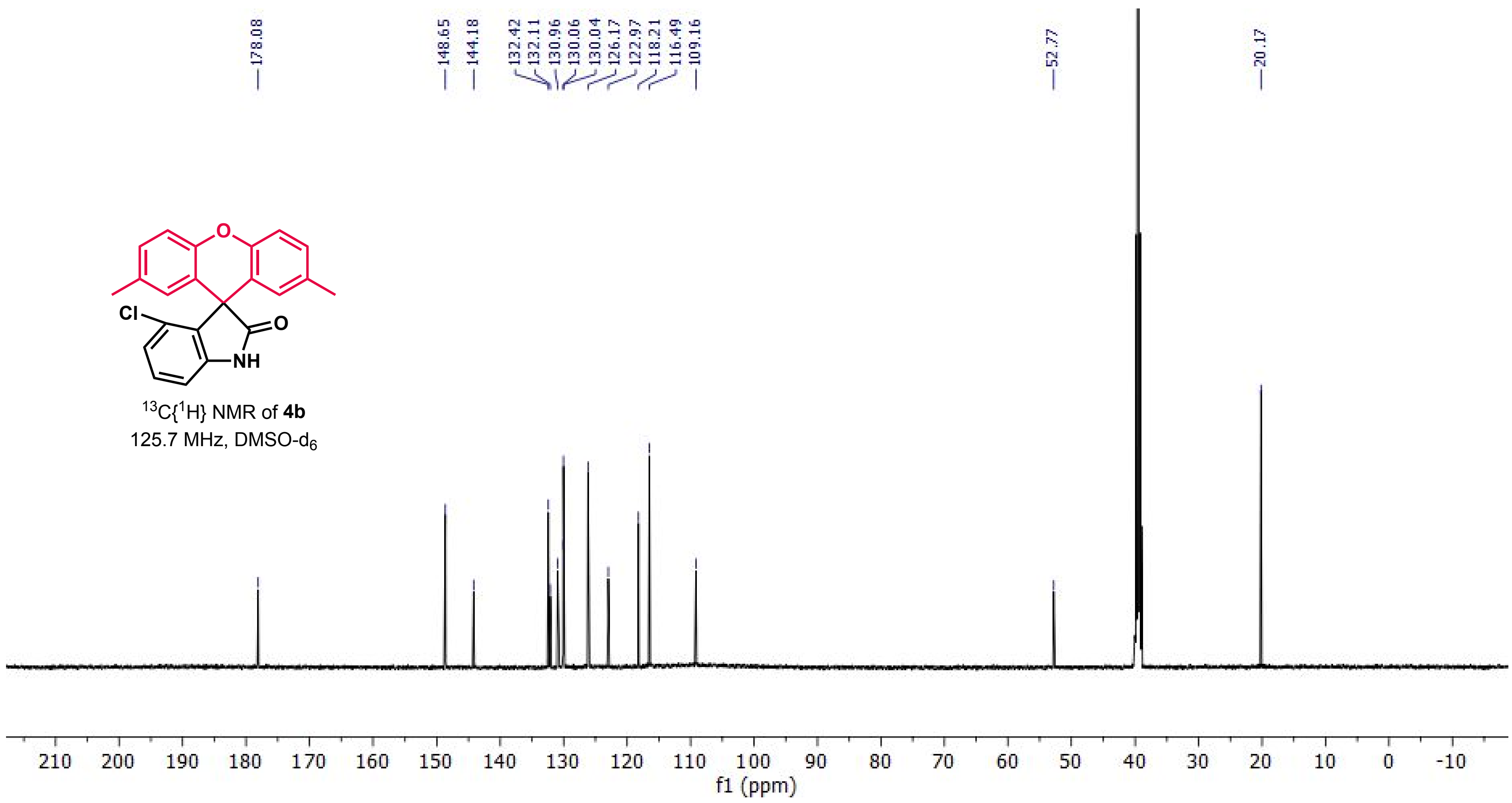
178.08
148.65
144.18
132.42
132.11
130.96
130.06
130.04
126.17
122.97
118.21
116.49
109.16
52.77
20.17
Cl
O
NH
13C{1H} NMR of 4b
125.7 MHz, DMSO-d6
210 200 190 180 170 160 150 140 130 120 110 100 90 80 70 60 50 40 30 20 10 0 -10
f1 (ppm)

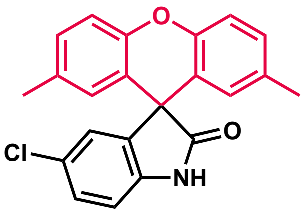

^{1}H NMR of **4c**
500 MHz, DMSO-d$_6$

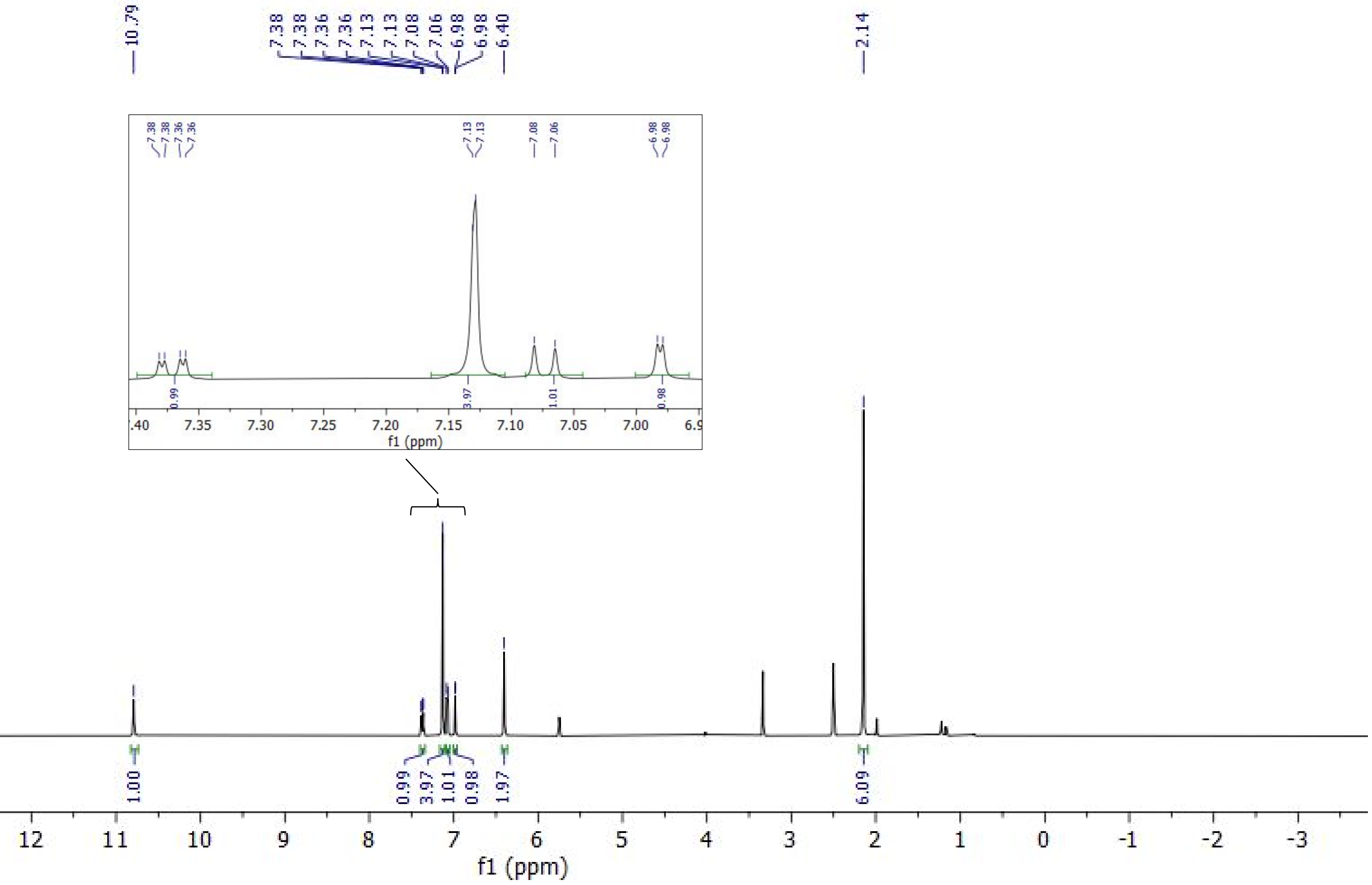

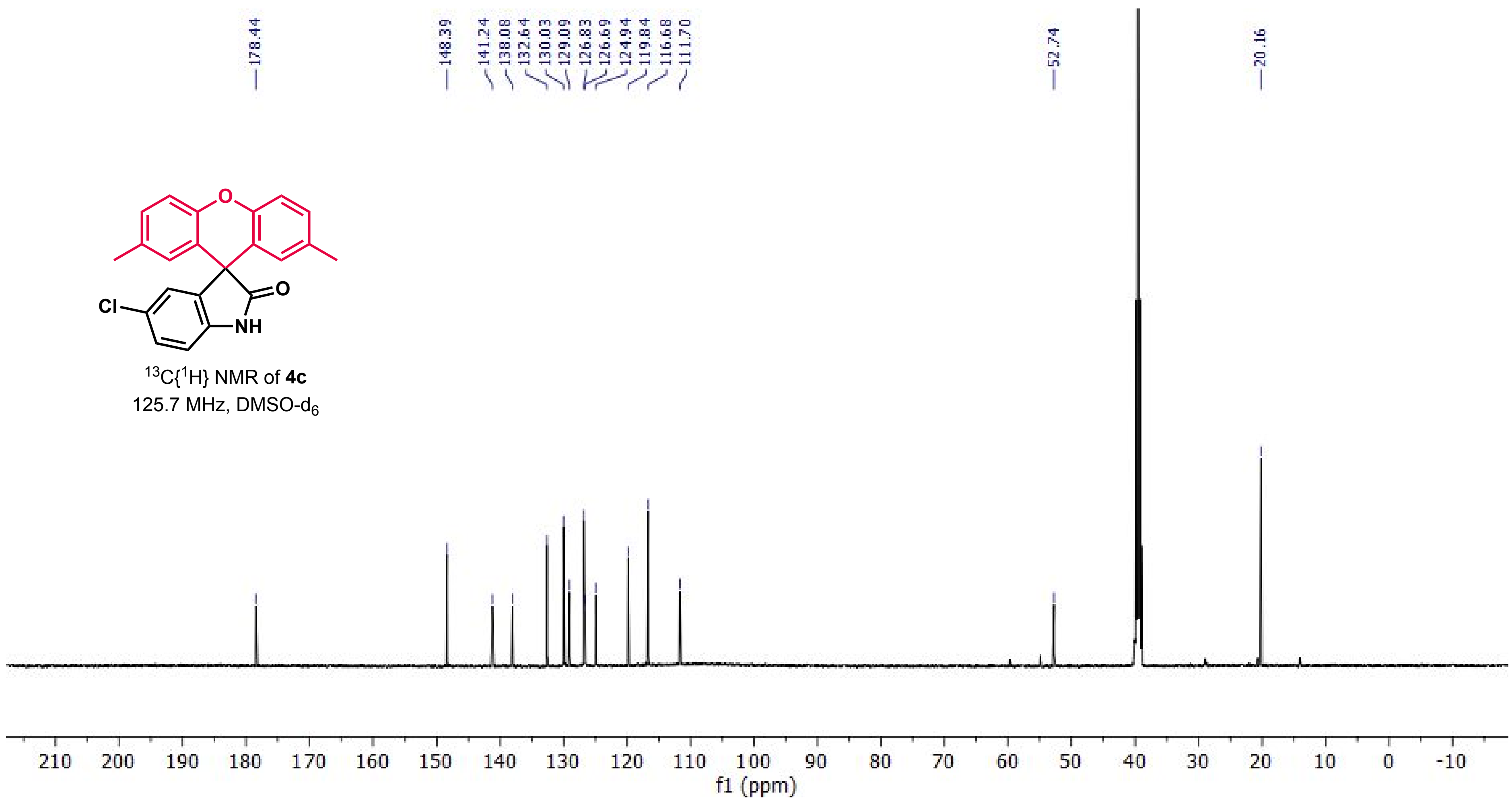
178.44
148.39
141.24
138.08
132.64
130.03
129.09
126.83
126.69
124.94
119.84
116.68
111.70
52.74
20.16
O
Cl
NH
O
^{13}C{^{1}H} NMR of **4c**
125.7 MHz, DMSO-d_6
210 200 190 180 170 160 150 140 130 120 110 100 90 80 70 60 50 40 30 20 10 0 -10
f1 (ppm)

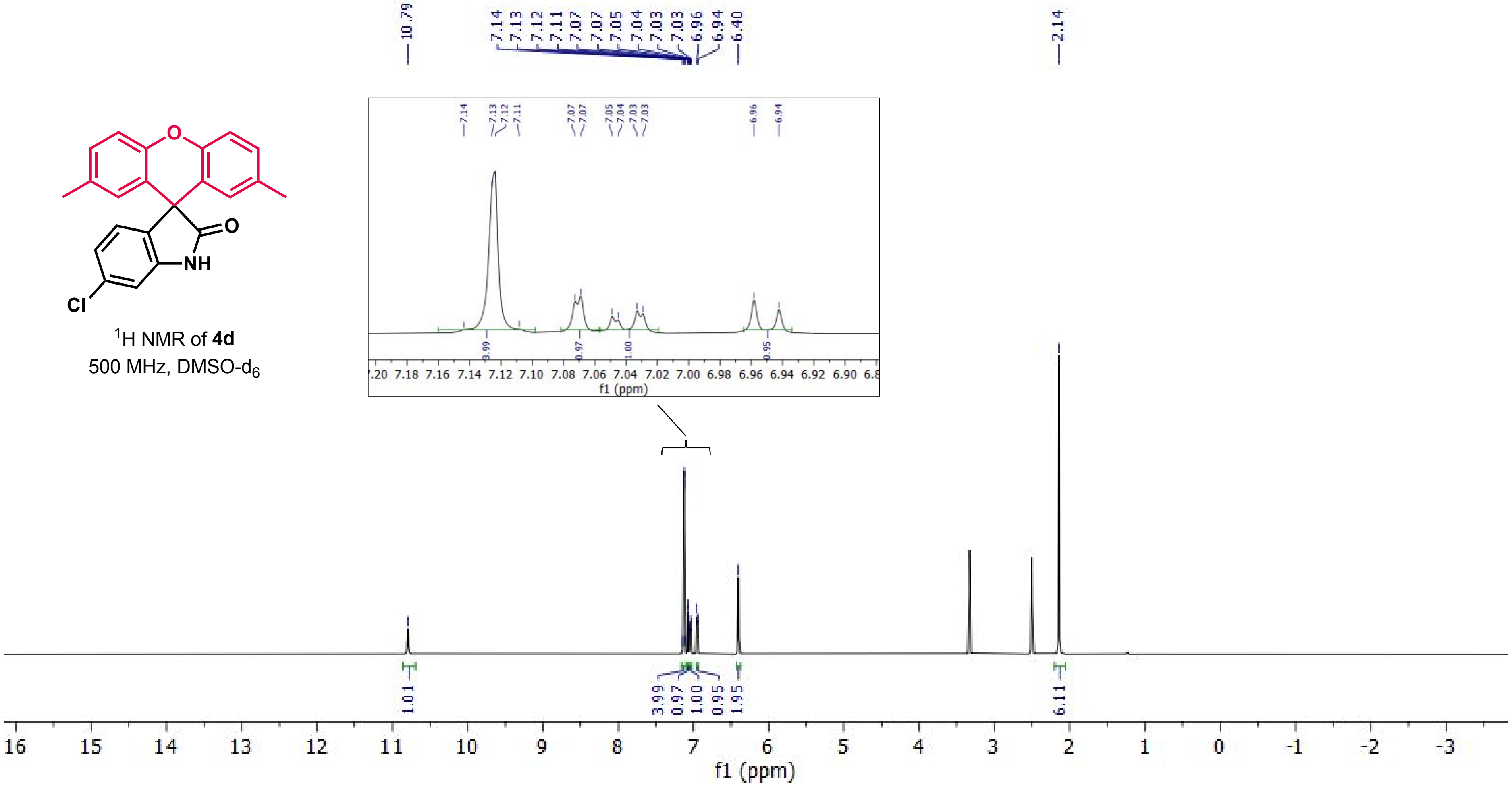
^{1}H NMR of 4d
500 MHz, DMSO-d6
10.79
7.14
7.13
7.12
7.11
7.07
7.07
7.05
7.04
7.03
7.03
6.96
6.94
6.40
2.14
1.01
3.99
0.97
1.00
0.95
1.95
6.11
f1 (ppm)

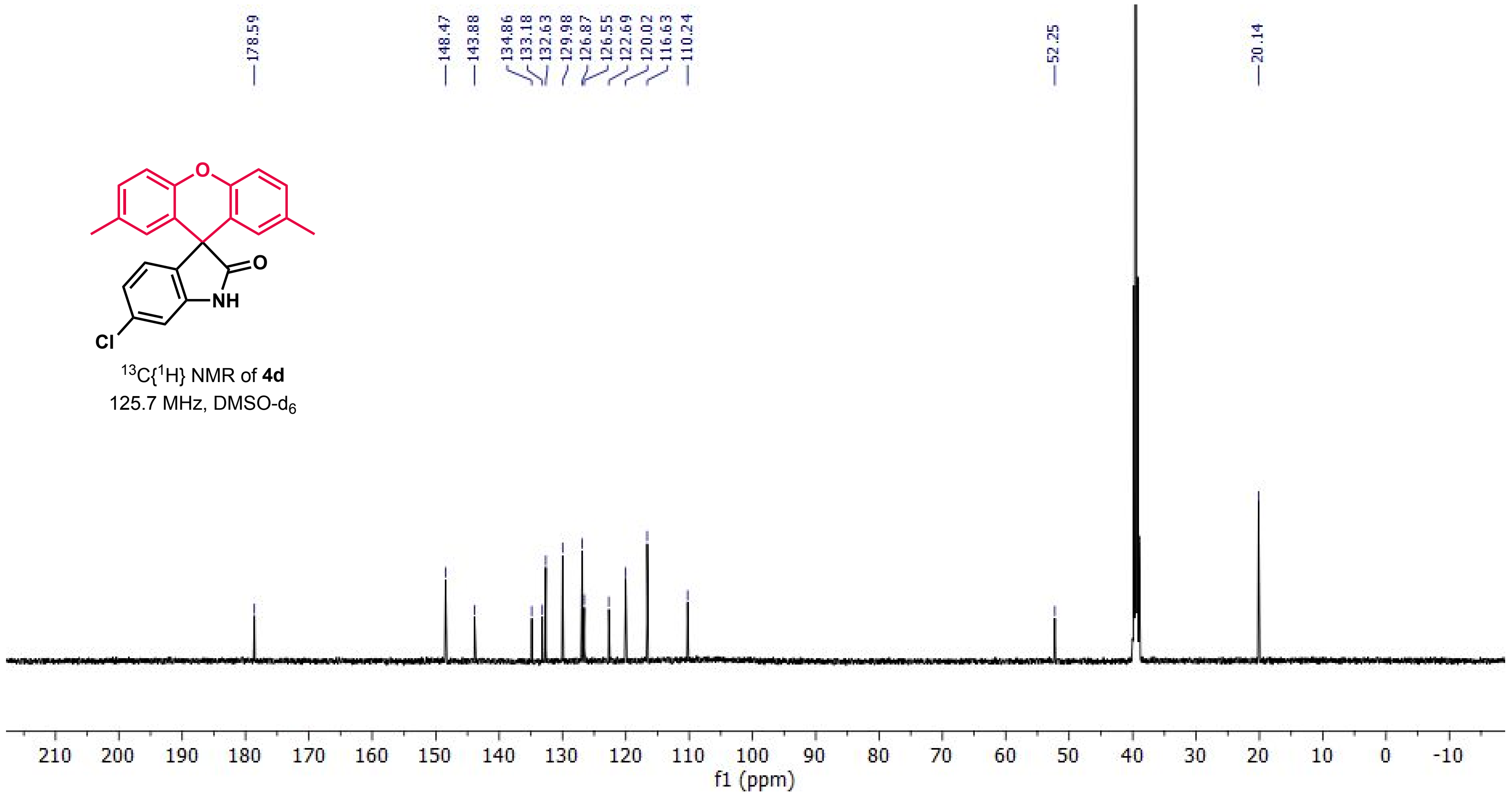
178.59
148.47
143.88
134.86
133.18
132.63
129.98
126.87
126.55
122.69
120.02
116.63
110.24
52.25
20.14
O
NH
Cl
$^{13}C\{^{1}H\}$ NMR of **4d**
125.7 MHz, DMSO-d_6
210 200 190 180 170 160 150 140 130 120 110 100 90 80 70 60 50 40 30 20 10 0 -10
f1 (ppm)

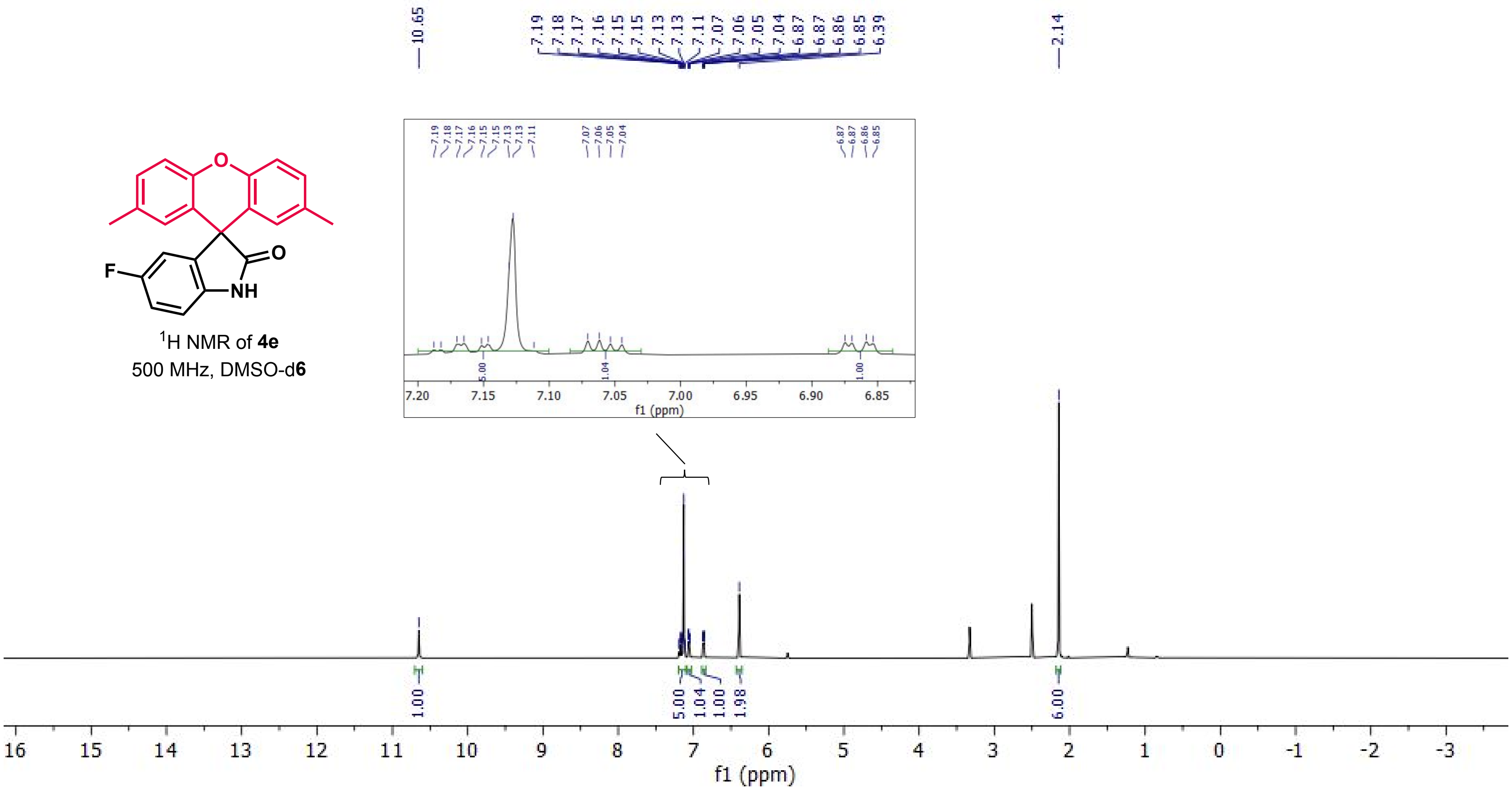

^{1}H NMR of **4e**
500 MHz, DMSO-d**6**

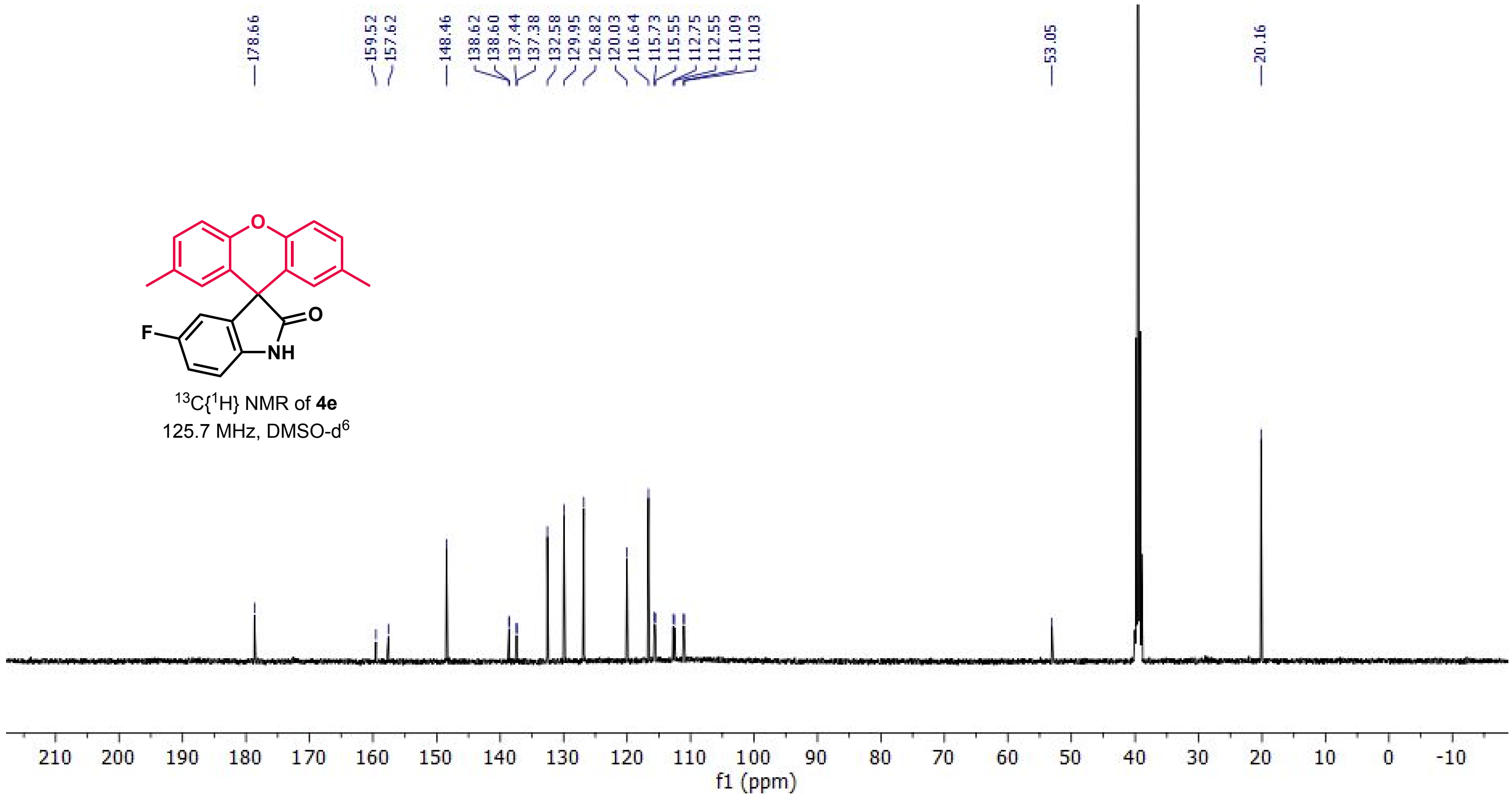
178.66
159.52
157.62
148.46
138.62
138.60
137.44
137.38
132.58
129.95
126.82
120.03
116.64
115.73
115.55
112.75
112.55
111.09
111.03
53.05
20.16
O
F
NH
13C{1H} NMR of 4e
125.7 MHz, DMSO-d6
210
200
190
180
170
160
150
140
130
120
110
100
90
80
70
60
50
40
30
20
10
0
-10
f1 (ppm)

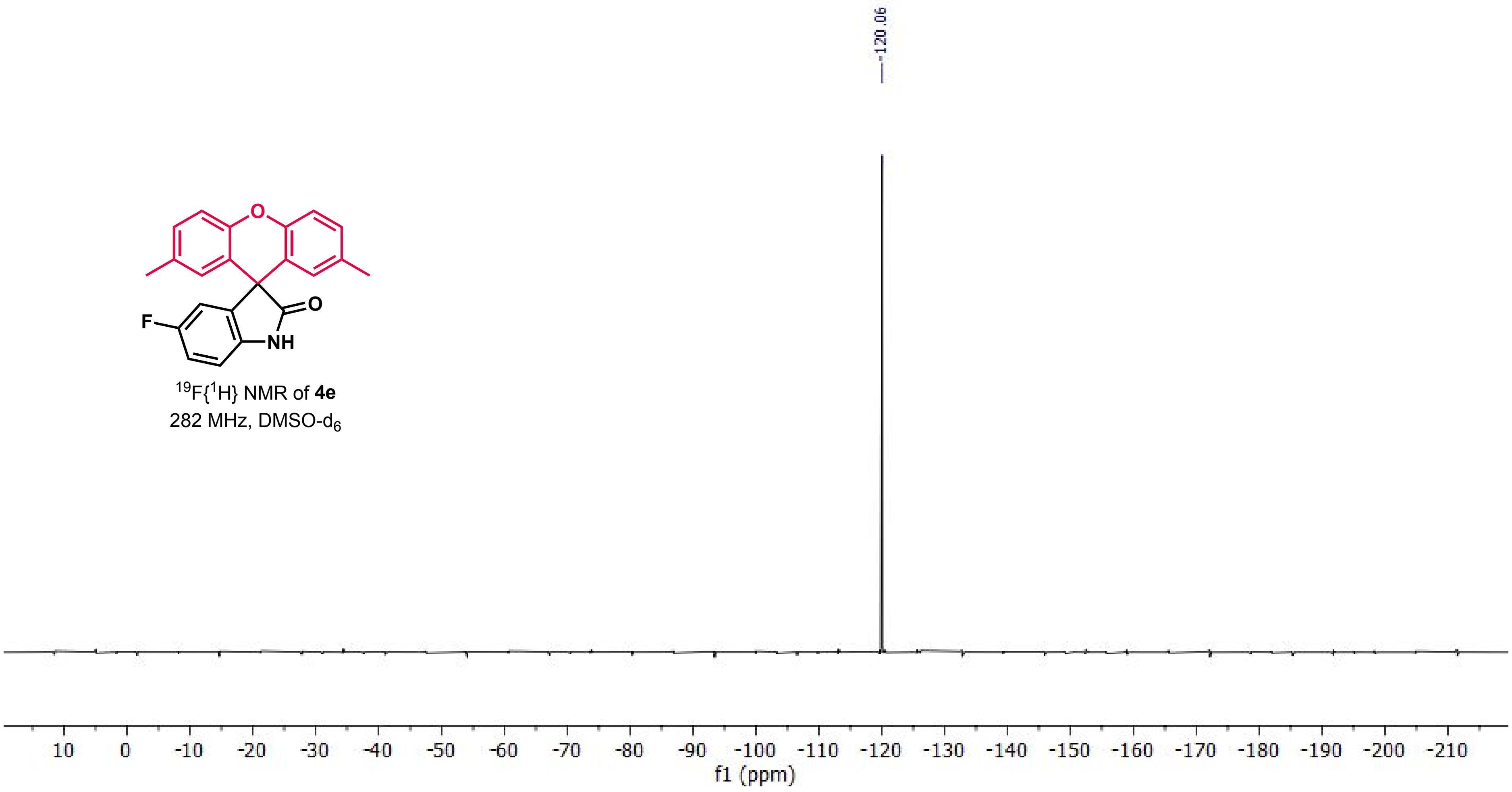
-120.06
F
O
NH
$^{19}F\{^{1}H\}$ NMR of **4e**
282 MHz, DMSO-d_6
10 0 -10 -20 -30 -40 -50 -60 -70 -80 -90 -100 -110 -120 -130 -140 -150 -160 -170 -180 -190 -200 -210
f1 (ppm)

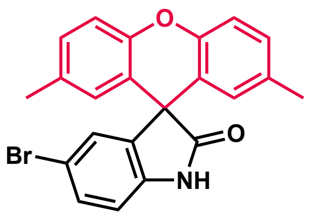

^{1}H NMR of **4f**
500 MHz, DMSO-d**6**

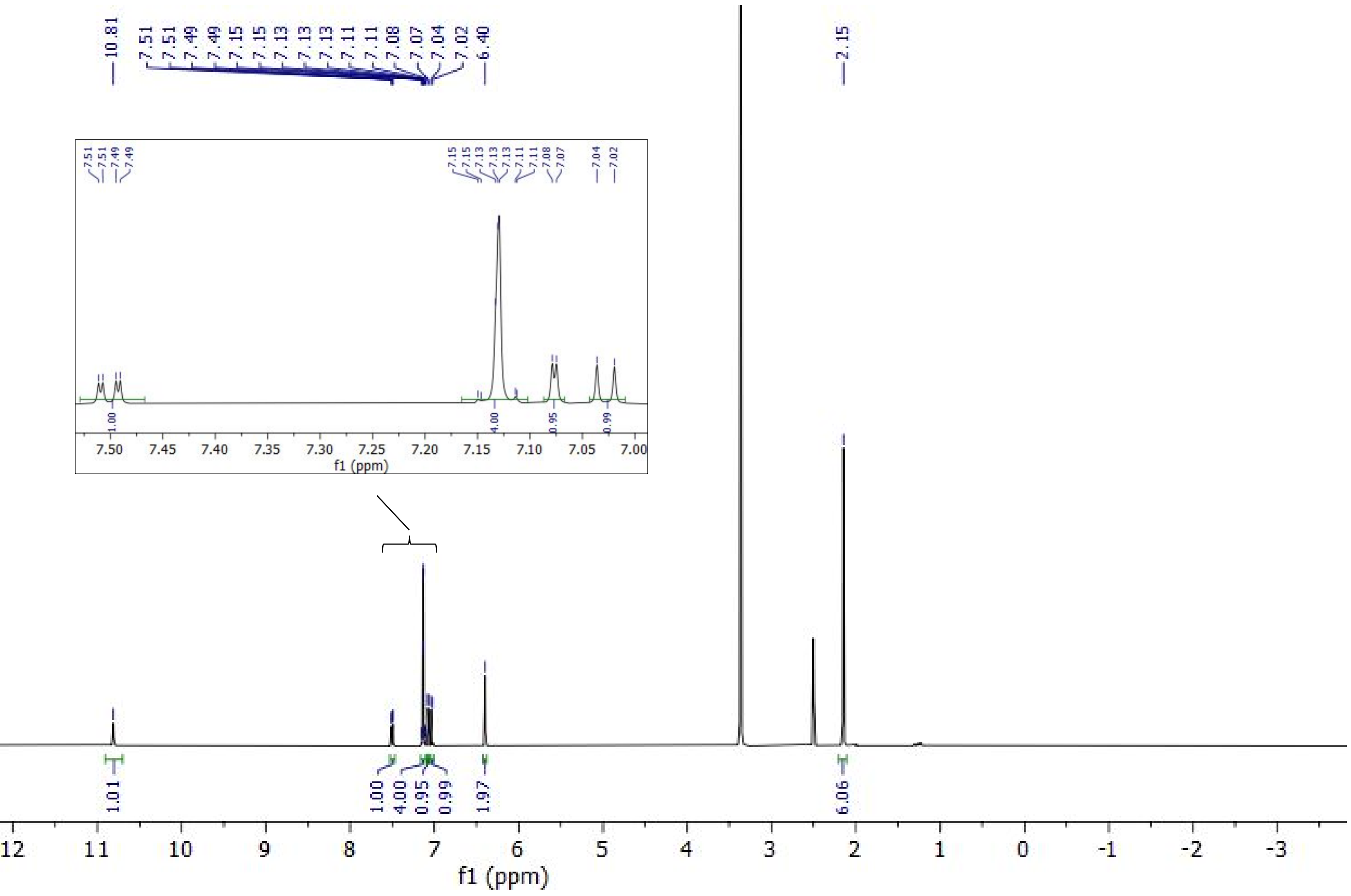

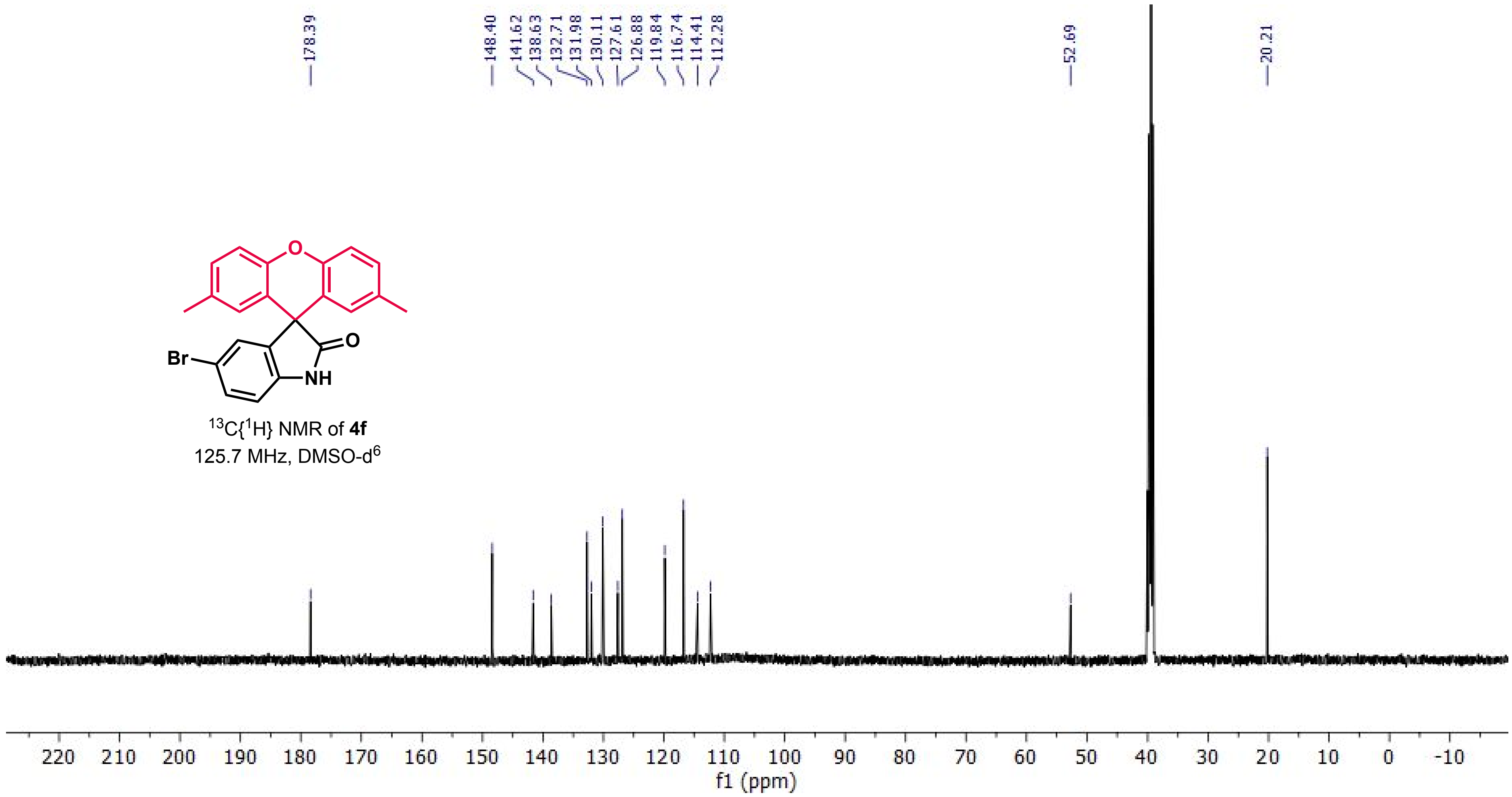

$^{13}C\{^{1}H\}$ NMR of **4f**
125.7 MHz, DMSO-d^{6}

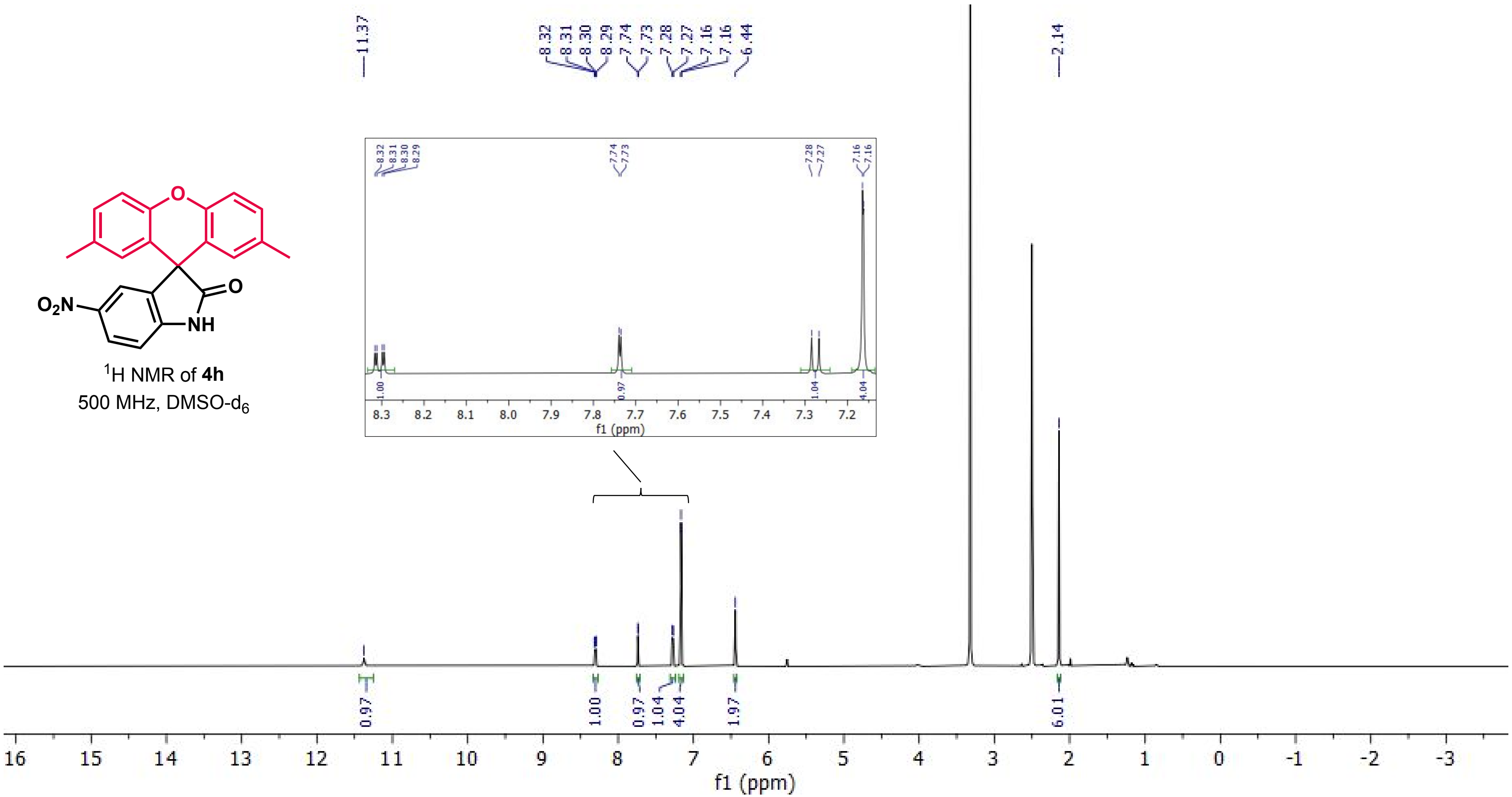

^{1}H NMR of **4h**
500 MHz, DMSO-d$_6$

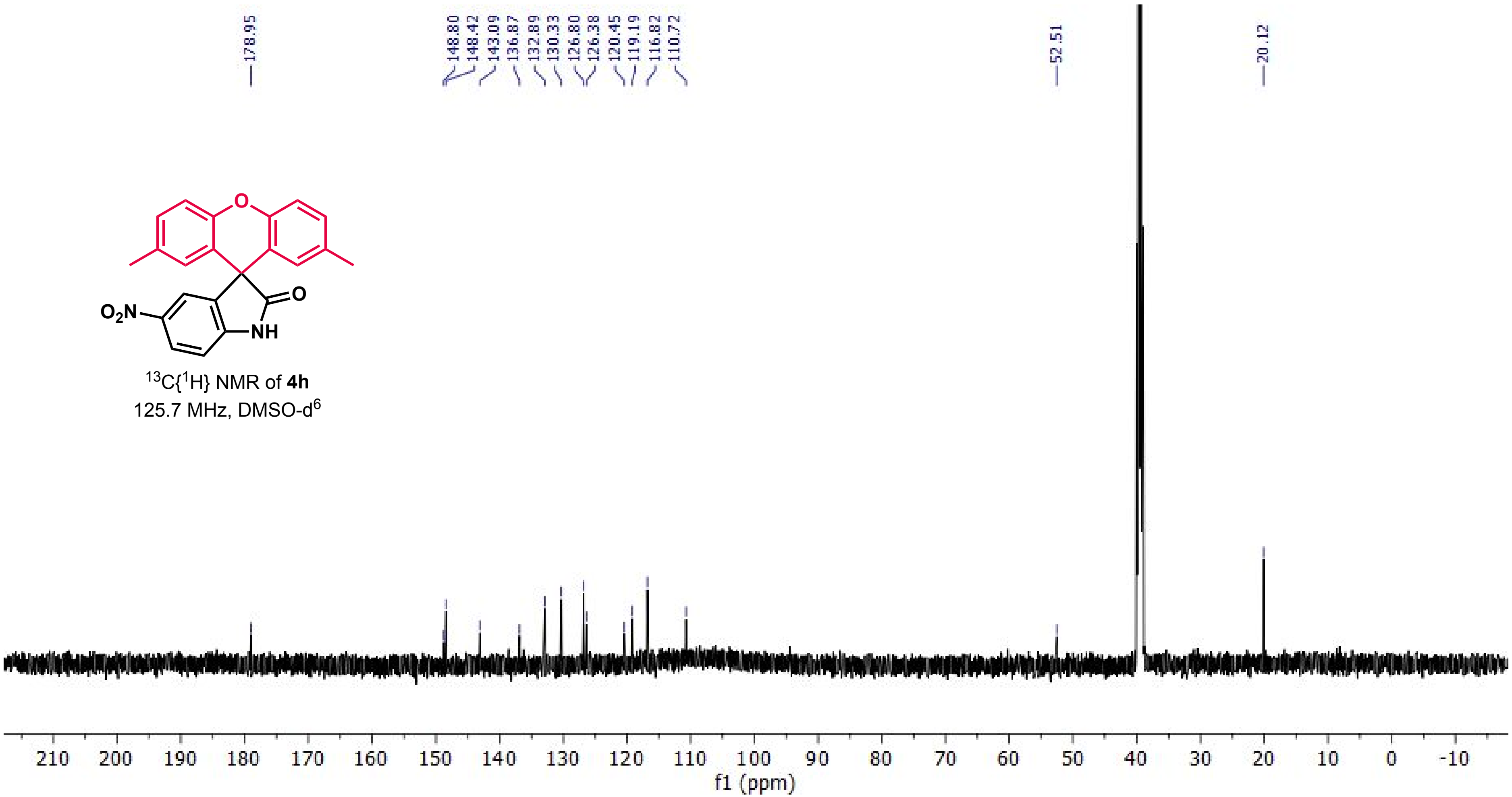
178.95
148.80
148.42
143.09
136.87
132.89
130.33
126.80
126.38
120.45
119.19
116.82
110.72
52.51
20.12
O
O₂N
O
NH
13C{1H} NMR of 4h
125.7 MHz, DMSO-d6
210 200 190 180 170 160 150 140 130 120 110 100 90 80 70 60 50 40 30 20 10 0 -10
f1 (ppm)

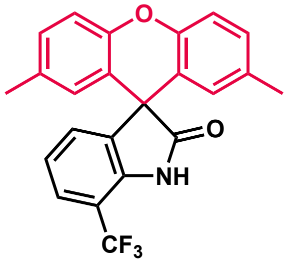

^{1}H NMR of **4i**
500 MHz, DMSO-d$_6$

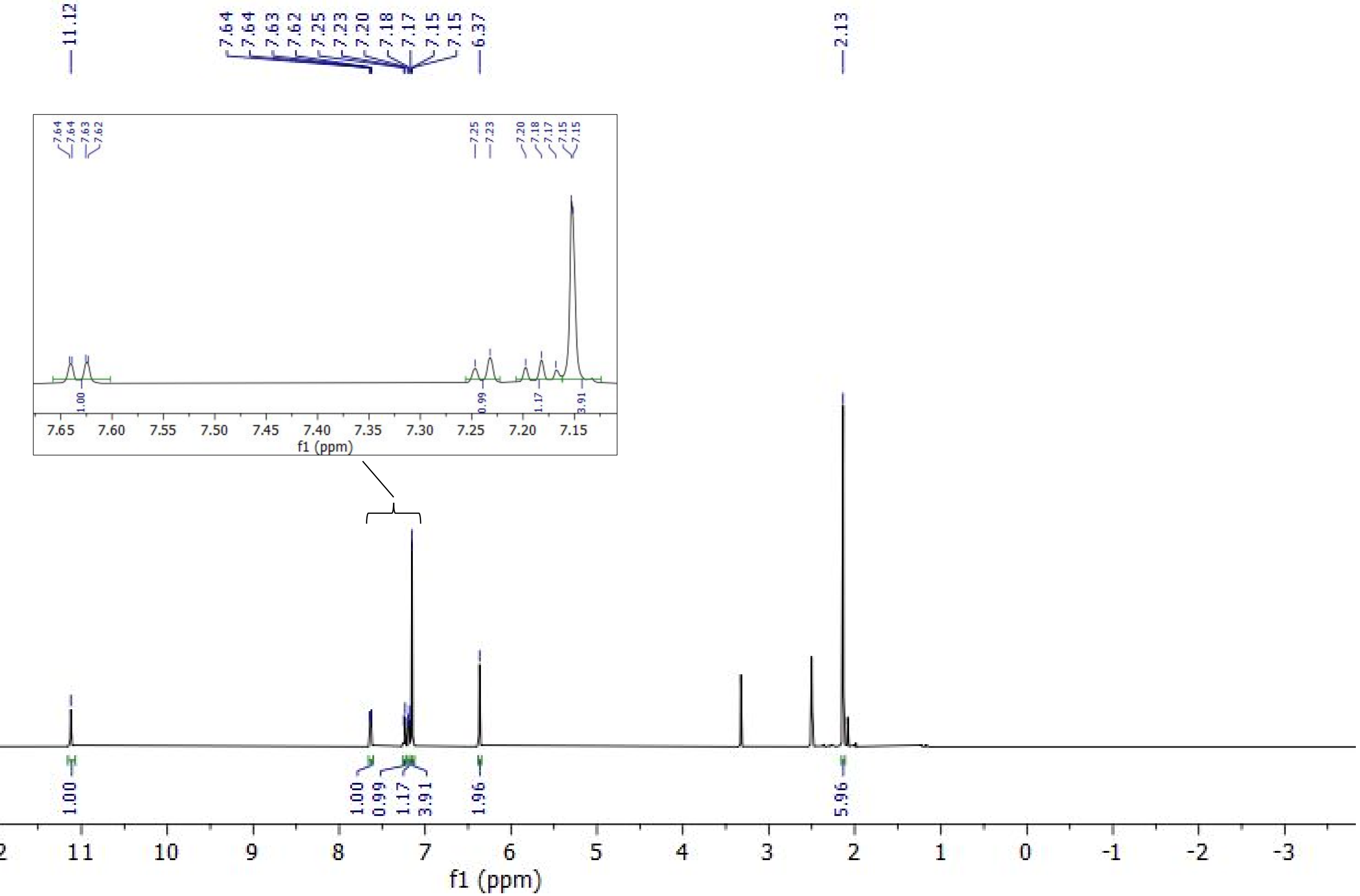

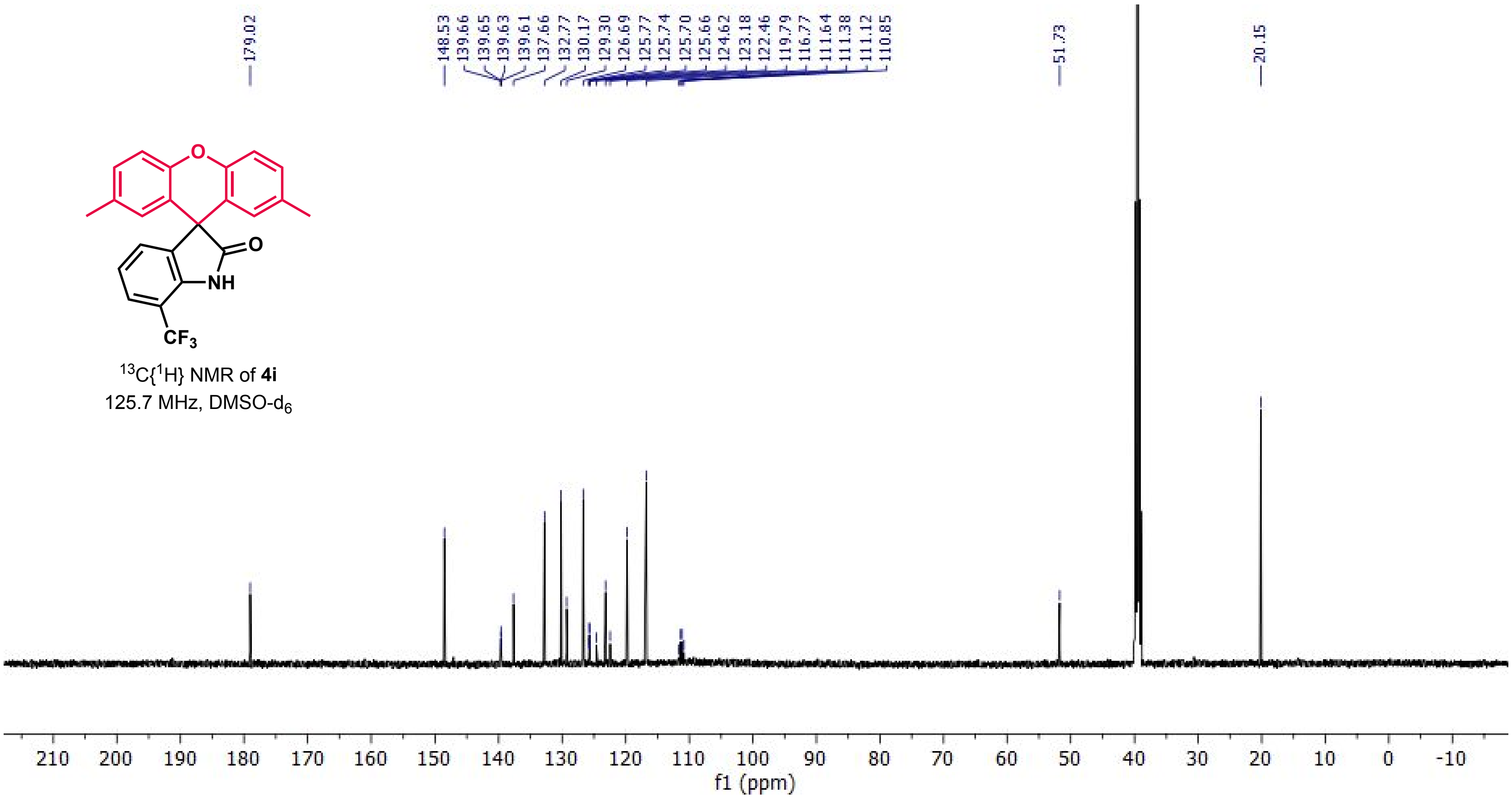

$^{13}C\{^1H\}$ NMR of **4i**
125.7 MHz, DMSO-d_6

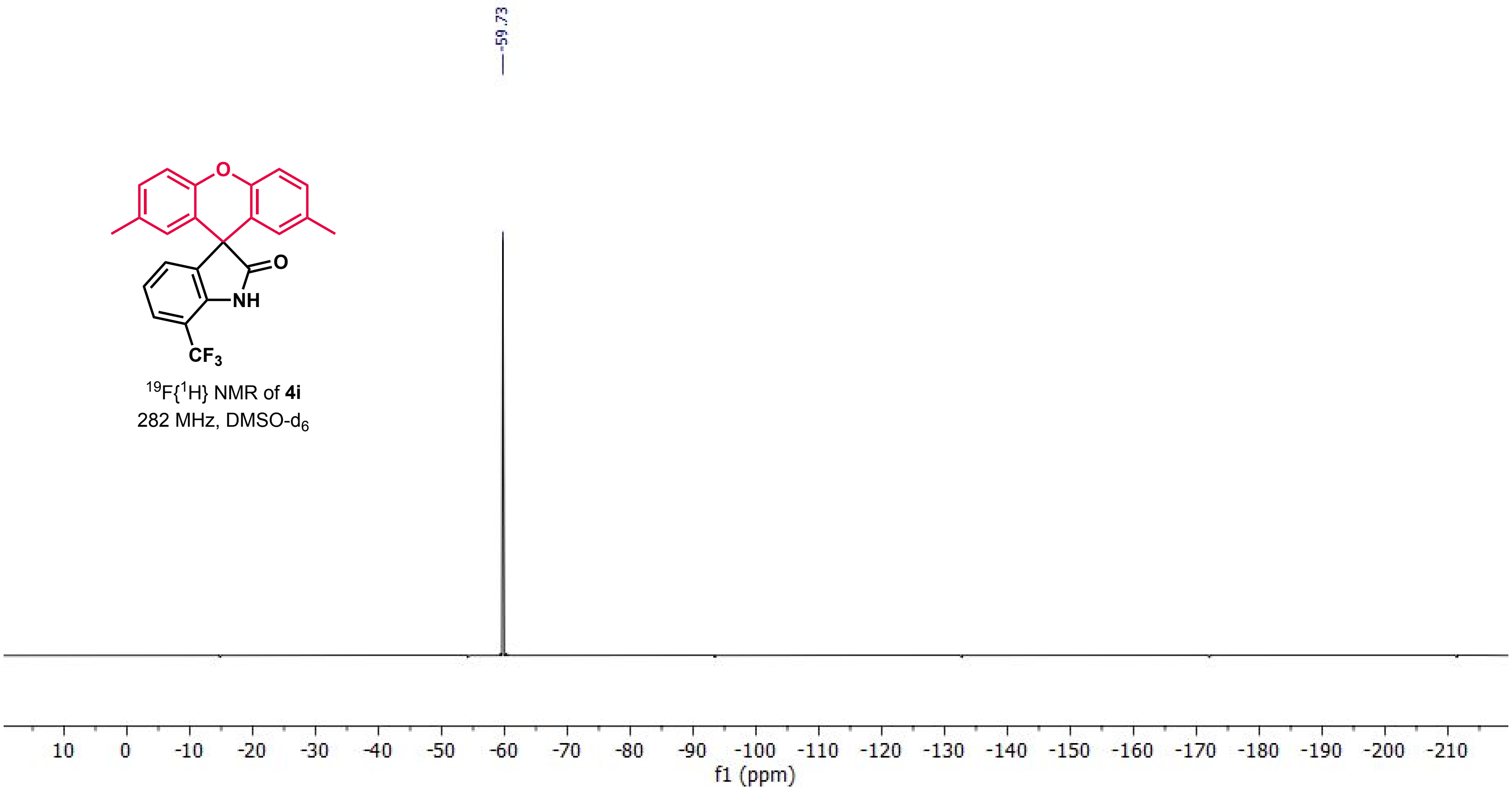

$^{19}F\{^{1}H\}$ NMR of **4i**
282 MHz, DMSO-d_6

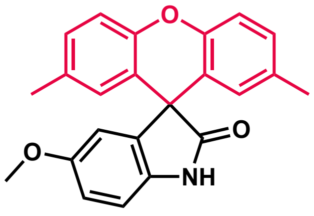

^{1}H NMR of **4j**
500 MHz, DMSO-d$_6$

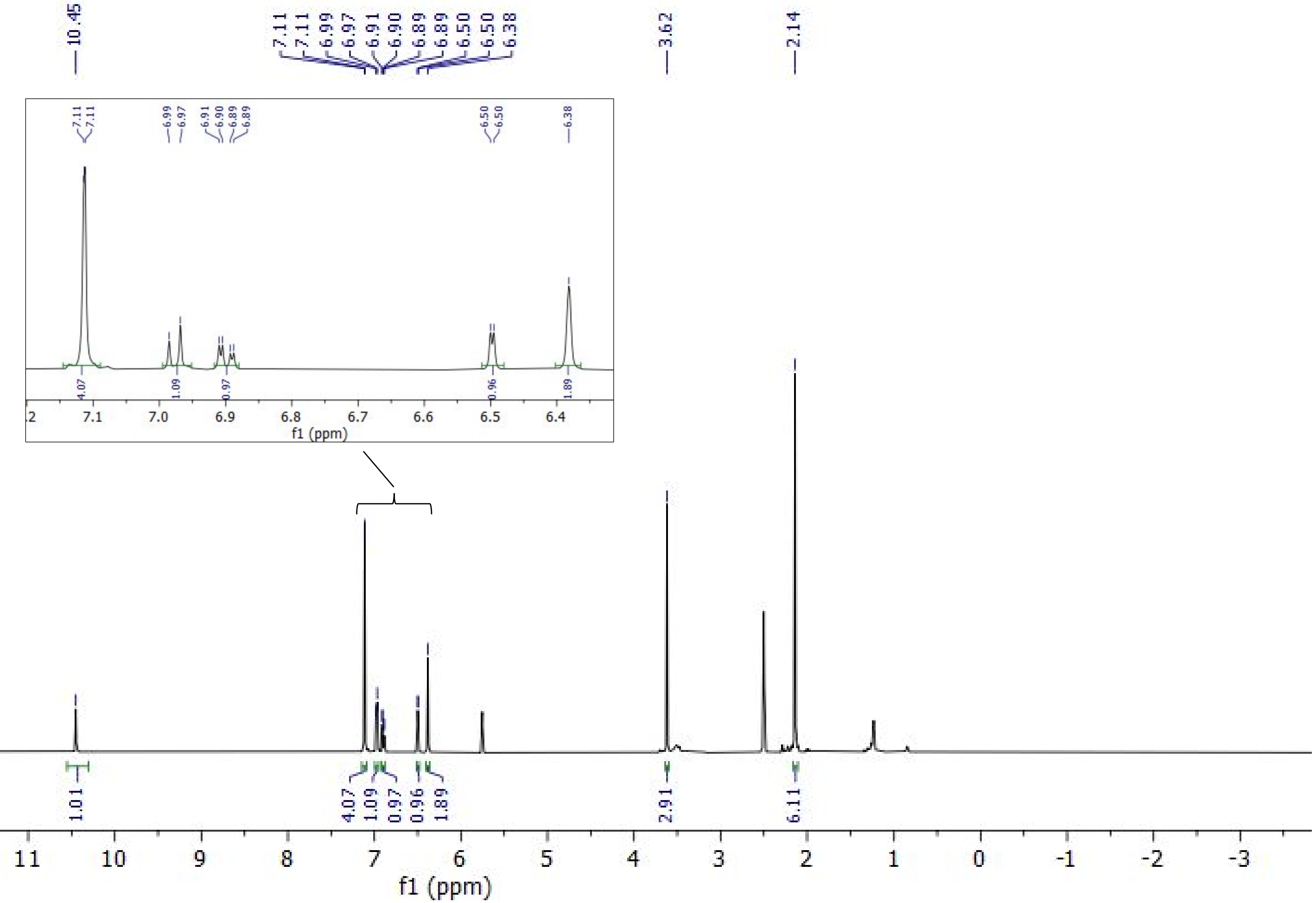

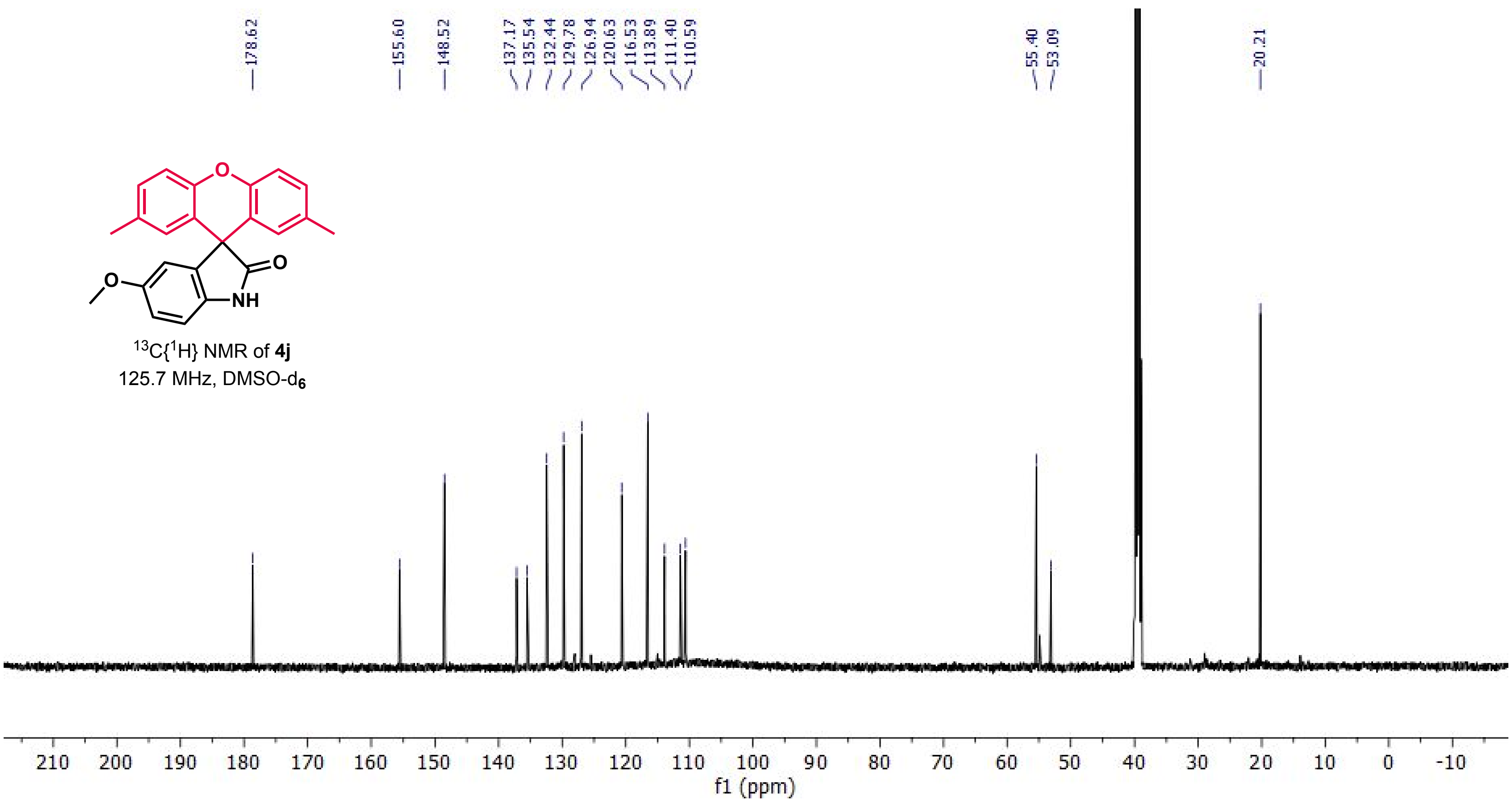
178.62
155.60
148.52
137.17
135.54
132.44
129.78
126.94
120.63
116.53
113.89
111.40
110.59
55.40
53.09
20.21
O
NH
13C{1H} NMR of 4j
125.7 MHz, DMSO-d6
210 200 190 180 170 160 150 140 130 120 110 100 90 80 70 60 50 40 30 20 10 0 -10
f1 (ppm)

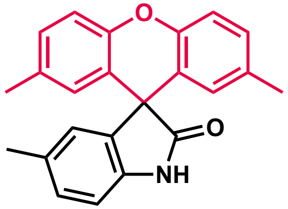

^1H NMR of **4k**
500 MHz, DMSO-d_6

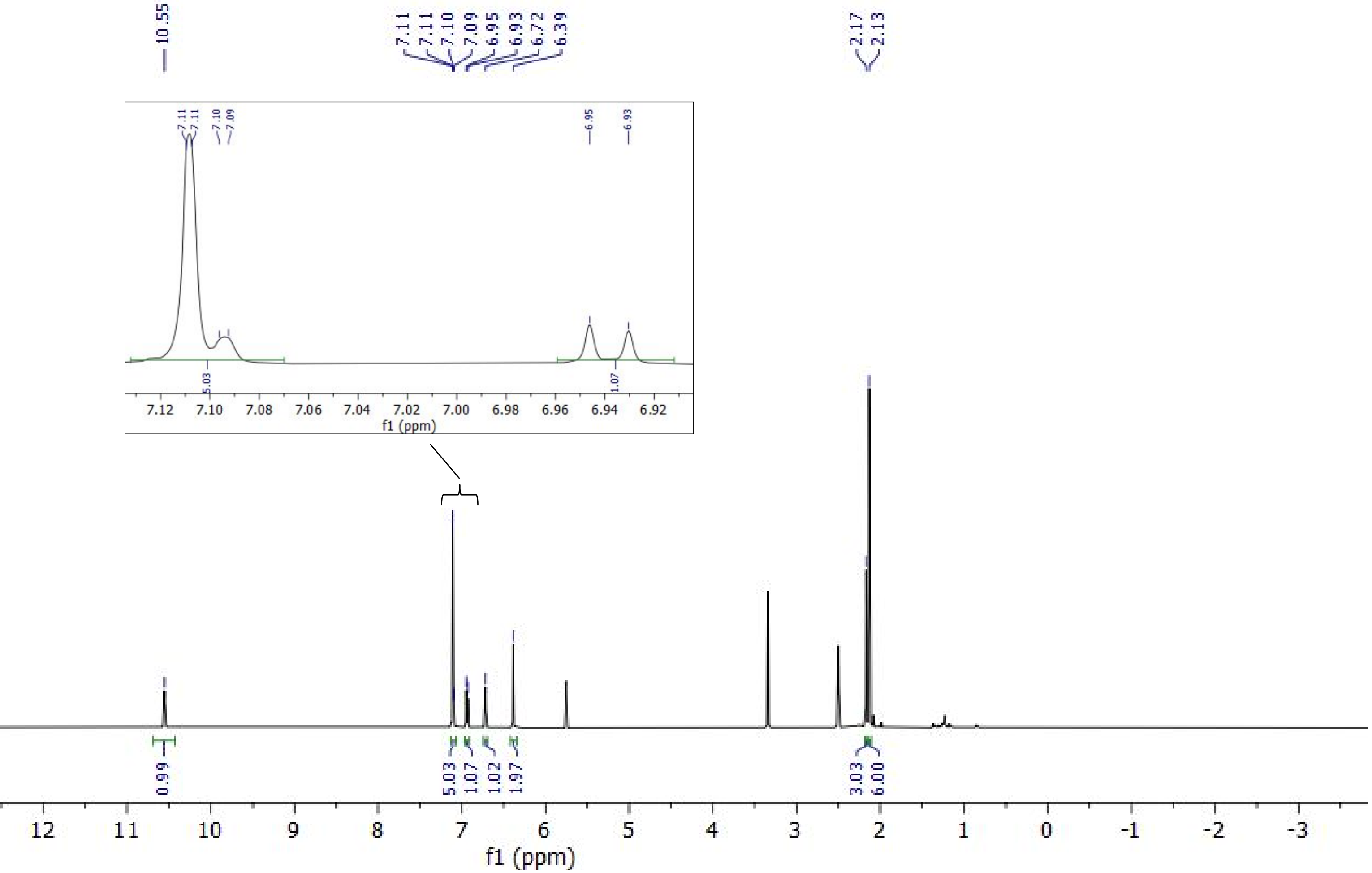

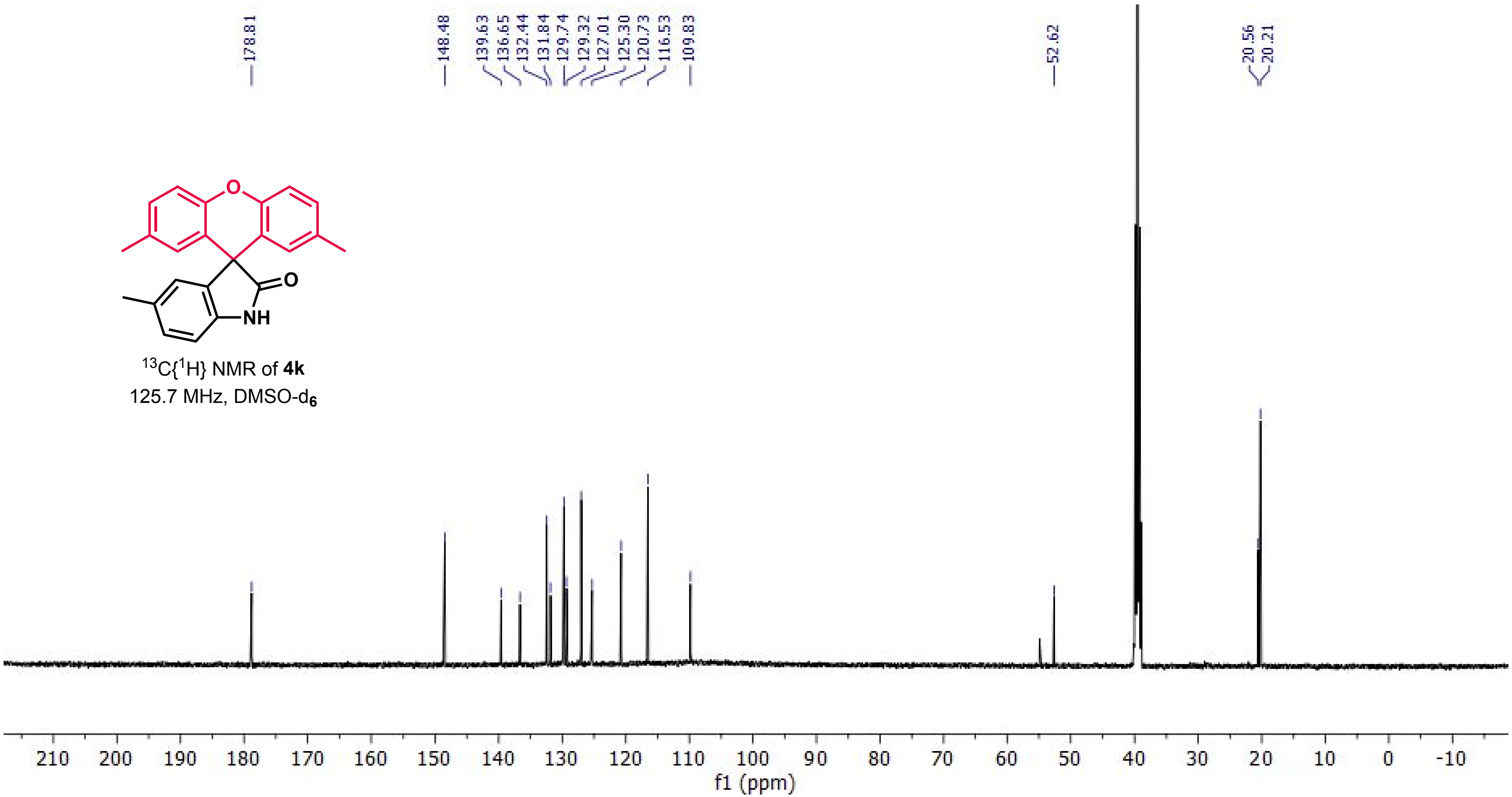

$^{13}C\{^{1}H\}$ NMR of **4k**
125.7 MHz, DMSO-d_6

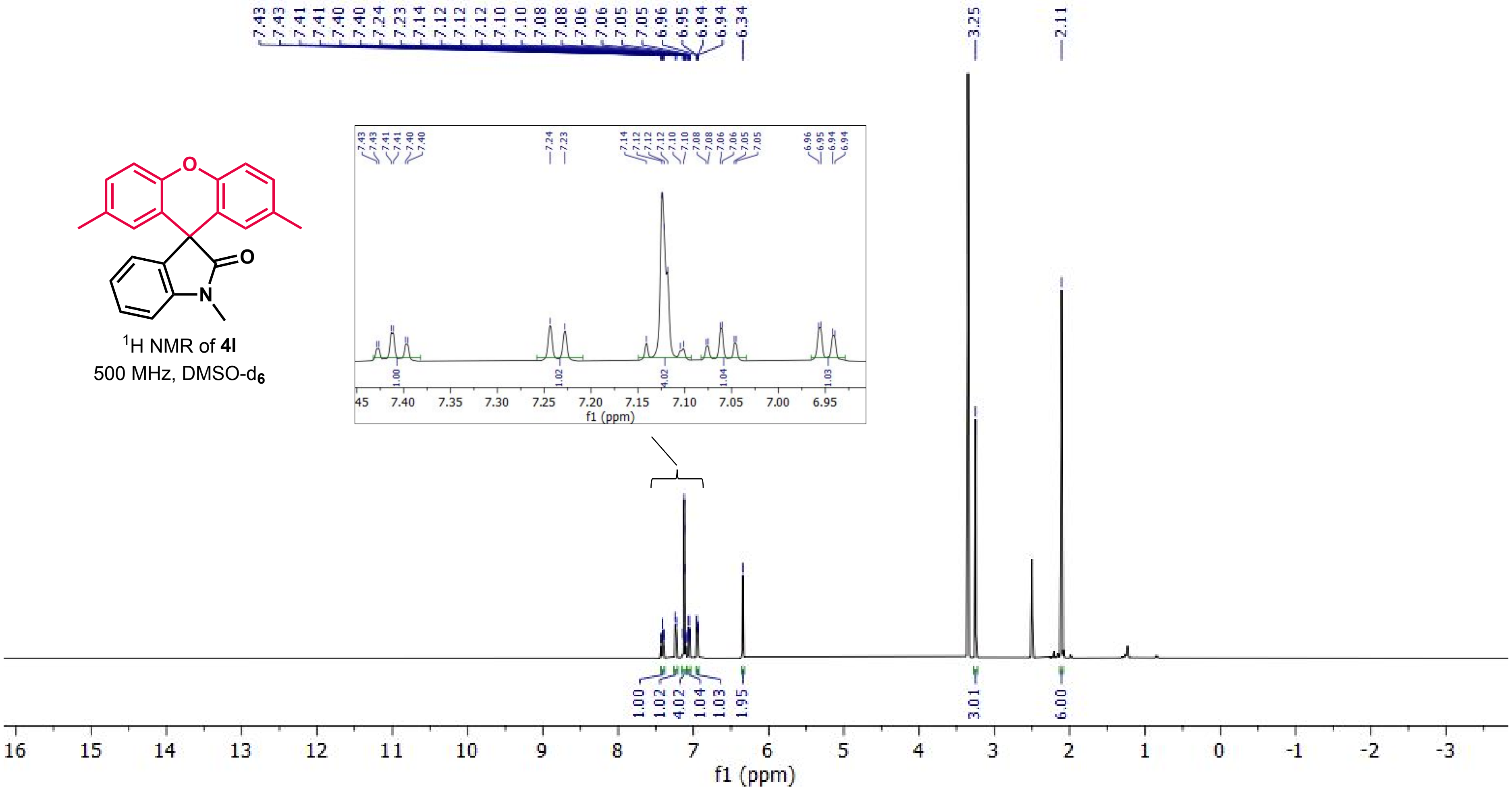

^{1}H NMR of **4l**
500 MHz, DMSO-d_6

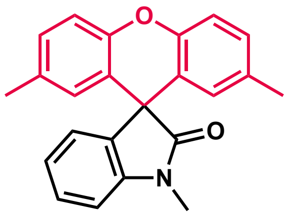

$^{13}C\{^1H\}$ NMR of **4l**
125.7 MHz, DMSO-d_6

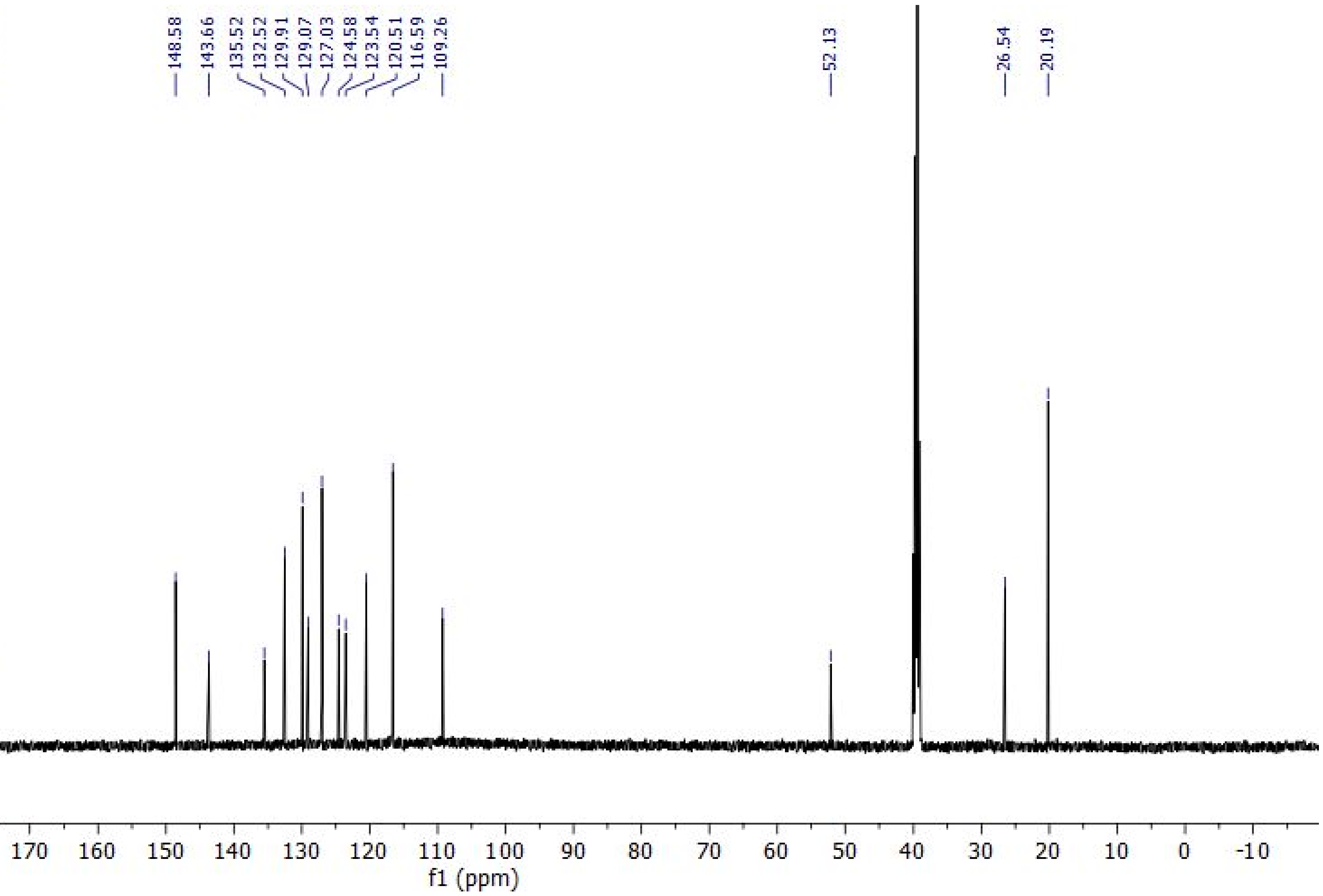

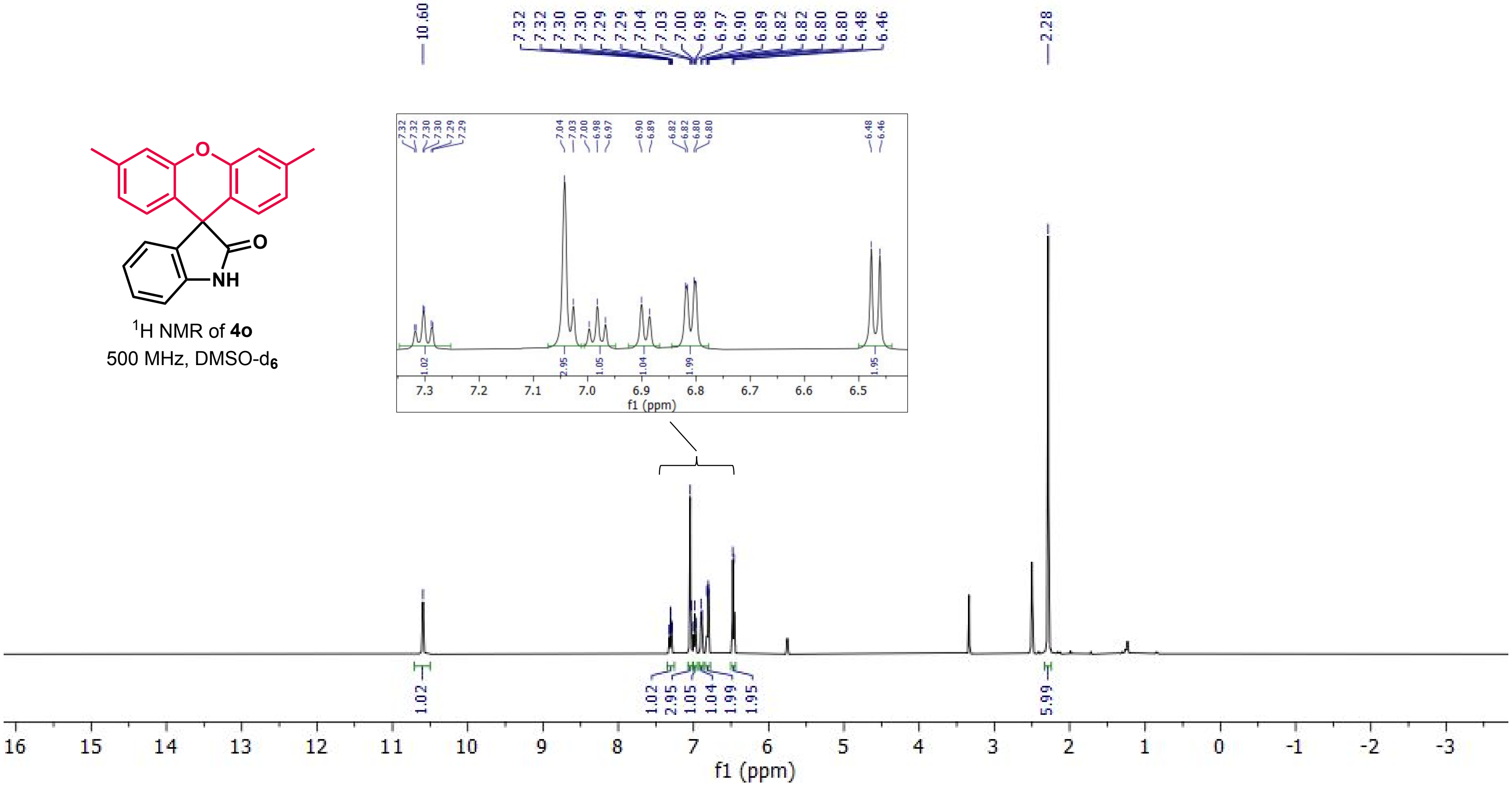

^{1}H NMR of **4o**

500 MHz, DMSO-d_6

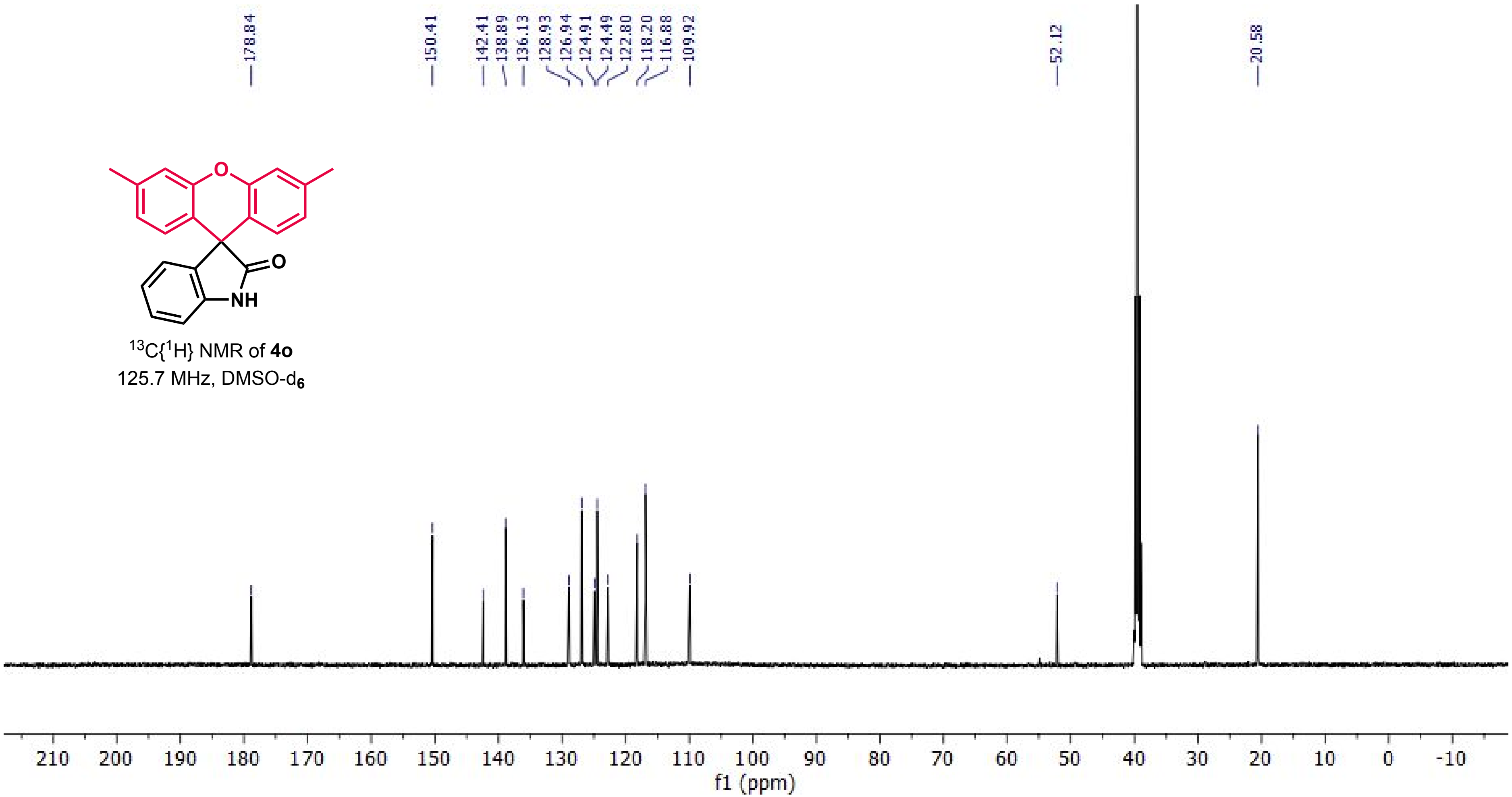
178.84
150.41
142.41
138.89
136.13
128.93
126.94
124.91
124.49
122.80
118.20
116.88
109.92
52.12
20.58
O
O
NH
$^{13}C\{^1H\}$ NMR of **4o**
125.7 MHz, DMSO-d_6
210 200 190 180 170 160 150 140 130 120 110 100 90 80 70 60 50 40 30 20 10 0 -10
f1 (ppm)

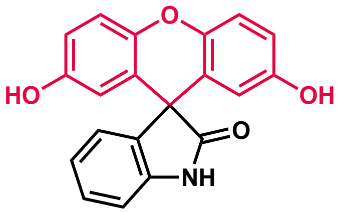

^{1}H NMR of **4q**
500 MHz, DMSO-d_6

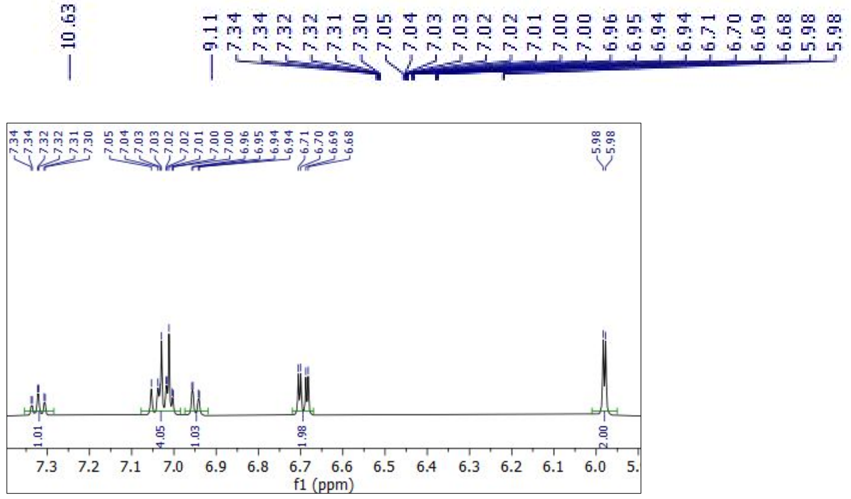

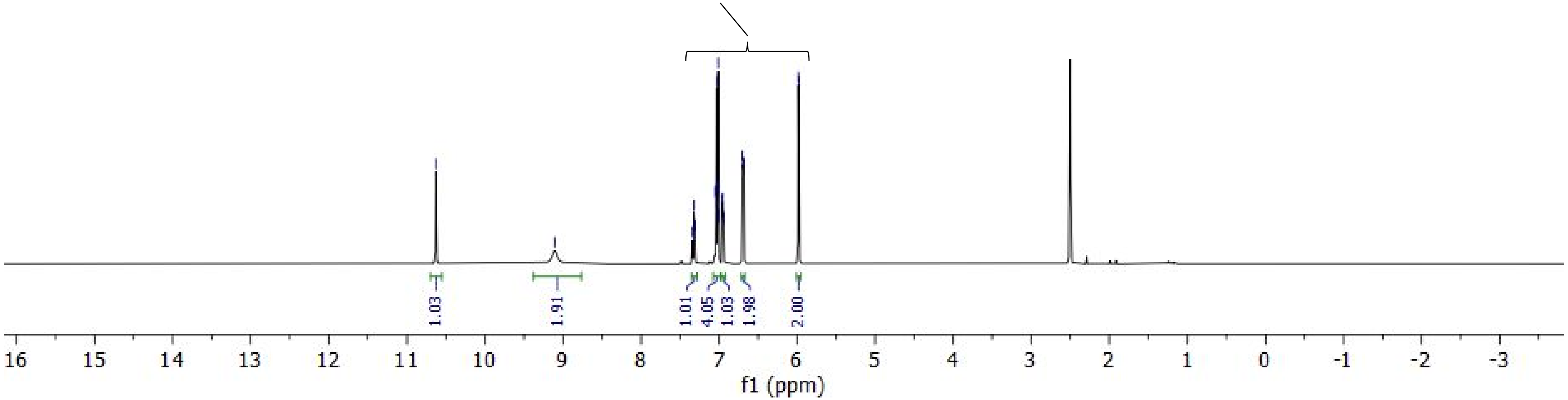

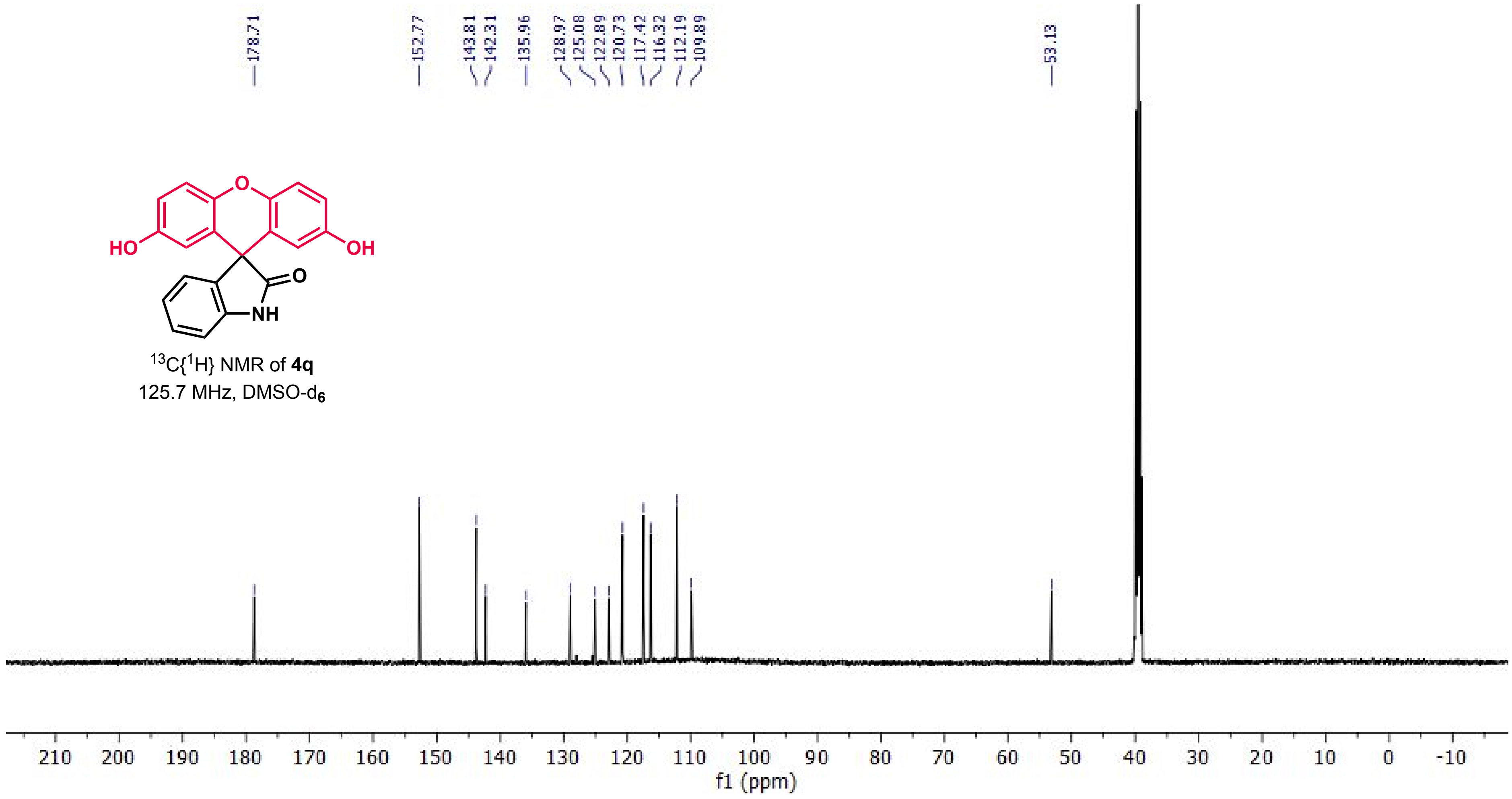

$^{13}C\{^{1}H\}$ NMR of **4q**

125.7 MHz, DMSO-d_6

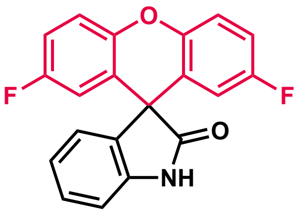

^{1}H NMR of **4s**
500 MHz, DMSO-d$_6$

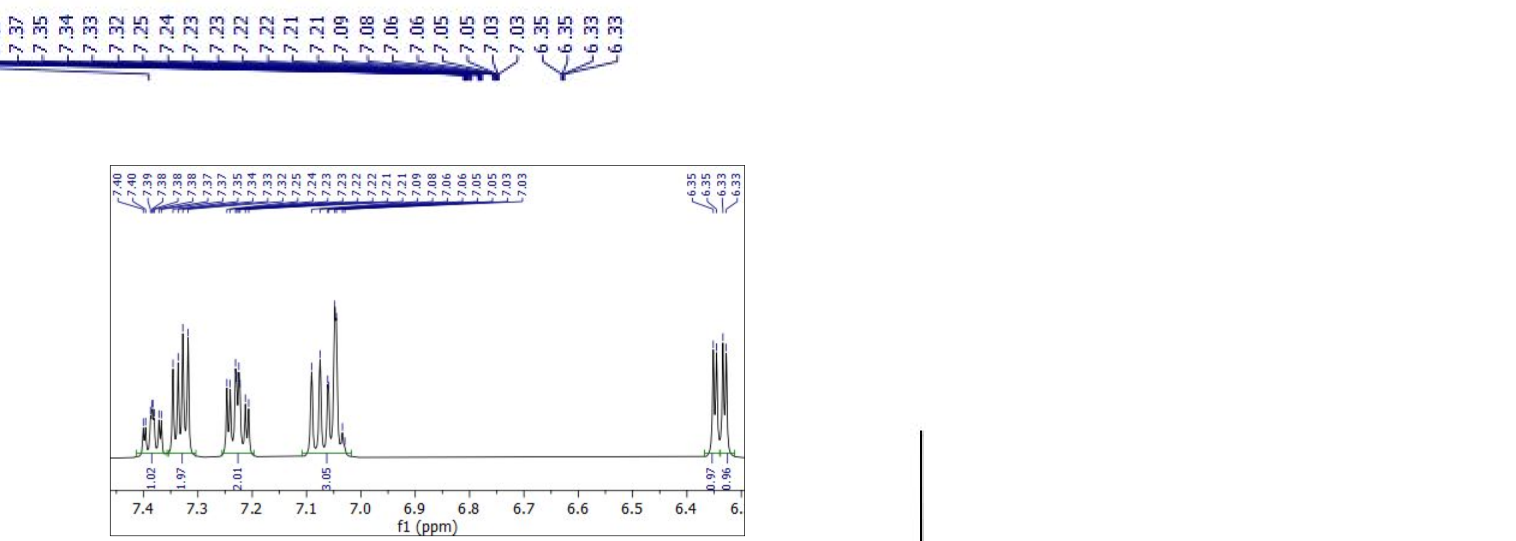

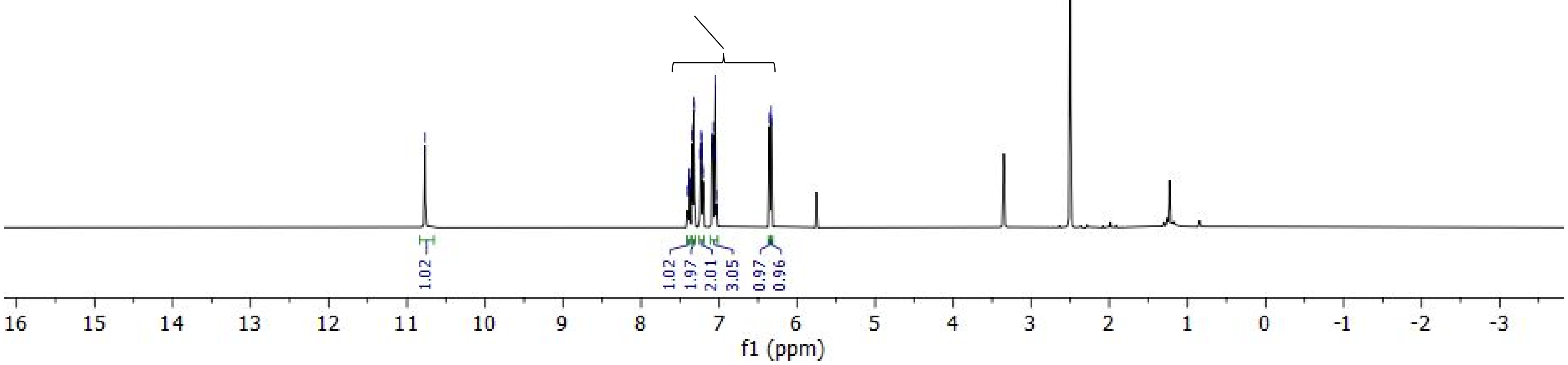

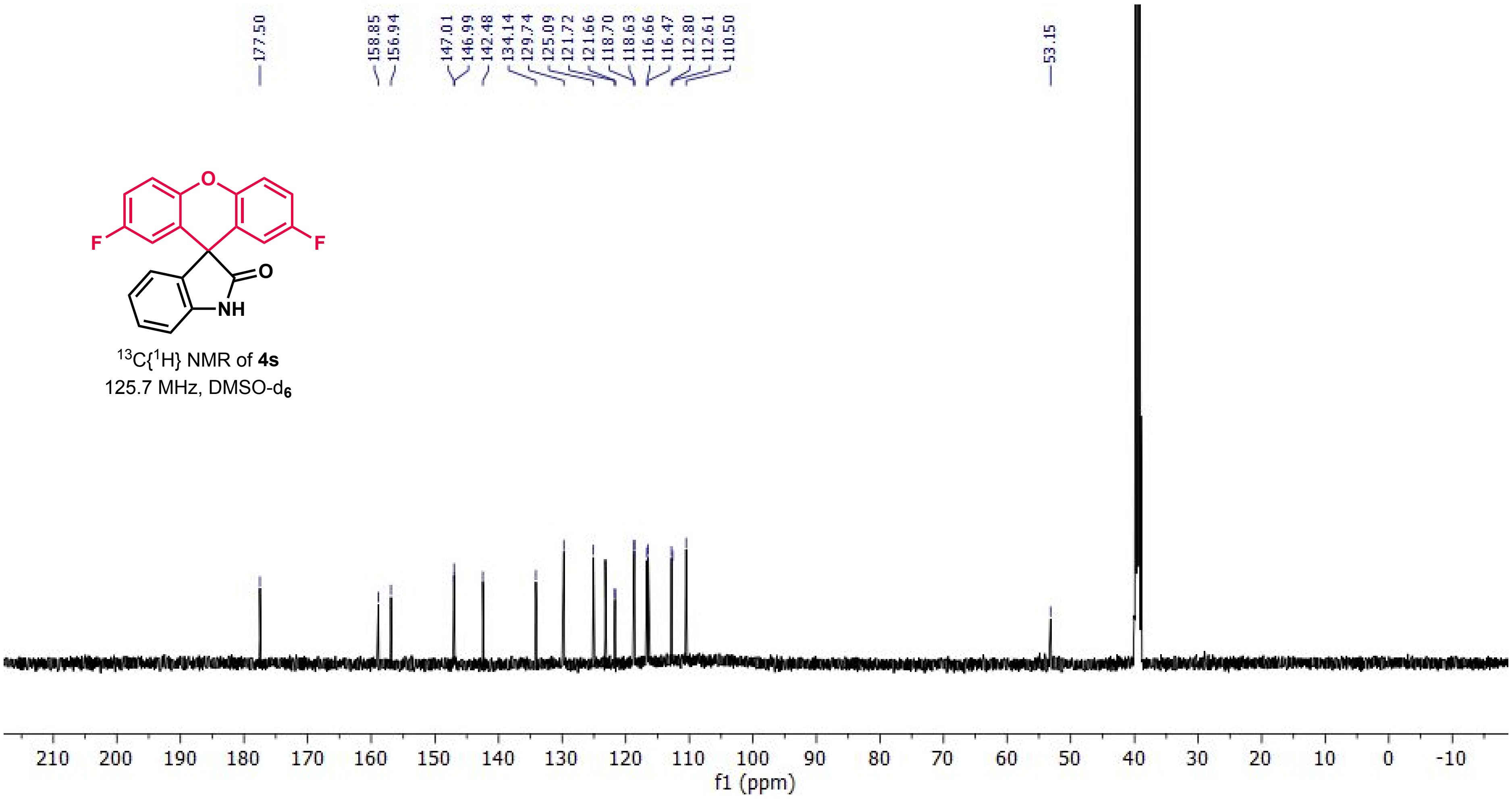

$^{13}C\{^{1}H\}$ NMR of **4s**
125.7 MHz, DMSO-d_6

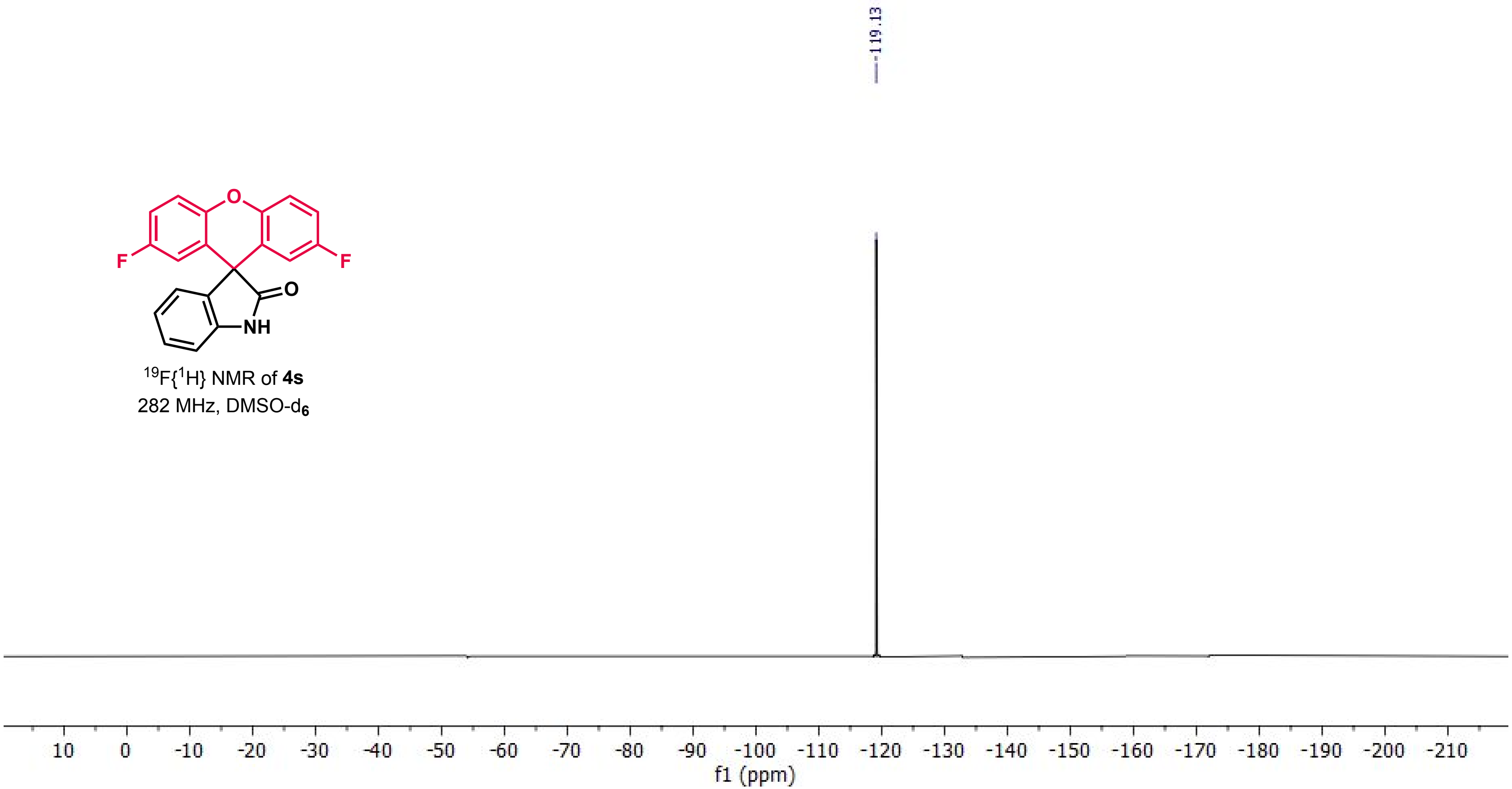

$^{19}F\{^{1}H\}$ NMR of **4s**
282 MHz, DMSO-d_6

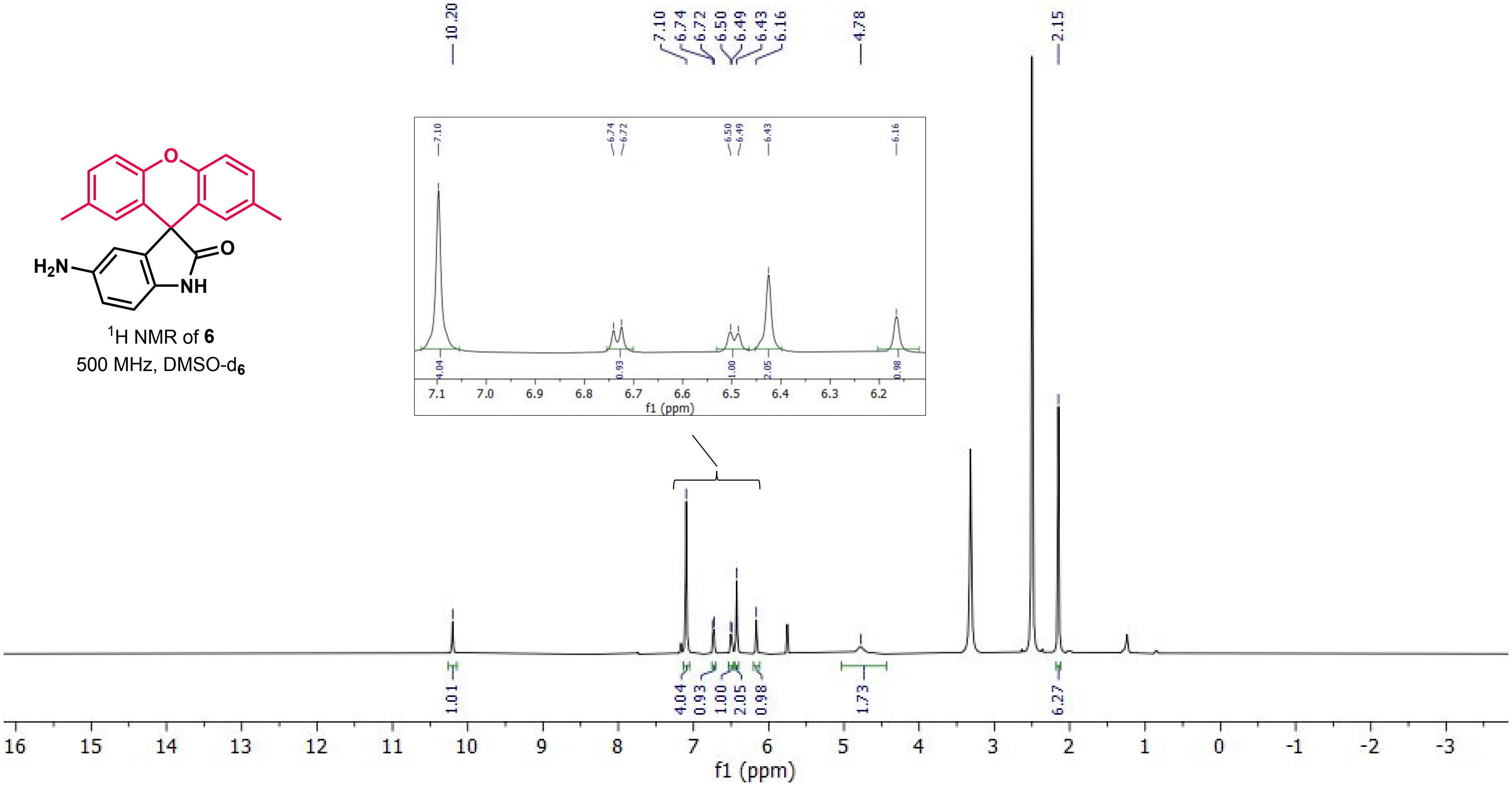

^{1}H NMR of **6**
500 MHz, DMSO-d$_6$
10.20
7.10
6.74
6.72
6.50
6.49
6.43
6.16
4.78
2.15
1.01
4.04
0.93
1.00
2.05
0.98
1.73
6.27
f1 (ppm)

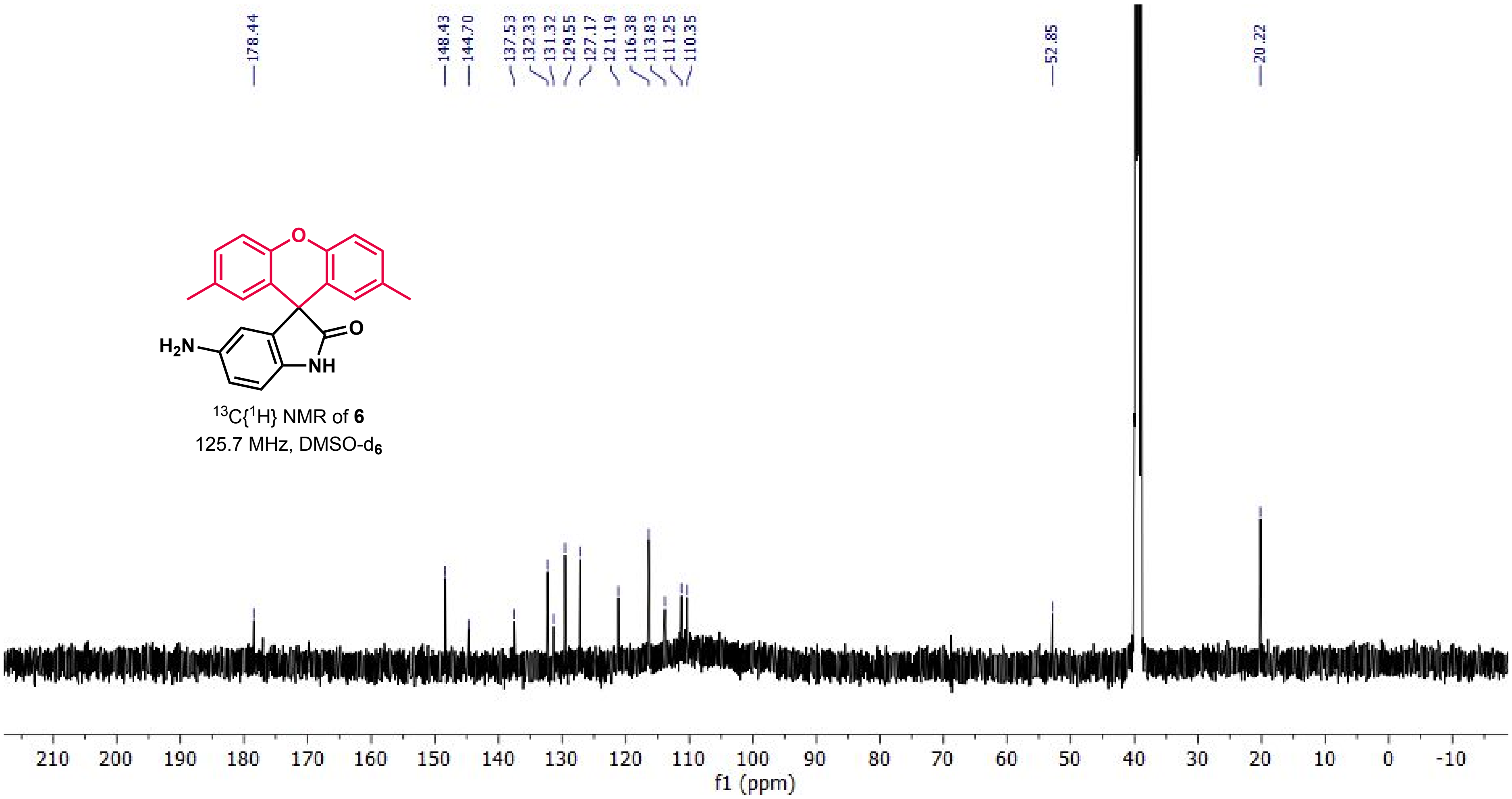

$^{13}C\{^1H\}$ NMR of **6**
125.7 MHz, DMSO-d_6

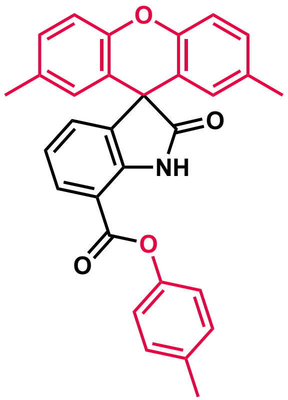

^{1}H NMR of **10**
500 MHz, DMSO-d$_6$

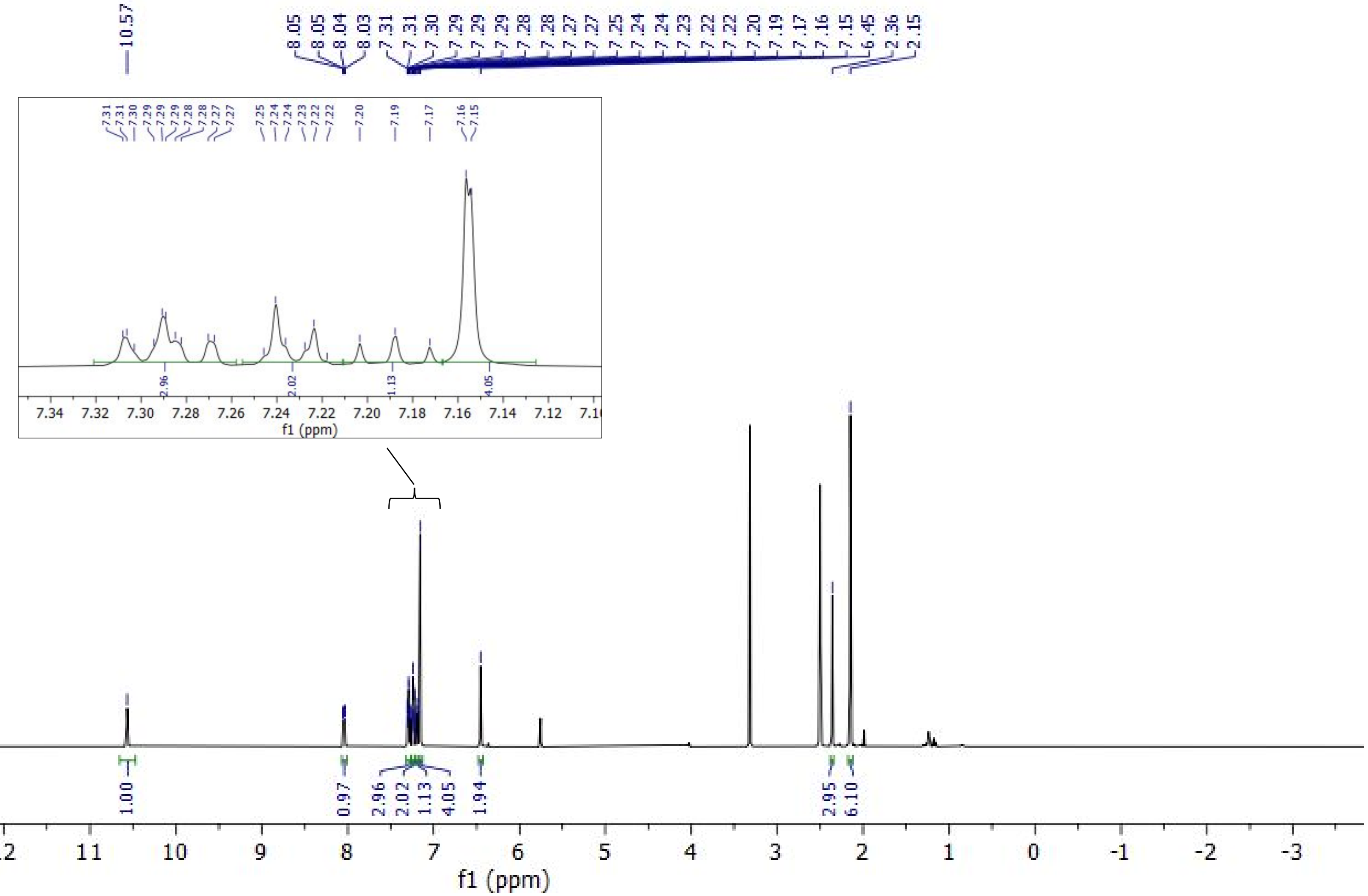

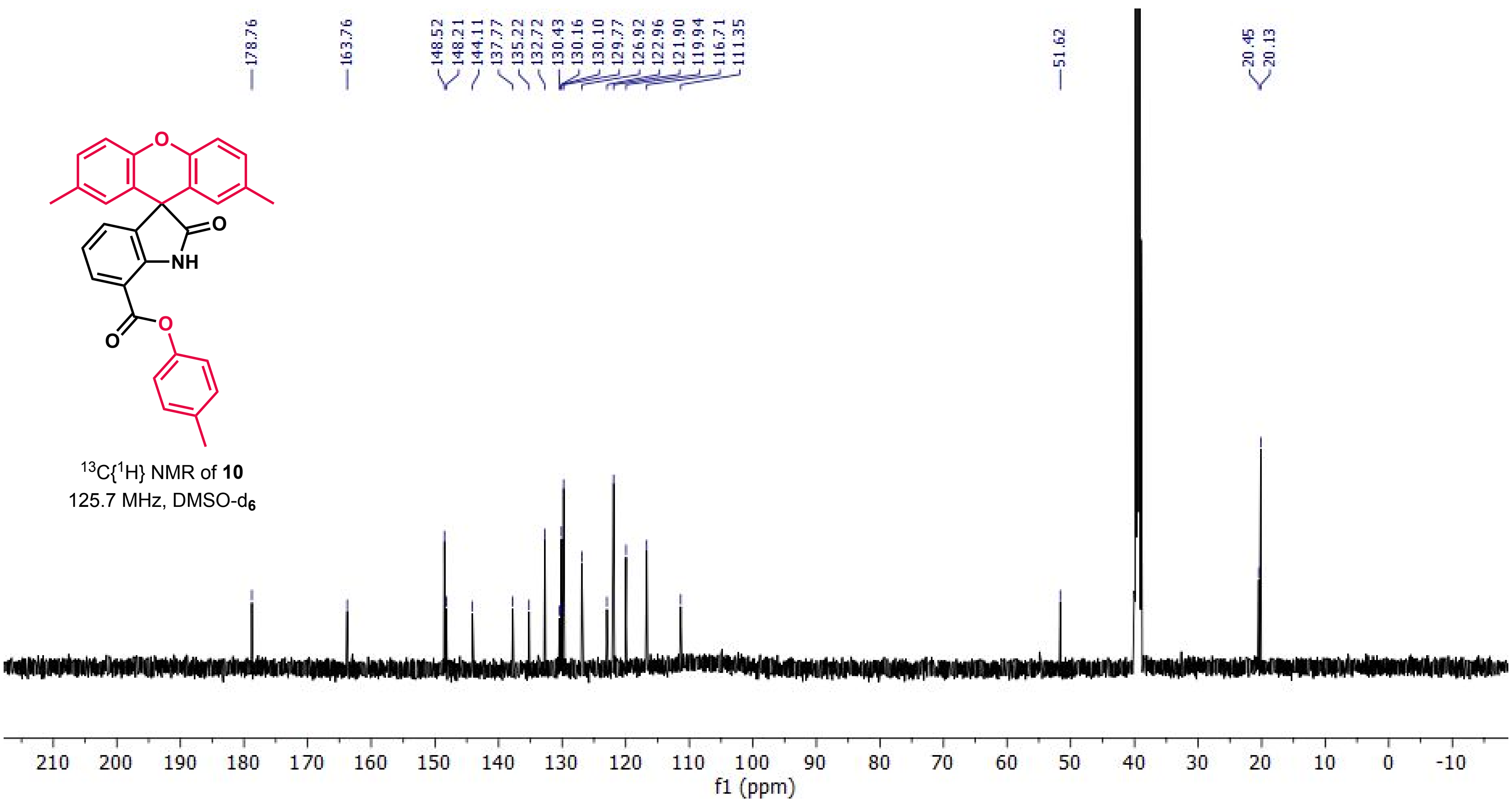

$^{13}C\{^{1}H\}$ NMR of **10**
125.7 MHz, DMSO-d_6

5. 2D NMR spectra of compound 3a

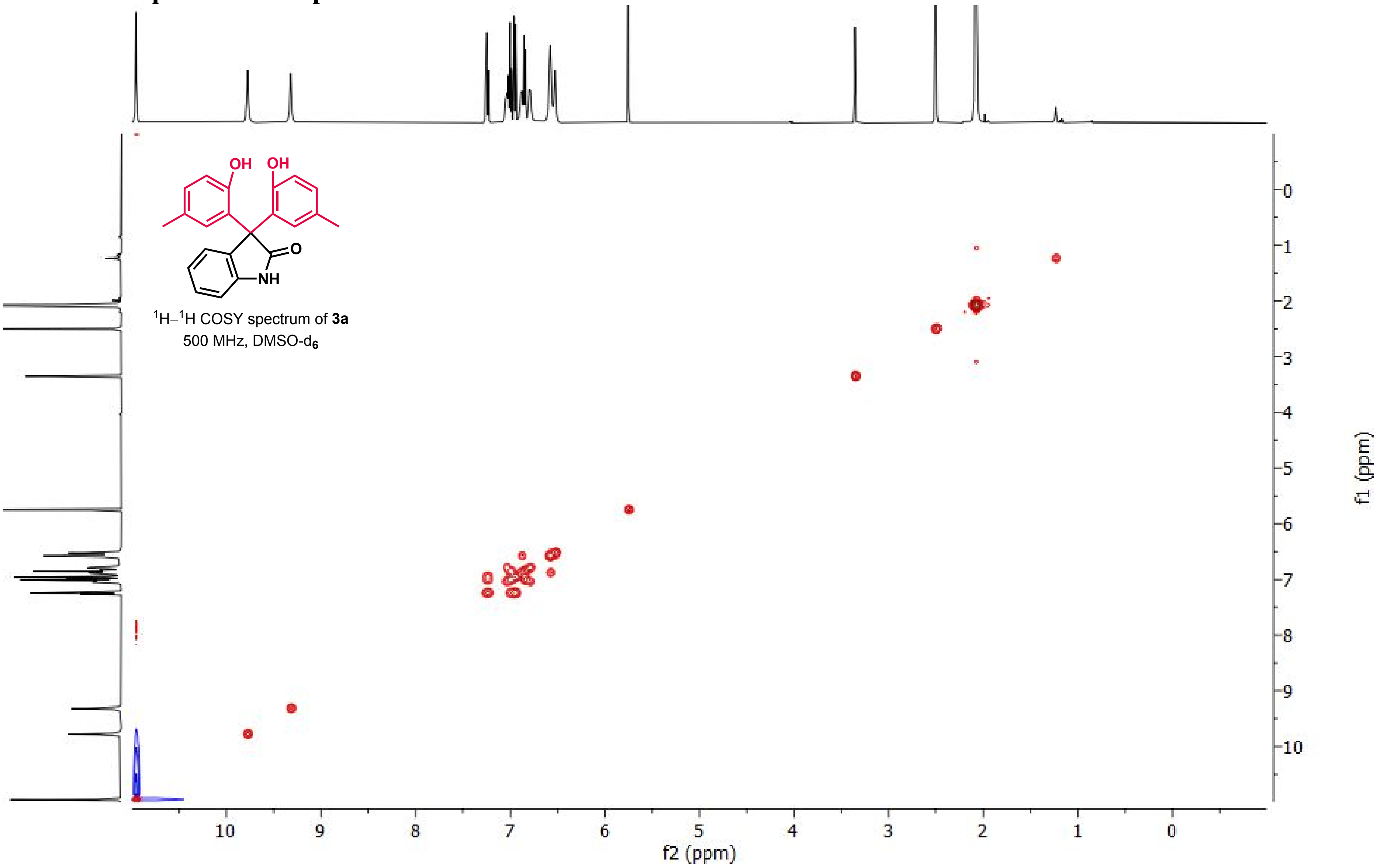

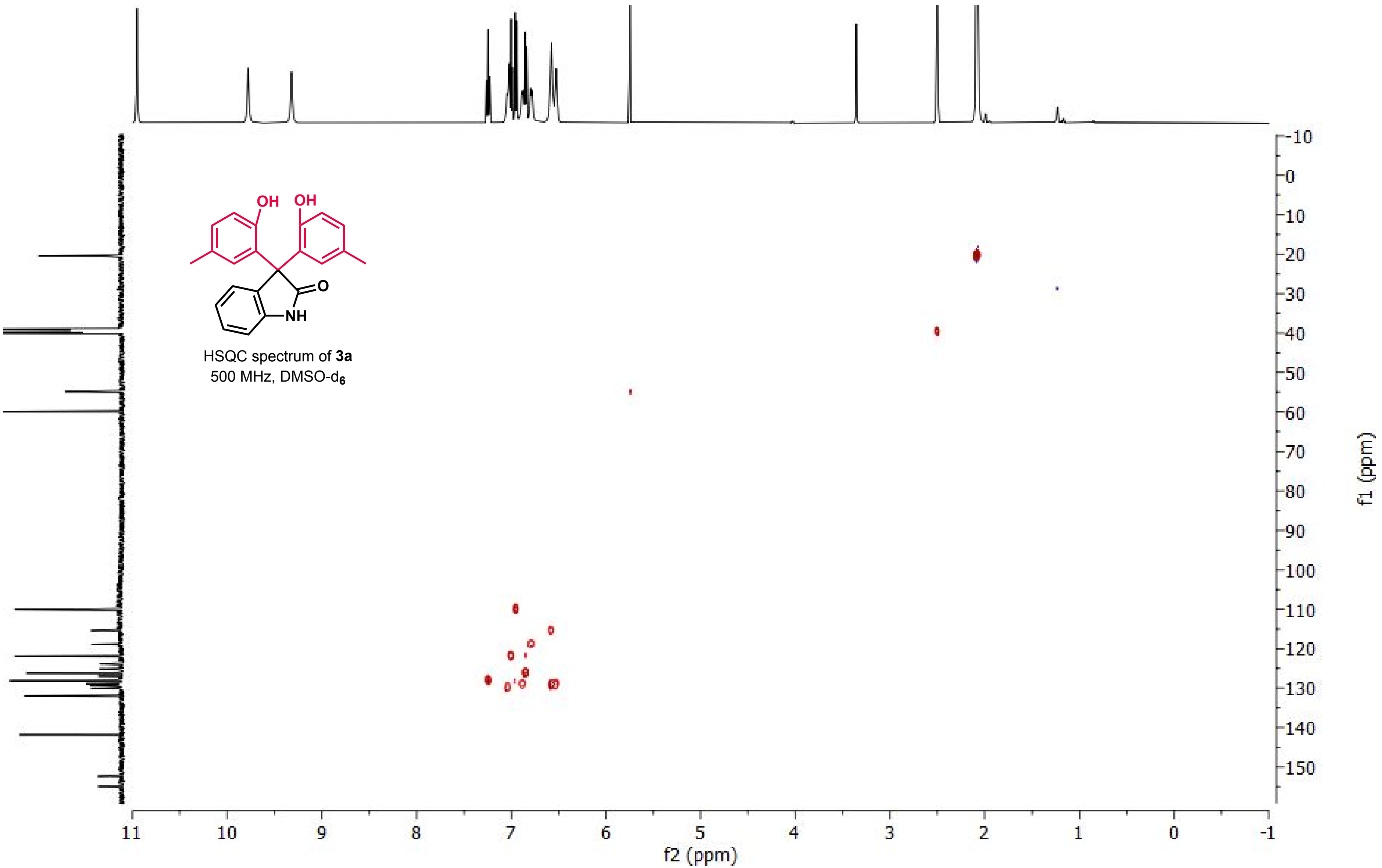
OH
OH
O
NH
HSQC spectrum of 3a
500 MHz, DMSO-d6
f2 (ppm)
f1 (ppm)

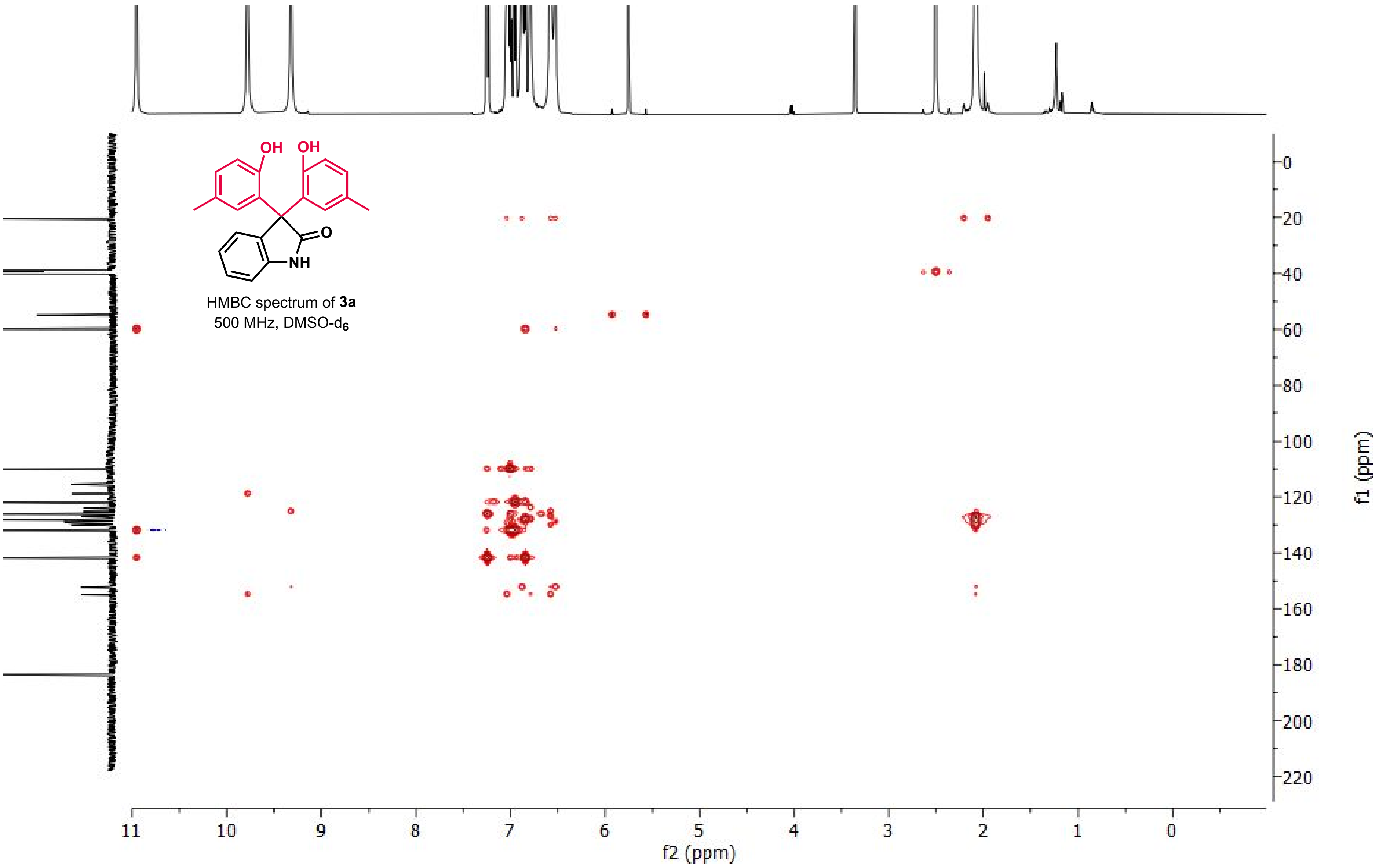
OH
OH
O
NH
HMBC spectrum of **3a**
500 MHz, DMSO-d_6
f1 (ppm)
f2 (ppm)

6. 2D NMR spectra of compound 4a

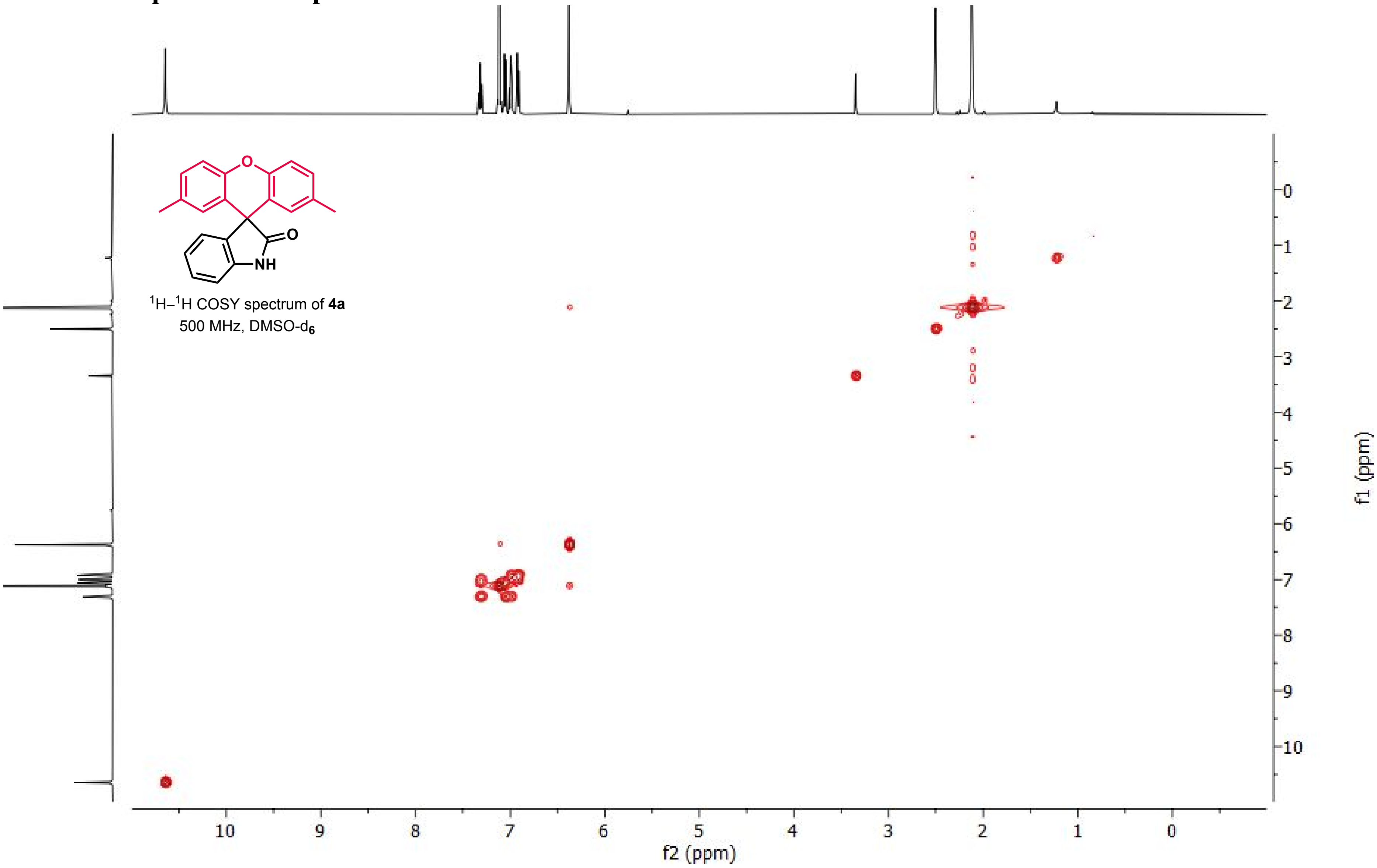

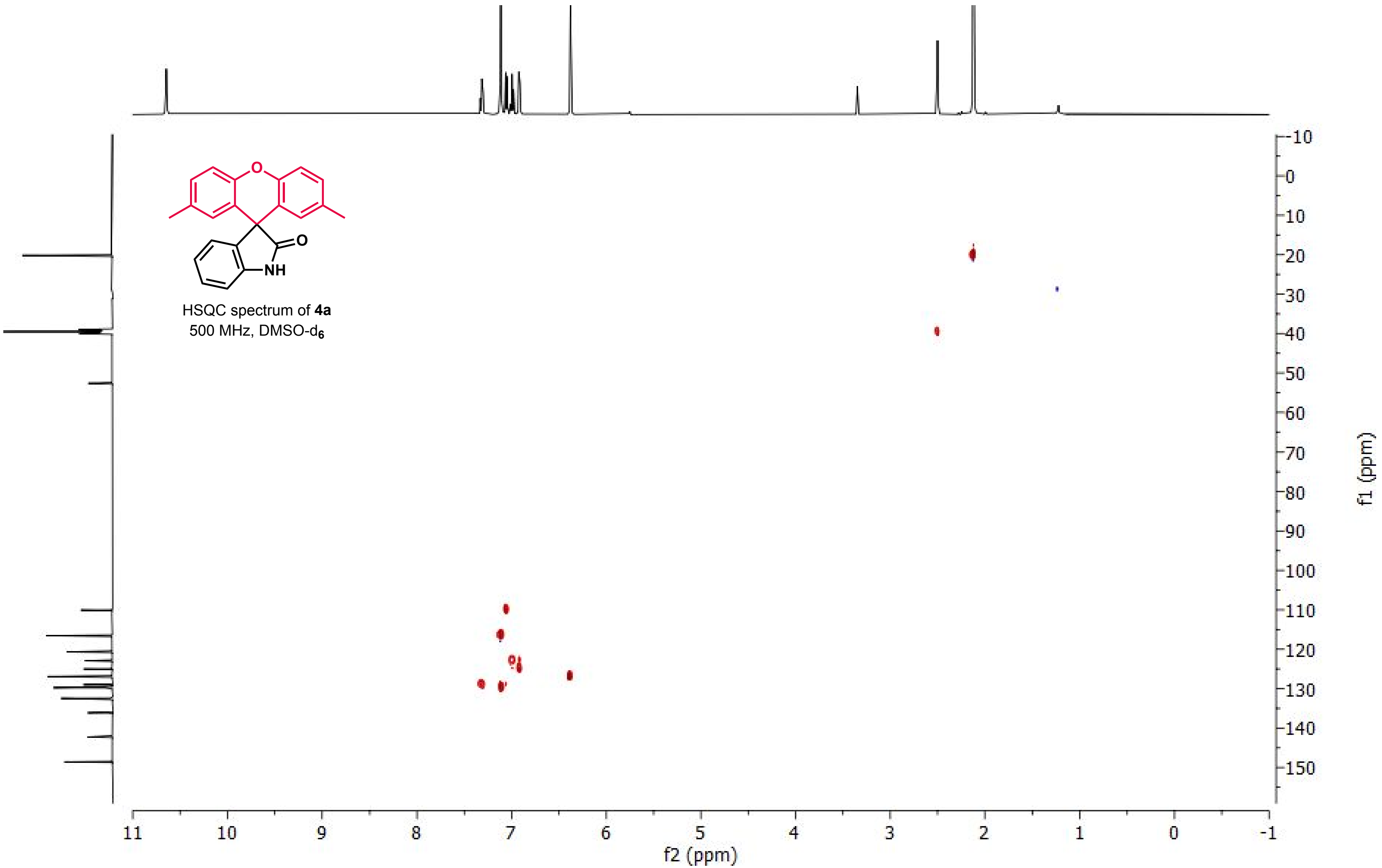
O
NH
O
HSQC spectrum of 4a
500 MHz, DMSO-d6
-10
0
10
20
30
40
50
60
70
80
90
100
110
120
130
140
150
f1 (ppm)
11
10
9
8
7
6
5
4
3
2
1
0
-1
f2 (ppm)

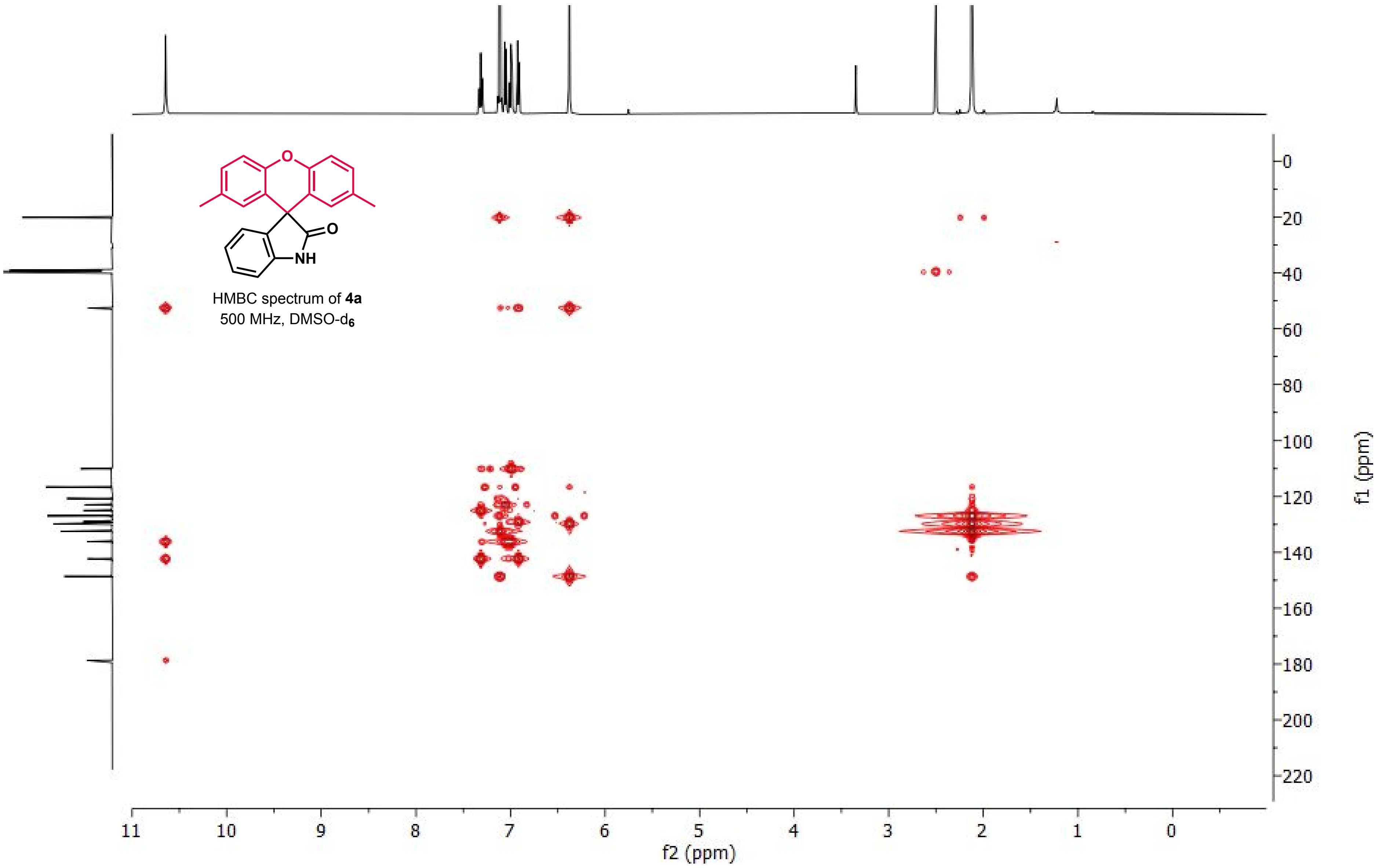

O
NH
HMBC spectrum of **4a**
500 MHz, DMSO-d6
f2 (ppm)
f1 (ppm)
