# Supplementary figures and images for: 3,3-Bis(hydroxyaryl)oxindoles and Spirooxindoles Bearing a Xanthene Moiety: Synthesis, Mechanism, and Biological Activity
Source: J Org Chem. 2025 May 7;90(19):6454–67. doi: 10.1021/acs.joc.5c00270 (PMC12090213; doi:10.1021/acs.joc.5c00270)

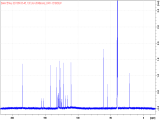

Supplement: Supplementary file 2 — jo5c00270_si_002.zip [file jo5c00270_si_002.zip › FID for Publication revised/FID/3a/3a-13C/pdata/1/thumb.png]

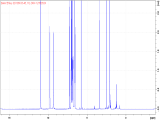

Supplement: Supplementary file 2 — jo5c00270_si_002.zip [file jo5c00270_si_002.zip › FID for Publication revised/FID/3a/3a-1H/pdata/1/thumb.png]

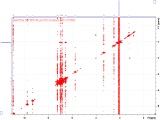

Supplement: Supplementary file 2 — jo5c00270_si_002.zip [file jo5c00270_si_002.zip › FID for Publication revised/FID/3a/3a-COSY/pdata/1/thumb.png]

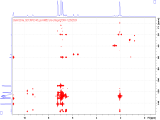

Supplement: Supplementary file 2 — jo5c00270_si_002.zip [file jo5c00270_si_002.zip › FID for Publication revised/FID/3a/3a-HMBC/pdata/1/thumb.png]

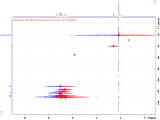

Supplement: Supplementary file 2 — jo5c00270_si_002.zip [file jo5c00270_si_002.zip › FID for Publication revised/FID/3a/3a-HSQC/pdata/1/thumb.png]

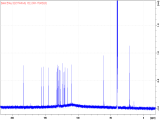

Supplement: Supplementary file 2 — jo5c00270_si_002.zip [file jo5c00270_si_002.zip › FID for Publication revised/FID/3b/3b-13C/pdata/1/thumb.png]

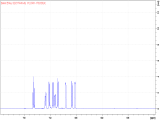

Supplement: Supplementary file 2 — jo5c00270_si_002.zip [file jo5c00270_si_002.zip › FID for Publication revised/FID/3b/3b-1H/pdata/1/thumb.png]

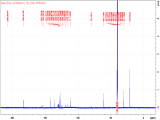

Supplement: Supplementary file 2 — jo5c00270_si_002.zip [file jo5c00270_si_002.zip › FID for Publication revised/FID/3c/3c-13C/pdata/1/thumb.png]
